# Supplementary material for: Transcription levels of a noncoding RNA orchestrate opposing regulatory and cell fate outcomes in yeast
Source: Cell Rep. 2021 Jan 19;34(3):108643. doi: 10.1016/j.celrep.2020.108643 (PMC7816125; doi:10.1016/j.celrep.2020.108643)
Supplement: Document S1. Figures S1–S6; Tables S1, S3, and S4; and Data S1 [file mmc1.pdf]

Cell Reports, Volume *34*

Supplemental Information

Transcription levels of a noncoding RNA orchestrate opposing regulatory and cell fate outcomes in yeast

Fabien Moretto, N. Ezgi Wood, Minghao Chia, Cai Li, Nicholas M. Luscombe, and Folkert J. van Werven

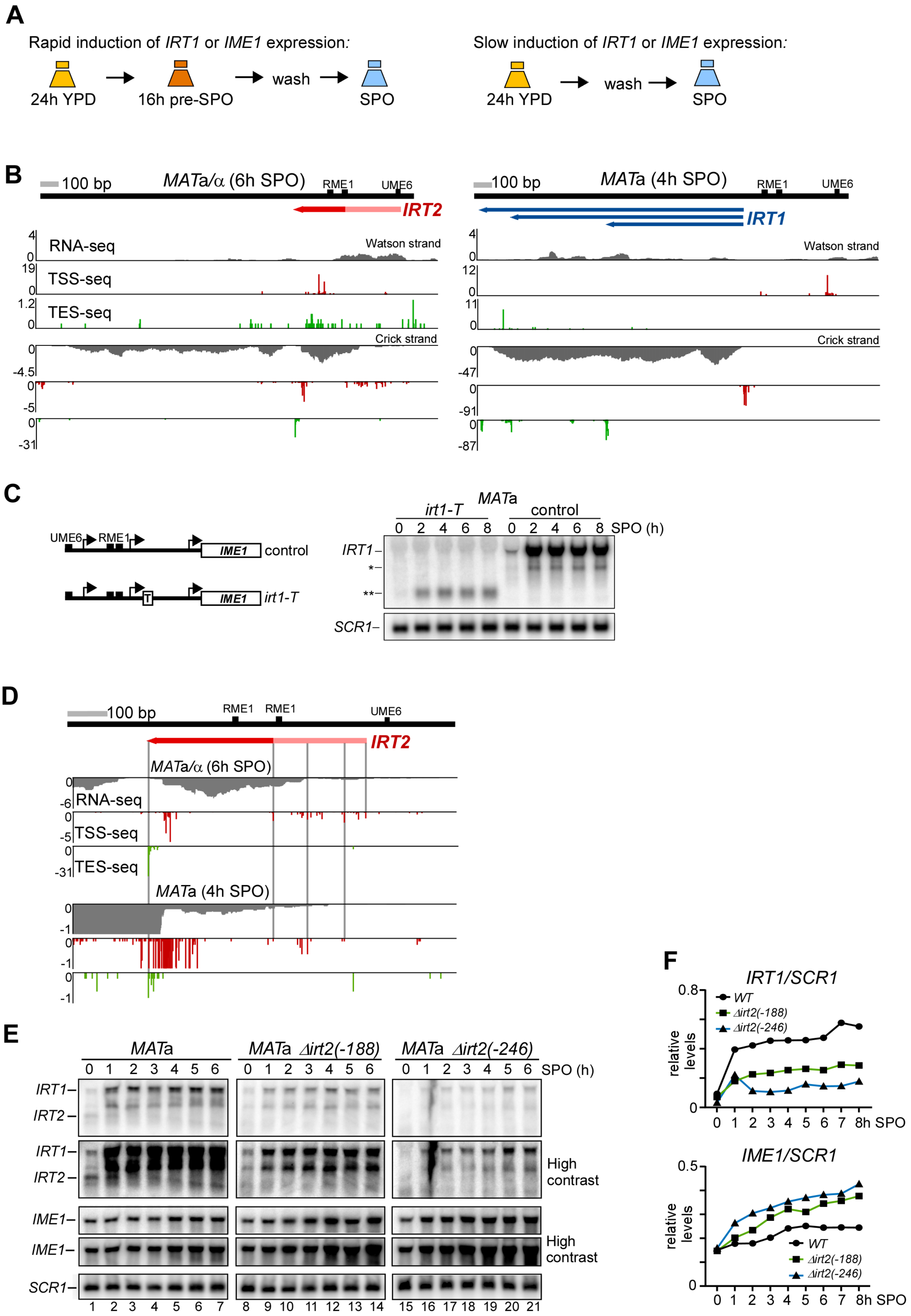
A
Rapid induction of IRT1 or IME1 expression:
24h YPD
16h pre-SPO
wash
SPO
Slow induction of IRT1 or IME1 expression:
24h YPD
wash
SPO
B
100 bp
MATa/α (6h SPO)
RME1
UME6
IRT2
RNA-seq
TSS-seq
TES-seq
Watson strand
Crick strand
MATa (4h SPO)
IRT1
C
UME6
RME1
IME1
control
irt1-T
MATa
SPO (h)
IRT1
SCR1
D
MATa/α (6h SPO)
MATa (4h SPO)
E
MATa
MATa Δirt2(-188)
MATa Δirt2(-246)
IRT2
IME1
High contrast
F
IRT1/SCR1
IME1/SCR1
WT
Δirt2(-188)
Δirt2(-246)
relative levels
8h SPO

Figure S1: related to Figure 1

***IRT2* is required for *IRT1* expression**

A, Scheme of growth conditions used throughout this study. Rapid induction of *IRT1* or *IME1* expression was achieved by growing cells in rich medium (YPD) till saturation (24 h), subsequently cells were shifted to pre-sporulation (pre-SPO) medium and grown for another 16 h. After a wash with sterile water, cells were transferred into sporulation medium (SPO). Slow induction of *IRT1* or *IME1* expression was achieved by shifting cells directly after growth till saturation in YPD to SPO.

B, RNA-seq, TSS-seq and TES-seq tracks of *IRT2* and *IRT1* in *MAT*a/α 6h in SPO (FW1511) and *MAT*a 4h in SPO (FW1509) cells. Displayed are the signals of normalized reads for both the Watson and Crick strands. Cells were grown as described in A (Rapid induction of *IRT1*).

C, *IRT1* expression as detected by northern blot in control (FW1509) and *irt1-T* strains (FW155). The *irt1-T* strain harbors a transcriptional terminator ~ 200 bp after the *IRT1* TSS. Cells were grown as described in A (Rapid induction of *IRT1*). *SCR1* was used as a loading control.

D, Similar data as in B, except that *IRT2* is highlighted, which demonstrates that *IRT2* is transcribed in *MAT*a cells (4h in SPO).

E, *IRT1*, *IRT2*, and *IME1* expression in WT *MAT*a cells (FW1509), and Δ*irt2*(-188) (FW1210) and Δ*irt2*(-246) (FW1356) mutants as presented in Figure 1E. Images with adjusted signals for both *IRT1-IRT2* combined probe and *IME1* blots are also included.

F, Northern blot quantification of *IRT1* (top) and *IME1* (bottom) signals from the blots presented in E. Signals from the blots were background corrected and normalised over loading control signal *SCR1*.

Moretto *et al*. Figure S2

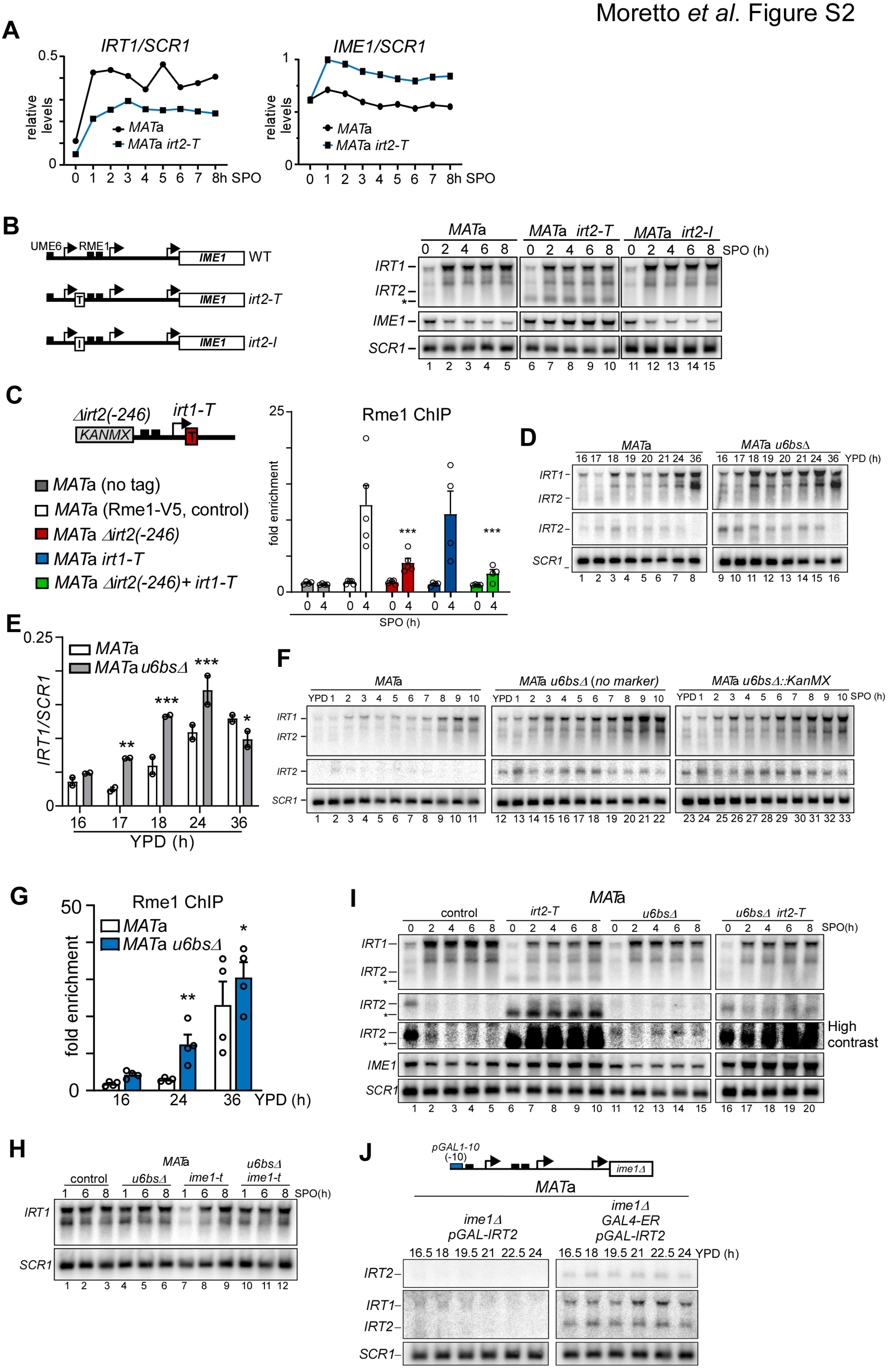

Figure S2: related to Figure 2

***IRT2* transcription is required and sufficient for inducing *IRT1* expression**

A, Northern blot quantification of *IRT1* (left) and *IME1* (right) signals from the blots presented in 2B. Signals were background corrected and normalised over loading control signal *SCR1*.

B, Scheme of the *IME1* locus harboring a transcriptional terminator *(irt2-T)* or a control insert (*irt2-I*) in *IRT2* (left). *IRT1*, *IRT2*, and *IME1* expression in WT *MAT*a cells (FW1509), Δ*irt2-T* (FW3596) and Δ*irt2-I* (FW4175) as detected by northern blot (right). *SCR1* was used as a loading control. The asterisk depicts the prematurely terminated form of *IRT2*. Cells were grown in YPD to saturation (24 h), shifted to pre-SPO (16 h), and subsequently transferred to SPO.

C, Rme1 ChIP followed by qPCR using oligos nested in *IRT2* in no tag control, *RME1-V5* control, *Δirt2(-246)* and *irt1-T* single mutants and *Δirt2(-246) irt1-T* double mutant (FW1509, FW4031, FW3140, FW155 and FW8909). Cells were grown as in B. qPCR were performed using a primer pair directed to Rme1 binding sites in the *IRT1* promoter. Signals were normalized to *HMR*. The error bars represent the standard error of the mean (SEM) of n = 3 biological repeats. *** correspond to a p-value < 0.0005 on a two-way ANOVA analysis followed by a Fisher's LSD test comparing the mutant strains with the control strain.

D, *IRT1* and *IRT2* expression in WT (FW1509) and *u6bs*Δ *MATa* cells (FW2438). Cells were grown in YPD to saturation and sampled at the indicated time points.

E, Quantification of *IRT1* expression described in D. *IRT1* signals were normalized over *SCR1*. n = 2 +/- SEM. *, ** and *** correspond to p-value < 0.05, < 0.005, < 0.0005 respectively on a two-way ANOVA analysis followed by a Fisher's LSD test.

F, *IRT1* and *IRT2* expression in *MAT*a cells (FW1509) and *u6bs*Δ cells harboring the deletion of the Ume6 binding site in absence of a selectable marker *u6bs*Δ (FW1378) or with a selectable marker *u6bsΔ::KanMX* (FW2438). Cells were grown in YPD to saturation and subsequently transferred to SPO.

G, ChIP of Rme1-V5 at *IRT1* under the same condition described in D, except for the presence of the *RME1-V5* allele in *MAT*a WT (FW4031) and *u6bs*Δ (FW3144). n = 4 +/- SEM. * and ** correspond to a p-value < 0.05 and < 0.005 respectively on a two-way ANOVA followed by Fisher's LSD test comparing *u6bs*Δ to the *MAT*a control.

H, *IRT1* and *IRT2* expression in *MAT*a cells (FW1509), *u6bsΔ::KanMX* (FW2438), *ime1-t* (FW2189) and *u6bsΔ::KanMX ime1-t* double mutant (FW2539). Cells were grown as in B.

I, *IRT1*, *IRT2*, and *IME1* expression in *MAT*a cells (FW1509), Δ*irt2-T* (FW3596), *u6bsΔ::KanMX* (FW2438) and *u6bsΔ::KanMX* Δ*irt2-T* double mutant (FW3493). Cells were grown as in B. An image presenting the adjusted signal highlighting *IRT2* expression from the northern blot is included.

J, *IRT1* and *IRT2* expression in *MAT*a cells harboring *ime1*Δ and the *GAL1-10* promoter integrated -10 bp from the *Ume6* binding site at *IRT2* site (*pGAL-IRT2*) (FW8759), or the same cells also expressing *GAL4-ER* (FW8758). Samples were taken from cells grown in YPD till the indicated time-points.

Moretto *et al.* Figure S3

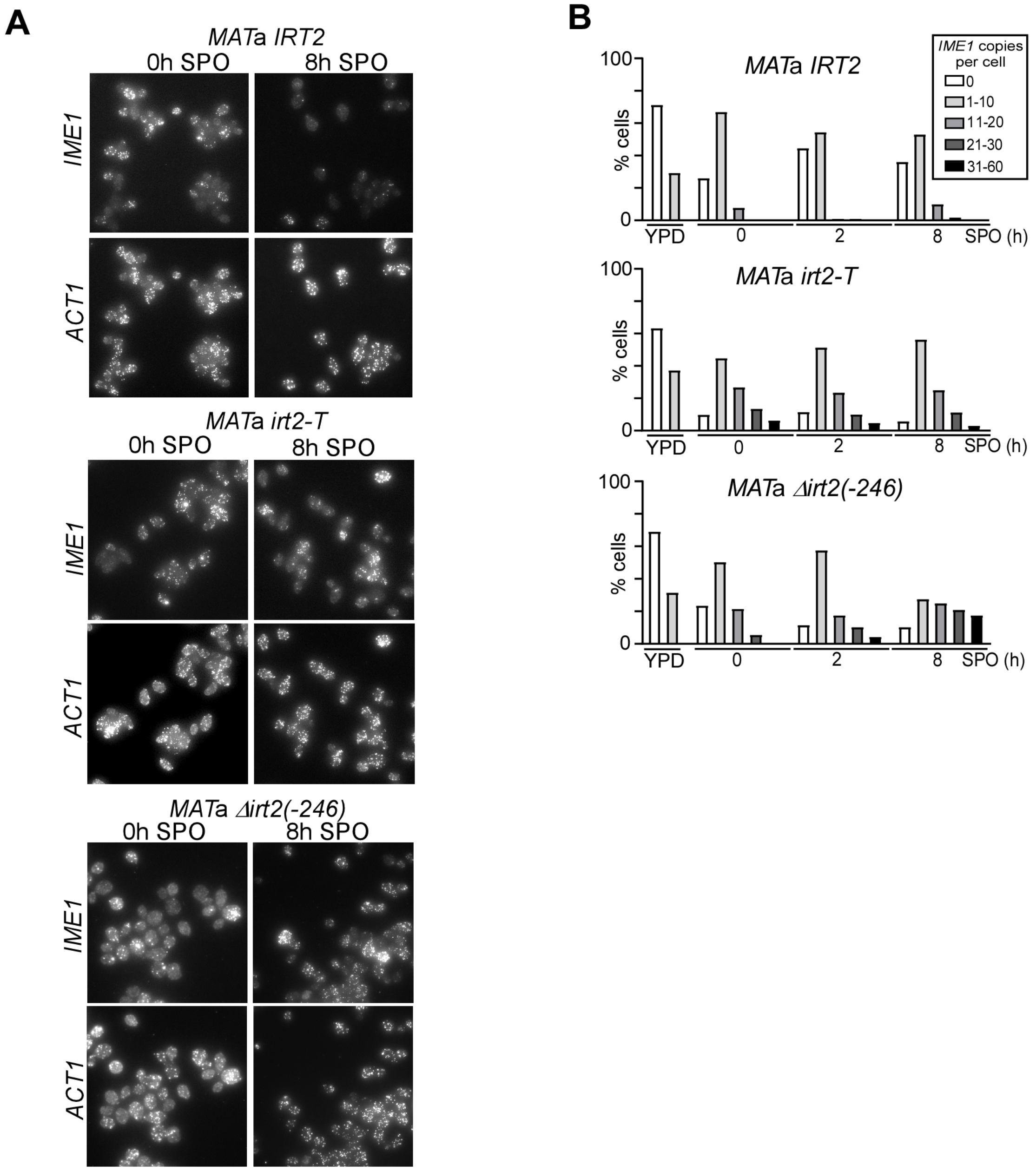

Figure S3: related to Figure 3

***IRT2* prevents entry into gametogenesis in cells with a single mating-type**

A, Representative images of *IME1* and *ACT1* expression in single cells as measured by single-molecule RNA FISH in control *MAT*a (FW1533), *irt2-T* (FW3585) and ∆*irt2*(-246) (FW3580). Cells were grown overnight to saturation in rich medium (YPD, 24 h), shifted to pre-SPO and grown for an addition 16 hours, and subsequently transferred to SPO. Cells were fixed at the indicated time points and hybridized with probes directed against *IME1* and *ACT1*.

B, Distribution of *IME1* expression in single cells. The data described in Figure 3A were binned according to the number of *IME1* copies per cell.

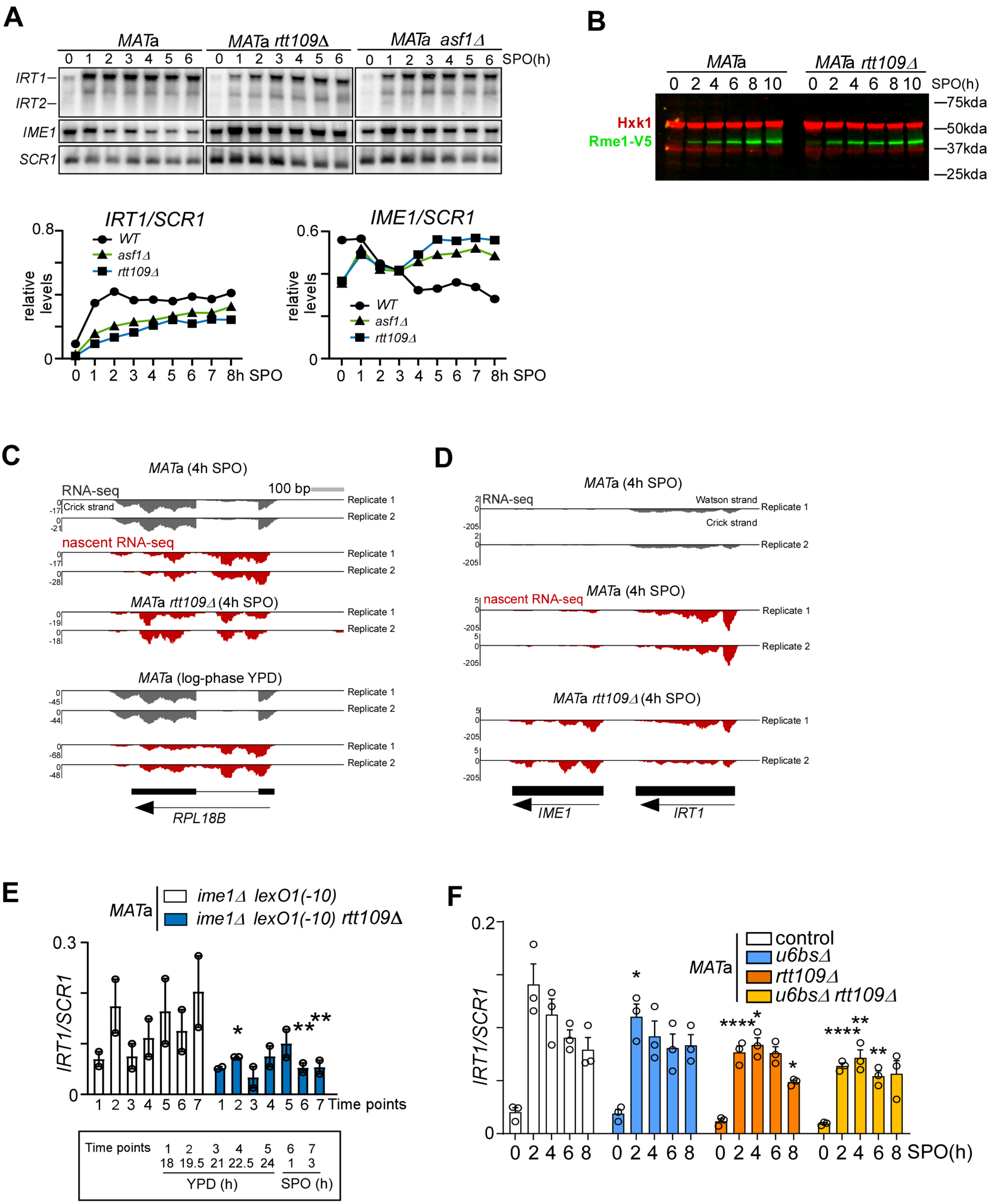

Time points	1	2	3	4	5	6	7
	18	19.5	21	22.5	24	1	3
	YPD (h)					SPO (h)	

Figure S4: related to Figure 4

Rtt109 and Asf1 promote *IRT1* transcription

A, *IRT1*, *IRT2*, and *IME1* expression in *MAT*a control (FW1509), *rtt109*Δ (FW4077) and *asf1Δ* (FW4521) cells as detected by northern blot (top). *SCR1* was used as loading control. Cells were grown in rich medium (YPD, 24 h) to saturation, shifted in pre-SPO (16 h), and subsequently transferred to SPO. Northern blot quantifications of the *IRT1* and *IME1* signals over SCR1 signals, are also presented (bottom).

B, Rme1-V5 control *MAT*a cells (FW4031) and *rtt109*Δ mutant (FW4075), as detected by western blot using anti-V5 (green channel). Hxk1 was used as loading control using anti-Hxk1 (red channel) antibodies. Cells were grown as described in A and samples were taken at the indicated time points.

C, Nascent RNA-seq data for *RPL18B*. Two replicates of RNA-seq and nascent RNA-seq (Pol II associated RNA) are displayed. Cells were harvested in SPO 4 h and during exponential growth (log phase YPD) for *MAT*a control, and SPO 4 h for *MAT*a *rtt109Δ*. Subsequently, Pol II was purified using the Rpb3-FLAG. The values on the y-axes are in Reads Per Million (RPM).

D, Similar to C, except that *IRT1* and *IME1* transcription data are displayed.

E, Northern blot quantification of *IRT1* expression for data shown in Figure 4F. *IRT1* expression was normalised over *SCR1*. n = 2 +/- SEM. * and ** correspond to p-value < 0.05 and <0.005 respectively on a two-way ANOVA followed by a Fisher's LSD test.

F, Northern blot quantification of *IRT1* expression for data shown in Figure 4G. *IRT1* expression was normalized over *SCR1*. n = 3 +/- SEM. *, ** and **** correspond to p-value < 0.05, < 0.005 and < 0.0001 respectively on a two-way ANOVA followed by a Fisher's LSD test.

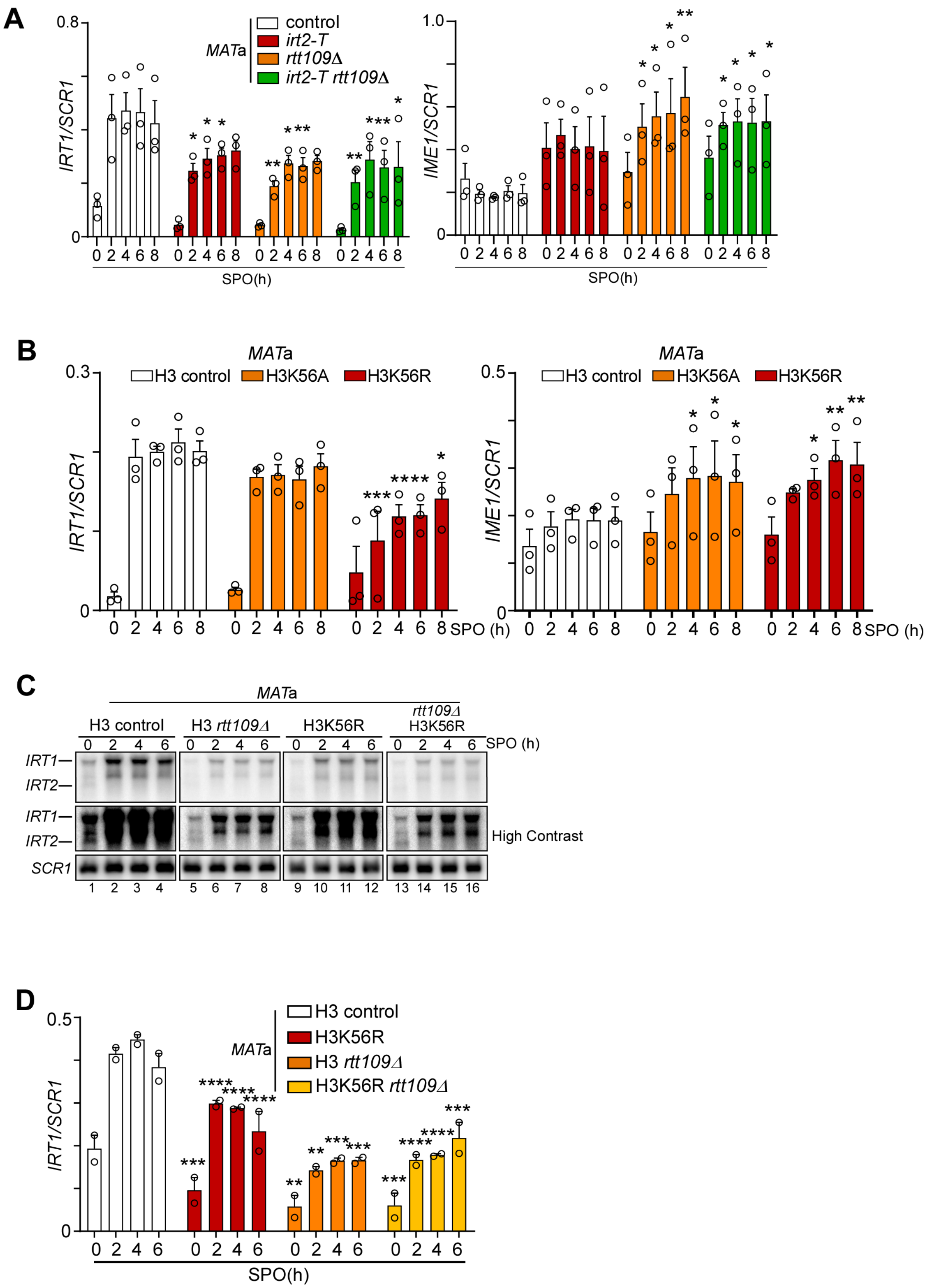
A
IRT1/SCR1
IME1/SCR1
MATa
control
irt2-T
rtt109Δ
irt2-T rtt109Δ
SPO(h)
B
MATa
H3 control
H3K56A
H3K56R
IRT1/SCR1
IME1/SCR1
SPO (h)
C
MATa
H3 control
H3 rtt109Δ
H3K56R
rtt109Δ H3K56R
SPO (h)
IRT1
IRT2
SCR1
High Contrast
D
MATa
H3 control
H3K56R
H3 rtt109Δ
H3K56R rtt109Δ
IRT1/SCR1
SPO(h)

Figure S5: related to Figure 5

***IRT2* transcription directs Histone H3 lysine 56 acetylation to chromatin locally to activate *IRT1* transcription**

A, Quantifications of *IRT1* and *IME1* expression for data shown in Figure 5A. *IRT1* and *IME1* signals were normalized over *SCR1*. n = 3, +/- SEM. *, ** and *** correspond to p-value < 0.05, < 0.005 and < 0.0005 respectively on a two-way ANOVA followed by a Fisher's LSD test.

B, Quantifications of *IRT1* and *IME1* expression for data shown in Figure 5D. *IRT1* and *IME1* signals were normalized over *SCR1*. n = 3, +/- SEM. *, **, *** and **** correspond to p-value < 0.05, < 0.005, < 0.0005 and < 0.0001 respectively on a two-way ANOVA followed by a Fisher's LSD test.

C, *IRT1* and *IRT2* expression in control *MAT*a H3 (FW5102), H3 *rtt109*Δ (FW6443), *H3K56R* (FW5116) and *rtt109*Δ *H3K56R* double mutant (FW5724) cells as detected by northern blot. *SCR1* was used as loading control. An image presenting an adjusted signal highlighting *IRT2* expression from the northern blot is also included. Cells were grown in rich medium (YPD, 24 h) to saturation, shifted and grown in pre-SPO (16 h), and subsequently transferred to SPO.

D, Quantification of *IRT1* expression as described in C. *IRT1* signals were normalized to *SCR1*. n = 2, mean values and data range are displayed. **, *** and **** correspond to p-value < 0.005, < 0.0005 and < 0.0001 respectively, on a two-way ANOVA followed by a Fisher's LSD test.

Moretto *et al.* Figure S6

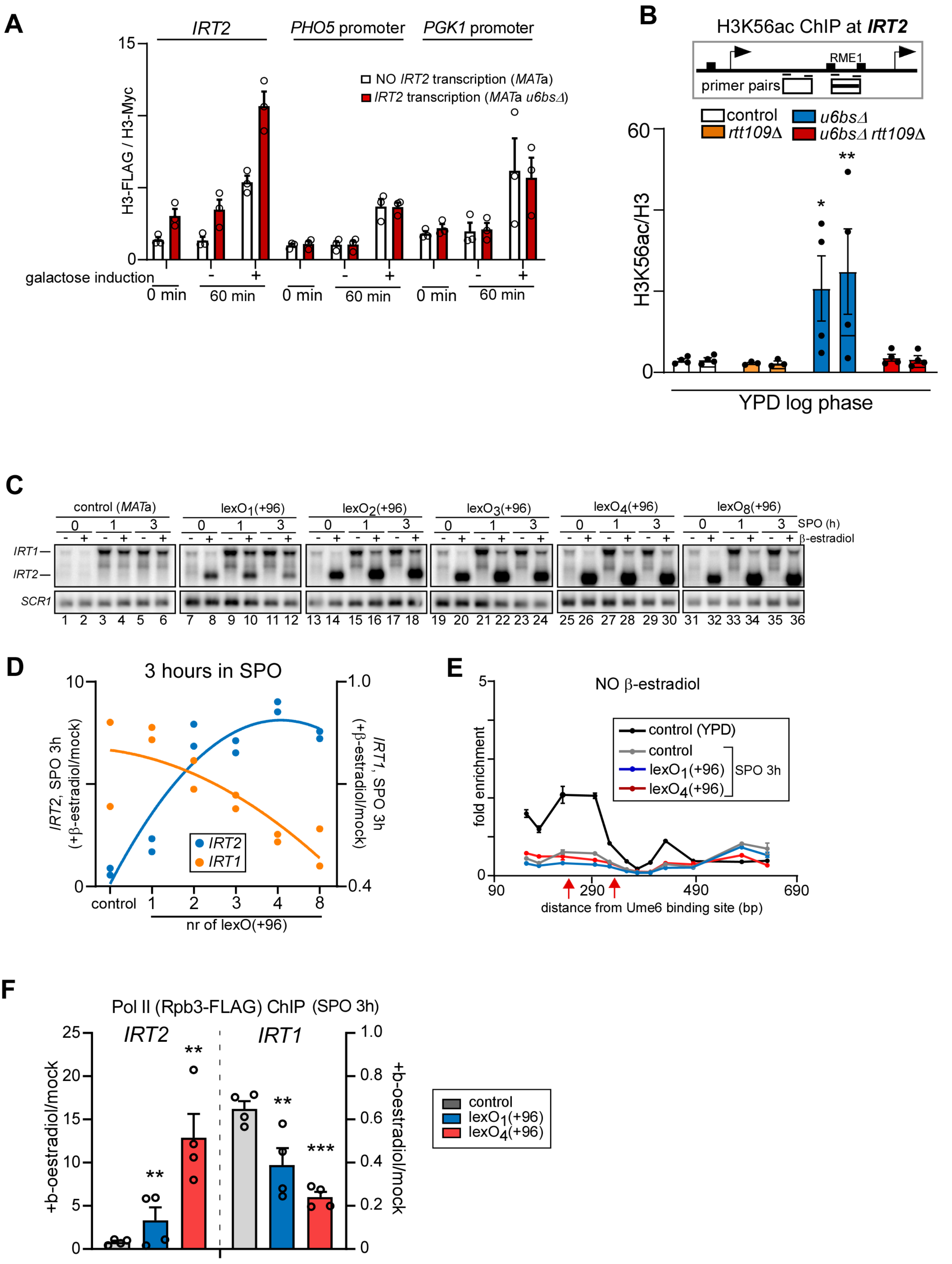

Figure S6: related to Figure 6

***IRT2* transcription has a level dependent effect on local chromatin and transcription states**

A, Histone exchange at the *IRT2, PHO5* and *PGK1* promoters as measured by ChIP. Strains and growth condition as described in Figure 6A and B. To have constitutive levels of *IRT2* transcription we used the *u6bs*Δ mutation (FW7880), while the wild-type cells (FW7853), display no *IRT2* transcription in rich medium (YP). qPCRs primer pairs for the *PHO5* and *PGK1* promoters were used for the analysis. The signal for each histone H3 ChIP (Myc-H3 and FLAG-H3) were normalized to a telomere locus, and ratio for n = 3 +/- SEM are displayed. The start (0 min) and end points (60 min) of galactose induced and non-induced cells are displayed.

B, Histone H3 lysine 56 acetylation (H3K56ac) levels in the *IRT1* promoter as measured by ChIP in control (*MAT*a, FW1509), rtt109 (FW4077), *u6bsΔ* (FW2438) and *u6bsΔ rtt109Δ* (FW5225) cells. Samples were taken during exponential growth (YPD log phase). H3K56ac ChIP signals were normalized to histone H3. n = 4 +/- SEM for all condition except n = 3 for *rtt109*Δ. * and ** correspond to a p-value < 0.05 and < 0.005 respectively on a two-way ANOVA followed by Fisher's LSD test performed on each primer pair individually. qPCRs primer pairs are presented on the top scheme.

C, *IRT1* and *IRT2* expression in control *MAT*a (FW6560), lexO_1(+96) (FW6594), lexO_2(+96) (FW6599), lexO_3(+96) (FW6607), lexO_4(+96) (FW6611), and lexO_8(+96) (FW6619) cells as detected by northern blot. These cells also expressed LexA-ER. Cells were grown in rich medium (YPD) to saturation (24 h), shifted and grown in pre-SPO for 15 h, and cells either mock treated or treated with β-estradiol (25 nM) for an additional 1 hour. Subsequently cells were transferred to SPO and the treatments were continued. Samples were taken at the indicated time points. *SCR1* was used as loading control.

D, Quantification of *IRT1* expression as described in C. Signals were normalized to *SCR1* and presented as a ratio of β-estradiol over mock treatment for the 3 h time points.

E, Chromatin structure at the *IRT1* promoter in the presence of distinct levels of *IRT2* transcription. *MAT*a control cells (FW6560) or cells harbouring 1 or 4 lexO(+96) sites (FW6594 or FW6611) were grown as described in C and the mock treated condition is displayed. β-estradiol treated condition is displayed in figure 6F. Cells grown in rich nutrient conditions (YPD) till exponential growth were included in the analysis. Chromatin was digested with micrococcal nuclease (MNase) followed by qPCR using scanning primer pairs in *IRT2*. The red arrows indicate the position of the Rme1 binding sites. The signals were normalized over a telomeric region from chromosome VI. n = 3 +/- SEM.

F, Pol II binding at *IRT1* as measured by ChIP. Similar to E, except that cells also contained Rpb3-FLAG, which was used for the immunoprecipitation step (FW8808, FW8812 and FW8827). Cells were grown as described in C, and samples for ChIP were taken at 3 h in SPO. Signals were normalized over the silent mating type locus *HMR*. The ratio β-estradiol treated over mock treated are presented. n = 4 +/- SEM. ** and *** correspond to p-value < 0.005 and < 0.0005 respectively on a one-way ANOVA followed by a Fisher's LSD test.

Moretto *et al.* Figure S7

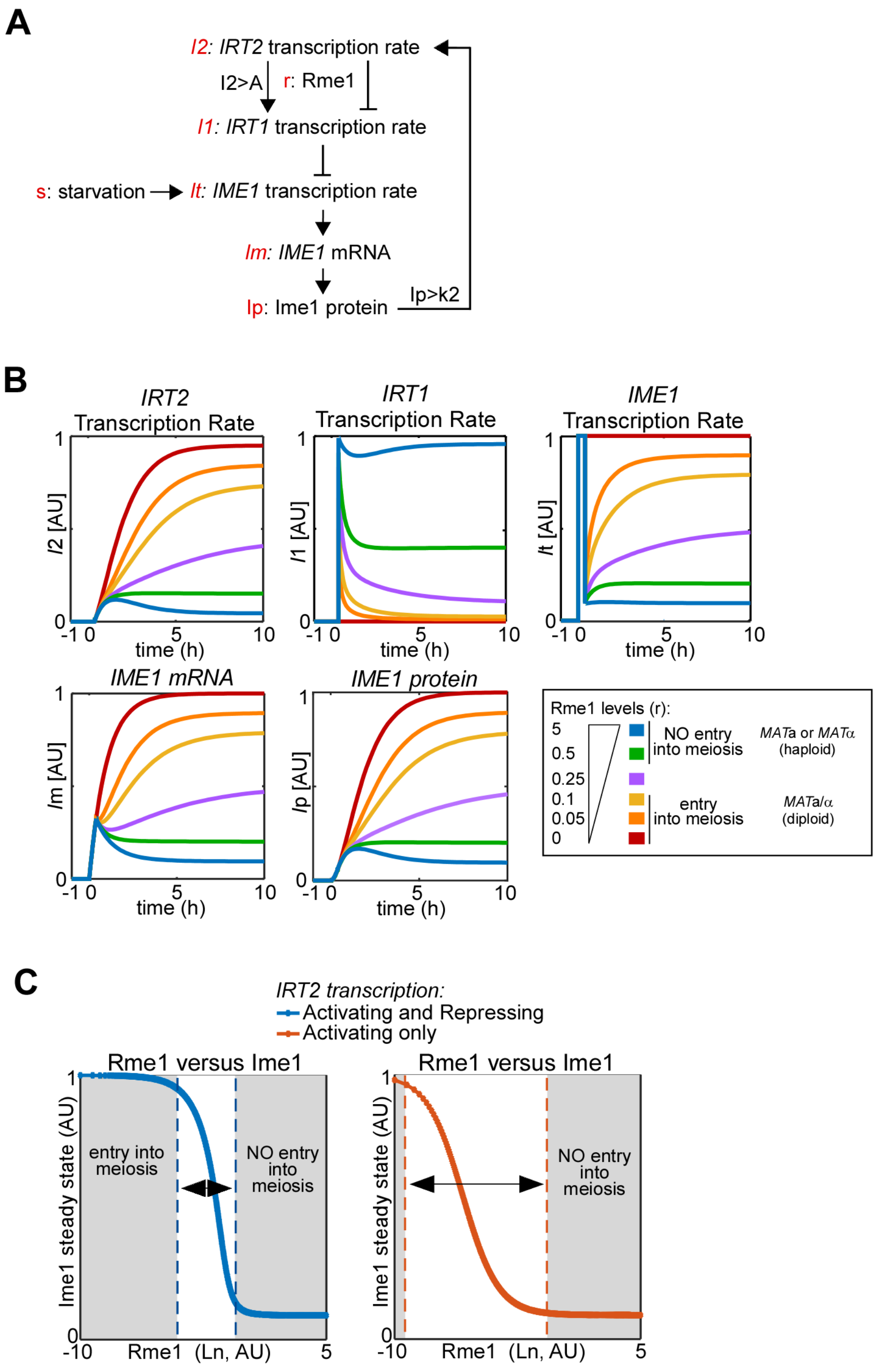

Figure S7: related to Figure 7

Modelling of cell-type specific control of Ime1 expression involving noncoding transcription

A, Model for Ime1 regulation by *IRT1*, *IRT2*, and *IME1* (see Methods section for details).

B, Simulation of *IRT1*, *IRT2*, and *IME1* transcription rates, *IME1* mRNA and Ime1 protein levels in the presence of different concentrations of Rme1. Different Rme1 concentrations are indicated (r= 0, 0.05, 0.1, 0.25, 0.5 and 5, arbitrary units), leading to either no entry into meiosis in *MAT*a or *MAT*α (haploid), or entry in meiosis in *MAT*a/α (diploid) cells.

C, Dose response relationship between Ime1 steady state levels and Rme1 concentration, simulated for WT *IRT2* (activating and repressing levels) and *IRT2* activating levels only, which are simulated by setting I2=A. Indicated are the levels of Rme1 corresponding to either no entry into meiosis in haploid cells, or entry in meiosis into diploid cells.

Table S1. Screen for mutants that affect *IRT1* expression. Related to Figure 4.

gene delete	*IRT1*	*IME1*	*RME1*
RME1	---	+++	none
ASF1	-	+	NT
CAC2	0	NT	NT
CTK1	---	+	NT
ELP4	0	0	NT
ELP6	0	0	NT
GCN5	---	+++	---
HIR1	0	NT	NT
HST3	0	0	NT
HST4	0	0	NT
JHD2	0	0	NT
NAP1	0	0	NT
RLF2	+	NT	NT
RPB9	--	++	NT
RTR1	0	0	NT
RTT106	0	NT	NT
RTT109	--	++	0
SET1	0	0	NT
SET3	0	+	NT
SNF5	---	NT	---
SUB1	0	0	NT
SWC3	0	0	NT
SWC5	0	0	NT
SWR1	0	NT	0
VPS75	0	0	NT
HOS2	0	0	NT

NT: not tested
+/-: difference with WT
0: no difference with WT

Table S3: Oligo nucleotide sequences used. Related to STAR methods.

FW463	caacgcctccgataatgtatatg	IME1 northern probe
FW464	acgtcgaaggcaatttctaatg	IME1 northern probe
FW481	atttttagcgactgccgaaa	IRT2 qPCR (Rme1 ChIP)
FW482	atgcaacgcctacttgtttt	IRT2 qPCR (Rme1 ChIP)
FW1895	aaaatgaaaggcagaagatg	IRT2 qPCR (H3 ChIP oligo pair 1)
FW1896	ctggtatggtattgtaagga	IRT2 qPCR (H3 ChIP oligo pair 1)
FW1899	atcatgctgttctttccgcc	IRT2 qPCR (H3 ChIP oligo pair 2)
FW1900	cccacccttcttttattgag	IRT2 qPCR (H3 ChIP oligo pair 2)
FW1905	tcaagaagtccactaaatgg	IRT2 qPCR (H3 ChIP oligo pair 3)
FW1906	acaattttatgcttttgagg	IRT2 qPCR (H3 ChIP oligo pair 3)
FW43	acgatccccgtccaagttatg	HMR qPCR (Rme1 and Pol II ChIP)
FW50	cttcaaaggagtcttaatttccctg	HMR qPCR (Rme1 and Pol II ChIP)
FW490	tgatatgtatgggttaaaaaggatg	IRT1-IRT2 dual northern probe
FW540	ggcagttcaaaggcttttctta	IRT1-IRT2 dual northern probe, ChIP and RT-qPCR, IRT1
FW489	atgcaacgcctacttgtttt	IRT2 northern probe
FW493	gatggagggttggcataaaa	IRT2 northern probe
FW1555	gctgcagaacttggttcataca	IME1 northern probe
FW464	acgtcgaaggcaatttctaatg	IME1 northern probe
FW1841	gaagtgtcccggctataataaa	SCR1 northern probe
FW1842	gacgctggataaaactcccc	SCR1 northern probe
FW2448	tccgaacgctattccagaaagt	ChIP/MNase normalisation (Telomere locus)
FW2449	ccataatgcctcctatatttagcctttt	ChIP/MNase normalisation (Telomere locus)
FW2450	catatattatctatatcatgc	MNase qPCR
FW2451	ccgtgagggaatacattaat	MNase qPCR
FW1899	atcatgctgttctttccgcc	MNase qPCR
FW1900	cccacccttcttttattgag	MNase qPCR
FW1901	attaatgtattccctcacgg	MNase qPCR
FW1902	cttcttgaggttcttttgacatc	MNase qPCR
FW1087	ggatgtcaaaagaacctcaaga	MNase qPCR
FW1088	tttcggcagtcgctaaaaat	MNase qPCR
FW1905	tcaagaagtccactaaatgg	MNase qPCR
FW1906	acaattttatgcttttgagg	MNase qPCR
FW1907	gccgaaaacgtacggctaac	MNase qPCR
FW488	atttttagcgactgccgaaa	MNase qPCR
FW487	tttttgttatctgcctgaaacg	MNase qPCR
FW1908	gctcactttttttcctaccca	MNase qPCR

FW2452	gctgtacctcaaaagcataa	MNase qPCR
FW2453	tccagaaacggtttcttatat	MNase qPCR
FW1909	aaaacaagtaggcgttgcat	MNase qPCR
FW1910	accctatttcttcacgaggg	MNase qPCR
FW1030	gagcgccaacactatataag	MNase qPCR
FW1031	caaattctttaaactaagcgc	MNase qPCR
FW1913	gcgcttagtttaaagaatttg	MNase qPCR
FW1914	cctttgttttctctttatcccc	MNase qPCR
FW1915	cttgattattggcattccgc	MNase qPCR
FW1916	ttgctcggaggtactagtca	MNase qPCR
FW539	gggtcttaaatacgcagggaat	ChIP and RT- qPCR, IRT1
FW106	gtaccaccatgttcccaggtatt	RT- qPCR, ACT1 control
FW107	caagatagaaccaccaatccaga	RT- qPCR, ACT1 control

Table S4: Plasmids. Related to STAR methods.

pFW7	pRS306-MATa-URA3	(van Werven et al., 2012)
pFW244	pRS304 MATa-TRP1	This study
pFW355	pRS304-GPDpr-CRE-EBD78-CYC1t	Gift Celine Bouchoux
pFW211	pUG6-Myc-C-Avitag	(van Werven and Timmers, 2006)
pFW502	pDM9-HHT2-HHF2-URA3	(Sommermeyer et al., 2013)
pFW503	pDM9-H3K56A-HFF1-URA3	This study
pFW504	pDM9-H3K56R-HFF1-URA3	This study
pFW643	pUB921_p1LexOCYC1-3V5	Gift Elçin Ünal (Chia et al., 2017)
pFW644	pUB922_p2LexOCYC1-3V5	Gift Elçin Ünal (Chia et al., 2017)
pFW645	pUB923_p3LexOCYC1-3V5	Gift Elçin Ünal (Chia et al., 2017)
pFW646	pUB924_p4LexOCYC1-3V5	Gift Elçin Ünal
pFW647	pUB925_p8LexOCYC1-3V5	Gift Elçin Ünal (Chia et al., 2017)

Data S1. Compilation of uncropped northern blot scans used for the figures throughout the manuscript. Related to Figures 1, 2, 4, 5, S1, S2, S4, S5, and S6.

Figure 1B

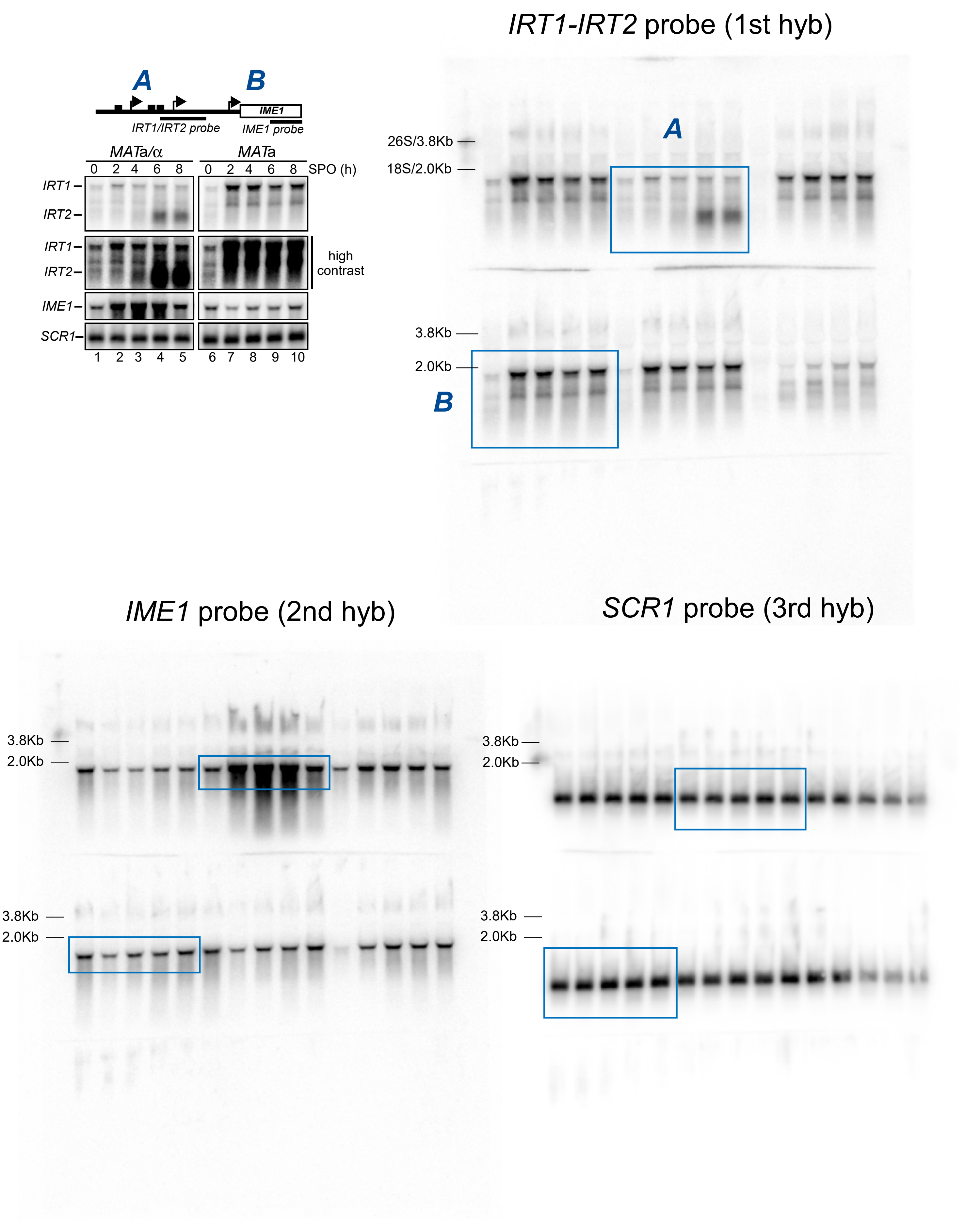
IRT1-IRT2 probe (1st hyb)
IME1 probe (2nd hyb)
SCR1 probe (3rd hyb)
A
B
IME1
IRT1/IRT2 probe
IME1 probe
MATa/α
MATa
0 2 4 6 8
0 2 4 6 8
SPO (h)
IRT1
IRT2
high contrast
IME1
SCR1
1 2 3 4 5 6 7 8 9 10
26S/3.8Kb
18S/2.0Kb
3.8Kb
2.0Kb

Figure 1D

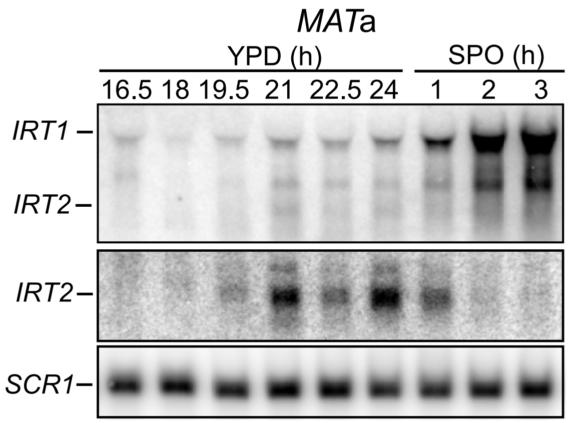
MATa
YPD (h)
SPO (h)
16.5 18 19.5 21 22.5 24 1 2 3
IRT1
IRT2
IRT2
SCR1

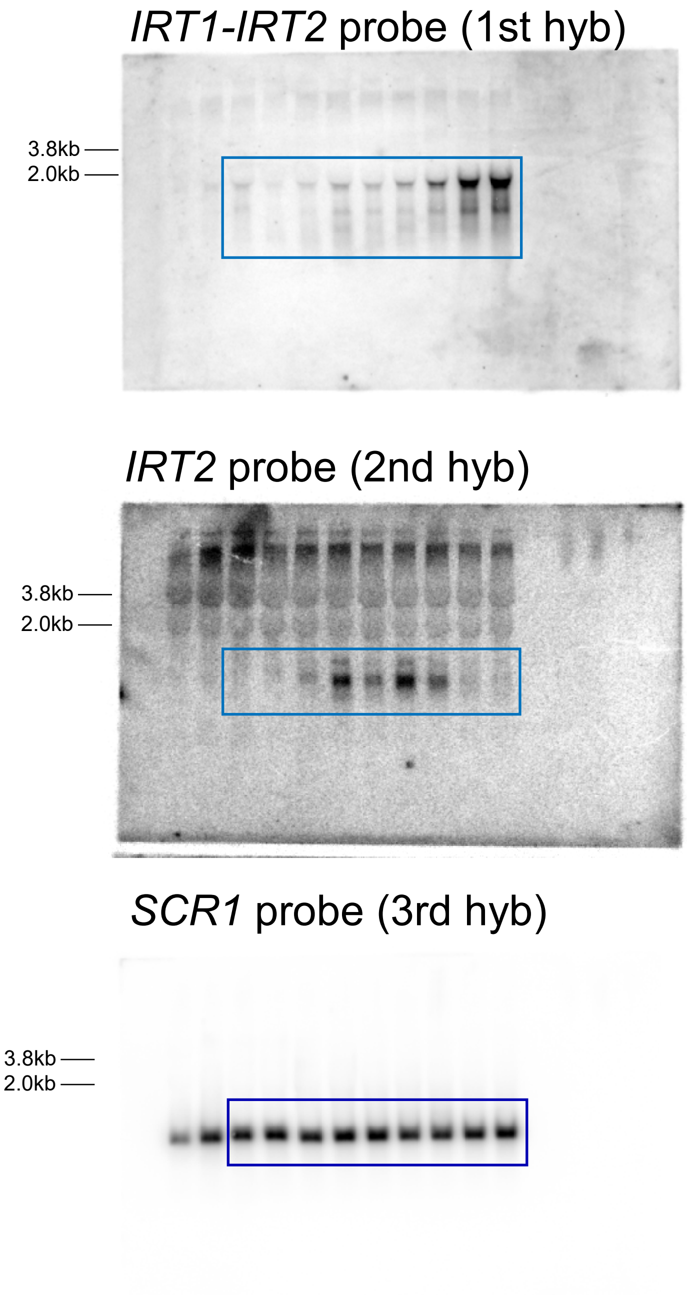
IRT1-IRT2 probe (1st hyb)
3.8kb
2.0kb
IRT2 probe (2nd hyb)
3.8kb
2.0kb
SCR1 probe (3rd hyb)
3.8kb
2.0kb

Figure 1E and Figure S1E

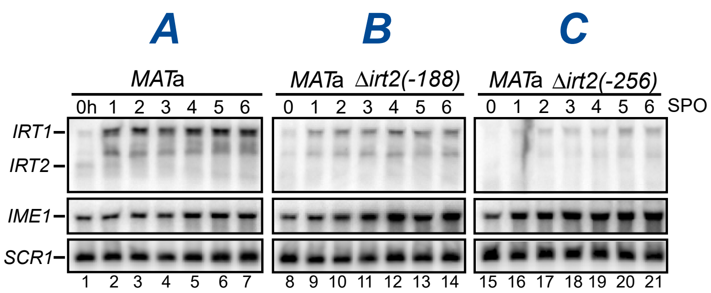

IRT1-IRT2 probe (1st hyb)

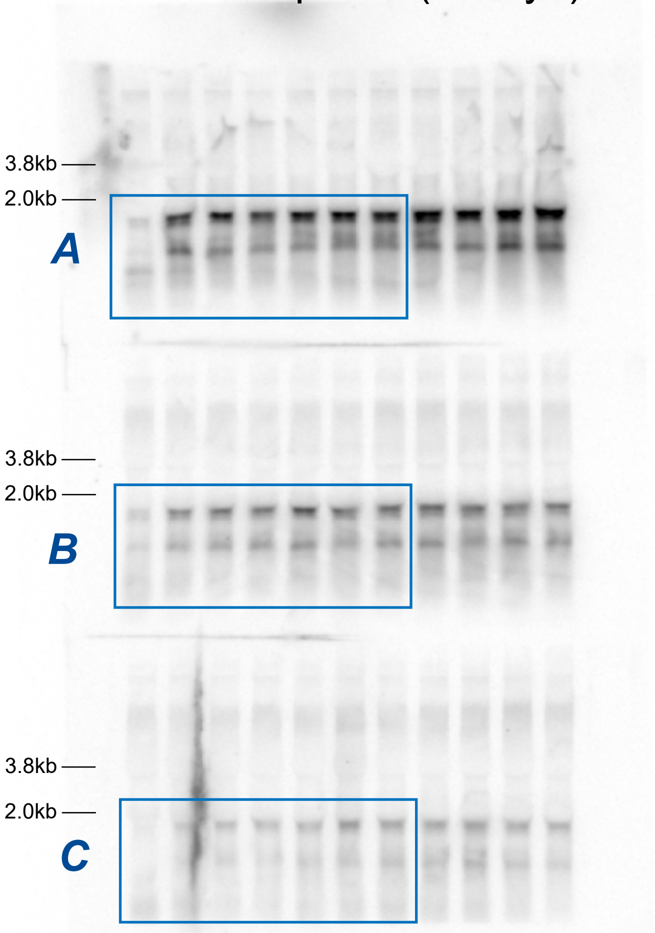

SCR1 probe (3rd hyb)

IME1 probe (2nd hyb)

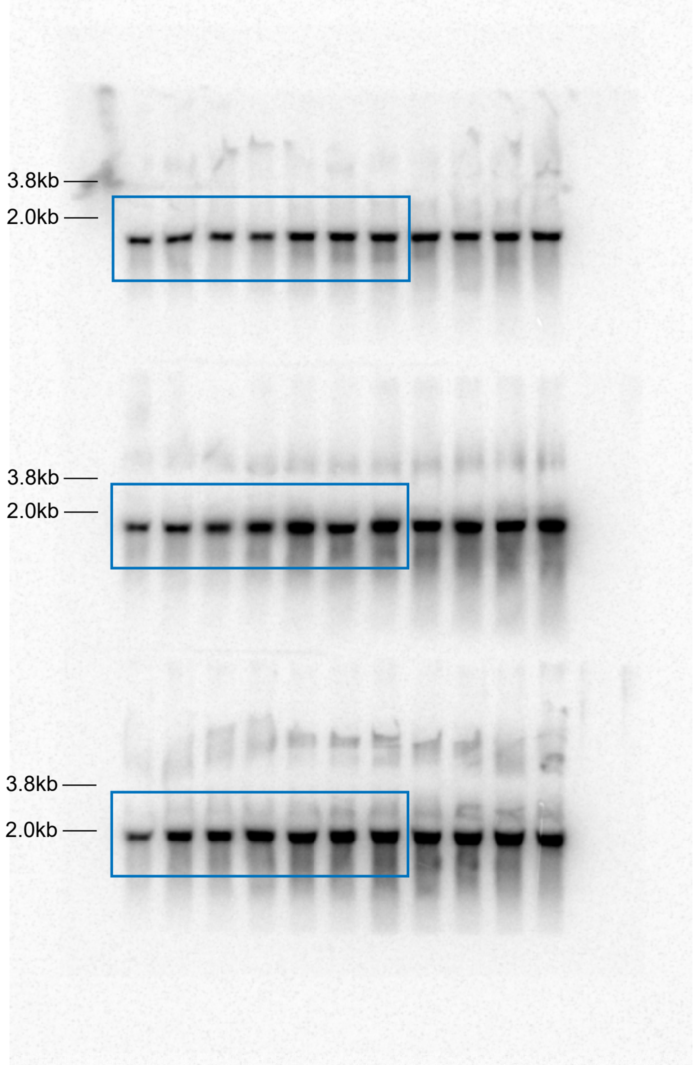

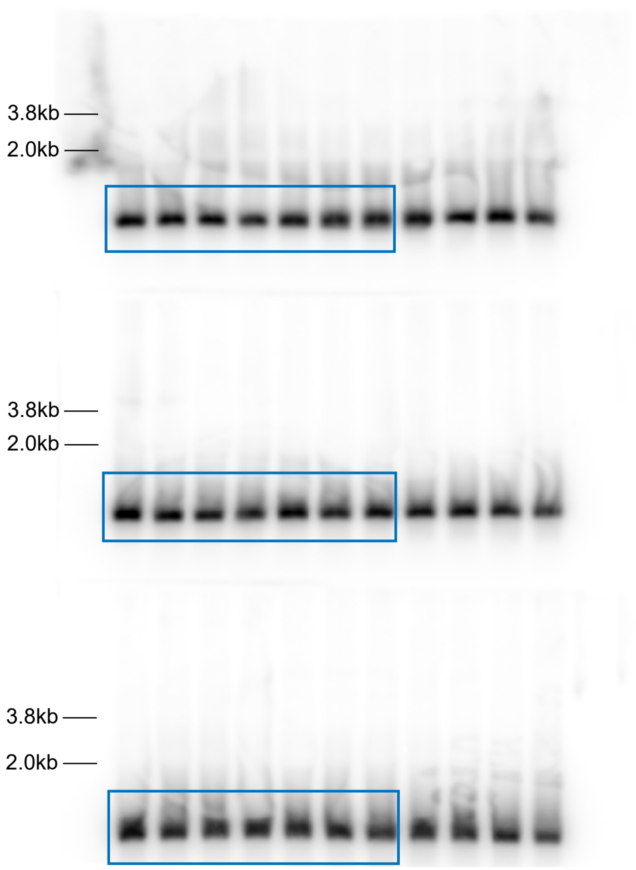

Figure 2B

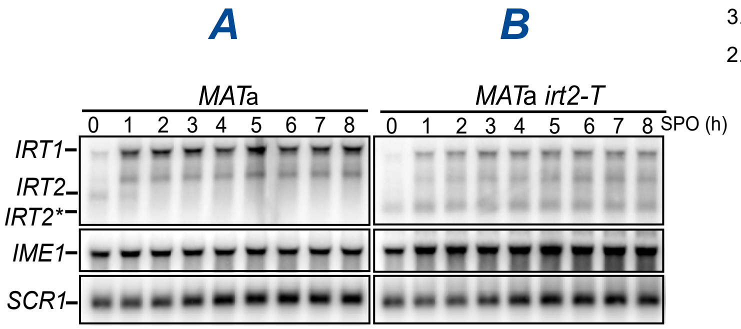

IRT1-IRT2 probe (1st hyb)

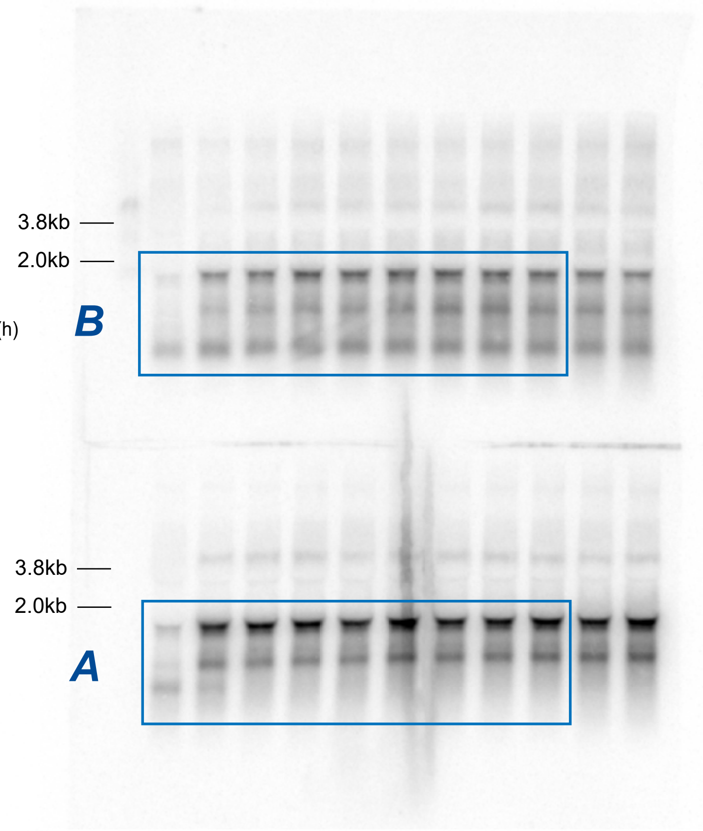

IME1 probe (2nd hyb)

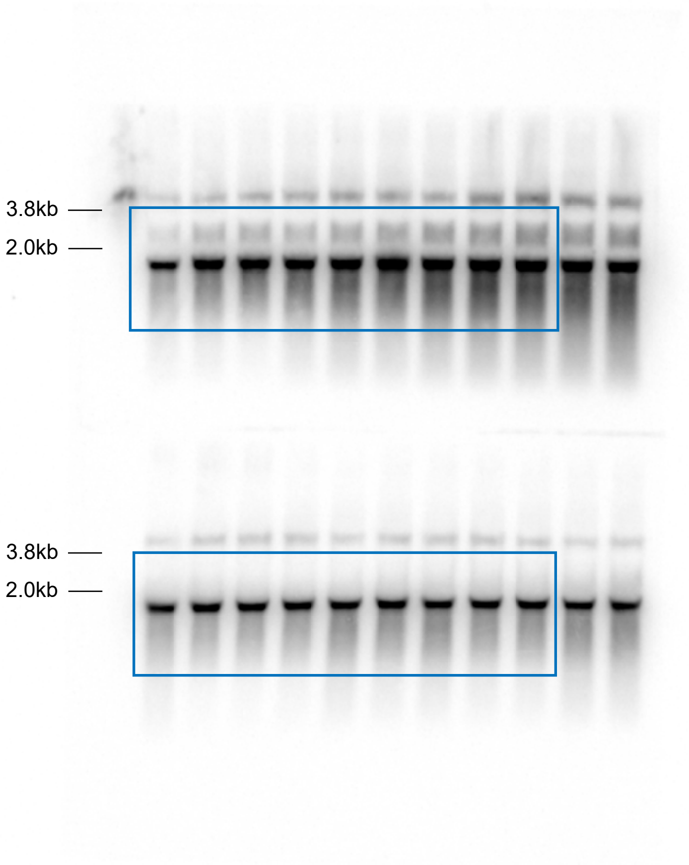

SCR1 probe (3rd hyb)

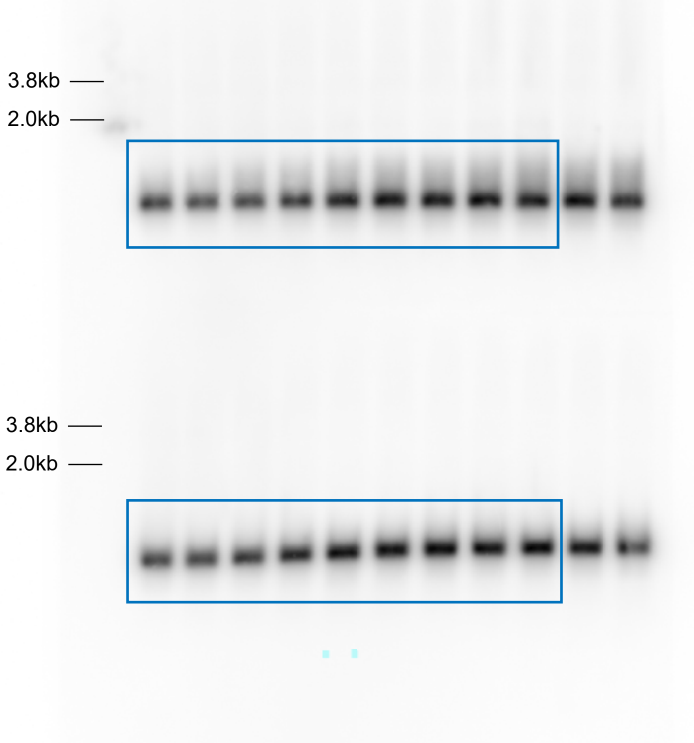

Figure 2F

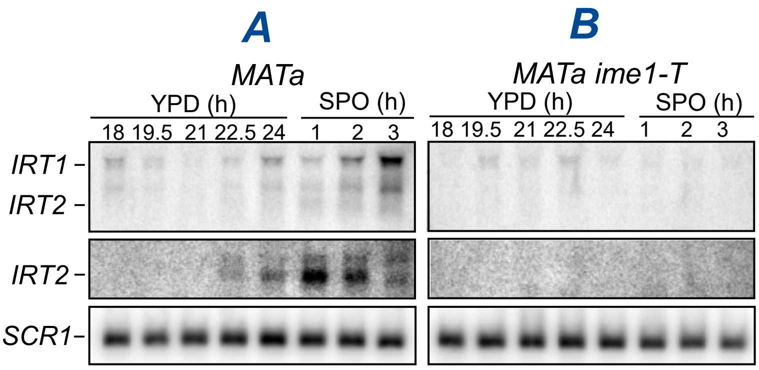
A
MATa
YPD (h)
SPO (h)
18 19.5 21 22.5 24 1 2 3
B
MATa ime1-T
YPD (h)
SPO (h)
18 19.5 21 22.5 24 1 2 3
IRT1
IRT2
IRT2
SCR1

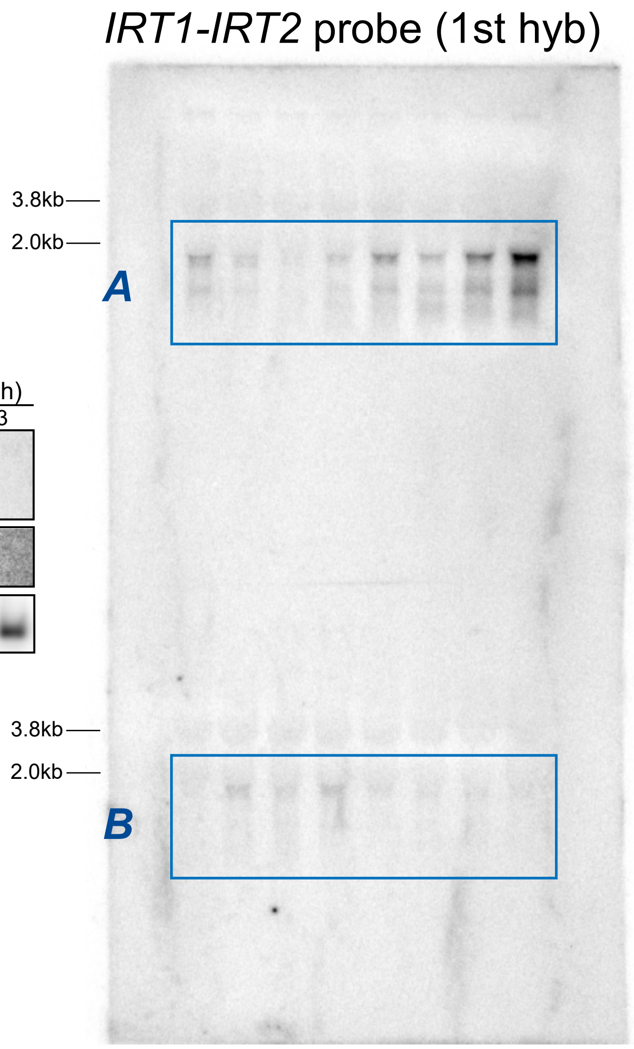
IRT1-IRT2 probe (1st hyb)
3.8kb
2.0kb
A
3.8kb
2.0kb
B

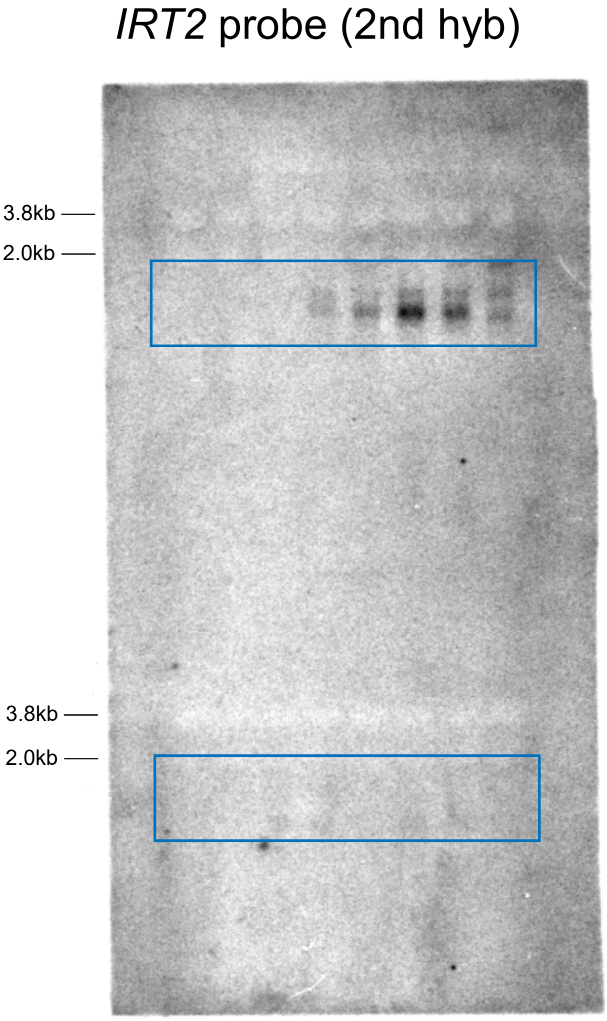
IRT2 probe (2nd hyb)
3.8kb
2.0kb
3.8kb
2.0kb

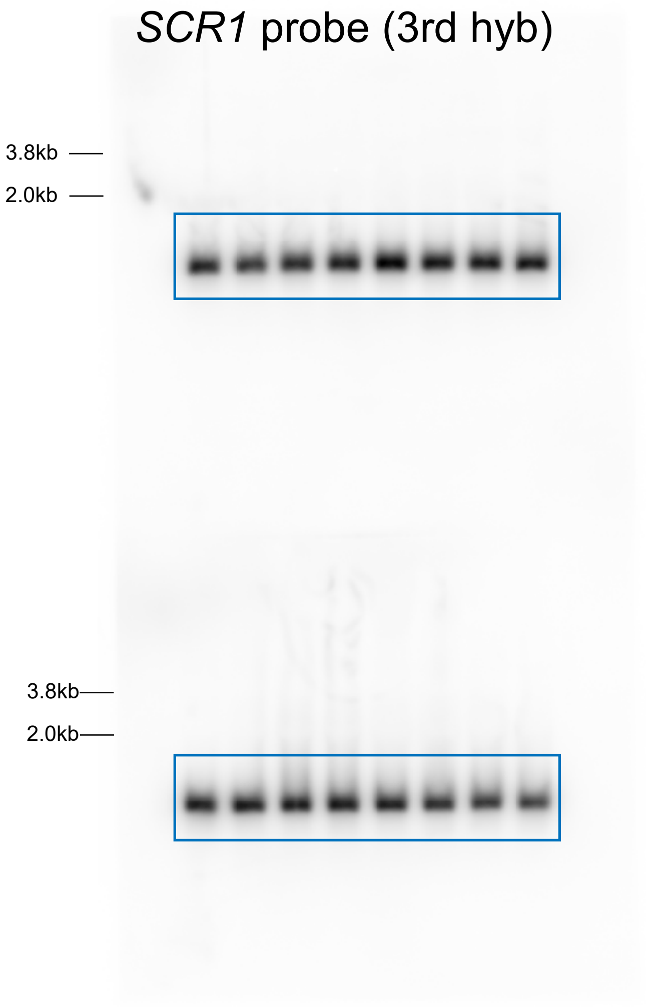
SCR1 probe (3rd hyb)
3.8kb
2.0kb
3.8kb
2.0kb

Figure 2H

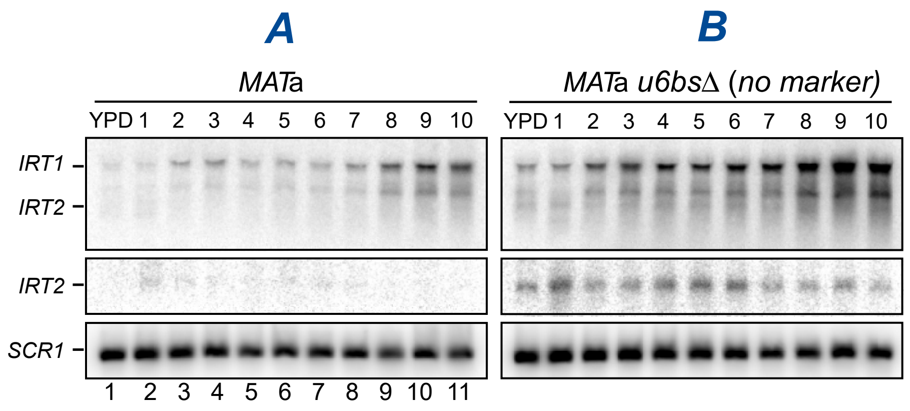
A
B
MATa
MATa u6bsΔ (no marker)
YPD 1 2 3 4 5 6 7 8 9 10
YPD 1 2 3 4 5 6 7 8 9 10
IRT1
IRT2
IRT2
SCR1
1 2 3 4 5 6 7 8 9 10 11

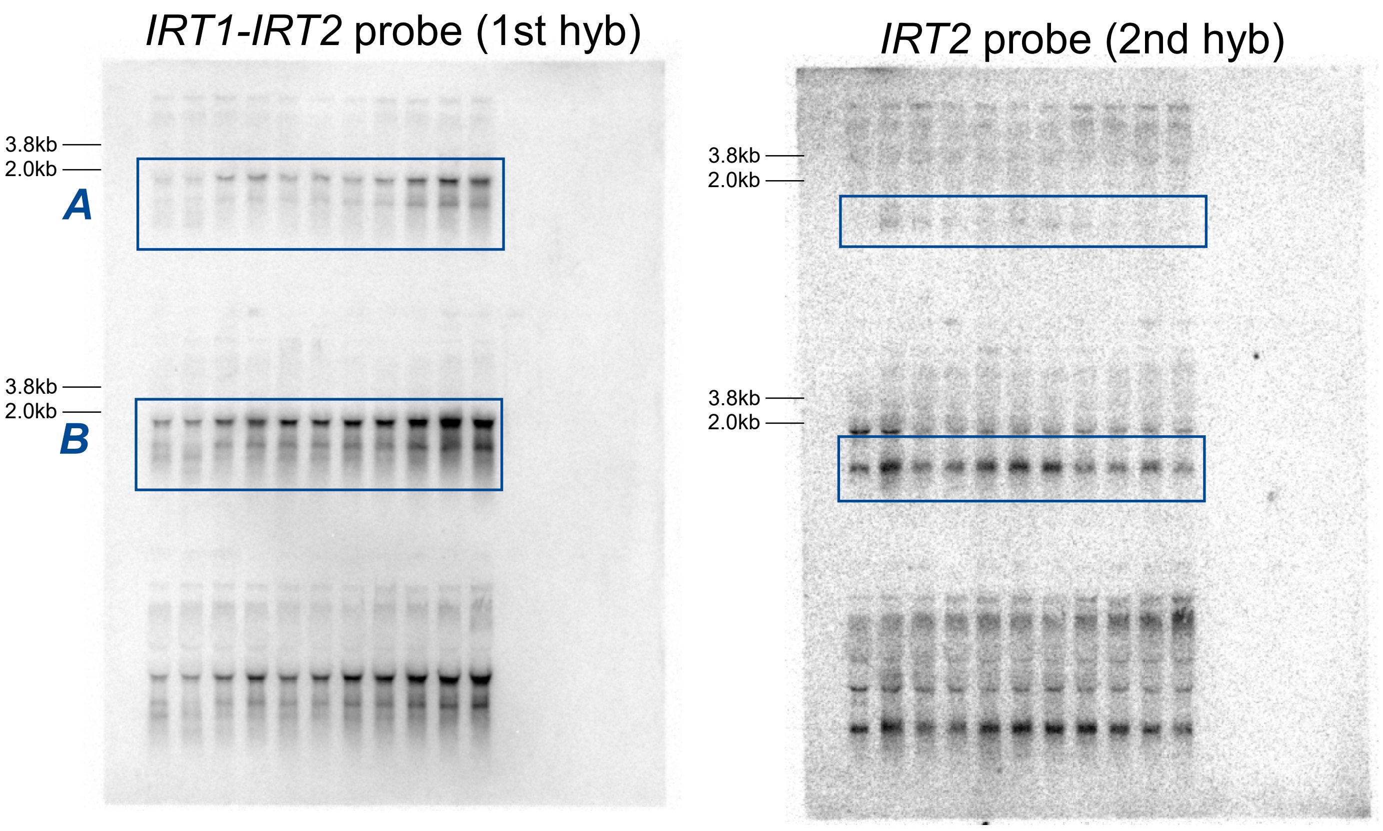
IRT1-IRT2 probe (1st hyb)
IRT2 probe (2nd hyb)
3.8kb
2.0kb
A
3.8kb
2.0kb
B
3.8kb
2.0kb
3.8kb
2.0kb

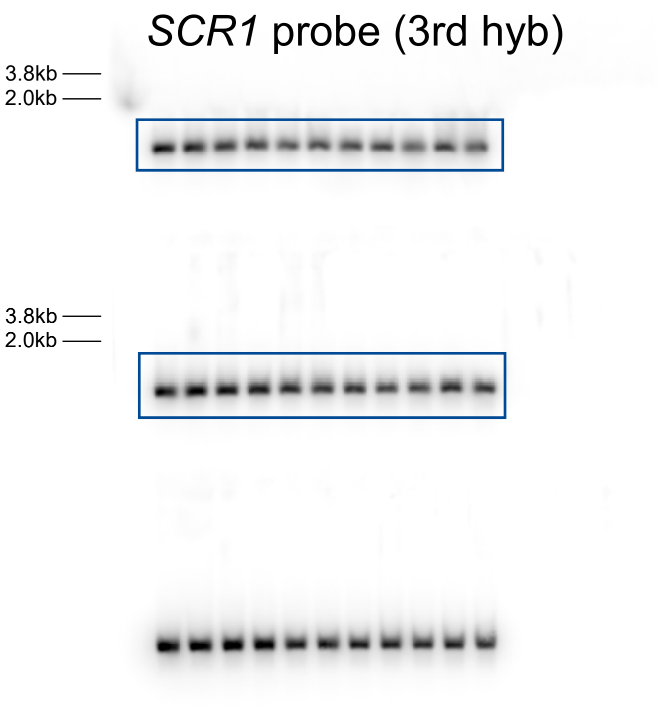
SCR1 probe (3rd hyb)
3.8kb
2.0kb
3.8kb
2.0kb

Figure 2I

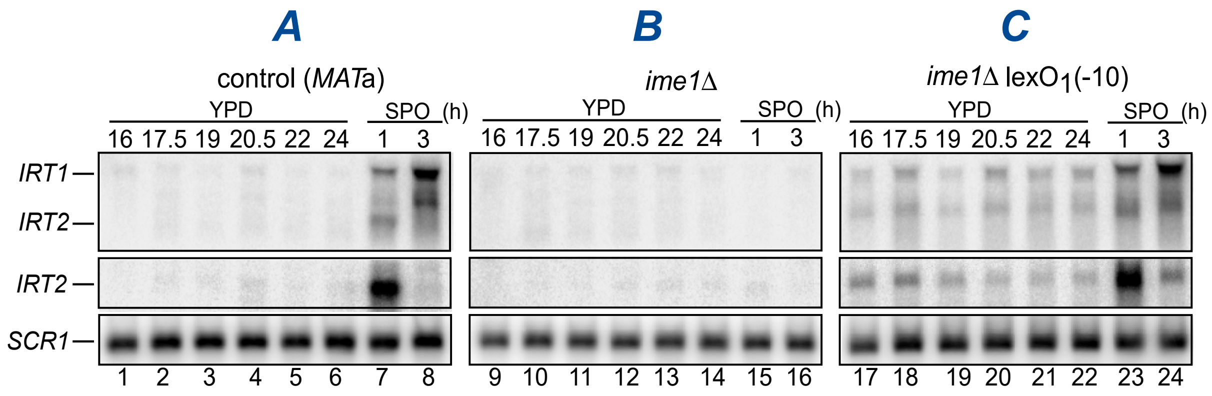
A
B
C
control (MATa)
ime1Δ
ime1Δ lexO1(-10)
YPD
SPO (h)
16 17.5 19 20.5 22 24 1 3
IRT1
IRT2
IRT2
SCR1
1 2 3 4 5 6 7 8
9 10 11 12 13 14 15 16
17 18 19 20 21 22 23 24

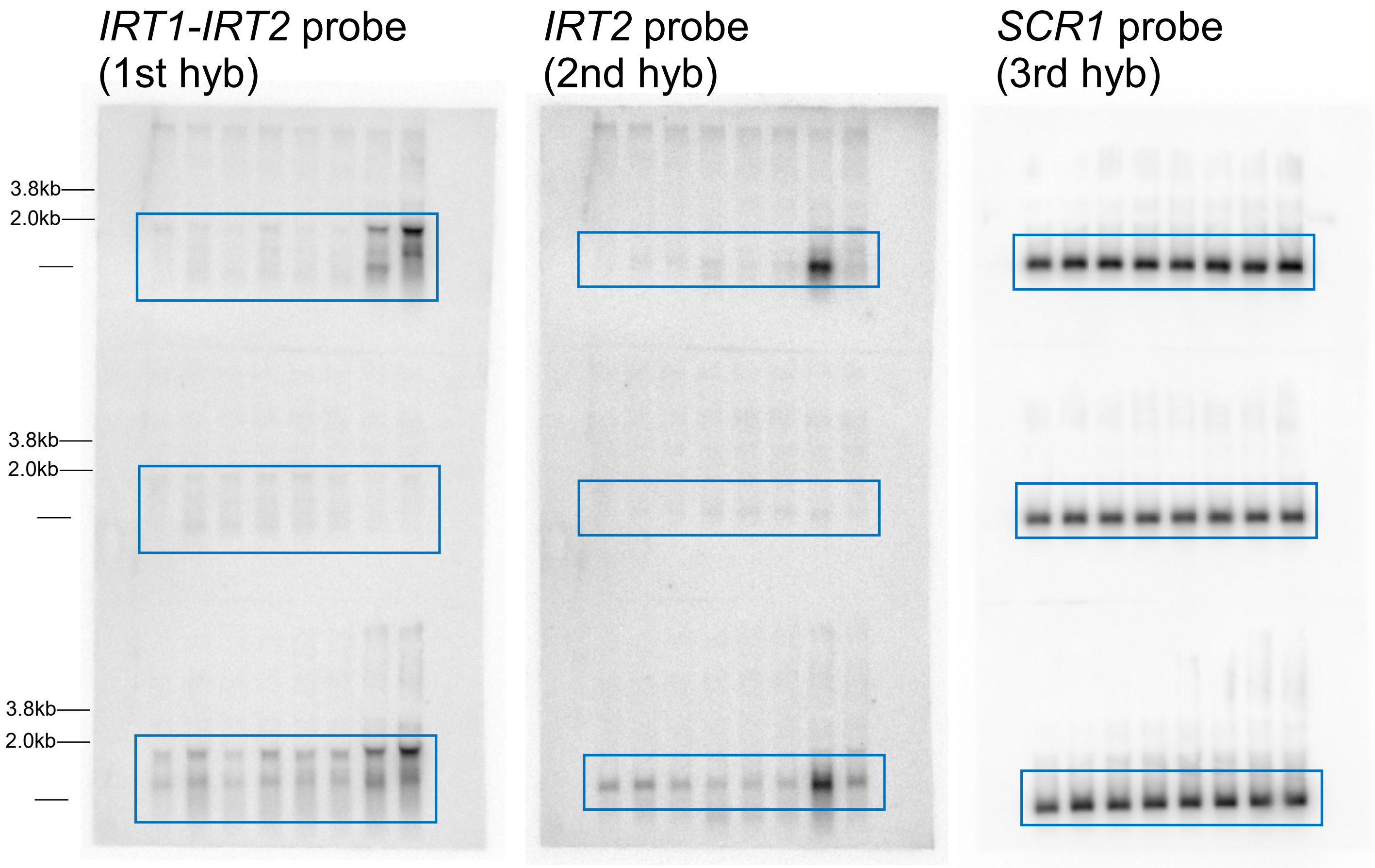
IRT1-IRT2 probe (1st hyb)
IRT2 probe (2nd hyb)
SCR1 probe (3rd hyb)
3.8kb
2.0kb

Figure 4B (panels A and B) and Figure S4A (panels A and C)

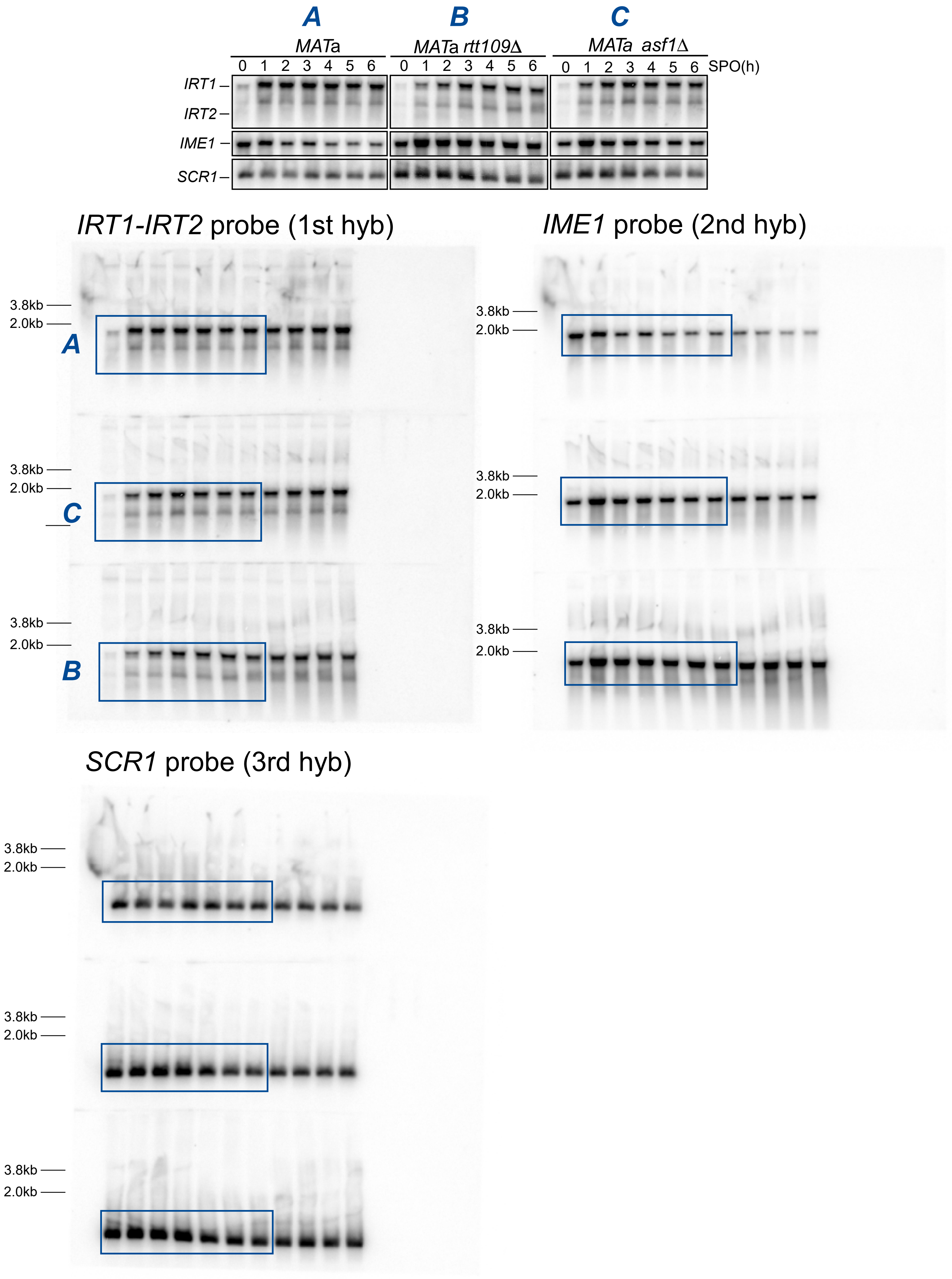

Figure 4F

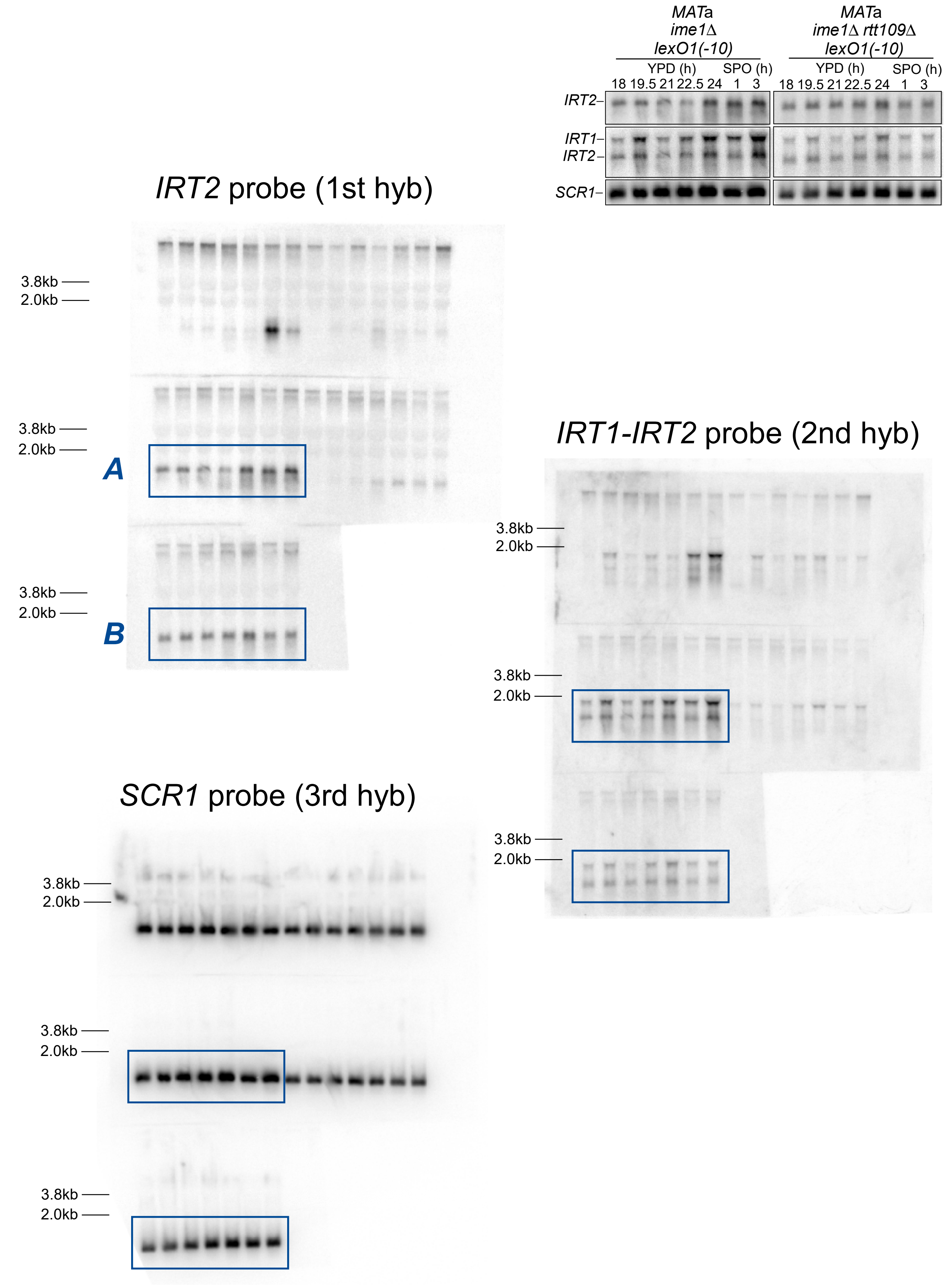

MATa
ime1Δ
lexO1(-10)
MATa
ime1Δ rtt109Δ
lexO1(-10)
YPD (h)
SPO (h)
18 19.5 21 22.5 24 1 3
18 19.5 21 22.5 24 1 3
IRT2
IRT1
IRT2
SCR1
IRT2 probe (1st hyb)
3.8kb
2.0kb
A
B
IRT1-IRT2 probe (2nd hyb)
SCR1 probe (3rd hyb)

Figure 4G

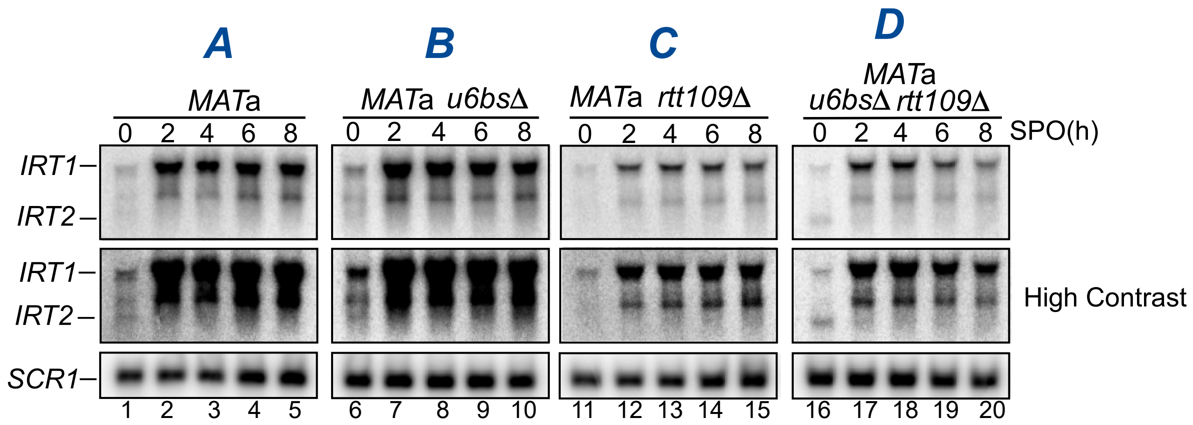
A
B
C
D
MATa
MATa u6bsΔ
MATa rtt109Δ
MATa u6bsΔ rtt109Δ
0 2 4 6 8
SPO(h)
IRT1
IRT2
High Contrast
SCR1
1 2 3 4 5 6 7 8 9 10 11 12 13 14 15 16 17 18 19 20

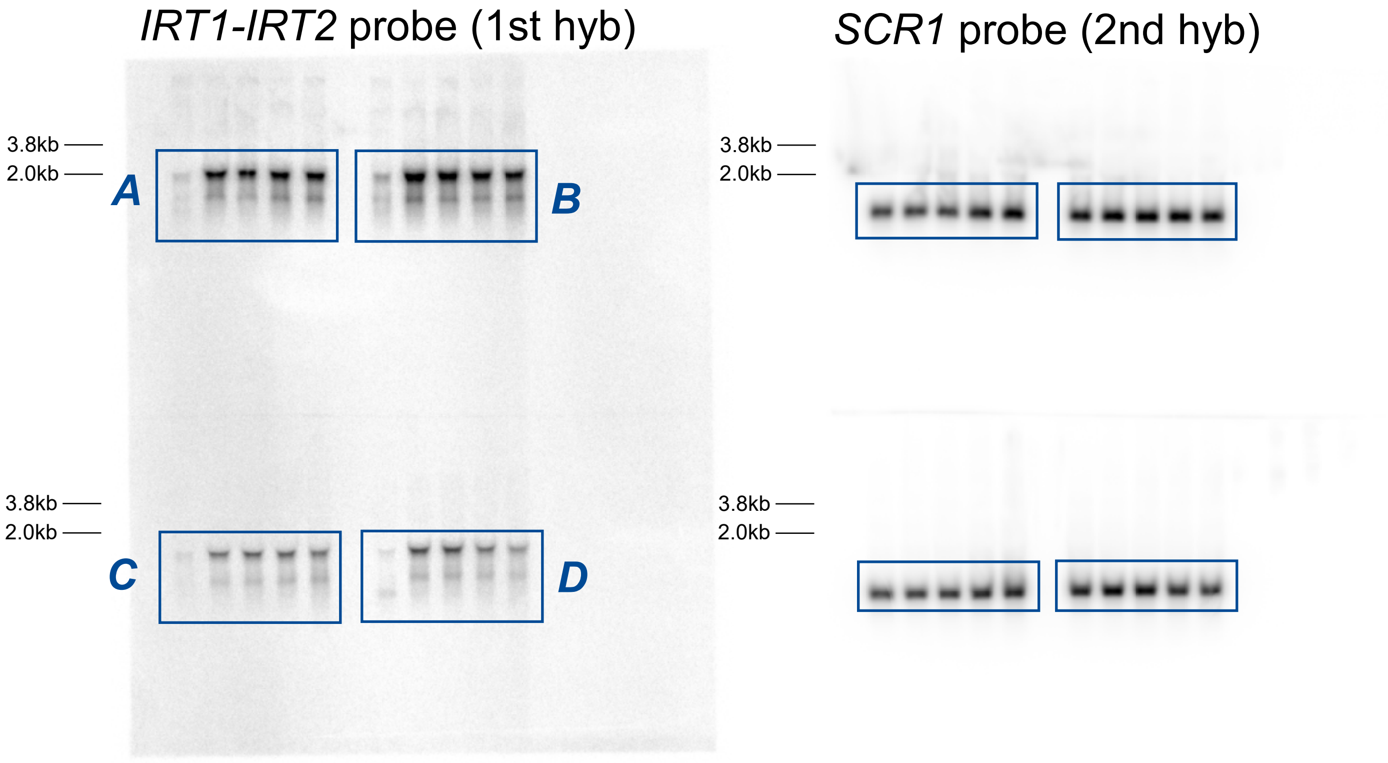
IRT1-IRT2 probe (1st hyb)
SCR1 probe (2nd hyb)
3.8kb
2.0kb
A
B
C
D

Figure 5A

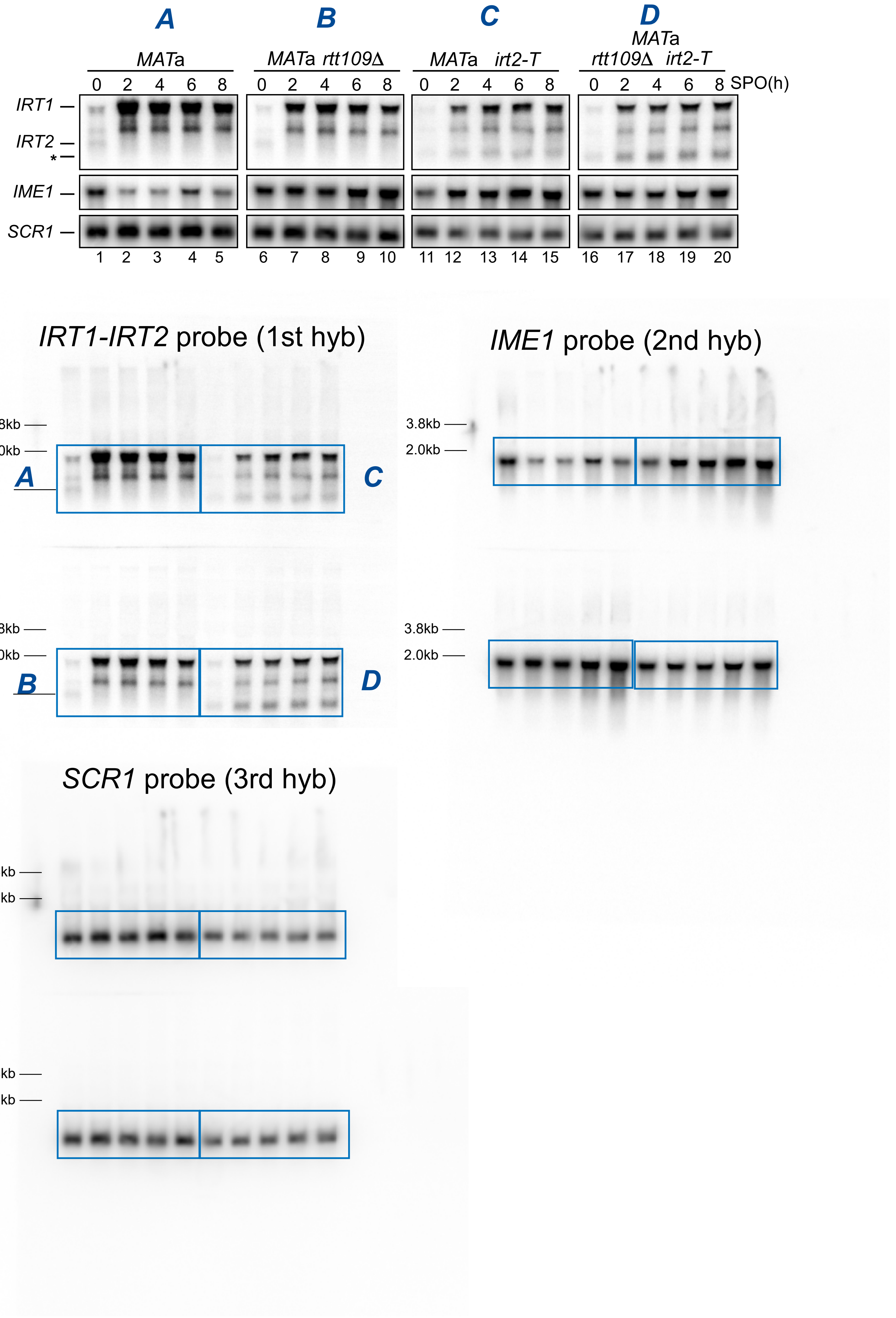
A
B
C
D
MATa
MATa rtt109Δ
MATa irt2-T
MATa rtt109Δ irt2-T
0 2 4 6 8
SPO(h)
IRT1
IRT2
*
IME1
SCR1
1 2 3 4 5 6 7 8 9 10 11 12 13 14 15 16 17 18 19 20
IRT1-IRT2 probe (1st hyb)
IME1 probe (2nd hyb)
SCR1 probe (3rd hyb)
3.8kb
2.0kb

Figure 5D

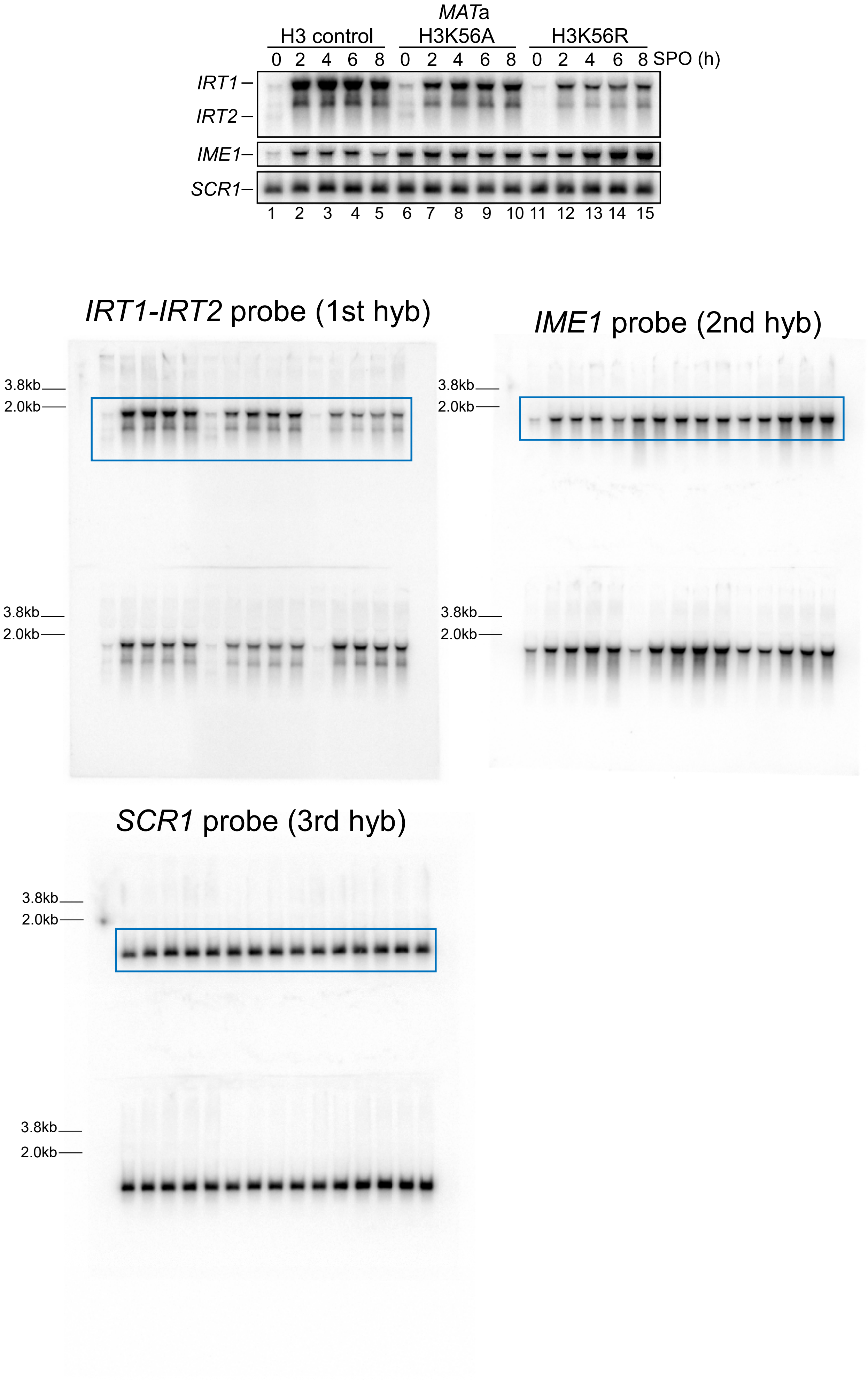
MATa
H3 control
H3K56A
H3K56R
0 2 4 6 8 0 2 4 6 8 0 2 4 6 8 SPO (h)
IRT1
IRT2
IME1
SCR1
1 2 3 4 5 6 7 8 9 10 11 12 13 14 15
IRT1-IRT2 probe (1st hyb)
IME1 probe (2nd hyb)
SCR1 probe (3rd hyb)
3.8kb
2.0kb

Figure S1C

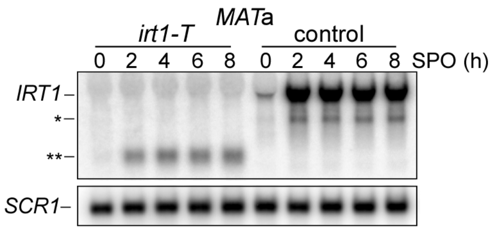
MATa
irt1-T
control
0 2 4 6 8 0 2 4 6 8 SPO (h)
IRT1
*
**
SCR1

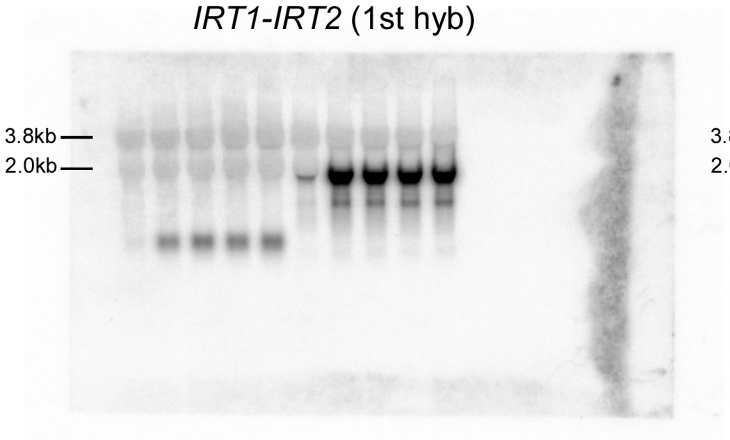
IRT1-IRT2 (1st hyb)
3.8kb
2.0kb

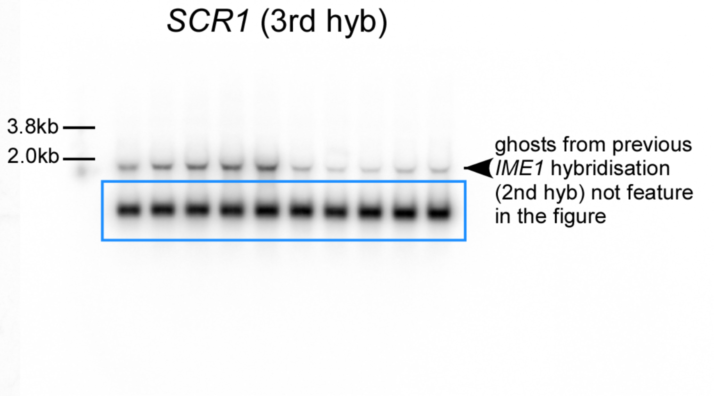
SCR1 (3rd hyb)
3.8kb
2.0kb
ghosts from previous
IME1 hybridisation
(2nd hyb) not feature
in the figure

Figure S2B

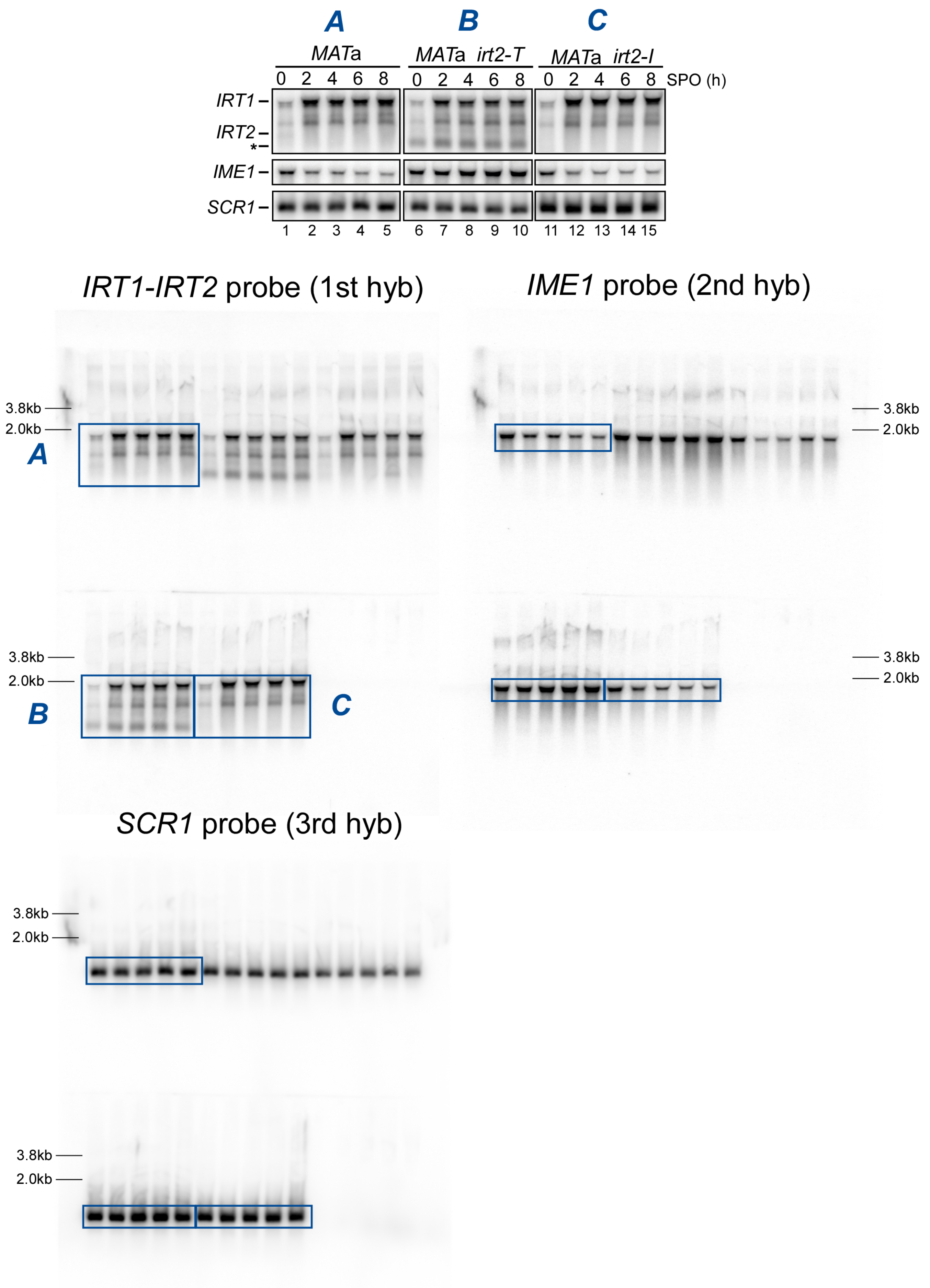
A
B
C
MATa
MATa irt2-T
MATa irt2-I
0 2 4 6 8
SPO (h)
IRT1
IRT2
*
IME1
SCR1
1 2 3 4 5 6 7 8 9 10 11 12 13 14 15
IRT1-IRT2 probe (1st hyb)
IME1 probe (2nd hyb)
SCR1 probe (3rd hyb)
3.8kb
2.0kb

Figure S2D

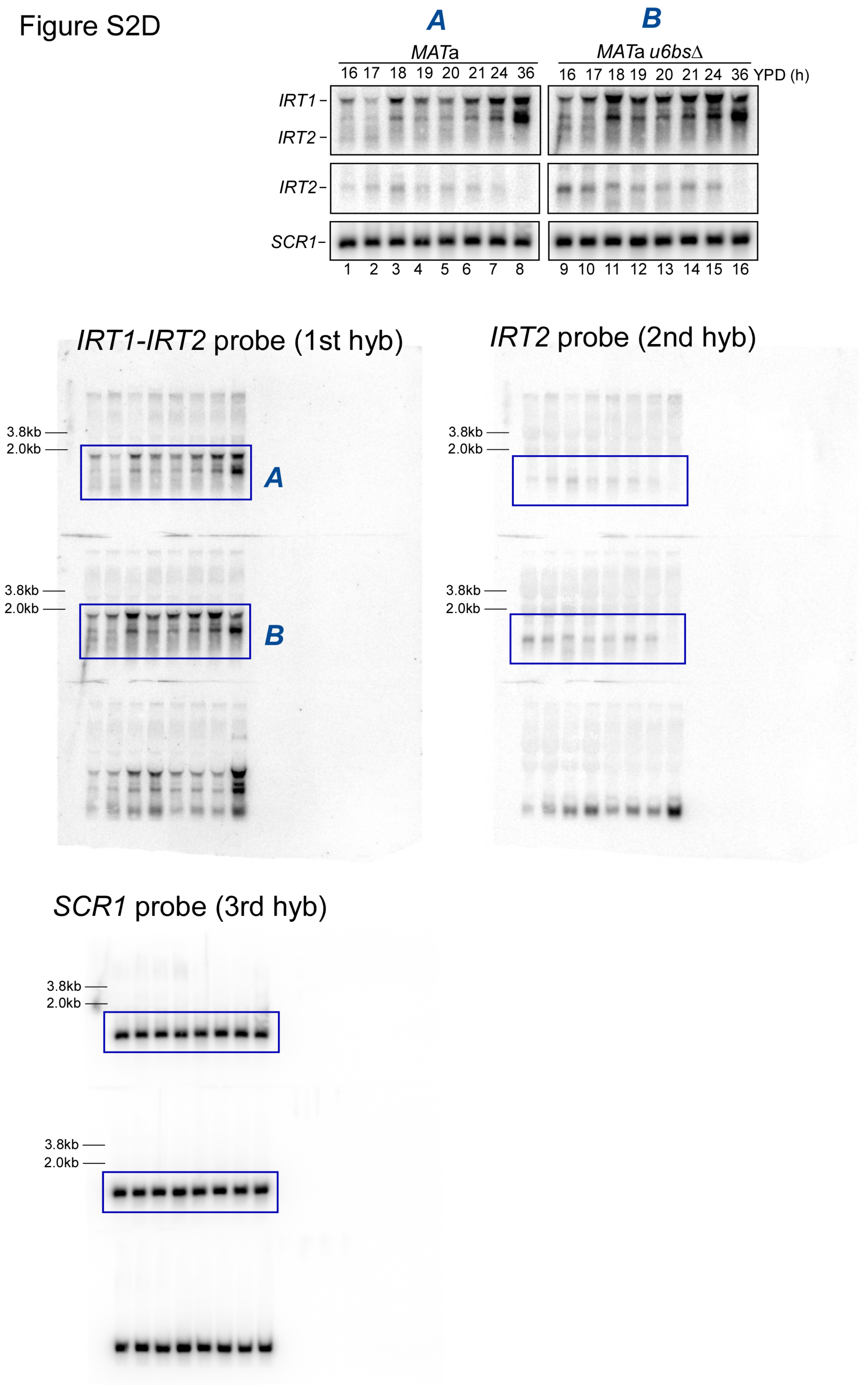

A
B
MATa
MATa u6bsΔ
16 17 18 19 20 21 24 36
16 17 18 19 20 21 24 36 YPD (h)
IRT1
IRT2
IRT2
SCR1
1 2 3 4 5 6 7 8
9 10 11 12 13 14 15 16
IRT1-IRT2 probe (1st hyb)
IRT2 probe (2nd hyb)
3.8kb
2.0kb
A
B
SCR1 probe (3rd hyb)

Figure S2F

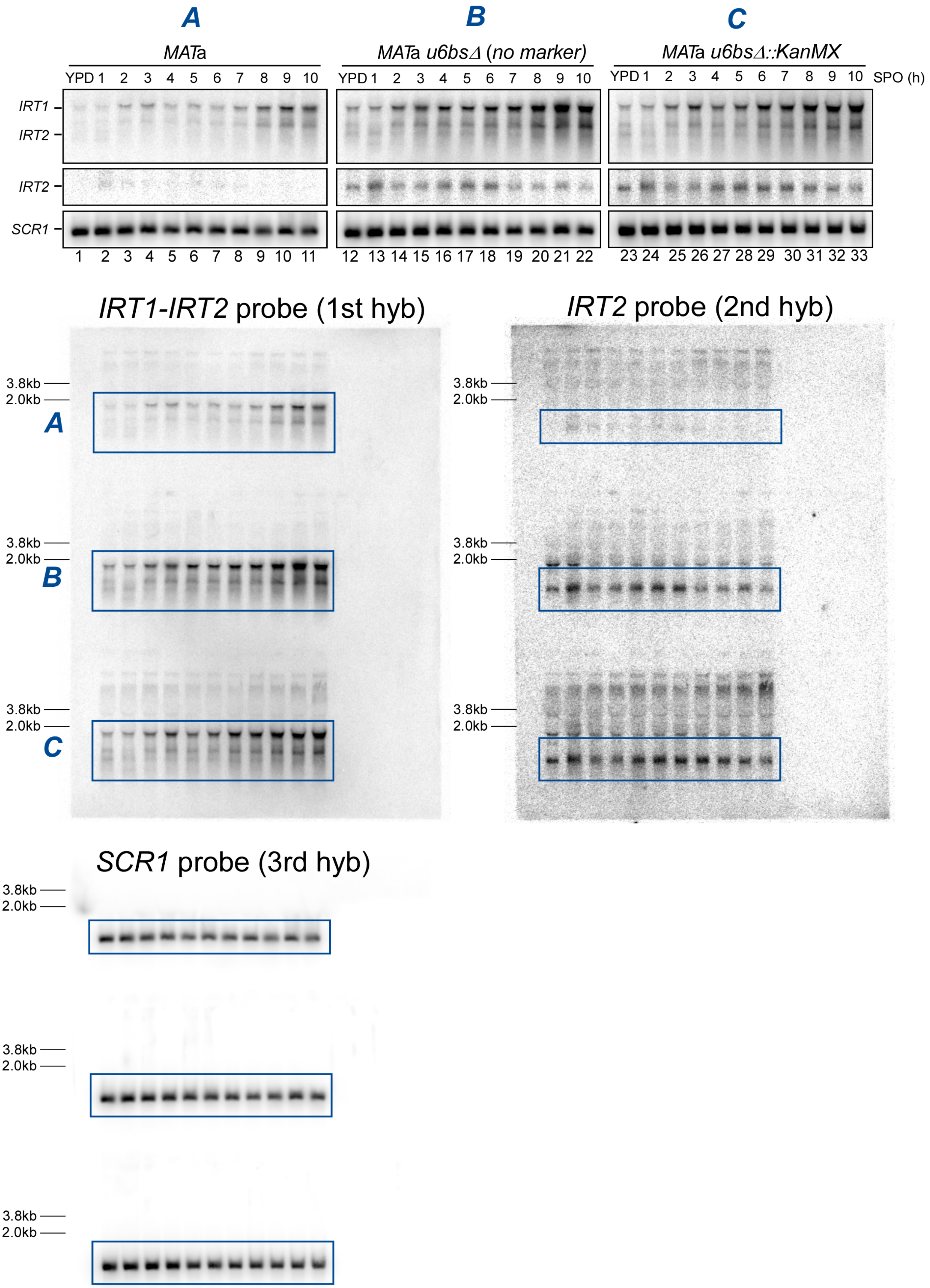
A
B
C
MATa
MATa u6bsΔ (no marker)
MATa u6bsΔ::KanMX
YPD 1 2 3 4 5 6 7 8 9 10
SPO (h)
IRT1
IRT2
SCR1
1 2 3 4 5 6 7 8 9 10 11
12 13 14 15 16 17 18 19 20 21 22
23 24 25 26 27 28 29 30 31 32 33
IRT1-IRT2 probe (1st hyb)
IRT2 probe (2nd hyb)
SCR1 probe (3rd hyb)
3.8kb
2.0kb

Figure S2H

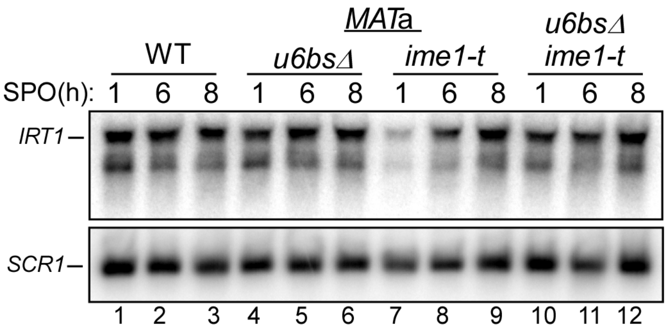
MATa
WT
u6bsΔ
ime1-t
u6bsΔ
ime1-t
SPO(h): 1 6 8 1 6 8 1 6 8 1 6 8
IRT1
SCR1
1 2 3 4 5 6 7 8 9 10 11 12

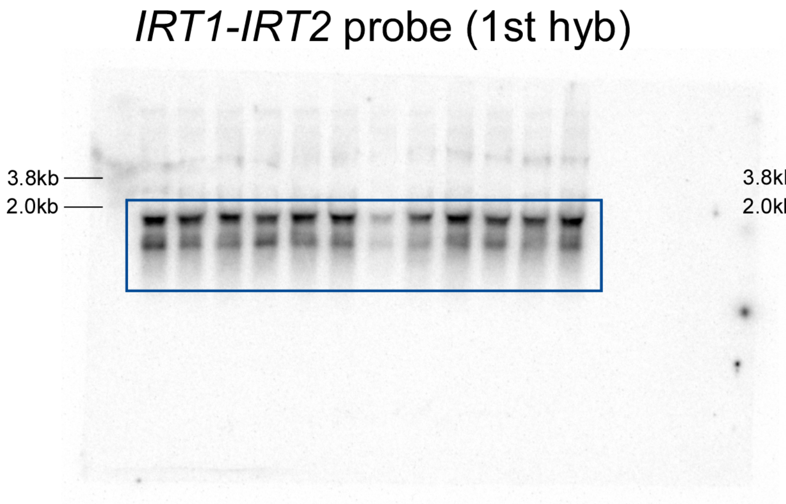
IRT1-IRT2 probe (1st hyb)
3.8kb
2.0kb

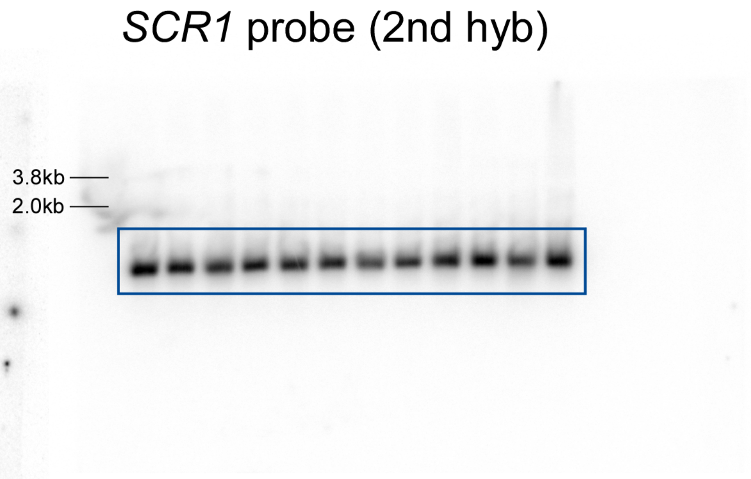
SCR1 probe (2nd hyb)
3.8kb
2.0kb

Figure S2I

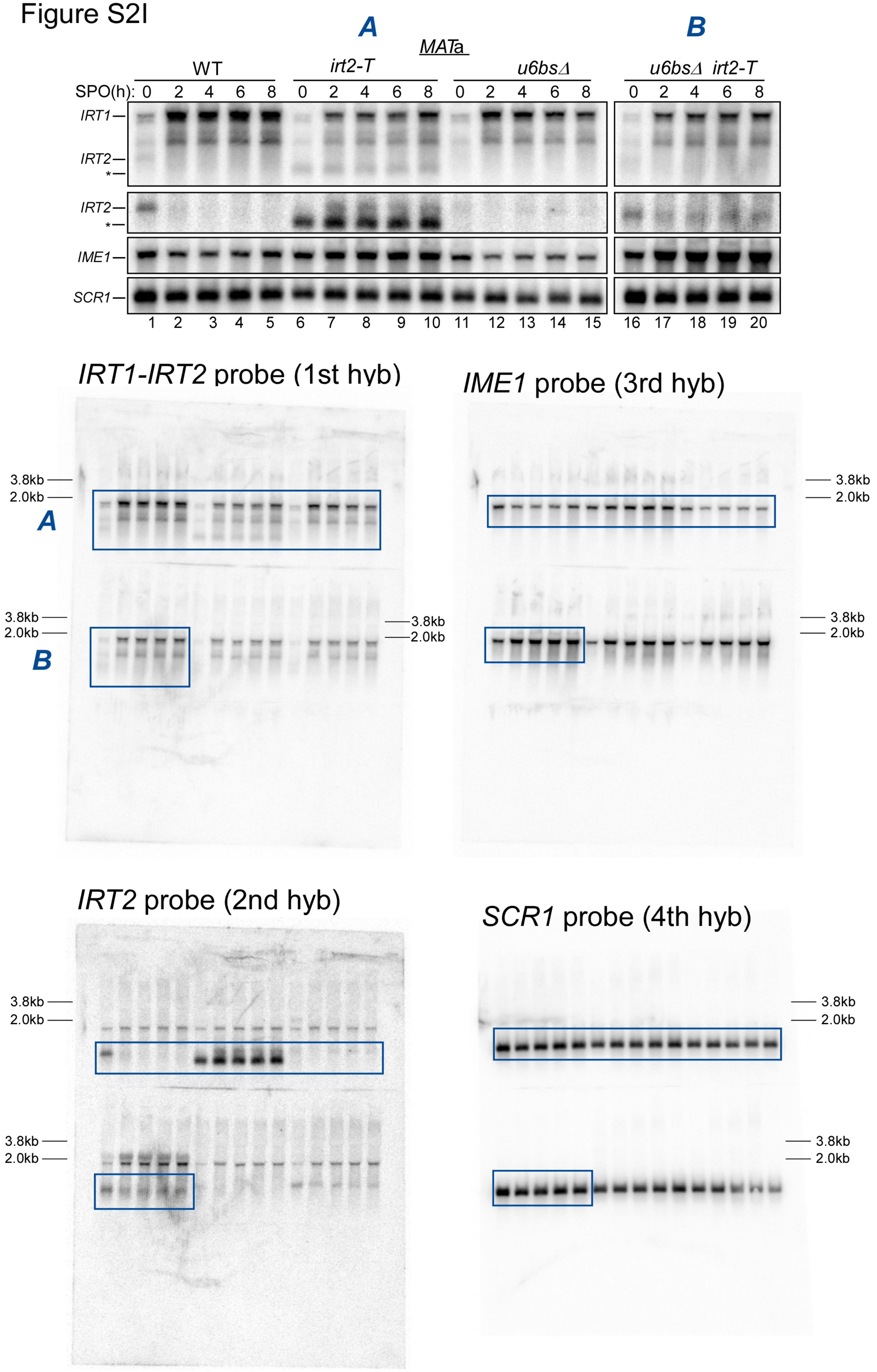
A
B
MATa
WT
irt2-T
u6bsΔ
u6bsΔ irt2-T
SPO(h): 0 2 4 6 8 0 2 4 6 8 0 2 4 6 8 0 2 4 6 8
IRT1
IRT2
*
IRT2
*
IME1
SCR1
1 2 3 4 5 6 7 8 9 10 11 12 13 14 15 16 17 18 19 20
IRT1-IRT2 probe (1st hyb)
IME1 probe (3rd hyb)
IRT2 probe (2nd hyb)
SCR1 probe (4th hyb)
3.8kb
2.0kb

Figure S2J

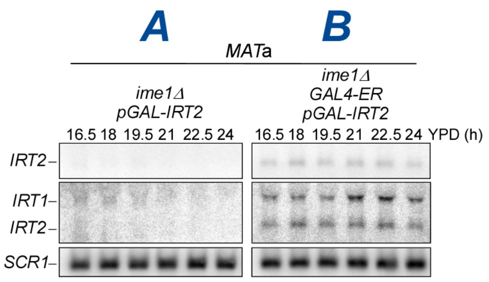

IRT2 probe (1st hyb)

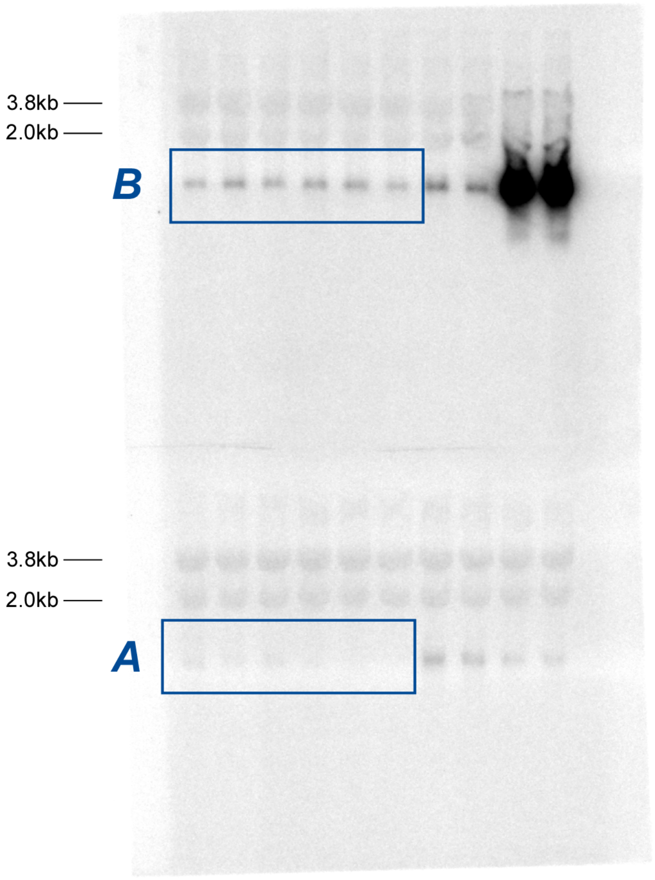

IRT1-IRT2 probe (2nd hyb)

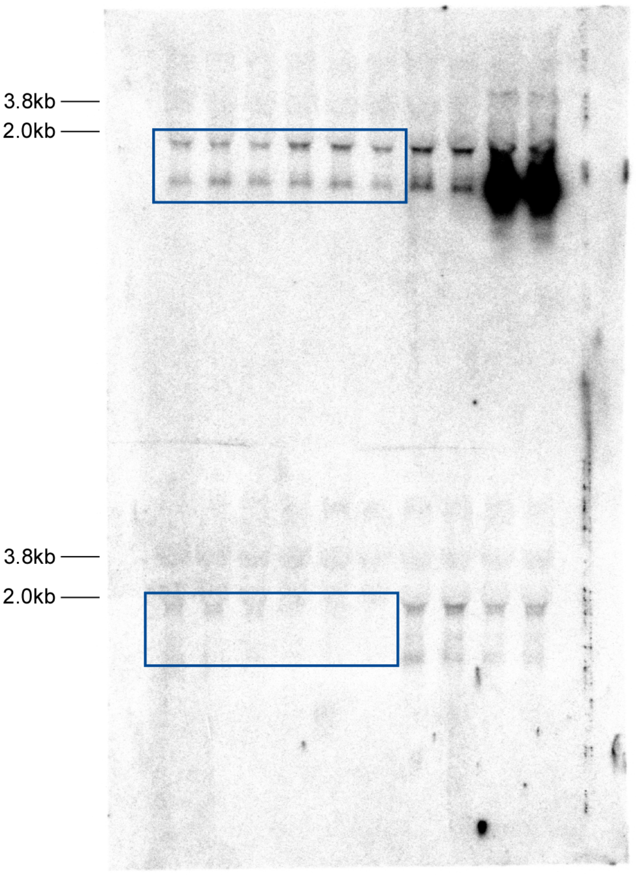

SCR1 probe (3rd hyb)

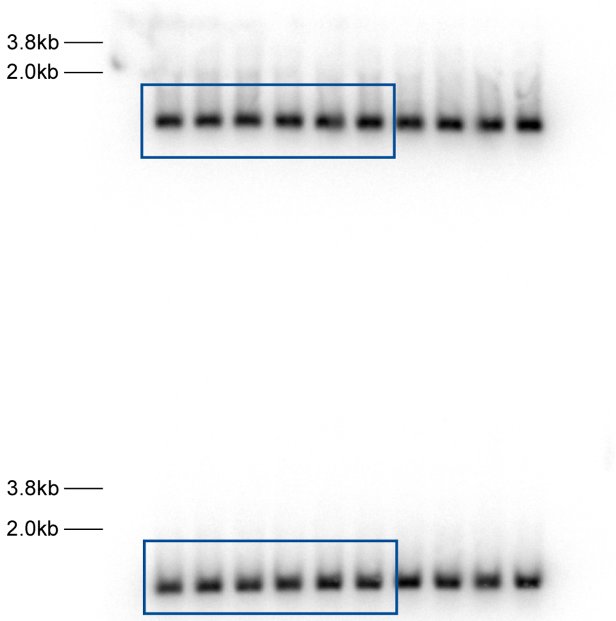

Figure S4A

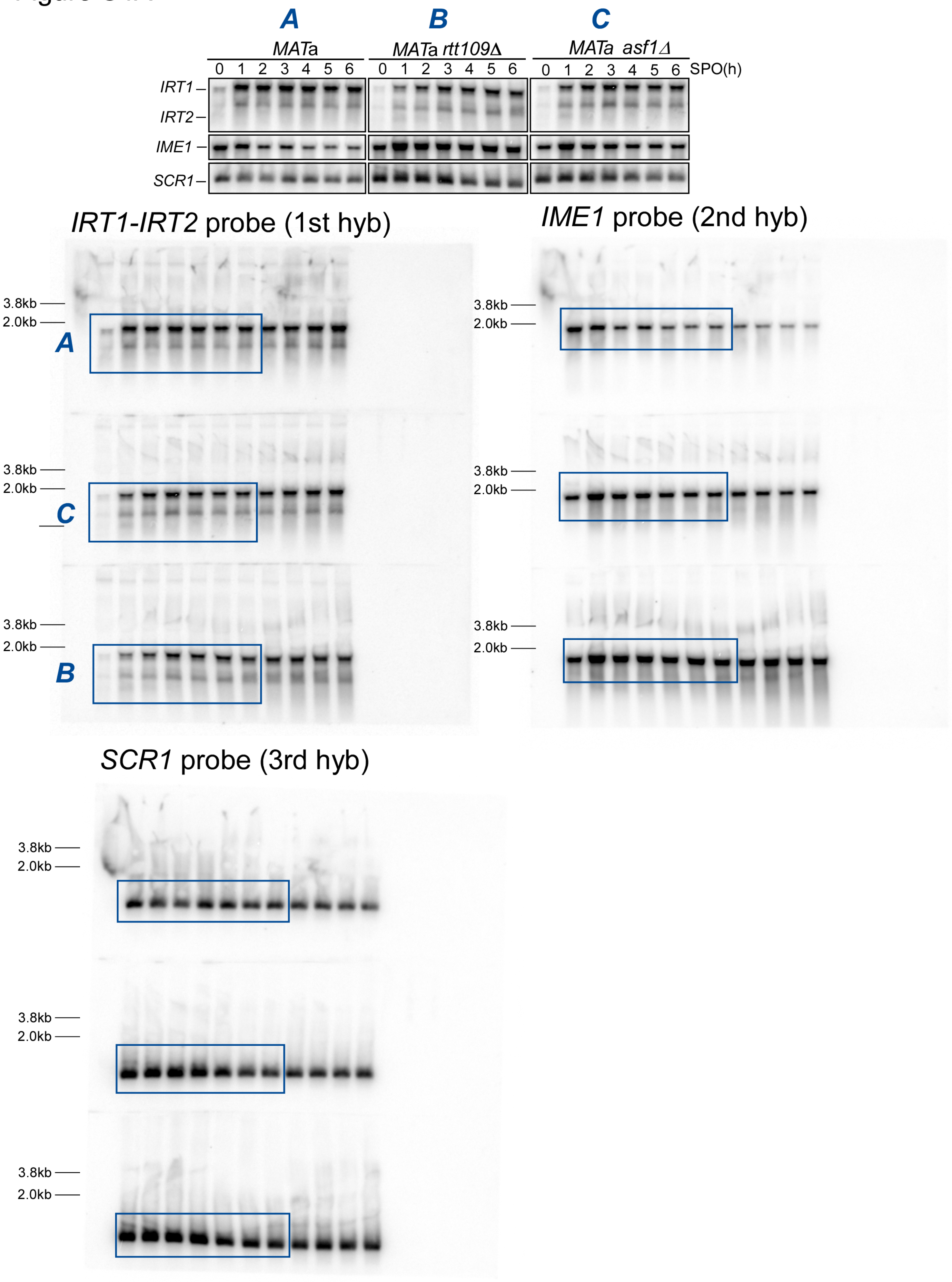

A
B
C
MATa
MATa rtt109Δ
MATa asf1Δ
0 1 2 3 4 5 6
SPO(h)
IRT1
IRT2
IME1
SCR1
IRT1-IRT2 probe (1st hyb)
IME1 probe (2nd hyb)
SCR1 probe (3rd hyb)
3.8kb
2.0kb

Figure S5C

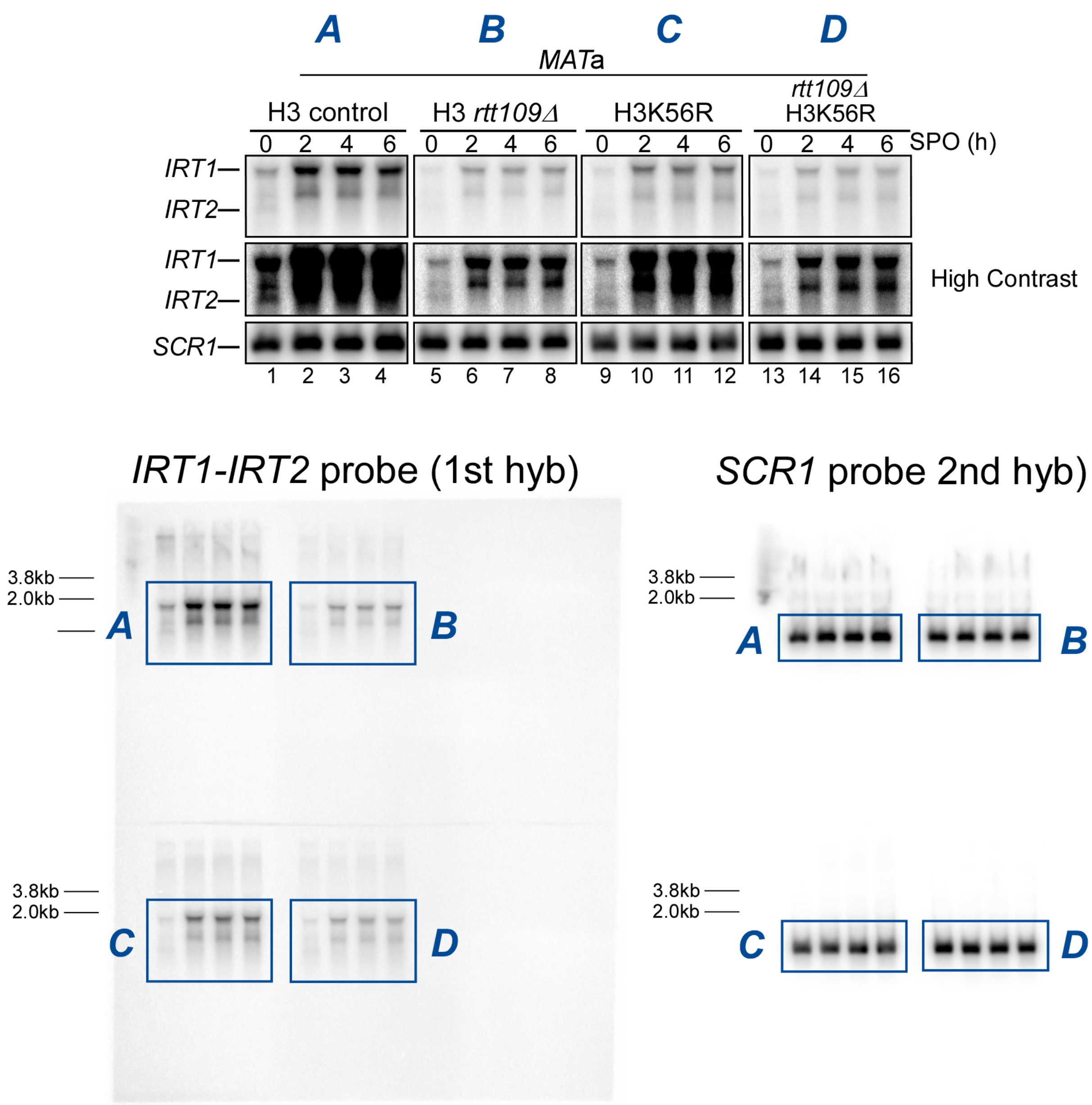
A B C D
MATa
H3 control
H3 rtt109Δ
H3K56R
rtt109Δ H3K56R
0 2 4 6 0 2 4 6 0 2 4 6 0 2 4 6 SPO (h)
IRT1
IRT2
IRT1
IRT2
High Contrast
SCR1
1 2 3 4 5 6 7 8 9 10 11 12 13 14 15 16
IRT1-IRT2 probe (1st hyb)
SCR1 probe 2nd hyb)
3.8kb
2.0kb
A B C D

Figure S6C

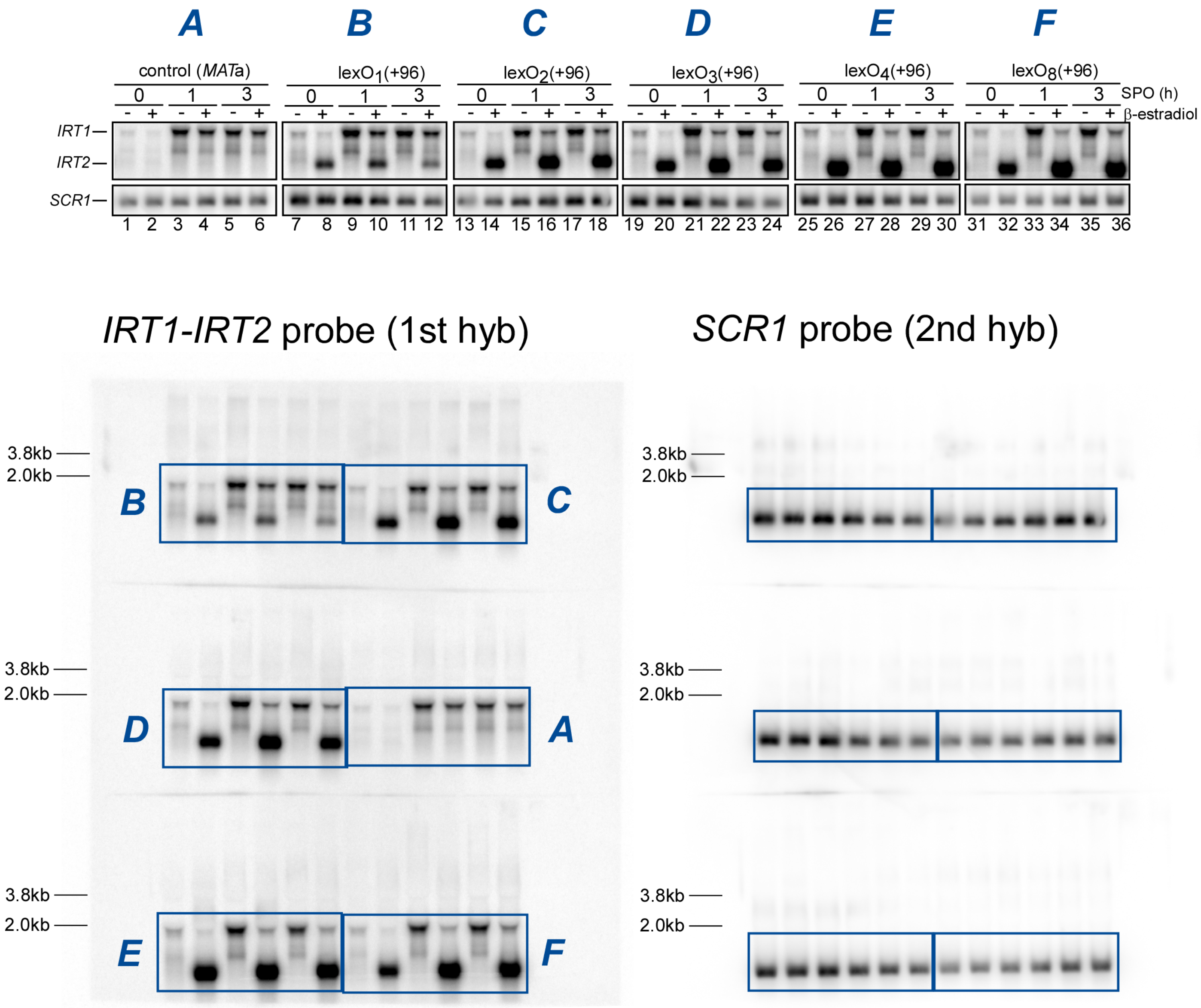

A
B
C
D
E
F
control (MATa)
lexO1(+96)
lexO2(+96)
lexO3(+96)
lexO4(+96)
lexO8(+96)
SPO (h)
β-estradiol
IRT1
IRT2
SCR1
1 2 3 4 5 6 7 8 9 10 11 12 13 14 15 16 17 18 19 20 21 22 23 24 25 26 27 28 29 30 31 32 33 34 35 36
IRT1-IRT2 probe (1st hyb)
SCR1 probe (2nd hyb)
3.8kb
2.0kb
