# Supplementary material for: Transcription levels of a noncoding RNA orchestrate opposing regulatory and cell fate outcomes in yeast
Source: Cell Rep. 2021 Jan 19;34(3):108643. doi: 10.1016/j.celrep.2020.108643 (PMC7816125; doi:10.1016/j.celrep.2020.108643)
Supplement: Document S2. Article plus supplemental information [file mmc3.pdf]

Cell Reports

Article

Transcription levels of a noncoding RNA orchestrate opposing regulatory and cell fate outcomes in yeast

Graphical Abstract

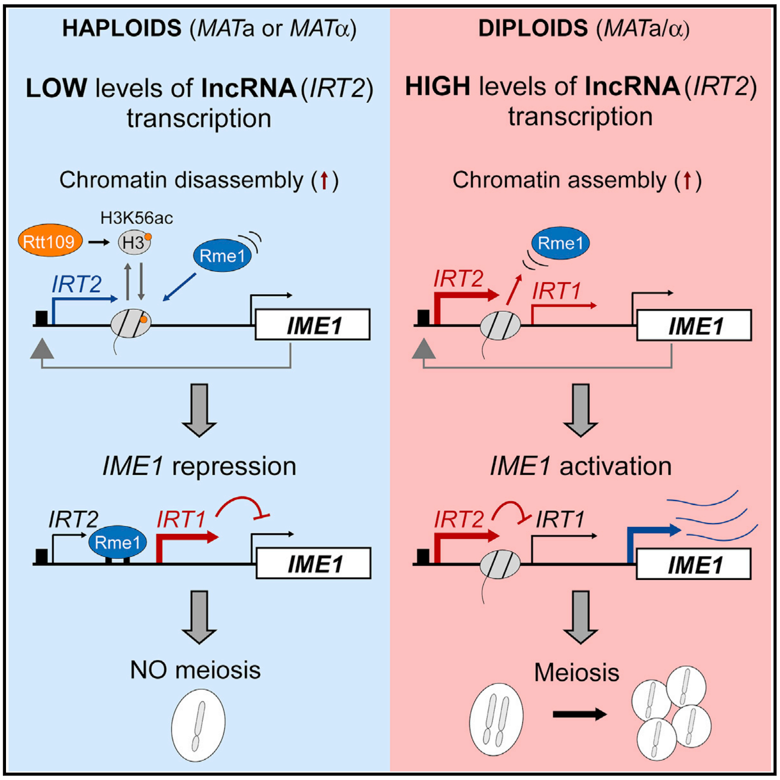

Authors

Fabien Moretto, N. Ezgi Wood, Minghao Chia, Cai Li, Nicholas M. Luscombe, Folkert J. van Werven

Correspondence

folkert.vanwerven@crick.ac.uk

In Brief

Moretto et al. demonstrate that transcription levels of a lncRNA named *IRT2* can have opposing effects on local gene transcription, thereby controlling entry into yeast meiosis. Low levels of *IRT2* promote H3K56ac, chromatin disassembly, transcription factor recruitment, and, thus, transcriptional activation. Increasing levels promote chromatin assembly and, consequently, transcriptional repression.

Highlights

- Transcription levels of the lncRNA *IRT2* regulate opposing local transcription states
- Low levels of *IRT2* promote H3K56ac, chromatin disassembly, and TF recruitment
- Increasing *IRT2* levels promote chromatin assembly and transcriptional repression
- *IRT2* transcription shapes the regulatory circuit for cell-type-specific control of yeast meiosis

Moretto et al., 2021, Cell Reports *34*, 108643
January 19, 2021 © 2020 The Author(s).
https://doi.org/10.1016/j.celrep.2020.108643

Cell Reports

CellPress
OPEN ACCESS

Article

Transcription levels of a noncoding RNA orchestrate opposing regulatory and cell fate outcomes in yeast

Fabien Moretto,[1,2] N. Ezgi Wood,[3] Minghao Chia,[1,4] Cai Li,[5,6] Nicholas M. Luscombe,[5,7,8] and Folkert J. van Werven[1,9,*]
[1]Cell Fate and Gene Regulation Laboratory, The Francis Crick Institute, 1 Midland Road, London NW1 1AT, UK
[2]Institute of Molecular Biology and Biotechnology, Foundation for Research and Technology Hellas, Heraklion, Crete 70013, Greece
[3]Department of Cell Biology, UT Southwestern Medical Center, 6000 Harry Hines Boulevard, Dallas, TX 75390, USA
[4]Genome Institute of Singapore, 60 Biopolis Street, Genome, #02-01, Singapore 138672, Singapore
[5]Bioinformatics and Computational Biology Laboratory, The Francis Crick Institute, 1 Midland Road, London NW1 1AT, UK
[6]School of Life Sciences, Sun Yat-sen University, Guangzhou, China
[7]Okinawa Institute of Science and Technology Graduate University, Okinawa 904-0495, Japan
[8]UCL Genetics Institute, University College London, London WC1E 6BT, UK
[9]Lead contact
*Correspondence: folkert.vanwerven@crick.ac.uk
https://doi.org/10.1016/j.celrep.2020.108643

SUMMARY

Transcription through noncoding regions of the genome is pervasive. How these transcription events regulate gene expression remains poorly understood. Here, we report that, in *S. cerevisiae*, the levels of transcription through a noncoding region, *IRT2*, located upstream in the promoter of the inducer of meiosis, *IME1*, regulate opposing chromatin and transcription states. At low levels, the act of *IRT2* transcription promotes histone exchange, delivering acetylated histone H3 lysine 56 to chromatin locally. The subsequent open chromatin state directs transcription factor recruitment and induces downstream transcription to repress the *IME1* promoter and meiotic entry. Conversely, increasing transcription turns *IRT2* into a repressor by promoting transcription-coupled chromatin assembly. The two opposing functions of *IRT2* transcription shape a regulatory circuit, which ensures a robust cell-type-specific control of *IME1* expression and yeast meiosis. Our data illustrate how intergenic transcription levels are key to controlling local chromatin state, gene expression, and cell fate outcomes.

INTRODUCTION

Transcription in intergenic regions of the genome is widespread. The noncoding RNAs emanating from these transcription events embody a large fraction of the transcriptome (Hon et al., 2017; Iyer et al., 2015; Kung et al., 2013). Particularly, the long noncoding RNAs (lncRNAs) constitute a diverse class of transcripts that control various cellular processes, including cell differentiation and stress, and have been implicated in diseases such as cancer (Guttman et al., 2011; Ponting et al., 2009; Wapinski and Chang, 2011). Despite extensive efforts, the function of the majority of noncoding RNAs remains poorly understood.

Noncoding transcription occurs near protein coding genes in promoter regions or at the 3′ ends of genes where they produce sense or antisense lncRNA transcripts (Neil et al., 2009; Pelechano and Steinmetz, 2013; Tisseur et al., 2011; Xu et al., 2009). Noncoding transcription can locally regulate the expression of coding genes through various mechanisms (Gil and Ulitsky, 2020; Wang and Chang, 2011). A widespread mechanism by which noncoding regions regulate gene expression is through the act of transcription via RNA polymerase II (Pol II) (Kornienko et al., 2013). During noncoding transcription, Pol II recruits chromatin remodelers that modify chromatin locally, thereby regulating the transcription of nearby genes (Ard et al., 2017; Venkatesh and Workman, 2015).

Noncoding transcription events can repress and activate gene transcription. Examples from yeasts have demonstrated that transcription through promoters of protein coding genes exerts gene repression (Ard et al., 2014; Bumgarner et al., 2009; Latos et al., 2012; Martens et al., 2004; Rom et al., 2019; van Werven et al., 2012). At these loci, chromatin regulators such as Facilitator of Chromatin Transcription (FACT), Set2, SET3C, and others mediate repression evoked by lncRNA transcription (Ard and Allshire, 2016; Hainer et al., 2011; Kim et al., 2012; van Werven et al., 2012). Conversely, noncoding transcription can also stimulate opening of chromatin and promote coding gene transcription (Hirota et al., 2008; Takemata et al., 2016). Transcription through enhancers, which produces enhancer RNAs (eRNAs), can contribute to enhancer activity and, thus, promotes gene expression (Li et al., 2016). How some noncoding transcription events promote while others repress gene expression remains unclear.

Two lncRNAs are transcribed in the promoter of the Inducer of Meiosis 1 gene, *IME1* (Moretto et al., 2018; van Werven et al.,

This is an open access article under the CC BY license (http://creativecommons.org/licenses/by/4.0/).

2012). This master transcription factor (TF) controls the cell fate decision of whether to enter meiosis (Kassir et al., 1988; Nachman et al., 2007). In diploid cells, expression of Ime1 activates the so-called early meiotic genes, thereby driving meiotic entry and the production of four haploid spores (Primig et al., 2000; van Werven and Amon, 2011). The *IME1* gene is highly regulated at the level of transcription through its unusually large promoter (about 2.5 kb), at which nutrient and mating-type signals integrate (Tam and van Werven, 2020; van Werven and Amon, 2011). These signals ensure that *IME1* transcription is only induced in cells expressing both mating-type loci (*MAT*a and *MAT*α) under starvation conditions.

Mating-type control of *IME1* expression is mediated by the transcription of two lncRNAs in the *IME1* promoter (Moretto et al., 2018; van Werven et al., 2012). In cells with a single mating type (*MAT*a or *MAT*α), typically haploid cells, transcription of a lncRNA named *IME1* regulating transcript 1 (*IRT1*) represses the *IME1* promoter to prevent meiotic entry (van Werven et al., 2012). The act of *IRT1* transcription establishes a repressed chromatin state in the *IME1* promoter, where TFs important for *IME1* activation bind (Kahana et al., 2010; Sagee et al., 1998; Tam and van Werven, 2020; van Werven et al., 2012). In *MAT*a/α diploid cells, *IRT1* transcription is reduced, because the a1α2 heterodimer (expressed from opposite mating-type loci) represses the transcriptional activator of *IRT1*, *RME1*, enabling *IME1* induction and, thus, meiotic entry (Figure 1A) (Mitchell and Herskowitz, 1986; van Werven et al., 2012). Despite the presence of a1α2 in *MAT*a/α diploid cells, Rme1 (and, thus, *IRT1*) is expressed to moderate levels in various genetic backgrounds (Deutschbauer and Davis, 2005; Gerke et al., 2009). To overcome *IRT1* transcription in *MAT*a/α diploid cells, Ime1 activates the transcription of a second lncRNA, named *IRT2*, located upstream in its own promoter (Moretto et al., 2018). Transcription through *IRT2*, in turn, interferes with Rme1 recruitment, represses *IRT1* transcription, and thereby promotes *IME1* expression (Figure 1A).

Here, we report the surprising finding of a dual function for transcription of the more upstream lncRNA in the *IME1* promoter, *IRT2*. We show that the levels of *IRT2* transcription regulate opposing chromatin and transcription states to ensure a robust transition from nutrient to mating-type control of the *IME1* promoter. Low levels of *IRT2* transcription direct H3 lysine 56 acetylation to chromatin, thereby promoting disassembly of chromatin, Rme1 recruitment, and activation of *IRT1* transcription. Remarkably, increasing transcription converts *IRT2* into a repressor. The dual function of *IRT2* transcription shapes the regulatory circuit that ensures that only cells expressing opposite, but not one of either, mating-type loci (*MAT*a and *MAT*α) enter meiosis in yeast. Our data illustrate how noncoding transcription levels are key to controlling local chromatin state, gene expression, and cell fate outcomes.

RESULTS

IRT2 is required for repression of *IME1* in cells with a single mating type

We hypothesized that there is a mechanism to ensure a robust transition from nutrient to mating-type control of yeast meiosis, involving the two lncRNAs expressed in the *IME1* promoter. To examine this, we determined *IRT1* and *IRT2* expression levels and mapped the transcription start sites (TSSs) and polyadenylation sites (transcription end sites; TESs) of both transcripts in cells harboring a single mating type (*MAT*a) and both mating types (*MAT*a/α) in the sporulation-proficient SK1 strain background. As expected, *MAT*a/α diploid cells entering meiosis synchronously induced *IME1* expression rapidly and displayed strong induction of *IRT2*, whereas *IRT1* levels remained relatively low (Figures 1A, 1B, and S1A) (van Werven et al., 2012). In these cells (6 h in sporulation medium [SPO]; Figures 1C and S1B), we detected a single *IRT2* TES while multiple TSSs spread over ~215 base pairs (bp) were detected, matching the slight smear observed on the northern blot for the *IRT2* transcript (Figure 1B). In haploid (*MAT*a) cells, *IRT1* expression was higher than in *MAT*a/α cells, and *IME1* expression was repressed (Figures 1A and 1B). The *IRT1* TSS mapped to a single region, while three TES regions were mapped: one to the middle of *IRT1* and two near the 3′ end (Figures 1C and S1B). Indeed, two distinct *IRT1* species were detected by northern blot, which were reduced to one truncated transcript when *IRT1* transcription was terminated early (*irt1-T*) (Figure 1B, lanes 7–10; Figure S1C). Surprisingly, we also detected low levels of *IRT2* expression before *IRT1* induction in *MAT*a cells (Figure 1B, lane 6). The *IRT2* transcript, TSS, and TES were also detectable at low levels in starved *MAT*a cells (SPO 4 h), demonstrating that *IRT2* is also expressed in this cell type (Figure S1D). To capture the *IRT2* expression window in haploid *MAT*a cells, we sampled during conditions for slow induction of *IME1* and *IRT1* expression (Figure S1A). Strikingly, we detected *IRT2* expression in several time points before *IRT1* induction (Figure 1D). Since the *IME1* promoter is highly regulated by nutrient signaling, the expression of *IRT2* that we detected may reflect changes in nutrient environment, such as glucose availability (van Werven and Amon, 2011).

To test whether *IRT2* is required for *IRT1* induction, we created deletions in the *IRT2* TSS cluster, Δ*irt2*(−188) and Δ*irt2*(−246), while keeping the Rme1 binding sites intact (Figures 1E, S1E, and S1F). Remarkably, in Δ*irt2*(−188) and Δ*irt2*(−246) cells, *IRT1* expression decreased; *IME1* levels increased; and, as expected, *IRT2* expression was not detectable. Both *IRT2* mutants also displayed reduced association of Rme1 to the *IRT1* promoter (Figure 1F). Thus, in addition to its transcriptional repressor function described in *MAT*a/α cells (Moretto et al., 2018), *IRT2* is also required for *IRT1* expression and repression of the *IME1* gene in cells with a single mating type.

The act of *IRT2* transcription is required and sufficient for activating *IRT1* expression

We next evaluated whether the act of transcription of *IRT2* contributes to *IRT1* activation. We integrated a transcriptional terminator between the *IRT2* TSS and the Rme1 binding sites to generate the *irt2-T* allele (Figure 2A). A shorter form of *IRT2* was detected in *irt2-T* cells (*IRT2**; Figure 2B). Remarkably, *irt2-T* cells showed diminished association of Rme1 with the *IRT1* promoter, reduced *IRT1* expression and Pol II binding to *IRT1*, and increased *IME1* expression (Figures 2B, 2C, and S2A). A control sequence (*irt-l*) did not alter *IRT1* and *IME1* expression (Figure S2B, lanes 2–5 and 12–15). In addition, a transcriptional terminator integrated into *IRT1* (*irt1-T*) showed wild-

CellPress
OPEN ACCESS

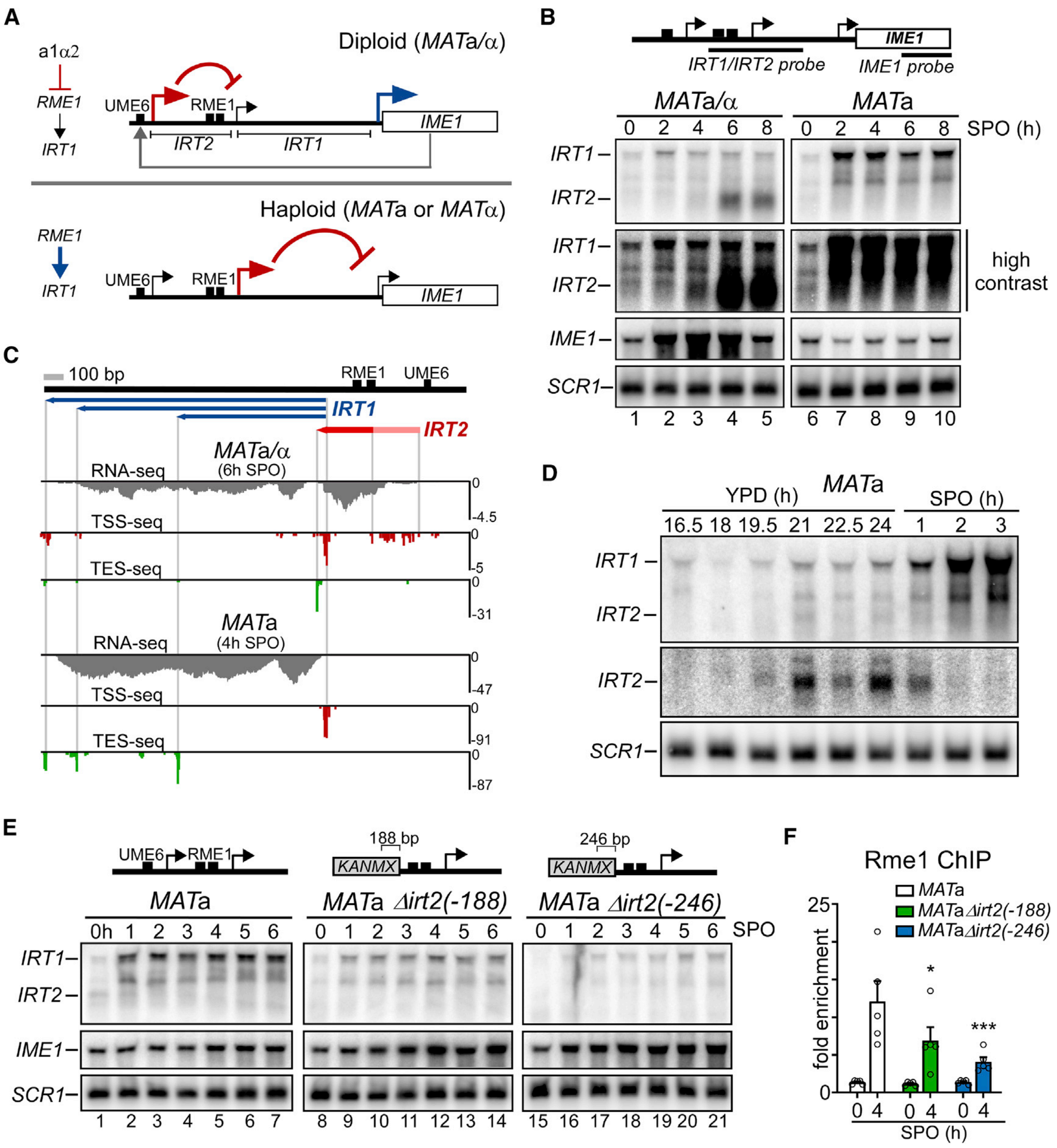

Figure 1. *IRT2* is required for activation of *IRT1* transcription

(A) Scheme of the two lncRNAs, *IRT1* and *IRT2*, expressed in the *IME1* promoter. In *MATa/α* (diploid) cells, *RME1*, the activator of *IRT1*, is repressed by a1α2. A feedback regulatory circuit consisting of *IRT2*, *IRT1*, and Ime1 facilitates *IME1* expression in diploids. In single-mating-type cells (*MATa* or *MATα*, haploids), Rme1 is expressed, and *IME1* expression is repressed by transcription of *IRT1*.

(B) *IRT1*, *IRT2* (combined probe), and *IME1* expression in *MATa/α* (FW1509) and *MATa* (FW1511) cells, as detected by northern blot. Cells were grown in rich medium (YPD) for 24 h and shifted to pre-sporulation medium (pre-SPO) for 16 h before being transferred and sampled in sporulation medium (SPO). *SCR1* was used as a loading control. High-contrast blots for *IRT1/IRT2* illustrate *IRT2* signal in haploids.

(C) *IRT1* and *IRT2* transcription start site sequencing (TSS-seq), transcription end site sequencing (TES-seq), and RNA-seq data for *MATa/α* (6 h SPO, top panel) and *MATa* (4 h SPO, bottom panel). Cells were grown as described in (B). Blue lines indicate different *IRT1* RNA isoforms. Red line indicates the *IRT2* transcript, and light red line indicates where *IRT2* transcription initiates. The y axes are in reads per million (RPMs).

(D) Haploid *MATa* cells (FW1509) were grown in YPD (24 h) before being transferred to SPO. An *IRT2*-specific probe is also featured.

(E) Similar to (B), except using *MATa* Δ*irt2*(−188) (FW1210) and Δ*irt2*(−246) (FW1356) mutants along with WT cells (FW1509).

(F) Rme1 association to the *IRT1* promoter as detected by ChIP in mutants described in (E) but also harboring *RME1* tagged with V5 epitope (FW4031, FW3132, and FW3140). Cells were grown as in (B). qPCR primer pair was nested over Rme1 binding sites in the *IRT1* promoter. Signals were normalized to *HMR*, which does not bind Rme1. The error bars represent the standard error of the mean (SEM) of n = 5. *p < 0.05; ***p < 0.005, compared to *MATa* control on a two-way ANOVA followed by a Fisher's least significant difference (LSD) test.

See also Figure S1.

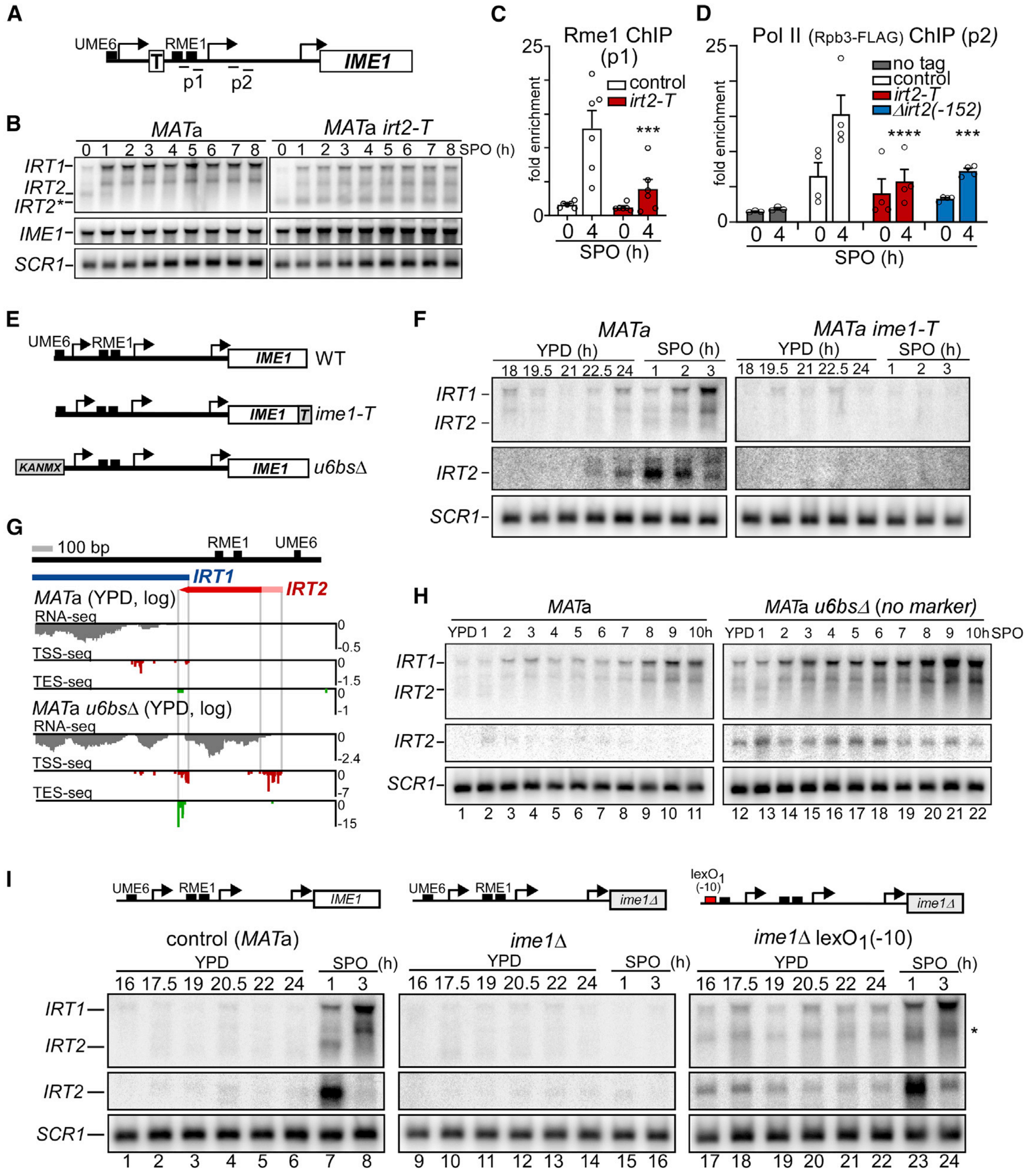

Figure 2. Transcription of *IRT2* is required for induction of *IRT1* expression

(A) Scheme of *IME1* promoter harboring a transcriptional terminator integrated between the *IRT2* TSS and the Rme1 binding site, *irt2-T*. Primer pairs (p1 and p2) used for ChIP in (C) and (D) are also depicted.

(B) *IRT1*, *IRT2*, and *IME1* expression in WT and *irt2-T MATa* cells (FW1509 and FW3596). The asterisk depicts the *IRT2* short form caused by early termination in *IRT2*.

(C) Rme1 association at the *IRT1* promoter in WT and *irt2-T MATa* cells (FW4031 and FW3128) by ChIP. qPCRs were performed using primer pair p1 depicted in (A) Error bars represent SEM; n = 6. ***$p < 0.005$, two-way ANOVA followed by a Fisher's LSD test.

(D) Pol II association at *IRT1* in *MATa* (control), *irt2-T*, and Δ*irt2*(−246) (FW8515, FW8512, and FW8510, respectively) cells expressing FLAG-tagged Rpb3 along with a no-tag control (FW1509). qPCRs were performed using the primer pair p2. Error bars represent SEM; n = 4, except for the no-tag condition (n = 3). ***$p < 0.0005$; ****$p < 0.0001$, two-way ANOVA followed by a Fisher's LSD test performed on the whole group of samples, including the one presented in Figure S2E.

(E) Schemes of the *ime1-T* mutant, which harbors point mutations in the C terminus of *IME1* (which impairs Ime1 function), and *u6bs*Δ, which harbors a deletion of the *IRT2* Ume6 binding site.

(F) *IRT1* and *IRT2* expression in WT and *ime1-T MATa* cells (FW1509 and FW2189) detected by northern blot. (G) *IRT2* transcript start and end sites in *u6bs*Δ cells determined by TSS-seq and TES-seq during exponential growth in YPD.

(legend continued on next page)

CellPress
OPEN ACCESS

type (WT)-like Rme1 recruitment and displayed no additional reduction in Rme1 binding when combined with Δ*irt2*(−246), indicating that transcription through *IRT1* is not required for inducing *IRT1* expression (Figure S2C).

Additionally, we modulated *IRT2* transcription without affecting the *IRT2* sequence. We reasoned that, if *IRT2* transcription was involved in *IRT1* activation, then changing *IRT2* expression should affect the *IRT1* expression pattern. Therefore, we first abrogated *IRT2* activation by introducing point mutations in Ime1 (*ime1-T*), which impairs Ime1 function (Moretto and van Werven, 2017). Little or no *IRT1* expression was detected in the absence of *IRT2* transcription (Figures 2E and 2F). Moreover, we constitutively expressed *IRT2* by deleting the Ume6 repressor binding site (*u6bs*Δ) in the *IRT2* promoter, which decouples *IRT2* expression from *IME1* activation (Figure 2E) (Moretto et al., 2018). To confirm that *u6bs*Δ gives rise to *IRT2* transcription, we mapped the transcript (Figure 2G). As expected, the *IRT2* start and end sites in *u6bs*Δ overlapped with positions we identified for *IRT2* in WT cells (Figures 1C and S1D). Constitutive levels of *IRT2* transcription (*u6bs*Δ) led to earlier *IRT1* transcription and earlier Rme1 recruitment (Figures 2H and S2D–S2G). Furthermore, *u6bs*Δ rescued the *IRT1* expression defect observed when Ime1 function was impaired (*ime1-T*), but not when *IRT2* transcription was terminated earlier (*irt2-T*) (Figure S2H, lanes 7–9 and 10–12; and S2I, lanes 12–15 and 17–20). We conclude that *IRT2* is required for *IRT1* activation and that Ime1 activates *IRT1* expression *via IRT2* transcription.

We next determined whether *IRT2* transcription was also sufficient for full activation of *IRT1*. To control *IRT2* transcription, we used a system that can titrate the levels of transcription using a combination of lexO sites together with LexA-ER (estrogen receptor) activator and β-estradiol inducer (Ottoz et al., 2014). We integrated a single lexO site, lexO(−10), upstream of the Ume6 binding site in *ime1*Δ cells, thus preserving *IRT2* repression (Figure 2I). Remarkably, we were able to rescue the *IRT1* expression defects of *ime1*Δ cells with a single lexO(−10) site without the need to activate LexA-ER with β-estradiol (Figure 2I, lanes 17–24). These cells displayed constitutive low levels of *IRT2* and marked levels of *IRT1* expression across all time points, despite the presence of the Ume6 binding site. A similar result was obtained when using the *GAL1-10* promoter (*pGAL-IRT2*) in cells harboring Gal4-ER (no β-estradiol) (Figure S2J). In the absence of Gal4-ER, we detected neither *IRT2* expression nor *IRT1* expression in *pGAL-IRT2* cells, indicating that Gal4-ER was required for inducing *IRT2* transcription and, consequently, *IRT1* (Figure S2J). Taken together, low levels of *IRT2* transcription are required and sufficient for induction of *IRT1* expression in cells with a single mating type.

IRT2 transcription prevents meiosis in cells with a single mating type

Mis-expression of meiotic genes can have detrimental consequences to haploid cells (Lino and Yamamoto, 1985; Wagstaff et al., 1982). Haploid cells harboring a single mating type, but lacking Rme1 or *IRT1*, undergo a lethal type of meiosis (van Werven et al., 2012). We sought to determine the importance of *IRT2*-mediated activation of *IRT1* in preventing haploid cells from entering meiosis. We found that in *irt2* mutants Δ*irt2*(−246) and *irt2-T*, a large fraction of cells displayed high levels of *IME1* expression (more than 30 mRNA copies per cell) (Figures 3A, S3A, and S3B). After a prolonged period of starvation, *irt2* mutants Δ*irt2*(−188), Δ*irt2*(−246), and *irt2-T* also displayed reduced viability, possibly due to entering meiosis (Figure 3B). We also generated diploid cells with a single mating type (*MAT*a/a), mimicking mating-type repression of *IME1* expression. Approximately 30% to 40% of *MAT*a/a diploid cells for each *irt2* mutant underwent at least one meiotic division (Figure 3C). This was comparable to the *rme1* mutant that has impaired *IRT1* transcription. Thus, *IRT2* is essential for inhibiting meiotic entry in starved cells with a single mating type.

Rtt109 facilitates *IRT2* transcription-mediated *IRT1* activation

To gain insight into the mechanism by which *IRT2* transcription stimulates *IRT1* expression, we screened for mutants that displayed decreased *IRT1* and increased *IME1* expression. We selected a small set of known histone-modifying enzymes as well as several Pol II machinery factors. Gene deletions affecting Pol II transcription fidelity (*RPB9* and *CTK1*), histone acetylation (*GCN5* and *RTT109*), and histone chaperone function (*ASF1*) were identified (Figure 4A; Table S1). We focused our analyses on two candidate genes: *RTT109* and *ASF1*. Rtt109 is the sole histone acetyltransferase that acetylates histone H3 lysine 56 (H3K56ac) in yeast, whereas Asf1 is involved directly in chromatin assembly and acts as a chaperone for Rtt109-directed H3K56ac (Driscoll et al., 2007; Masumoto et al., 2005; Recht et al., 2006; Schneider et al., 2006; Tsubota et al., 2007). H3K56ac-marked histones are assembled into nucleosomes during DNA replication, where they buffer gene transcription, but they are also present at promoters, where they are incorporated into nucleosomes in a replication-independent manner (Kaplan et al., 2008; Rufiange et al., 2007; Schneider et al., 2006; Voichek et al., 2016; Williams et al., 2008; Xu et al., 2005). Furthermore, nucleosomes harboring H3K56ac mark active transcription and active enhancers in higher eukaryotes (Schneider et al., 2006; Skalska et al., 2015; Värv et al., 2010).

We found that, in *rtt109*Δ—and, to a lesser extent, in *asf1*Δ *MAT*a cells—*IRT1* expression was reduced and *IME1* expression levels were increased (Figures 4B and S4A). Importantly, Rme1 protein levels were not affected in *rtt109*Δ cells (Figure S4B). In addition to steady-state RNA measurements, we also determined whether *IRT1* and *IME1* transcription (nascent RNA sequencing [RNA-seq] and Pol II chromatin immunoprecipitation [ChIP]) were affected in *rtt109*Δ cells during starvation (Figures 4C–4E, S4C, and S4D). *rtt109*Δ cells displayed reduced

(H) Same as (F), except that WT cells (FW1509, lanes 1–11) and *u6bs*Δ *MATa* cells (FW2438, lanes 12–22) were used, and cells were shifted to SPO before saturation.

(I) *MAT*a WT cells (FW1509, lanes 1–8), or cells harboring *ime1*Δ together with the WT *IRT2* promoter (FW1555, lanes 9–16) or a $lexO_1$(−10) site (FW7142, lanes 17–24) were grown as in (F), and samples were taken at the indicated time points. Asterisk indicates a longer version of *IRT2* originated from $lexO_1$(−10) that has about the same size of one *IRT1* isoform.

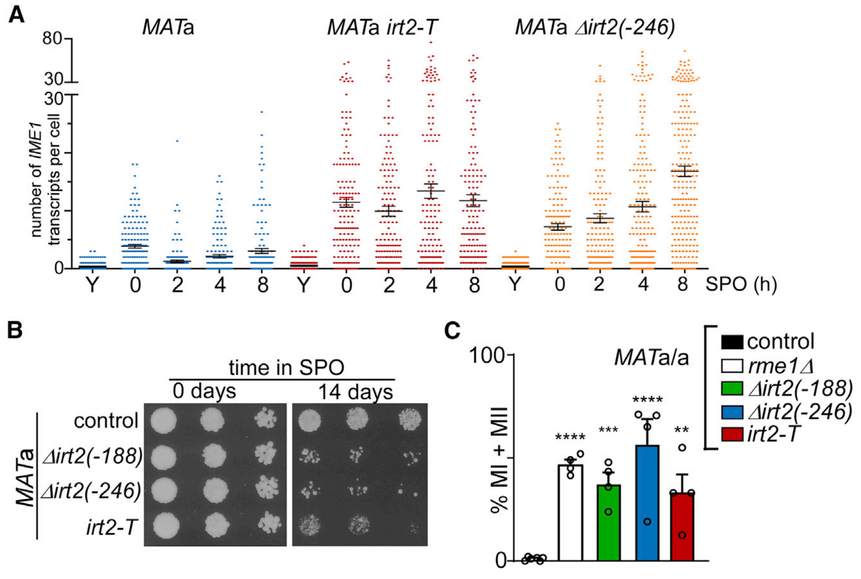

Figure 3. Transcription of *IRT2* prevents meiotic entry in cells with a single mating type

(A) *IME1* expression in single cells, as measured by single-molecule RNA fluorescence *in situ* hybridization (FISH). *MAT*a control, Δ*irt2(−246)*, and *irt2-T* (FW1533, FW3580, and FW3585) cells. The strains used also harbored a *flo8* deletion. Formaldehyde-fixed cells were hybridized with probes directed against *IME1* and *ACT1*. Each dot represents the number of *IME1* transcripts in a single cell positive for *ACT1* expression. Error bars represent SEM; n = 150 cells.

(B) Spot assay of cells on rich medium agar plates in 5-fold serial dilutions after 0 or 14 days in SPO. *MAT*a control, Δ*irt2(−188)*, Δ*irt2(−246)*, and *irt2-T* cells (FW1509, FW1210, FW1356, and FW3596).

(C) *MAT*a/a diploid control cells (FW15) and cells harboring *rme1*Δ, Δ*irt2(−188)*, Δ*irt2(−246)*, or *irt2-T* (FW1317, FW3453, FW3402, and FW3629) were grown as described in (A). DAPI masses were counted from cells fixed at 72 h in SPO. Cells harboring >2 DAPI masses were considered to have undergone meiotic divisions (MI + MII). Error bars represent ±SEM; n = 4, except for the control sample (n = 6). **$p < 0.005$; ***$p < 0.0005$; and ****$p < 0.0001$, on a one-way ANOVA followed by Fisher's LSD test.

IRT1 transcription, less Pol II binding to *IRT1*, and increased *IME1* transcription. We conclude that Rtt109 regulates *IRT1* transcription.

One possible explanation for decreased *IRT1* expression in *rtt109*Δ cells is that *IRT2* transcription is affected. Therefore, we de-repressed *IRT2* transcription using two different genetic approaches in the *rtt109*Δ background. First, we measured *IRT1* expression in cells when *IRT2* transcription was driven from a single lexO(−10) site. Whereas *IRT2* was clearly expressed in lexO(−10) *rtt109*Δ cells, *IRT1* expression was still compromised, suggesting that Rtt109 acts in concert with *IRT2* transcription in inducing *IRT1* (Figures 4F and S4F). Second, we combined the *u6bs*Δ and *rtt109*Δ mutants. De-repression of *IRT2* transcription in *u6bs*Δ also did not rescue the *IRT1* expression defect in *rtt109*Δ cells (Figure 4G, lanes 6–10, 11–15, and 16–20; Figure S4E). Furthermore, the *IRT2* RNA was clearly detectable in *u6bs*Δ *rtt109*Δ and lexO(−10) *rtt109*Δ cells, suggesting that the *IRT2* RNA by itself has little function in *IRT2*-mediated activation of *IRT1* (Figures 4F and 4G, lane 16; Figure S4F). We conclude that Rtt109 likely acts downstream of *IRT2* transcription in activating *IRT1* transcription.

H3K56ac is directed to chromatin *via IRT2* transcription and prevents meiotic entry

To further dissect how Rtt109 contributes to *IRT1* activation, we combined the *rtt109*Δ with the early termination of *IRT2 (irt2-T)* allele and measured the effect on *IRT1* activation. *IRT1* expression in the *irt2-T rtt109*Δ double mutant was affected to a degree comparable to that of the single mutants (Figure 5A, lanes 6–10, 11–15, and 16–20; Figure S5A). Importantly, Rme1 recruitment was also affected to similar levels in the *irt2-T rtt109*Δ single and double mutants (Figure 5B). This suggests that Rtt109 acts downstream of *IRT2* transcription and facilitates Rme1 recruitment and *IRT1* activation.

Rtt109-mediated H3K56ac occurs off chromatin on newly synthesized histones (Driscoll et al., 2007; Han et al., 2007). In addition, H3K56ac can be incorporated in chromatin at promoter and transcribed regions in the genome (Rufiange et al., 2007; Schneider et al., 2006; Värv et al., 2010; Williams et al., 2008). To examine whether there is a link between *IRT2* transcription and H3K56ac, we evaluated whether transcription of *IRT2* directs H3K56ac to chromatin locally and whether H3K56ac mediates activation of *IRT1* transcription. When we measured H3K56ac levels in the *IRT2* region, we found that H3K56ac was enriched at the time of *IRT2* transcription (0 h and 3 h), but not when *IRT2* was repressed during exponential growth (YPD) or when *RTT109* was deleted (Figure 5C). Importantly, H3K56ac levels were reduced in *irt2-T* cells, further supporting that *IRT2* transcription is required for H3K56ac deposition (Figure 5C). This suggest that *IRT2* transcription facilitates H3K56ac deposition in chromatin.

Although the main substrate of Rtt109 is H3K56, it is also known to acetylate lysine 9 of histone H3 (Adkins et al., 2007). To examine the role of H3K56ac directly, we mutated the H3K56 residue to alanine or arginine (H3K56A and H3K56R) to mimic the absence of H3K56ac in cells. The H3K56R mutant—and, to a lesser extent, H3K56A—displayed reduced *IRT1* expression and increased *IME1* expression (Figure 5D, lanes 1–5 and 11–15; Figures S5C and S5D). Importantly, *IRT1* expression levels in the H3K56R mutant were affected to a degree comparable to that of the *rtt109*Δ H3K56R double mutant, suggesting that other targets of Rtt109 do not play a major role in *IRT2*-mediated activation of *IRT1* (Figure S5C, lanes 14–16, 10–12, and 6–8, and S5D). H3K56ac was also necessary to prevent meiotic entry in cells with a single mating type.

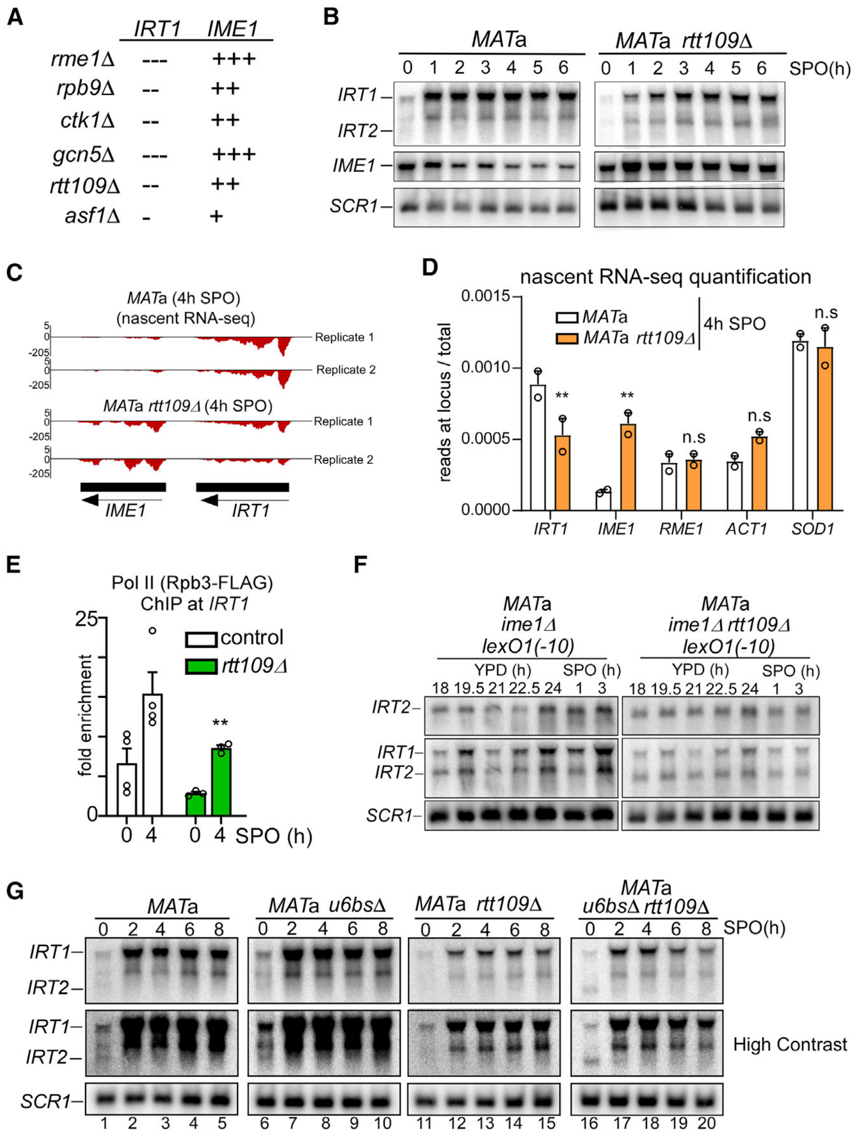

Figure 4. Rtt109 mediates *IRT2*-directed activation of *IRT1* transcription

(A) Candidate genes involved in *IRT2*-mediated activation of *IRT1*. The minus symbol represents lower *IRT1* expression compared to *MAT*a WT cells, and the plus symbol represents higher *IME1* expression compared to *MAT*a WT cells.

(B) *IRT1*, *IRT2*, and *IME1* expression in WT *MAT*a (FW1509) and *rtt109*Δ (FW4077) cells detected by northern blot.

(C) Nascent RNA-seq (Pol II-associated RNA) for the *IRT1* and *IME1* loci from *MAT*a control (FW4031) and *rtt109Δ* (FW4075) cells harvested 4 h in SPO. Two replicates are presented. Pol II was purified using the Rpb3-FLAG. The y axes indicate reads per million (RPM).

(D) Nascent RNA-seq read quantifications of selected transcripts (*IRT1*, *IME1*, and the controls *RME1*, *ACT1*, and *SOD1*) as obtained in (C). Error bars represent ±SEM; n = 2. **p < 0.005, parametric unpaired two-tailed Student's t test.

(E) Pol II ChIP with *IRT1* in control *MAT*a and *rtt109*Δ cells (FW8515 and FW8561). Error bars represent SEM; n ≥ 3. **p < 0.005, two-way ANOVA followed by a Fisher's LSD test performed on the whole group of samples, including those presented in Figure 2D.

(F) *IRT1* and *IRT2* expression in *MAT*a *ime1*Δ cells with a lexO$_1$(−10) site at *IRT2* (FW7142) and in the same cells including a *rtt109*Δ mutation (FW8555). Cells were grown in YPD (24 h) and shifted to SPO.

(G) *IRT1* and *IRT2* expression in control *MAT*a (FW1509), *u6bs*Δ (FW2438), *rtt109*Δ (FW4077), and *u6bs*Δ *rtt109*Δ double-mutant (FW5225) cells.

Approximately 20% to 30% of *MAT*a/a diploid cells underwent at least one meiotic division when H3K56ac deposition was impaired (Figure 5E). These data demonstrate that Rtt109-mediated deposition of H3K56ac is critical for the activation of *IRT1* and prevention of inappropriate meiotic entry.

IRT2 transcription has a level-dependent effect on local chromatin state

We showed that low levels of *IRT2* transcription activate *IRT1* expression by directing H3K56ac to chromatin (Figures 2H, 2I, and 4C). How is H3K56ac incorporated into chromatin by *IRT2* transcription? Given that Rtt109-mediated H3K56ac occurs off chromatin, perhaps at low levels *IRT2* transcription mediates histone exchange to deliver H3K56ac to chromatin (Tsubota et al., 2007). Indeed, it is known that transcription can deliver free histones to nucleosomes in exchange for old ones (Das and Tyler, 2013; Jackson, 1990; Venkatesh and Workman, 2015). To examine whether *IRT2* promotes incorporation of new histones, we measured histone H3 exchange rates in the presence or absence of *IRT2* transcription. A strain harboring differentially epitope-tagged histone H3, with one copy expressed from the endogenous promoter and the other expressed from a *GAL1* inducible promoter, was used for the analysis (Figure 6A) (Schermer et al., 2005). Remarkably, the rate of incorporation of newly synthesized histone H3 significantly increased in the presence of constitutive levels of *IRT2* transcription (Figure 6B; compare WT [no *IRT2* transcription] to *u6bs*Δ [*IRT2* transcription]). Galactose induction had no effect on *IME1* expression under these conditions, which excludes the possibility that the effects were due to changes in *IME1* promoter activity (Figure 6C). The histone H3 exchange rates at two control promoters were not affected by *IRT2* transcription (Figures 6D and S6A). Importantly, elevated histone H3 exchange rate, as observed in the presence of constitutive levels of *IRT2* transcription, correlated with the enrichment of H3K56ac at *IRT2*. We propose that low levels of *IRT2* transcription stimulates histone exchange by a mechanism that is yet to be determined. Consequently, free histone H3, which is typically acetylated at lysine 56, is directed to chromatin. Subsequently, the presence of the H3K56ac mark facilitates chromatin

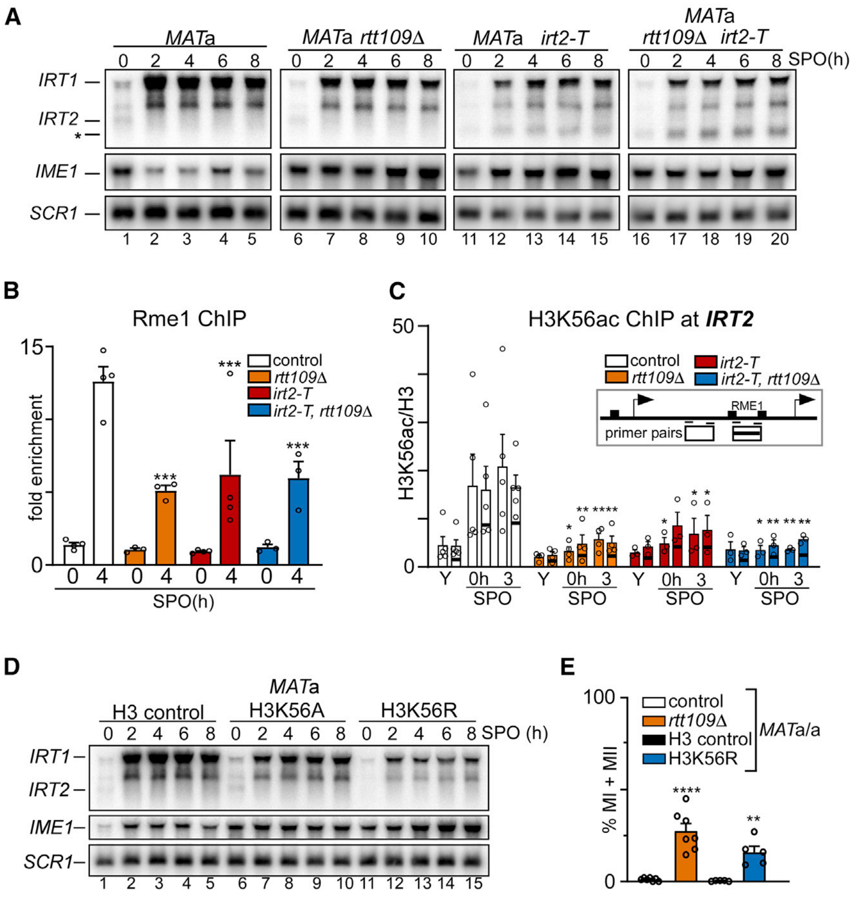

Figure 5. *IRT2* transcription directs histone H3 lysine 56 acetylation to chromatin locally to activate *IRT1* transcription

(A) Expression of *IRT1*, *IRT2*, and *IME1* in *MAT*a WT (FW1509), *rtt109*Δ (FW4077, lanes), *irt2-T* (FW3596), and *rtt109*Δ, *irt2-T* double-mutant (FW4072) cells.

(B) ChIP of Rme1-V5 at the *IRT1* promoter in strains described in (A) but harboring the *RME1-V5* allele (FW4031, FW3128, FW4075, and FW4073). Error bars represent ±SEM; n = 4, except for *rtt109*Δ and *irt2-T rtt109*Δ (n = 3). ***p < 0.0005, two-way ANOVA followed by Fisher's LSD test.

(C) Histone H3 lysine 56 acetylation (H3K56ac) levels in the *IRT1* promoter as measured by ChIP in control (*MAT*a, FW1509) and *irt2-T* (FW3596) cells. H3K56ac ChIP signals were normalized to histone H3. As control, *rtt109*Δ and *irt2-T rtt109*Δ cells (FW4077 and FW4072) were included. Error bars represent ±SEM; n = 5 for control, n = 4 for *rtt109*Δ, and n = 3 for *irt2-T* and *irt2-T rtt109*Δ. *p < 0.05; **p < 0.005, two-way ANOVA followed by Fisher's LSD test performed on each primer pair individually.

(D) *IRT1*, *IRT2*, and *IME1* expression in the *MAT*a histone H3 control (FW5102), H3K56A (FW5113), and H3K56R (FW5116) cells.

(E) *MAT*a/a diploid cells harboring *rtt109*Δ or H3K56R and matching controls (FW15, FW4557, FW7413, and FW7417). DAPI masses were counted from cells fixed at 72 h in SPO. Cells harboring >2 DAPI masses were considered to have undergone meiotic divisions (MI + MII). Error bars represent ±SEM; n = 7 for control and *rtt109*Δ, and n = 5 for H3 control and H3K56R. **p < 0.005; ****p < 0.0001, on an unpaired parametric two-tailed Student t test comparing mutant with respective control.

disassembly, recruitment of Rme1, and activation of *IRT1* transcription.

Previously, we showed that transcription of *IRT2* is important for repressing *IRT1* in *MAT*a/α diploid cells (Moretto et al., 2018). In *MAT*a/α diploid cells, *IRT2* levels were much higher than in *MAT*a haploid cells (Figure 1B). This raises the question of how *IRT2* transcription levels control opposing regulatory outcomes on *IRT1* transcription. In *MAT*a/α diploid cells, *IRT2* transcription increases nucleosome assembly around the Rme1 binding sites, which, consequently, interferes with Rme1 recruitment and activation of *IRT1* transcription (Moretto et al., 2018). In the wake of transcription, nucleosomes disassemble and re-assemble to maintain the chromatin structure (Venkatesh and Workman, 2015). With this view, increasing *IRT2* levels in *MAT*a haploid cells should elevate transcription-coupled chromatin assembly and, thereby, turn *IRT2* into a repressor of transcription. To test this, we modulated the level of *IRT2* transcription with different degrees in *MAT*a haploid cells. We integrated LexA operator (lexO) sequence repeats near the *IRT2* TSS ($lexO_n$(+96), +96 bp from the Ume6 binding site) and measured *IRT2* and *IRT1* levels together with nucleosome positioning (Figures 6E, 6F, and S6C–S6E). Upon activation by LexA-ER with β-estradiol, *IRT2* levels as well as Pol II binding increased with the number of integrated $lexO_n$(+96) repeats (Figures 6E and S6C–S6E). We found that the higher *IRT2* transcription was, the greater the repression of *IRT1* transcription was. As expected, nucleosome occupancy signal encompassing the Rme1 binding sites was reduced when *IRT1* was transcribed (SPO, 3 h) compared to when the locus was repressed (YPD) (Figures 6F and S6F). With increasing levels of *IRT2* transcription, the signals of the nucleosome around the Rme1 binding sites increased concomitantly (Figures 6F and S6F). Thus, with increasing *IRT2* transcription levels, the rate of chromatin assembly increases as well as the degree of *IRT1* transcription repression.

Taken together, these data indicate that low levels of *IRT2* transcription direct H3K56ac to chromatin, which, in turn, promotes the recruitment of Rme1 and activation of *IRT1* transcription. Conversely, increasing levels of *IRT2* transcription set in place a well-positioned nucleosome that likely interferes with Rme1 recruitment and, consequently, represses *IRT1* transcription (Moretto et al., 2018).

IRT2 transcription levels regulate the decision to enter meiosis

Our observation that *IRT2* transcription displays an opposing effect on chromatin state and *IRT1* expression prompted us to examine how increasing *IRT2* levels affect the fate of cells. Specifically, we determined the effect of increased *IRT2* transcription on meiosis in *MAT*a/a diploids (which behave like *MAT*a haploid cells) by replacing the endogenous promoter with the *CUP1* promoter (*pCUP-IRT2*) (Figure 6G). Under noninducing conditions (−Cu)

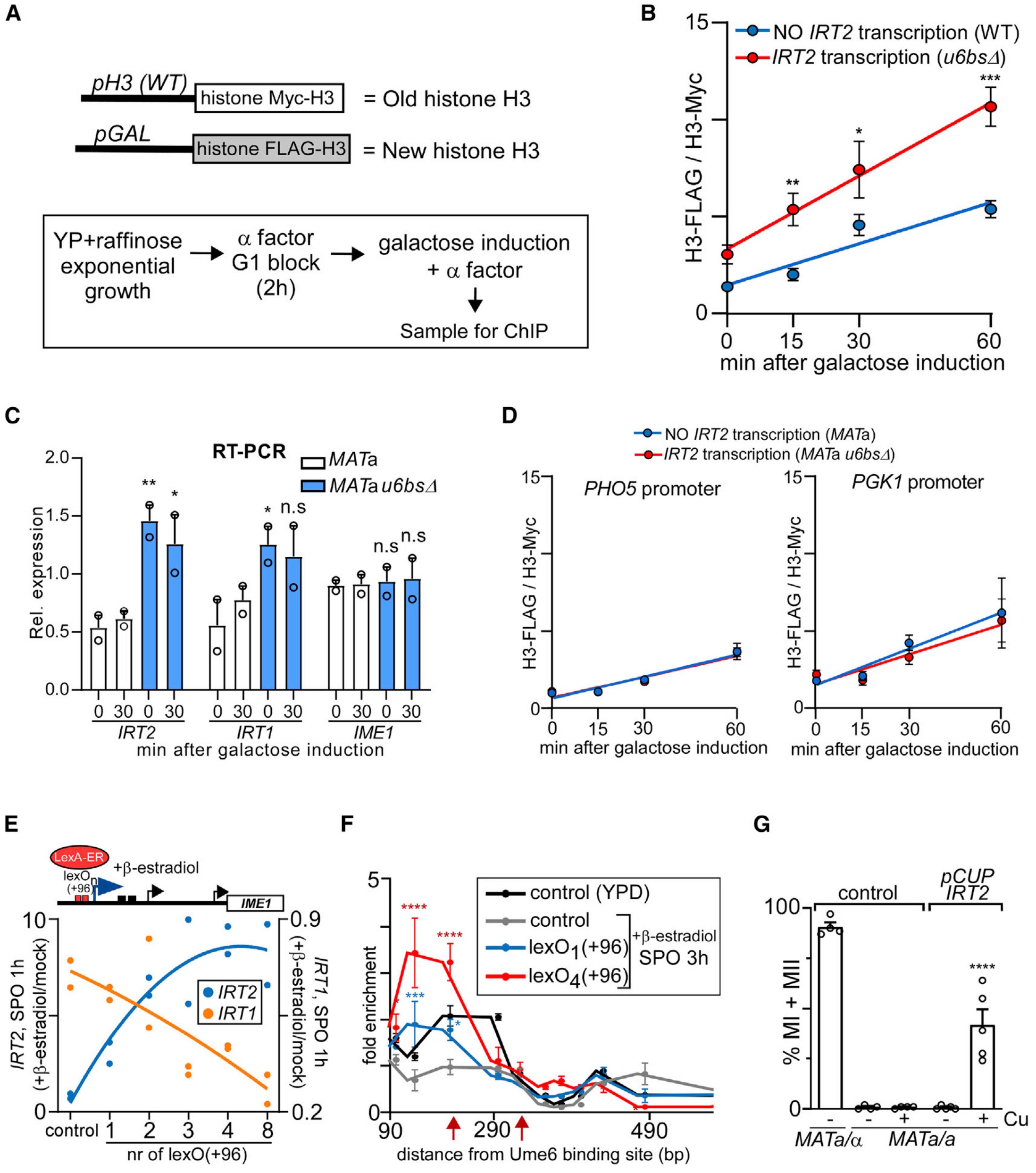

Figure 6. *IRT2* transcription has a level-dependent effect on local chromatin and transcription states

(A) Scheme for measuring histone H3 exchange rates.

(B) Histone H3 exchange rate at the *IRT1* promoter in the presence or absence of *IRT2* transcription. A strain harboring differentially epitope-tagged histone H3, with one copy expressed from the endogenous promoter (Myc-H3) and the other expressed from a *GAL1-10* inducible promoter (*pGAL-FLAG-H3*) were used for the analysis. Constitutive low levels of *IRT2* transcription are achieved using the *u6bs*Δ mutation (FW7880), whereas WT cells (FW7853) display no *IRT2* transcription in rich medium (YP). Cells were grown till mid-log in YP raffinose and arrested in G1 with α factor, and FLAG-H3 was induced with galactose. The signals for H3 ChIP (Myc-H3 and FLAG-H3) were normalized to a telomere locus, and ratios for n = 3 (error bars represent ±SEM) are displayed. *p < 0.05; **p < 0.005; and ***p < 0.0005, on a two-way ANOVA followed by Fisher's LSD test. The slopes of the linear regression equations (Y = [0.07105 · X] + 1.466 for control, and Y = [0.1258 · X] + 3.325 for *u6bs*Δ) are significantly different.

(C) Relative expression of *IRT2*, *IRT1*, and *IME1* in cells and grown as described in (B). qPCR signals were normalized to *ACT1*. Error bars represent ±SEM; n = 2. *p < 0.05; **p < 0.005, on an unpaired Student's t test.

(D) Similar to (B), histone exchange at the *PHO5* and *PGK1* control promoters in strains described in (B). Error bars represent ±SEM; n = 3. The slopes of the linear regression equations for both loci are not significantly different.

(E) Scheme for controlling different levels of *IRT2* by LexA-ER (top). Multiple lexA operator (lexO) sequences were integrated in the *IRT2* promoter at +96 bp relative to *IRT2* start site (lexO(+96)). LexA-ER is activated by β-estradiol. *IRT1* and *IRT2* levels as quantified by northern blot normalized to *SCR1*, in cells harboring 1, 2, 3, 4, and 8 lexO(+96) sites in the *IRT2* promoter (FW6594, FW6599, FW6607, FW6611, and FW6619) at 1 h in SPO. The *MATa* LexA-ER control

(legend continued on next page)

the *CUP1* promoter is not fully repressed, which explains that *MAT*a/a cells did not undergo meiotic divisions (Moretto et al., 2018). Approximately 50% of cells underwent meiosis when we induced *IRT2* transcription to high levels (+Cu). These data show that increasing *IRT2* transcription levels suppresses *IRT1*-mediated repression of the *IME1* promoter, allowing these cells to enter meiosis. Conversely, low levels of *IRT2* are required for activating *IRT1* transcription and preventing meiotic entry (Figure 3G).

Mathematic model of how *IRT2* transcription levels control meiotic entry

Our data demonstrate that low levels of *IRT2* transcription activates *IRT1* expression, whereas higher levels of *IRT2* transcription repress *IRT1* expression in a dose-dependent manner. *IRT1* transcription levels are also linked to the levels of Rme1, which vary greatly between cells expressing a single mating type (*MAT*a or *MAT*α) and cells expressing opposite mating types (*MAT*a/α), and between *MAT*a/α cells of different genetic backgrounds (Deutschbauer and Davis, 2005; Gerke et al., 2009; Mitchell and Herskowitz, 1986). To quantitatively dissect how the different signals of *IRT2* and Rme1 impinge on Ime1 expression, we developed a mathematical model describing the regulatory circuit consisting of *IRT2*, Rme1, *IRT1*, and *IME1* (Figures 7A and S7A).

To test the model, we simulated high and low Rme1 levels representing the single mating type (*MAT*a or *MAT*α, haploid) and diploid (*MAT*a/α) cell type, respectively, which resulted in the repression or activation of Ime1 expression (Figures 7B and 7C). In agreement with our experimental data, the model predicted that, in the absence of *IRT2*, activation of *IME1* transcription is independent of mating-type status. We then assessed the dose-response association between Rme1 and Ime1 (Figures 7D, S7B, and S7C). The analyses revealed a sigmoidal relationship, where over a wide range of high to low Rme1 levels, Ime1 expression was either repressed or activated. The two extremes of the curve, Ime1 expression or repression, represent the ability for diploid (*MAT*a/α) and the inability for haploid (*MAT*a or *MAT*α) cells to enter meiosis. Importantly, the absence of *IRT2* transcription or the presence of only activating low levels of *IRT2* abrogated the bimodal relationship between Rme1 and Ime1 expression levels (Figures 7D and S7C). The modeling further illustrates the importance of the dual function for *IRT2* transcription in controlling the timely expression of Ime1 in a cell-type-specific manner and thereby regulating the decision to enter meiosis in yeast.

DISCUSSION

We showed how transcription levels of an lncRNA have a critical role in regulating gene expression and cell fate outcomes. Specifically, we demonstrated that opposing transcription levels of the lncRNA *IRT2* ensure a robust transition from nutrient to mating-type control of *IME1* promoter activity. This dual role for *IRT2* transcription is an essential component of the regulatory circuit enabling meiosis in yeast cells.

Mechanism of *IRT2*-mediated activation of transcription

Two lncRNAs, *IRT1* and *IRT2*, are transcribed through different parts of the *IME1* promoter to control *IME1* expression and the decision to enter meiosis (Moretto et al., 2018; van Werven et al., 2012). Several lines of evidence indicate that *IRT2* transcription directly promotes *IRT1* transcription in single-mating-type cells. First, partial deletions in *IRT2* compromise Rme1 recruitment and activation of *IRT1* transcription. Second, insertion of a transcriptional terminator between the Rme1 binding sites and the *IRT2* TSS affects *IRT1* activation, suggesting that the act of transcription is required. Third, altered *IRT2* transcription patterns due to mutations in Ime1 or the Ume6 binding sites upstream of *IRT2* affected *IRT1* expression in a comparable way. Finally, low levels of *IRT2* transcription controlled from a heterologous promoter directly upstream of *IRT2* were sufficient to induce *IRT1* transcription.

How does *IRT2* transcription promote *IRT1* activation? We identified Rtt109 as a regulator of *IRT1* transcription. Cells with a single mating type can enter meiosis when *RTT109* is deleted or when H3K56ac, the main substrate of Rtt109, is directly disrupted. We propose that low levels of *IRT2* transcription stimulate H3K56ac incorporation into nucleosomes locally. The H3K56ac mark, in turn, facilitates Rme1 recruitment and activation of *IRT1* transcription. Our results are consistent with a model describing that H3K56ac increases nucleosome unwrapping, facilitating TF binding and transcription activation (Bernier et al., 2015; Neumann et al., 2009; Williams et al., 2008).

We show that *IRT2* transcription stimulates histone exchange, thereby directing free histone H3K56ac to chromatin. The underlying mechanism, however, remains to be determined. One possibility is that, during Pol II transcription, chaperones facilitate the incorporation of free histones into nucleosomes (Park and Luger, 2008). During DNA replication in yeast, free histones and, thus, H3K56ac are directed to nucleosomes by chromatin assembly factors (Kaplan et al., 2008; Topal et al., 2019). Perhaps these factors also play a role during *IRT2* transcription. Another possibility is that, in the wake of transcription, partial disassembly of nucleosomes leads to stochastic exchange of histones (Jamai et al., 2007). In line with both ideas, H3K56ac is enriched in transcribed gene bodies in the *set2* deletion mutant—thus, in the absence of H3K36 methylation—suggesting that transcription promotes histone exchange (Venkatesh et al., 2012). It is

strain (control, FW6560) was included. Cells were treated (+β-estradiol) or not (mock) for 3 h and shifted to SPO plus β-estradiol. The ratio of +β-estradiol/mock is displayed. n = 2 data points and a trend line representing second-degree polynomial fit are shown.

(F) Chromatin structure at the *IRT1* promoter in the presence of distinct levels of *IRT2* transcription. Control cells (*MAT*a LexA-ER, FW6560) or cells harboring 1 or 4 lexO(+96) sites (FW6594 or FW6611) were treated as described in (E). MNase-digested fragments were subject to qPCRs using primer pairs nested in *IRT2*. The red arrows indicate the position of the Rme1 binding sites. The signals were normalized over a telomere locus. Error bars represent ±SEM; n = 3. *$p < 0.05$; ***$p < 0.0005$; and ****$p < 0.0001$, on a two-way ANOVA followed by Fisher's LSD test performed on lexO strains compared to control SPO for 3 h.

(G) *MAT*a/α and *MAT*a/a diploid cells (FW1511 and FW15) and *MAT*a/a cells harboring *pCUP-IRT2* (FW8923) were shifted to SPO and either treated (+Cu) or not (−Cu) with copper sulfate. DAPI masses were counted from cells fixed at 72 h in SPO. Cells harboring >2 DAPI masses were considered to have undergone meiotic divisions (MI + MII). Error bars represent ±SEM; n = 4 for the controls, and n = 5 for *pCUP-IRT2*. ****$p < 0.0001$, two-way ANOVA followed by Fisher's LSD test performed on *MAT*a/a strains with or without copper sulfate treatment.

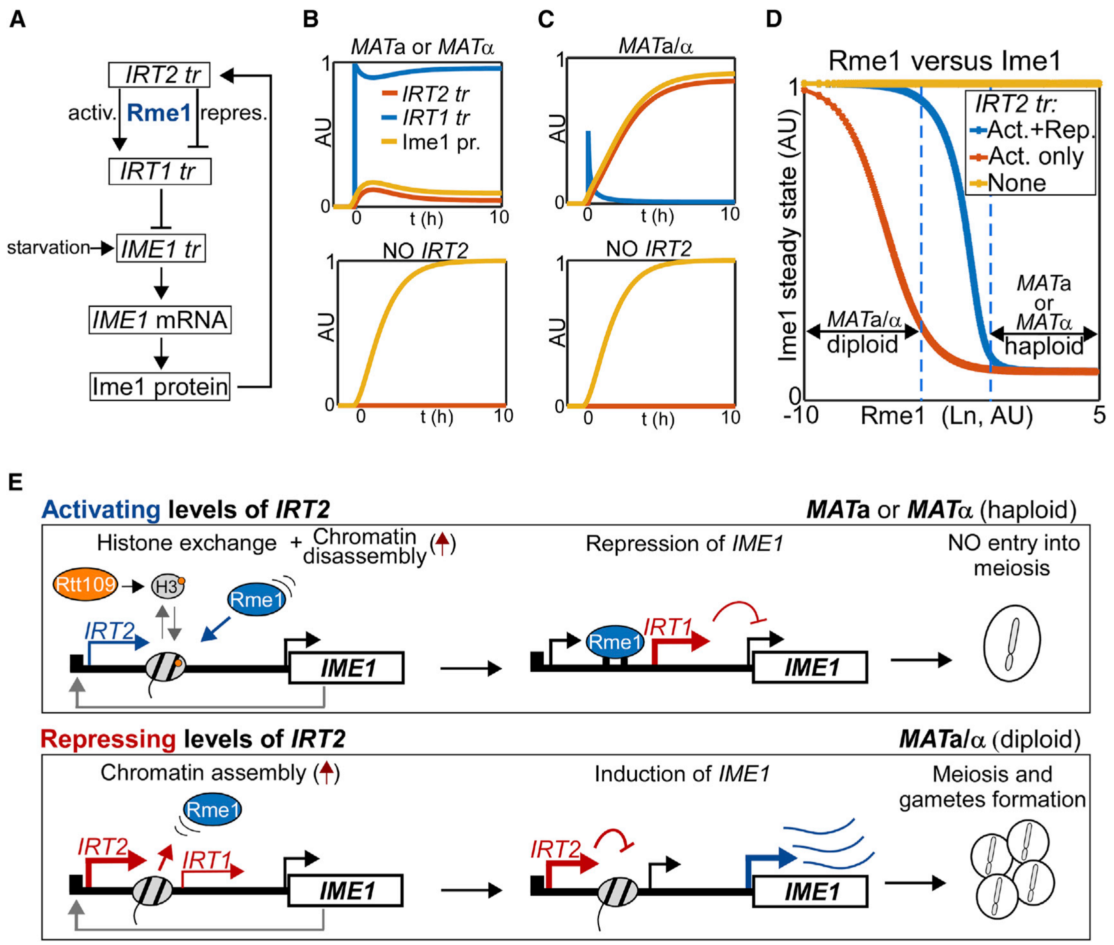

Figure 7. Modeling of cell-type-specific control of Ime1 expression involving noncoding transcription

(A) Scheme of the mathematical model.

(B and C) Simulation of *MAT*a and *MAT*a/α cells in the presence or absence of *IRT2*.

(D) Simulation of Ime1 steady-state protein levels over a range of Rme1 concentrations (Ln), in the presence of either WT levels of *IRT2* (activating and repressive effects on *IRT1*), activating levels of *IRT2* (activating effect on *IRT1* only), or no *IRT2*.

(E) Model describing how distinct levels of *IRT2* control the decision of whether to enter meiosis and form gametes in haploid (*MAT*a or *MAT*α) and diploid (*MAT*a/α) cells.

important to note that free H3K56ac histones are unlikely to promote histone exchange themselves during transcription (Ferrari and Strubin, 2015). Finally, H3K56ac is widespread at promoter proximal nucleosomes where divergent noncoding transcription takes place, which raises the interesting possibility that H3K56ac incorporation via noncoding transcription may be widespread (Topal et al., 2019; Xu et al., 2009).

The transcription-controlled histone exchange and incorporation of acetylated histones into chromatin, as we described here, could be reminiscent of how a class of lncRNAs, called eRNAs, regulate gene expression (Li et al., 2016). Like *IRT2* transcription, transcription through enhancers can facilitate recruitment of TFs and correlates with certain histone marks, including histone acetylation. Interestingly, in mammalian cells, H3K56ac is enriched at active enhancers and promoters, perhaps suggesting that noncoding transcription-directed H3K56ac deposition could be conserved (Skalska et al., 2015; Tan et al., 2013).

Model for the dual function of transcription of an lncRNA

Previously, we described the regulatory circuit consisting of *IRT2*, *IRT1*, and *IME1* (Moretto et al., 2018). We showed that *IRT2* transcription interferes with *IRT1* activation, which, in turn, leads to the upregulation of *IME1* expression and entry into meiosis in *MAT*a/α cells. Here, we demonstrated that the *IRT2* effect on gene regulation follows a hormetic pattern. In the absence of *IRT2* transcription, no activation of *IRT1* transcription will occur. However, relatively low levels of *IRT2* will promote *IRT1* activation, whereas increasing *IRT2* transcription will repress *IRT1* (Figure 7E).

How do transcription levels of *IRT2* determine whether to activate or repress gene expression? Our data suggest that there is a dynamic interplay between nucleosome assembly, disassembly, and TF concentration. In the wake of transcription, nucleosomes disassemble and re-assemble to maintain chromatin structure (Ard et al., 2017; Venkatesh and Workman, 2015). With this

view, as the transcription levels of *IRT2* increases, the rate of transcription-coupled nucleosome assembly also increases, eventually leading to repression of *IRT1*. In activation context, *IRT2* transcription directs histone H3K56ac to nucleosomes, which, in turn, facilitates nucleosome disassembly (Kaplan et al., 2008; Rufiange et al., 2007). In principle, one round of transcription can direct histone exchange and, thus, nucleosome disassembly. When the rate of transcription-coupled nucleosome assembly is higher than the rate of nucleosome disassembly, it "tips the scale" toward repression of *IRT1* (Figure 7E). As depicted in our mathematical model, the concentration of Rme1 also plays an important role in activation of *IRT1* transcription. The higher Rme1 levels are, the earlier a stable association to its binding site will occur and, thus, activate *IRT1* transcription. Our model further shows that the dual function of *IRT2* and Rme1 concentration in the cell form a circuit to regulate mating-type signaling to Ime1 expression. Taken together, our findings in this study of the *IME1* promoter demonstrate that noncoding transcription levels play a determining role on whether repression or activation of gene transcription will occur.

Many examples of gene regulation by lncRNA transcription with different outcomes have been reported (Engreitz et al., 2016; Gil and Ulitsky, 2020; Hirota et al., 2008; Kornienko et al., 2013; Martens et al., 2004; van Werven et al., 2012). Our finding of distinct transcription levels of a noncoding RNA directly opposing chromatin and transcription states illustrates the gene regulatory potential for noncoding transcription events in general. Given that lncRNAs are transcribed across many parts of the genome, from yeast to humans, we propose that the act of noncoding transcription itself through promoters or other regulatory regions may have extensive functions in regulating gene expression (David et al., 2006; Hon et al., 2017; Iyer et al., 2015; Kung et al., 2013).

STAR★METHODS

Detailed methods are provided in the online version of this paper and include the following:

- KEY RESOURCES TABLE
- RESOURCE AVAILABILITY
  - ○ Lead contact
  - ○ Materials availability
  - ○ Data and code availability
- EXPERIMENTAL MODEL AND SUBJECT DETAILS
  - ○ Yeast strains
  - ○ Growth and conditions
- METHOD DETAILS
  - ○ Plasmids and yeasts transformation
  - ○ Oligonucleotides Used in This Study
  - ○ Nuclei/DAPI counting
  - ○ Chromatin immunoprecipitation
  - ○ Micrococcal nuclease (MNase) qPCR
  - ○ Northern blotting
  - ○ RNA-seq
  - ○ TSS-seq and TES-seq
  - ○ Nascent RNA-Seq
  - ○ Bioinformatic analysis
  - ○ Single molecule RNA fluorescent *in situ* hybridization (FISH)
  - ○ Western blotting
  - ○ Spot growth assay
  - ○ Mathematical modeling
  - ○ List of variables (scaled between 0 and 1)
  - ○ List of parameters used for simulation
  - ○ Equations
- QUANTIFICATION AND STATISTICAL ANALYSIS

SUPPLEMENTAL INFORMATION

Supplemental Information can be found online at https://doi.org/10.1016/j.celrep.2020.108643.

ACKNOWLEDGMENTS

We are grateful to Frank Uhlmann, Jesper Svejstrup, and members of the van Werven lab for advice and critical reading of the manuscript. We thank Elçin Ünal, Philipp Korber, Valerie Borde, and Celine Bouchoux for sharing reagents. N.E.W. is supported by the Cancer Prevention and Research Institute of Texas (RR150058, which was obtained by Andreas Doncic). F.M. and F.J.v.W. are supported by the Francis Crick Institute (FC001203), which receives its core funding from Cancer Research UK (FC001203), the UK Medical Research Council (FC001203), and the Wellcome Trust (FC001203). C.L. and N.M.L. are supported by the Francis Crick Institute (FC010110), which receives its core funding from Cancer Research UK (FC010110), the UK Medical Research Council (FC01011), and the Wellcome Trust (FC010110). M.C. is supported by a fellowship from the Agency for Science, Technology and Research (A*STAR) of Singapore.

AUTHOR CONTRIBUTIONS

F.M. and F.J.v.W. conceived and designed the study. F.M. and F.J.v.W. designed the experiments. F.M. performed the experiments. F.M. and F.J.v.W. analyzed the data. N.E.W. generated the mathematical model. M.C., C.L., and N.M.L. helped with generating and analyzing sequencing data. F.M. and F.J.v.W. wrote the manuscript, with input from all authors. F.J.v.W. supervised the project.

DECLARATION OF INTERESTS

The authors declare no competing interests.

Received: June 1, 2020
Revised: October 28, 2020
Accepted: December 22, 2020
Published: January 19, 2021

REFERENCES

Adjalley, S.H., Chabbert, C.D., Klaus, B., Pelechano, V., and Steinmetz, L.M. (2016). Landscape and Dynamics of Transcription Initiation in the Malaria Parasite Plasmodium falciparum. Cell Rep *14*, 2463–2475.

Adkins, M.W., Carson, J.J., English, C.M., Ramey, C.J., and Tyler, J.K. (2007). The histone chaperone anti-silencing function 1 stimulates the acetylation of newly synthesized histone H3 in S-phase. J. Biol. Chem. *282*, 1334–1340.

Ard, R., and Allshire, R.C. (2016). Transcription-coupled changes to chromatin underpin gene silencing by transcriptional interference. Nucleic Acids Res. *44*, 10619–10630.

Ard, R., Tong, P., and Allshire, R.C. (2014). Long non-coding RNA-mediated transcriptional interference of a permease gene confers drug tolerance in fission yeast. Nat. Commun. *5*, 5576.

CellPress
OPEN ACCESS

Ard, R., Allshire, R.C., and Marquardt, S. (2017). Emerging Properties and Functional Consequences of Noncoding Transcription. Genetics *207*, 357–367.

Arribere, J.A., and Gilbert, W.V. (2013). Roles for transcript leaders in translation and mRNA decay revealed by transcript leader sequencing. Genome Res *23*, 977–987.

Bernier, M., Luo, Y., Nwokelo, K.C., Goodwin, M., Dreher, S.J., Zhang, P., Parthun, M.R., Fondufe-Mittendorf, Y., Ottesen, J.J., and Poirier, M.G. (2015). Linker histone H1 and H3K56 acetylation are antagonistic regulators of nucleosome dynamics. Nat. Commun. *6*, 10152.

Bumgarner, S.L., Dowell, R.D., Grisafi, P., Gifford, D.K., and Fink, G.R. (2009). Toggle involving cis-interfering noncoding RNAs controls variegated gene expression in yeast. Proc. Natl. Acad. Sci. USA *106*, 18321–18326.

Carlile, T.M., and Amon, A. (2008). Meiosis I is established through division-specific translational control of a cyclin. Cell *133*, 280–291.

Chia, M., Tresenrider, A., Chen, J., Spedale, G., Jorgensen, V., Unal, E., and van Werven, F.J. (2017). Transcription of a 5' extended mRNA isoform directs dynamic chromatin changes and interference of a downstream promoter. Elife *6*.

Das, C., and Tyler, J.K. (2013). Histone exchange and histone modifications during transcription and aging. Biochim. Biophys. Acta *1819*, 332–342.

David, L., Huber, W., Granovskaia, M., Toedling, J., Palm, C.J., Bofkin, L., Jones, T., Davis, R.W., and Steinmetz, L.M. (2006). A high-resolution map of transcription in the yeast genome. Proc. Natl. Acad. Sci. USA *103*, 5320–5325.

Deutschbauer, A.M., and Davis, R.W. (2005). Quantitative trait loci mapped to single-nucleotide resolution in yeast. Nat. Genet. *37*, 1333–1340.

Dobin, A., Davis, C.A., Schlesinger, F., Drenkow, J., Zaleski, C., Jha, S., Batut, P., Chaisson, M., and Gingeras, T.R. (2013). STAR: ultrafast universal RNA-seq aligner. Bioinformatics *29*, 15–21.

Driscoll, R., Hudson, A., and Jackson, S.P. (2007). Yeast Rtt109 promotes genome stability by acetylating histone H3 on lysine 56. Science *315*, 649–652.

Engreitz, J.M., Haines, J.E., Perez, E.M., Munson, G., Chen, J., Kane, M., McDonel, P.E., Guttman, M., and Lander, E.S. (2016). Local regulation of gene expression by lncRNA promoters, transcription and splicing. Nature *539*, 452–455.

Ferrari, P., and Strubin, M. (2015). Uncoupling histone turnover from transcription-associated histone H3 modifications. Nucleic Acids Res. *43*, 3972–3985.

Gerke, J., Lorenz, K., and Cohen, B. (2009). Genetic interactions between transcription factors cause natural variation in yeast. Science *323*, 498–501.

Gil, N., and Ulitsky, I. (2020). Regulation of gene expression by cis-acting long non-coding RNAs. Nat. Rev. Genet. *21*, 102–117.

Guttman, M., Donaghey, J., Carey, B.W., Garber, M., Grenier, J.K., Munson, G., Young, G., Lucas, A.B., Ach, R., Bruhn, L., et al. (2011). lincRNAs act in the circuitry controlling pluripotency and differentiation. Nature *477*, 295–300.

Hainer, S.J., Pruneski, J.A., Mitchell, R.D., Monteverde, R.M., and Martens, J.A. (2011). Intergenic transcription causes repression by directing nucleosome assembly. Genes Dev. *25*, 29–40.

Han, J., Zhou, H., Horazdovsky, B., Zhang, K., Xu, R.M., and Zhang, Z. (2007). Rtt109 acetylates histone H3 lysine 56 and functions in DNA replication. Science *315*, 653–655.

Hirota, K., Miyoshi, T., Kugou, K., Hoffman, C.S., Shibata, T., and Ohta, K. (2008). Stepwise chromatin remodelling by a cascade of transcription initiation of non-coding RNAs. Nature *456*, 130–134.

Hon, C.C., Ramilowski, J.A., Harshbarger, J., Bertin, N., Rackham, O.J., Gough, J., Denisenko, E., Schmeier, S., Poulsen, T.M., Severin, J., et al. (2017). An atlas of human long non-coding RNAs with accurate 5′ ends. Nature *543*, 199–204.

Iyer, M.K., Niknafs, Y.S., Malik, R., Singhal, U., Sahu, A., Hosono, Y., Barrette, T.R., Prensner, J.R., Evans, J.R., Zhao, S., et al. (2015). The landscape of long noncoding RNAs in the human transcriptome. Nat. Genet. *47*, 199–208.

Jackson, V. (1990). In vivo studies on the dynamics of histone-DNA interaction: evidence for nucleosome dissolution during replication and transcription and a low level of dissolution independent of both. Biochemistry *29*, 719–731.

Jamai, A., Imoberdorf, R.M., and Strubin, M. (2007). Continuous histone H2B and transcription-dependent histone H3 exchange in yeast cells outside of replication. Mol. Cell *25*, 345–355.

Kahana, S., Pnueli, L., Kainth, P., Sassi, H.E., Andrews, B., and Kassir, Y. (2010). Functional dissection of IME1 transcription using quantitative promoter-reporter screening. Genetics *186*, 829–841.

Kaplan, T., Liu, C.L., Erkmann, J.A., Holik, J., Grunstein, M., Kaufman, P.D., Friedman, N., and Rando, O.J. (2008). Cell cycle- and chaperone-mediated regulation of H3K56ac incorporation in yeast. PLoS Genet. *4*, e1000270.

Kassir, Y., Granot, D., and Simchen, G. (1988). IME1, a positive regulator gene of meiosis in S. cerevisiae. Cell *52*, 853–862.

Kent, W.J., Zweig, A.S., Barber, G., Hinrichs, A.S., and Karolchik, D. (2010). BigWig and BigBed: enabling browsing of large distributed datasets. Bioinformatics *26*, 2204–2207.

Kim, T., Xu, Z., Clauder-Münster, S., Steinmetz, L.M., and Buratowski, S. (2012). Set3 HDAC mediates effects of overlapping noncoding transcription on gene induction kinetics. Cell *150*, 1158–1169.

Kornienko, A.E., Guenzl, P.M., Barlow, D.P., and Pauler, F.M. (2013). Gene regulation by the act of long non-coding RNA transcription. BMC Biol. *11*, 59.

Kung, J.T., Colognori, D., and Lee, J.T. (2013). Long noncoding RNAs: past, present, and future. Genetics *193*, 651–669.

Lai, D.P., Tan, S., Kang, Y.N., Wu, J., Ooi, H.S., Chen, J., Shen, T.T., Qi, Y., Zhang, X., et al. (2015). Genome-wide profiling of polyadenylation sites reveals a link between selective polyadenylation and cancer metastasis. Hum Mol Genet *24*, 3410–3417.

Latos, P.A., Pauler, F.M., Koerner, M.V., Şenergin, H.B., Hudson, Q.J., Stocsits, R.R., Allhoff, W., Stricker, S.H., Klement, R.M., Warczok, K.E., et al. (2012). Airn transcriptional overlap, but not its lncRNA products, induces imprinted Igf2r silencing. Science *338*, 1469–1472.

Li, H., Handsaker, B., Wysoker, A., Fennell, T., Ruan, J., Homer, N., Marth, G., Abecasis, G., and Durbin, R.; 10000 Genomes Project Data Processing Subgroup (2009). The Sequence Alignment/Map format and SAMtools. Bioinformatics *25*, 2078–2079.

Li, W., Notani, D., and Rosenfeld, M.G. (2016). Enhancers as non-coding RNA transcription units: recent insights and future perspectives. Nat. Rev. Genet. *17*, 207–223.

Lino, Y., and Yamamoto, M. (1985). Mutants of Schizosaccharomyces pombe which sporulate in the haploid state. Mol. Gen. Genet. *198*, 416–421.

Longtine, M.S., McKenzie, A., 3rd, Demarini, D.J., Shah, N.G., Wach, A., Brachat, A., Philippsen, P., and Pringle, J.R. (1998). Additional modules for versatile and economical PCR-based gene deletion and modification in Saccharomyces cerevisiae. Yeast *14*, 953–961.

Malabat, C., Feuerbach, F., Ma, L., Saveanu, C., and Jacquier, A. (2015). Quality control of transcription start site selection by nonsense-mediated-mRNA decay. Elife *4*.

Martens, J.A., Laprade, L., and Winston, F. (2004). Intergenic transcription is required to repress the Saccharomyces cerevisiae SER3 gene. Nature *429*, 571–574.

Martin, Marcel (2011). Cutadapt removes adapter sequences from high-throughput sequencing reads. EMBnet.journal *17*, 10–12.

Masumoto, H., Hawke, D., Kobayashi, R., and Verreault, A. (2005). A role for cell-cycle-regulated histone H3 lysine 56 acetylation in the DNA damage response. Nature *436*, 294–298.

Mitchell, A.P., and Herskowitz, I. (1986). Activation of meiosis and sporulation by repression of the RME1 product in yeast. Nature *319*, 738–742.

Moretto, F., and van Werven, F.J. (2017). Transcription of the mating-type-regulated lncRNA IRT1 is governed by TORC1 and PKA. Curr. Genet. *63*, 325–329.

Moretto, F., Wood, N.E., Kelly, G., Doncic, A., and van Werven, F.J. (2018). A regulatory circuit of two lncRNAs and a master regulator directs cell fate in yeast. Nat. Commun. *9*, 780.

Nachman, I., Regev, A., and Ramanathan, S. (2007). Dissecting timing variability in yeast meiosis. Cell *131*, 544–556.

Neil, H., Malabat, C., d'Aubenton-Carafa, Y., Xu, Z., Steinmetz, L.M., and Jacquier, A. (2009). Widespread bidirectional promoters are the major source of cryptic transcripts in yeast. Nature *457*, 1038–1042.

Neumann, H., Hancock, S.M., Buning, R., Routh, A., Chapman, L., Somers, J., Owen-Hughes, T., van Noort, J., Rhodes, D., and Chin, J.W. (2009). A method for genetically installing site-specific acetylation in recombinant histones defines the effects of H3 K56 acetylation. Mol. Cell *36*, 153–163.

Ng, P., Wei, C.L., Sung, W.K., Chiu, K.P., Lipovich, L., Ang, C.C., Gupta, S., Shahab, A., Ridwan, A., et al. (2005). Gene identification signature (GIS) analysis for transcriptome characterization and genome annotation. Nat Methods *2*, 105–111.

Ottoz, D.S., Rudolf, F., and Stelling, J. (2014). Inducible, tightly regulated and growth condition-independent transcription factor in Saccharomyces cerevisiae. Nucleic Acids Res. *42*, e130.

Park, Y.J., and Luger, K. (2008). Histone chaperones in nucleosome eviction and histone exchange. Curr. Opin. Struct. Biol. *18*, 282–289.

Pelechano, V., and Steinmetz, L.M. (2013). Gene regulation by antisense transcription. Nat. Rev. Genet. *14*, 880–893.

Ponting, C.P., Oliver, P.L., and Reik, W. (2009). Evolution and functions of long noncoding RNAs. Cell *136*, 629–641.

Primig, M., Williams, R.M., Winzeler, E.A., Tevzadze, G.G., Conway, A.R., Hwang, S.Y., Davis, R.W., and Esposito, R.E. (2000). The core meiotic transcriptome in budding yeasts. Nat. Genet. *26*, 415–423.

Quinlan, A.R., and Hall, I.M. (2010). BEDTools: a flexible suite of utilities for comparing genomic features. Bioinformatics *26*, 841–842.

Rando, O.J. (2010). Genome-wide mapping of nucleosomes in yeast. Methods Enzymol *470*, 105–118.

Recht, J., Tsubota, T., Tanny, J.C., Diaz, R.L., Berger, J.M., Zhang, X., Garcia, B.A., Shabanowitz, J., Burlingame, A.L., Hunt, D.F., et al. (2006). Histone chaperone Asf1 is required for histone H3 lysine 56 acetylation, a modification associated with S phase in mitosis and meiosis. Proc. Natl. Acad. Sci. USA *103*, 6988–6993.

Rom, A., Melamed, L., Gil, N., Goldrich, M.J., Kadir, R., Golan, M., Biton, I., Perry, R.B., and Ulitsky, I. (2019). Regulation of CHD2 expression by the Chaserr long noncoding RNA gene is essential for viability. Nat. Commun. *10*, 5092.

Rufiange, A., Jacques, P.E., Bhat, W., Robert, F., and Nourani, A. (2007). Genome-wide replication-independent histone H3 exchange occurs predominantly at promoters and implicates H3 K56 acetylation and Asf1. Mol. Cell *27*, 393–405.

Sagee, S., Sherman, A., Shenhar, G., Robzyk, K., Ben-Doy, N., Simchen, G., and Kassir, Y. (1998). Multiple and distinct activation and repression sequences mediate the regulated transcription of IME1, a transcriptional activator of meiosis-specific genes in Saccharomyces cerevisiae. Mol. Cell. Biol. *18*, 1985–1995.

Schermer, U.J., Korber, P., and Hörz, W. (2005). Histones are incorporated in trans during reassembly of the yeast PHO5 promoter. Mol. Cell *19*, 279–285.

Schneider, J., Bajwa, P., Johnson, F.C., Bhaumik, S.R., and Shilatifard, A. (2006). Rtt109 is required for proper H3K56 acetylation: a chromatin mark associated with the elongating RNA polymerase II. J. Biol. Chem. *281*, 37270–37274.

Skalska, L., Stojnic, R., Li, J., Fischer, B., Cerda-Moya, G., Sakai, H., Tajbakhsh, S., Russell, S., Adryan, B., and Bray, S.J. (2015). Chromatin signatures at Notch-regulated enhancers reveal large-scale changes in H3K56ac upon activation. EMBO J. *34*, 1889–1904.

Sommermeyer, V., Beneut, C., Chaplais, E., Serrentino, M.E., and Borde, V. (2013). Spp1, a member of the Set1 Complex, promotes meiotic DSB formation in promoters by tethering histone H3K4 methylation sites to chromosome axes. Mol Cell *49*, 43–54.

Storici, F., Lewis, L.K., and Resnick, M.A. (2001). In vivo site-directed mutagenesis using oligonucleotides. Nature Biotechnology *19*, 773.

Takemata, N., Oda, A., Yamada, T., Galipon, J., Miyoshi, T., Suzuki, Y., Sugano, S., Hoffman, C.S., Hirota, K., and Ohta, K. (2016). Local potentiation of stress-responsive genes by upstream noncoding transcription. Nucleic Acids Res. *44*, 5174–5189.

Tam, J., and van Werven, F.J. (2020). Regulated repression governs the cell fate promoter controlling yeast meiosis. Nat. Commun. *11*, 2271.

Tan, Y., Xue, Y., Song, C., and Grunstein, M. (2013). Acetylated histone H3K56 interacts with Oct4 to promote mouse embryonic stem cell pluripotency. Proc. Natl. Acad. Sci. USA *110*, 11493–11498.

Terweij, M., van Welsem, T., van Deventer, S., Verzijlbergen, K.F., Menendez-Benito, V., Ontoso, D., San-Segundo, P., Neefjes, J., and van Leeuwen, F. (2013). Recombination-induced tag exchange (RITE) cassette series to monitor protein dynamics in Saccharomyces cerevisiae. G3 (Bethesda) *3*, 1261–1272.

Tisseur, M., Kwapisz, M., and Morillon, A. (2011). Pervasive transcription - Lessons from yeast. Biochimie *93*, 1889–1896.

Topal, S., Vasseur, P., Radman-Livaja, M., and Peterson, C.L. (2019). Distinct transcriptional roles for Histone H3-K56 acetylation during the cell cycle in Yeast. Nat. Commun. *10*, 4372.

Tsubota, T., Berndsen, C.E., Erkmann, J.A., Smith, C.L., Yang, L., Freitas, M.A., Denu, J.M., and Kaufman, P.D. (2007). Histone H3-K56 acetylation is catalyzed by histone chaperone-dependent complexes. Mol. Cell *25*, 703–712.

van Werven, F.J., and Amon, A. (2011). Regulation of entry into gametogenesis. Philos. Trans. R. Soc. Lond. B Biol. Sci. *366*, 3521–3531.

van Werven, F.J., Neuert, G., Hendrick, N., Lardenois, A., Buratowski, S., van Oudenaarden, A., Primig, M., and Amon, A. (2012). Transcription of two long noncoding RNAs mediates mating-type control of gametogenesis in budding yeast. Cell *150*, 1170–1181.

van Werven, F.J., and Timmers, H.T.M. (2006). The use of biotin tagging in Saccharomyces cerevisiae improves the sensitivity of chromatin immunoprecipitation. Nucleic Acids Research *34*, 33–33.

Värv, S., Kristjuhan, K., Peil, K., Lõoke, M., Mahlakõiv, T., Paapsi, K., and Kristjuhan, A. (2010). Acetylation of H3 K56 is required for RNA polymerase II transcript elongation through heterochromatin in yeast. Mol. Cell. Biol. *30*, 1467–1477.

Venkatesh, S., and Workman, J.L. (2015). Histone exchange, chromatin structure and the regulation of transcription. Nat. Rev. Mol. Cell Biol. *16*, 178–189.

Venkatesh, S., Smolle, M., Li, H., Gogol, M.M., Saint, M., Kumar, S., Natarajan, K., and Workman, J.L. (2012). Set2 methylation of histone H3 lysine 36 suppresses histone exchange on transcribed genes. Nature *489*, 452–455.

Voichek, Y., Bar-Ziv, R., and Barkai, N. (2016). Expression homeostasis during DNA replication. Science *351*, 1087–1090.

Wagstaff, J.E., Klapholz, S., and Esposito, R.E. (1982). Meiosis in haploid yeast. Proc. Natl. Acad. Sci. USA *79*, 2986–2990.

Wang, K.C., and Chang, H.Y. (2011). Molecular mechanisms of long noncoding RNAs. Mol. Cell *43*, 904–914.

Wapinski, O., and Chang, H.Y. (2011). Long noncoding RNAs and human disease. Trends Cell Biol. *21*, 354–361.

Williams, S.K., Truong, D., and Tyler, J.K. (2008). Acetylation in the globular core of histone H3 on lysine-56 promotes chromatin disassembly during transcriptional activation. Proc. Natl. Acad. Sci. USA *105*, 9000–9005.

Xu, F., Zhang, K., and Grunstein, M. (2005). Acetylation in histone H3 globular domain regulates gene expression in yeast. Cell *121*, 375–385.

Xu, Z., Wei, W., Gagneur, J., Perocchi, F., Clauder-Münster, S., Camblong, J., Guffanti, E., Stutz, F., Huber, W., and Steinmetz, L.M. (2009). Bidirectional promoters generate pervasive transcription in yeast. Nature *457*, 1033–1037.

CellPress
OPEN ACCESS

STAR★METHODS

KEY RESOURCES TABLE

REAGENT or RESOURCE	SOURCE	IDENTIFIER
Antibodies		
Anti-V5 tag (mouse) antibody	Thermo Fisher Scientific	R96025; RRID: AB_2556564
Anti-hexokinase (rabbit) antibody	US Biological	H2035; RRID: AB_2629457
Anti-Myc tag (mouse) antibody	Merck Millipore	05-724; RRID: AB_309938
Anti-FLAG tag (mouse) antibody	Sigma-Aldrich (Merck)	F3165; RRID: AB_259529
Anti-V5 agarose affinity gel	Sigma-Aldrich (Merck)	A7345; RRID: AB_10062721
Anti-Mouse IgG IRDye® 800CW (goat)	LICOR (NE USA)	925-32210; RRID: AB_2687825
Anti-rabbit IgG IRDye 680RD (donkey)	LICOR (NE USA)	925-68073; RRID: AB_2716687
Anti-H3 (rabbit) antibody	Abcam	ab1791; RRID: AB_302613)
Anti-H3K56ac (rabbit) antibody	Sigma-Aldrich/Millipore (Merck)	07-677-I; RRID: AB_390167
Chemicals, peptides, and recombinant proteins		
dATP [α-32P]	PerkinElmer	NEG512H500UC
Proteinase K	Thermo Fisher Scientific	EO0491
rDNase	Machery-Nagel	740963
RNA Fragmentation Reagents (Ambion)	Thermo Fisher Scientific	AM8740
Shrimp Alkaline Phosphatase (rSAP)	NEB	M0371L
Cap-Clip Acid Pyrophosphatase	CellScript	C-CC15011H
T4 RNA ligase 1 (high concentration)	NEB	M0437M
SuperScript IV Reverse Transcriptase	Thermo Fisher Scientific	18090050
RNasin Plus Ribonuclease Inhibitor	Promega	N2115
RNase H	NEB	M0297L
RNase cocktail	Thermo Fisher Scientific	AM2286
Dynabeads MyOne Streptavidin C1	Thermo Fisher Scientific	65002
Phenol:chloroform: Isoamyl alcohol (125:24:1)	Ambion	AM9520
Random hexamers	ThermoFisher Scientific	N8080127
HighPrep PCR beads	MagBio	AC-60050
Novex 6% TBE gels	Invitrogen	EC62655BOX
Costar SpinX column	Corning Incorporrated	8161
glass pre-filters	Whatman	1823010
linear acrylamide	Ambion	AM9520
5-Methylcytosine-dNTPs	Zymo Research	D1030
NEbuffer 2	NEB	B7002S
NAD+	NEB	B9007S
E.coli DNA ligase	NEB	M0205S
DNA polymerase I	NEB	M0209S
GsuI	ThermoFisher Scientific	ER0461
Halt Protease Inhibitor Cocktail (100X)	ThermoFisher Scientific	78429
SUPERase●In RNase Inhibitor (20 U/μL)	ThermoFisher Scientific	AM2694
FLAG® Peptide	Millipore	F3290
Critical commercial assays		
Poly(A)Purist MAG kit	Ambion	AM1922
KAPA RNA hyperPrep kit	Roche	KK8540

(*Continued on next page*)

Continued		
REAGENT or RESOURCE	SOURCE	IDENTIFIER
Qbit RNA high sensitivity assay kit	ThermoFisher Scientific	Q32852
KAPA Hyper Prep Kit	Roche	KK8504
Qubit dsDNA HS assay kit	Invitrogen	Q32851
RNeasy MinElute Cleanup Kits	QIAGEN	74204
Platinum SYBR mix	ThermoFisher Scientific	11733046
2100 Bioanalyzer	Agilent	G2939BA
KAPA Hi-Fi hot start ready mix	Roche	KK2601
KAPA single indexed adapters Set A	Roche	KK8701
KAPA single indexed adapters Set B	Roche	KK8702
Prime-It II Random Primer Labeling Kit	Agilent	300385
IME1 and *ACT1* single molecule RNA-FISH probes	Biosearch Technologies	N/A
Deposited data		
mRNA-seq, TSS-seq, TES-seq and PolII associated RNA-seq	This paper	GEO: GSE138898
Experimental models: organisms/strains		
S. cerevisiae: Strain background: BY, SK1 see Table S2	This paper	N/A
Oligonucleotides		
Oligonucleotides and primers, see Table S3	This paper	N/A
Recombinant DNA		
Plasmids, see Table S4	This paper	N/A
Software and algorithms		
Cutadapt (version 1.9.1)	(Martin, 2011)	https://cutadapt.readthedocs.io/en/stable/
STAR (version 2.5.2)	(Dobin et al., 2013)	https://github.com/alexdobin/STAR
SAMTools (version 1.3.1)	(Li et al., 2009)	http://www.htslib.org/
BEDTools (version 2.26.0)	(Quinlan and Hall, 2010)	https://bedtools.readthedocs.io/en/latest/
BigWig and BigBed	(Kent et al., 2010)	http://hgdownload.cse.ucsc.edu/admin/exe/linux.x86_64/
ImageJ (version 1.48k)	Schneider et al., 2012	https://imagej.nih.gov/ij/index.html
MATLAB 2018a	MATLAB - MathWorks - MATLAB and Simulink	RRID: SCR_001622
StarSearch	Raj Laboratory, University of Pennsylvania	https://rajlab.seas.upenn.edu/StarSearch/launch.html

RESOURCE AVAILABILITY

Lead contact

Further information and requests for resources and reagents should be directed to and will be fulfilled by the Lead Contact, Folkert van Werven (folkert.vanwerven@crick.ac.uk).

Materials availability

Plasmids and strains generated for this study are available upon request to the lead contact.

Data and code availability

The accession number for the RNA sequencing, the TSS and TES sequencing, and the Pol II associated RNA sequencind data reported in this paper is GEO: GSE138898.

The code use for the mathematical modeling is available upon request to the lead contact.

EXPERIMENTAL MODEL AND SUBJECT DETAILS

Yeast strains

Yeast strains used in this paper were derived from the BY and SK1 strain background. Gene or promoter deletions were generated using the one-step deletion protocol as described previously (Longtine et al., 1998). The strain genotypes are listed in Table S2.

Growth and conditions

All experiments were performed at 30°C in a shaker incubator at 300rpm. A protocol for rapid induction of *IME1* or *IRT1* expression was described previously (van Werven et al., 2012). In short, cells were grown till saturation for 24h in YPD (1.0% (w/v) yeast extract, 2.0% (w/v) peptone, 2.0% (w/v) glucose, and supplemented with uracil (2.5 mg/l) and adenine (1.25 mg/l)), cells were then diluted at OD_{600} = 0.4 to pre-sporulation medium (1.0% (w/v) yeast extract, 2.0% (w/v) bacto tryptone, 1.0% (w/v) potassium acetate, 50 mM potassium phthalate) grown for about 16 h, subsequently centrifuged, washed with sterile miliQ water, centrifuged again, re-suspended at OD_{600} = 1.8 in sporulation medium (SPO) (0.3% (w/v) potassium acetate and 0.02% (w/v) raffinose)) and incubated at 30°C. For Figures 1D, 2E, 2H, 4F, and S2I, we used a protocol inducing *IRT1* or *IME1* expression with slow kinetics. In short, cells were grown till saturation for 24h in YPD then diluted in YPD aiming to reach OD_{600} = 6 after 16h, several samples were taken from that point till 24h, subsequently cells were washed, and transferred to SPO.

For the lexO/LexA-ER experiments described in Figures 6E, 6F, and S6B–S6E, cells were grown till saturation for 24h in YPD, diluted (OD_{600} = 0.4) in pre-sporulation medium and grown for another 16h. Cultures were then split and treated with β-estradiol (25nM) or ethanol (mock) for 1h, transferred to SPO (OD_{600} = 1.8), and incubated up till 3h in the presence of β-estradiol (15 nM) or ethanol.

For the and *pGAL-IRT2*/GAL4-ER experiment described in Figure S2I, cells were grown till saturation for 24h in YPD, diluted (OD_{600} = 0.4) in pre-sporulation medium and grown for another 16h. Subsequently samples were taken for the indicated time points.

For measuring histone exchange rates described in Figures 6 and S6, cells were grown till saturation for 24h in YPD then shifted and grown overnight in YP raffinose 2% (YPR) till it reached OD_{600} = 0.9. Cells were then arrested in G1 with α factor (5 μg/ml) for 2h, subsequently split to YP plus 2% galactose and YPR both containing α factor (5 μg/ml).

METHOD DETAILS

Plasmids and yeasts transformation

The *RPB3-FLAG* allele was generated through a one-step C-terminal tagging procedure of *RPB3* with a FLAG tag cassette which contains three copies of the FLAG epitope (gift Jesper Svejstrup). *MAT*a/a diploid strains were generated by replacing the *MAT*α locus in *MAT*a/α diploids with a plasmid linearized by EcoRI digestion harboring the *MAT*a fragment with *URA3* or *TRP1* selectable markers (pRS304-*MAT*a, this work; pRS306-*MAT*a)(van Werven et al., 2012). The *irt2-T* allele harbors the *CYC1* terminator sequence, which was integrated in the *IRT2* locus using the ‘‘delitto perfetto’’ strategy (Storici et al., 2001). In short, *Kluyveromyces lactis URA3* marker was first integrated in the *IRT2* locus, subsequently a PCR product containing the *CYC1* terminator and homology to the flanking sequences was used to excise the *URA3* marker by 5-fluoro-orotic acid counter selection. As a control we also generated an *irt2-I* allele, which harbors a control insert from pUG6-Myc-Avitag (van Werven and Timmers, 2006). To excise the *KanMX* marker from *irt2-I*, we expressed the Cre recombinase from a plasmid (pRS304-GPDpr-CRE-EBD78-CYC1t, which is pTW040 re-cloned into pRS304, gift from Celine Bouchoux) (Terweij et al., 2013). After excision of the *KanMX* marker, genetic crosses were used to remove the pRS304-GPDpr-CRE-EBD78-CYC1t from *irt2-I*. Histone H3K56A and H3K56R mutants were generated through site directed mutagenesis using a plasmid carrying histone H3 and H4 genes along with a *URA3* marker (*HHT1* and *HHF1*,pDM9 (Sommermeyer et al., 2013), gift from Valerie Borde). Mutant plasmids were transformed into a strain with all four genomic copies of histone H3 and H4 genes deleted and harboring a plasmid wild-type for the histone H3 and H4 genes on TRP1 selectable marker (*HHT2* and *HHF2,* pVB140 (Sommermeyer et al., 2013), gift from Valerie Borde). Genetic crosses were used to generate the histone H3K56A and H3K56R mutants in the absence of the wild-type histone H3 covering plasmid. The plasmids for generating strains with LexA operator (lexO) sequences and LexA fused to the estrogen receptor domain and activation domain (LexA-ER-HA-B112) were described previously (gift from Elçin Ünal) (Chia et al., 2017) (Ottoz et al., 2014). A different number of LexO sites were integrated at *IRT2* locus position +96 or −10 bp from the Ume6 binding site. A plasmid expressing LexA-ER-HA-B112 from the *GPD1* promoter was linearized with SfiI restriction enzyme and integrated at the *TRP1* locus. The strains used for the histone H3 exchange assay described in Figures 6 and S6 were described previously (Schermer et al., 2005). In short, we used a strain with the endogenous copies of histone H3 and H4 deleted and covered by a centromeric plasmid pNOY439 harboring *HHT2*-Myc (histone Myc-H3) and untagged *HHF2* (histone H4) under control of their endogenous promoters. In addition this stain also harbored a plasmid (YIplac211 pGAL1-10 HHF1 FLAG-HHT1) integrated at the *URA3* locus with *HHT1* FLAG tagged (histone FLAG-H3) and untagged *HHF1* (histone H4) under control of the *GAL1-10* promoter (Schermer et al., 2005). To generate *pGAL-IRT2* strain, the *GAL1-10* promoter was integrated 10 bp upstream of the Ume6 binding site. The *GAL4-ER* expression construct was described previously (Carlile and Amon, 2008). Plasmids are listed in Table S4.

Oligonucleotides Used in This Study

A table of oligonucleotides used in this study is available in Table S3.

Nuclei/DAPI counting

DAPI staining was used to monitor meiotic divisions throughout time courses. Cells were fixed in 80% (v/v) ethanol, pelleted by centrifugation and re-suspended in 100 mM phosphate buffer (pH 7) with 1 μg/ml 4′,6-diamidino-2-phenylindole (DAPI). Cells were then sonicated for a few seconds and left in the dark at room temperature for at least 5 min. The proportion of cells containing one, two, three or four (meiosis I + II) DAPI masses were counted using a fluorescence microscope.

Chromatin immunoprecipitation

Chromatin immunoprecipitation (ChIP) experiments were performed as described previously (Moretto et al., 2018). Cells were fixed in 1.0% w/v formaldehyde for 25 min at room temperature and quenched with 100 mM glycine. Cells were lysed in FA lysis buffer (50 mM HEPES–KOH, pH 7.5, 150 mM NaCl, 1mM EDTA, 1% Triton X-100, 0.1% Na-deoxycholate, 0.1% SDS and protease cocktail inhibitor used as recommended by the manufacturer (complete mini EDTA-free, Roche)) using beadbeater (BioSpec) and chromatin was sheared by sonication using a Bioruptor (Diagenode, 8 cycles of 30 s on/off). Extracts were incubated for 2 h at room temperature with anti-V5 agarose beads (Sigma) or overnight at 4°C with magnetic Prot A beads (Sigma) coupled with a polyclonal antibody raised against Histone H3 (Ab1791, Abcam) or Histone H3 acetylated lysine 56 (07-677-I, Millipore), washed twice with FA lysis buffer, twice with wash buffer 1 (FA lysis buffer containing 0.5M NaCl), and twice with wash buffer 2 (10 mM Tris–HCl, pH 8.0, 0.25M LiCl, 1 mM EDTA, 0.5% NP-40, 0.5% Na-deoxycholate). Subsequently, reverse cross-linking was done in 1% SDS-TE buffer (100 mM Tris pH 8.0, 10 mM EDTA, 1.0% v/v SDS) at 65°C overnight. After 2 h of proteinase K treatment, samples were purified, and DNA fragments were quantified by real-time PCR using SYBR green mix (Life Technologies) using primers described in Table S3. Signals were normalized over the *HMR* locus, which showed no binding for Rme1. For Figure 4G, H3K56ac ChIP signals were normalized to histone H3 ChIP. For the histone H3 turnover experiments described in Figures 6 and S6, ChIPs were performed as described above using antibodies against the Myc epitope (clone 9E11, Thermofisher) or against the FLAG epitope (M2 beads, Sigma). Signals were normalized using primers directed against a telomeric region from chromosome VI. For the Rpb3-FLAG ChIPs, we used FLAG-antibody coupled beads (M2 beads, Sigma). Signals were normalized over the *HMR* locus, which showed no binding for Rpb3.

Micrococcal nuclease (MNase) qPCR

Nucleosome positioning at the *IRT2* locus was determined by quantifying the abundance of mononucleosomal DNA using a MNase digestion protocol that was described previously (Rando, 2010). In short, approximately 90 OD_{600} units of cells were crosslinked for 25 min at 30°C with 1% (v/v) formaldehyde. Reaction was quenched with the addition of glycine to 125 mM. Subsequently, cells were re-suspended in 20 mL of buffer Z (1 M sorbitol, 50 mM Tris-HCl pH 7.4**)** plus β-mercaptoethanol (10mM) and treated with 250 μg of T100 Zymolyase for 60 min. Next, cells were re-suspended in 1 mL NP buffer (0.5 mM spermidine, 1 mM β-mercaptoethanol (β -ME), 0.075% (w/v) Tergitol solution-type NP-40 detergent (NP-40), 50 mM NaCl, 10 mM Tris-HCl pH 7.4, 5 mM $MgCl_2$, 1 mM $CaCl_2$), vortexed for 10 s, and 100 μL of extract was treated with 0.2 μL of MNase (2mg/ml, NEB) for 30 min at 37°C, the reaction was quenched with 10 mM EDTA, and reverse crosslinked overnight in 1% SDS-TE and 4 units per ml of proteinase K (NEB). Samples were treated with RNase A and purified DNA fragments were separated by gel electrophoresis before gel purification of the mono-nucleosome bands. MNase treated and input samples were quantified by qPCR on a 7500 FAST Real-Time PCR machine (Life Technologies) using Platinum SYBR mix (Thermofisher). The signals were normalized using primers directed against a telomere locus. The scanning primer pairs covering the *IRT2* locus and downstream region used for the analysis are available in Table S3.

Northern blotting

Northern blots were performed as described previously (Moretto et al., 2018). In short, total RNA was extracted with Acid Phenol:-chloroform:Isoamyl alcohol (125:24:1) and precipitated in ethanol with 0.3 M sodium acetate. RNA samples were denatured in a glyoxal/DMSO mix (1 M deionized glyoxal, 50% v/v DMSO, 10 mM NaPi buffer pH 6.5-6.8) at 70°C for 10 min. Samples were mixed with loading buffer (10% v/v glycerol, 2 mM NaPi buffer pH 6.8, 0.4% w/v bromophenol blue) and separated on an agarose gel (1.1% w/v agarose, 0.01 M NaPi buffer pH 6.8) for 2 h at 80 V. RNAs were then transferred onto nylon membranes overnight by capillary transfer in 0.025 M NaPi buffer pH 6.8. Membranes were blocked for 2–3 h at 42°C in Hybridization buffer (1% w/v SDS, 50% v/v de-ionized formamide, 25% w/v dextran sulfate, 58 g/L NaCl, 200 mg/L herring sperm single strand DNA, 2 g/L BSA, 2 g/L polyvinyl-pyrrolidone, 2 g/L ficoll, 1.7 g/L pyrophosphate, 50 mM Tris pH 7.5) before hybridization. Radioactive probes were synthesized using a Prime-It II Random Primer Labeling Kit (Agilent), a target-specific DNA template and dATP [α-32P] (Perkin-Elmer). To avoid differences in signal intensity due to labeling quality and blotting variations, for each experiment samples were run on a single gel, transferred onto a single membrane, and hybridized with probes in a single hybridization tube. Background normalized quantifications of the Northern blots were done using Imagej software (Schneider et al., 2012). The oligo nucleotide sequences used to generate target-specific DNA template for amplifying the Northern blot probes are displayed in Table S3. Data S1 contains all the raw blots.

RNA-seq

At least 5 μg of total RNA was treated with DNase and purified on column (Macherey-Nagel). At least 500 ng of purified total RNA was used as input for the KAPA mRNA Hyper Prep kit (KK8580, Roche). Libraries were prepared according to manufacturer's instructions. After bead based clean up, libraries were sequenced on an Illumina HiSeq 2500 to an equivalent of 75 bases single-end reads, at a depth of approximately 20 million reads per library.

TSS-seq and TES-seq

The TSS sequencing approach was adapted and modified from previously published protocols (Adjalley et al., 2016) (Arribere and Gilbert, 2013) (Malabat et al., 2015). At least 5 μg of mRNAs were purified from total RNA using the Poly(A)Purist MAG kit (AM1922, Ambion). poly(A)$^+$ RNA/mRNAs, together with *in vitro* spike-ins, were fragmented for 3 min at 70°C using a Zinc-based alkaline fragmentation reagent (AM8740, Ambion). RNAs were cleaned up using RNeasy MinElute Cleanup Kits (74204, QIAGEN) to enrich for 200-300 nt fragments. These fragments were dephosphorylated with 30 units of recombinant shrimp alkaline phosphatase (M0371, NEB) for 1 h at 37°C with RNasin Plus, the phosphatase was heat inactivated and the RNA was extracted with Acid Phenol:-chloroform: Isoamyl alcohol (125:24:1) and precipitated at −20°C overnight in ethanol with 0.3M sodium acetate and 1 μL linear acrylamide (AM9520, Ambion). RNA was then subjected to a decapping reaction with 2 units of Cap-Clip acid pyrophosphatase (C-CC15011H, Tebu-Bio) and with RNasin Plus. RNAs were then extracted using acid Phenol:chloroform:isoamyl alcohol (125:24:1) and precipitated in ethanol. Some RNA from a SPO (starvation) 0 h time point was set apart without the decapping reaction as a non-decapping control. Subsequently, the RNA was mixed with 10 μM of custom 5′ adaptor (CACTCTrGrArGrCrArArUrArCrC) and the ligation reaction was done using T4 RNA ligase 1 (M0437M, NEB) and with RNasin Plus. The ligation reaction was cleaned up with the RNeasy MinElute Cleanup Kit and RNAs were mixed with 2.5 μM random hexamers (N8080127, ThermoFisher Scientific) and RNasin Plus, denatured at 65°C for 5 min and cooled on ice. Reverse transcription reactions were carried out using SuperScript IV reverse transcriptase (18090010, Invitrogen) at 23°C for 10 min, 50°C for 10 min, 80°C for 10 min and held at 4°C. The RNA templates were degraded by incubating reactions with 5 units of RNase H (M0297, NEB) and 1.0 μL of RNase cocktail enzyme mix (AM2286, Ambion). DNA products were purified using 1.8x volume of HighPrep PCR beads (AC-60050, MagBio). Purified products were subjected to second strand synthesis using 0.3 μM of second strand biotinylated primer (GCAC/iBiodT/GCACTCTGAGCAA TACC) and the KAPA Hi-Fi hot start ready mix (KK2601, Roche). The second strand reaction was carried out at 95°C for 3 min, 98°C for 15 s, 50°C for 2 min, 65°C for 15 min and held at 4°C. Double stranded product (dsDNA) was purified with 1.8x volume High-Prep PCR beads and concentration was quantified using the Qubit dsDNA HS assay kit (Q32851, Invitrogen). At least 1 ng of dsDNA was then used as input for the KAPA Hyper Prep Kit (KK8504, Roche) and ligated to KAPA single indexed adapters Set A (KK8701, Roche) or Set B (KK8702, Roche). Samples were processed according to manufacturer's instructions with one exception: just prior to the library amplification step, samples were bound to MyOne Streptavidin C1 Dynabeads (65001, ThermoFisher Scientific) to capture biotinylated dsDNA. Library amplification was done on the biotinylated dsDNA fraction bound to the beads. Depending on the input amounts, 15 PCR cycles were used to generate libraries. Amplified libraries were quantified by Qubit, and adaptor-dimers were removed by electrophoresing libraries on Novex 6% TBE gels (EC62655BOX, Invitrogen) at 120 V for 1 h, and excising the smear above ~150 bp. Gel slices containing libraries were shredded by centrifugation at 13000 g for 3 min. Gel shreds were re-suspended in 500 μL crush and soak buffer (500 mM NaCl, 1.0 mM EDTA and 0.05% v/v SDS) and incubated at 65°C for 2 h on a thermomixer (1400 rpm for 15 s, rest for 45 s). Subsequently, the buffer was transferred into a Costar SpinX column (8161, Corning Incorporrated) with two 1 cm glass pre-filters (1823010, Whatman). Columns were centrifuged at 13000 g for 1 min. DNA libraries in the flowthrough were precipitated at −20°C overnight in ethanol with 0.3 M sodium acetate and 1 μL linear acrylamide (AM9520, Ambion). Purified libraries were further quantified and inspected on a Tapestation (Agilent Technologies) and sequenced on an Illumina HiSeq 2500 to an equivalent of 75 bases single-end reads, at a depth of approximately 20 million reads per library.

The TES sequencing approach was adapted and modified from previously published protocols (Lai et al., 2015) (Ng et al., 2005). From the same pool of fragmented mRNAs describe above in the 5′ end sequencing, at least 1 μg was used for 3′ end sequencing. RNA fragments were mixed with 2.5 μM GsuI20TVN primer (/5BiotinTEG/ GAGCTAGTTCTGGAGTTTTTTTTTTTTTTTTTTTTVN), 0.5 mM 5-Methylcytosine-dNTPs (D1030, Zymo Research) and 0.5 μL RNasin Plus. Reaction mixtures were denatured at 65°C for 5 min and held at 50°C without allowing to cool. SuperScript IV, reaction buffer and 0.4 μg of Actinomycin D were added to the hot reaction mixtures and reverse transcription was performed at 50°C for 10 min, 80°C for 10 min and held at 4°C. Samples were cleaned with 1.8x volume HighPrep beads and biotinylated RNA:DNA hybrids were captured on MyOne Streptavidin C1 Dynabeads. After capture, streptavidin beads were washed once with 1x NEbuffer 2 (B7002S, NEB), re-suspended in water and subjected to second strand synthesis. The 50 μL second strand synthesis reaction consisted of 20 μL re-suspended streptavidin beads, 1X NEbuffer 2, 250 μM dNTPs, 26 μM NAD+ (B9007S), 2.5 units RNase H, 10 units E.coli DNA ligase (M0205S), and 15 units DNA polymerase I (M0209S). Second strand synthesis reactions were conducted at 16°C for 2.5 h on a thermomixer (1400 rpm for 15 s, rest for 2 min). After reaction, beads were washed once with 1x binding and washing buffer (5.0 mM Tris-HCl pH 7.5, 0.5 mM EDTA, 1.0 M NaCl) and once with buffer B (10 mM Tris-HCl pH 7.5, 10 mM MgCl2, 0.1 mg/ml BSA). Washed beads were re-suspended in 18 μL buffer B and digested with 10 units of GsuI (ER0461, ThermoFisher Scientific) at 30°C for 1 h on a thermomixer (1400 rpm for 15 s, rest for 2 min). After digestion, the DNA fragments in the supernatant were extracted with Phenol/chloroform and precipitated at −20°C overnight in ethanol with 0.3 M sodium acetate and 1 μL linear acrylamide. The concentration was quantified using the Qubit dsDNA HS assay kit. At least 1 ng of dsDNA was then used as input for the KAPA Hyper Prep Kit (KK8504, Roche) and ligated to KAPA single

indexed adapters Set A (KK8701, Roche) or Set B (KK8702, Roche). Samples were processed according to manufacturer's instructions. Amplified libraries were cleaned and purified by gel extraction using the procedures described in the previous section for TSS sequencing. Purified libraries were further quantified and inspected on a Tapestation (Agilent Technologies) and sequenced on an Illumina HiSeq 2500 to an equivalent of 75 bases single-end reads, at a depth of approximately 20 million reads per library.

Nascent RNA-Seq

The nascent RNA sequencing procedure was adapted as described in (Churchman and Weissman, 2011). Briefly, about one liter of yeast culture was spin down at the harvest time point and pellet snap frozen in liquid nitrogen. Cell pellets were then grinded in fine powder using a cryo-mill instrument with the instrument maintained cooled with liquid nitrogen. All the powder was re-suspended in about 12 to 15 mL of lysis buffer (20 mM HEPES pH 7.4, 110 mM K acetate, 0.5% Triton X-100, 0.1% Tween-20, 10 mM MnCl2, 1X protease inhibitor cocktail HALT, SUPERasin RNase inhibitor 50 U/ml) and lysate subjected to DNase I treatment (1200 U per sample) for 20 min. Lysate was then clarified through two rounds of centrifugation at about 20 000 g for 10 min at 4C. Anti-FLAG immunoprecipitation was performed using 750 μL of anti-FLAG M2 agarose beads per sample and for 2.5 h at 4C on a rotating wheel. Beads were then washed 5 times using 10 mL of cold wash buffer (20 mM HEPES pH 7.4, 110 mM K acetate, 0.5% Triton X-100, 0.1% Tween-20, 10 mM MnCl2, SUPERasin RNase inhibitor 1 U/ml, 1 mM EDTA) for 5 min at 4C. Beads were then cleaned using a Pierce column and two rounds of elution were performed using FLAG peptide (450 μL 10 mg/ml Sigma FLAG peptide) for 30 min at 4C. Elution was then subjected to a phenol:Chloroform extraction and RNA precipitated overnight in ethanol containing 0.3 M Na acetate. RNAs were then resuspended in Nuclease free water, quantified by Qbit RNA high sensitivity assay kit (Q32852, ThermoFisher Scientific) and analyzed by Bioanalyser (Agilent technologies).

At least 500 ng of purified RNA was treated with DNase and purified on column (Macherey-Nagel). Then, about 100 ng of purified total RNA was used as the inputs for the KAPA RNA hyperPrep kit (KK8540, Roche). Libraries were prepared according to manufacturer's instructions. After bead based clean up, libraries were sequenced on an Illumina HiSeq 4000 to an equivalent of 75 bases single-end reads, at a depth of approximately 50 million reads per library.

Bioinformatic analysis

For RNA-seq data (including nascent RNA-seq), adaptor trimming was performed with cutadapt (version 1.9.1) with parameters "-a AGATCGGAAGAGCACACGTCTGAACTCCAGTCAC–minimum-length=20." STAR (version 2.5.2) with parameters "–alignIntronMin 3–alignIntronMax 5000" was used to perform the read mapping to the *S. cerevisiae* SK1 genome assembly from Keeney lab (http://cbio.mskcc.org/public/SK1_MvO/) (Martin, 2011). Alignments with mapping quality of < 10 or soft/hard-clipping were filtered (Dobin et al., 2013) (Martin, 2011). Alignments from forward strand and reverse strand were separated by using "samtools view -b -f 0x10" and "samtools view -b -F 0x10" to split the alignments. The tool "bedtools genomecov" (Quinlan and Hall, 2010) was used to generate the RNA-seq BedGraph tracks across the genome, for both forward and reverse strands. All the reads mapping to the rRNA were trimmed from the analyses prior the normalization. The BedGraph tracks were normalized by the number of usable reads in each library. BedGraph files were converted to bigWig using the tool bedGraphToBigWig from UCSC (Kent et al., 2010).

For the TSS transcript sequencing data, the custom 5′ adaptor sequence specific to the protocol was removed by re-running cutadapt with the parameters "-g CACTCTGAGCAATACC -O 16–minimum-length=20," and only the reads containing the adaptor sequence were used for further analysis. STAR (version 2.5.2) with parameters "–alignIntronMin 2–alignIntronMax 1" (i.e., not allowing introns) was used to align TSS-seq reads to the SK1 genome assembly (plus three spike-in sequences) (Dobin et al., 2013). The alignments with mapping quality of > = 10 were kept for further analysis. Alignments from forward strand and reverse strand were separated by using "samtools view -b -f 0x10" and "samtools view -b -F 0x10." The 5′-most nucleotide of aligned reads were extracted to generate the genome-wide tracks of TSSs. The BedGraph tracks were normalized by the number of usable reads in each library.

For the TES transcript sequencing data, Adaptor trimming was performed with cutadapt (version 1.9.1) with parameters "-a AGATCGGAAGAGCACACGTCTGAACTCCAGTCAC–minimum-length=20."

STAR (version 2.5.2) with parameters "–alignIntronMin 2–alignIntronMax 1" as for the 5′ end sequencing part, and alignments with mapping quality of > = 10 were kept for further analysis. The reads kept were those with soft-clipping at the 3′ end (size of soft-clipping part ≤ 10) and with at least two consecutive non-templated as in the soft-clipping part. Insertions/deletions were also not allowed. Alignments from forward strand and reverse strand were separated by using "samtools view -b -f 0x10" and "samtools view -b -F 0x10." The 3′-most nucleotide of aligned reads were extracted to generate the genome-wide tracks of TESs. The BedGraph tracks were normalized by the number of usable reads in each library.

Single molecule RNA fluorescent *in situ* hybridization (FISH)

The single molecule RNA FISH was performed as described previously (Moretto et al., 2018). In short, cells were fixed with formaldehyde overnight, treated with zymolyase and further fixed in 80% ethanol. Subsequently cells were hybridized with fluorophore labeled probes directed to *IME1* (AF594) and *ACT1* (Cy5) as an internal control. Cells were imaged using a 100x oil objective, NA 1.4, on a Nikon TI-E imaging system (Nikon). DIC, DAPI, AF594 (*IME1*), Cy5 (*ACT1*) images were collected every 0.3 micron (20 stacks) using an ORCA-FLASH 4.0 camera (Hamamatsu) and NIS-element software (Nikon). ImageJ software was used to make maximum intensity Z projections of the images (Schneider et al., 2012). StarSearch software (https://rajlab.seas.upenn.edu/StarSearch/launch.html, Raj laboratory, University of Pennsylvania) was used to quantify transcripts in single cells. Comparable thresholds were used to

count RNA foci in single cells. Only cells positive for the internal control *ACT1* were used for *IME1* analysis. At least a total n = 150 cells were counted for each experiment.

Western blotting

Western blots were performed as previously described (Moretto et al., 2018). Protein extracts were prepared using the trichloroacetic acid (TCA) extraction protocol. After SDS-polyacrylamide gel electrophoresis (4%–20% gradient), proteins were transferred onto PVDF membranes. The membranes were then incubated overnight primary antibodies in blocking buffer. Mouse anti-v5 (R96025, Sigma-Aldrich (MO, USA)) was used at a 1:2000 dilution and rabbit anti-hexokinase antibody (H2035, Stratech (Newmarket, UK)) at a 1:8000 dilution. Membranes were then washed in PBST buffer and incubated with IRDye 800CW goat anti-mouse and IRDye 680RD donkey anti-rabbit secondary antibodies (LI-COR (NE, USA)) at a 1:15000 dilution for LI-COR detection. Protein levels were detected on an Odyssey Imager for LI-COR detection.

Spot growth assay

Cells were grown using the protocol for rapid induction of *IRT1*. After 0 or 14 days in SPO cells were diluted to OD_{600} = 1 in sterile water, and serial dilutions (5-fold) were spotted onto YPD agar plates. Cells were incubated at 30°C for 2 days before imaging.

Mathematical modeling

To investigate of the complex regulation of *IME1* expression by *IRT1* and *IRT2*, we developed a mathematical model. We incorporated new observations described in this manuscript, into the model described previously (Moretto et al., 2018). Specifically, the new mathematical model describes both the activating and repressing properties of *IRT2* on *IRT1* expression. The *IRT2* activating property is incorporated by controlling the *IRT1* transcription rate with the indicator function, χ, which is 1 when *IRT2* transcription rate reaches an activating level threshold, A. After the activation of *IRT1* χ remains at 1 even when *IRT2* transcription rate falls below A. In addition, the model includes Rme1 concentration, *r*, which is assumed to be constant during the time-period of Ime1 activation. By changing Rme1 concentrations, *r*, we can simulate both haploid and diploid cell-types, which differ significantly in their Rme1 levels. The model variables are listed below. The parameters have been chosen to reproduce the qualitative behavior of this system.

List of variables (scaled between 0 and 1)

s	Starvation signal
$I1$	*IRT1* transcription rate
$I2$	*IRT2* transcription rate
I_t	Ime1 transcription rate
I_m	Ime1 mRNA concentration
I_p	Ime1 protein concentration

List of parameters used for simulation

k_1	controls the strength of $I1$ inhibition by $I2$	5
k_2	I_p threshold for activating $I2$	0.05
k_3	controls the strength of I_t inhibition by $I1$	10
k_4	degradation rate of I_m	1 /h
k_5	synthesis rate of I_p	1 /h
k_6	degradation rate of I_p	1 /h
r	Rme1 concentration	0-5
A	$I2$ threshold for activating $I1$	0.01

Equations

$$s(t) = 1, \text{ for } t \geq 0 \tag{1}$$

$$\chi = \begin{cases} 1, & I2 \geq A \\ 0, & \text{otherwise} \end{cases} \quad or\ I1 > 0 \tag{2}$$

$$I1 = \begin{cases} \chi \dfrac{r}{r + k_1 I2}, & r > 0 \text{ or } I2 > 0 \\ 0, & \text{otherwise} \end{cases} \quad (3)$$

$$I2 = \begin{cases} I_p - k_2, & Ip \geq k_2 \\ 0, & \text{otherwise} \end{cases} \quad (4)$$

$$\frac{dI_m}{dt} = \begin{cases} \dfrac{s}{s + k_3 I1} - k_4 I_m, & s > 0 \\ -k_4 I_m, & \text{otherwise} \end{cases} \quad (5)$$

$$\frac{dI_p}{dt} = k_5 I_m - k_6 I_p \quad (6)$$

The model is simulated with the initial conditions $I_m(0) = 0$ and $I_p(0) = 0$.

The first term in Equation 5, $(s / s + k_3 I1)$, describes the *IME1* transcription rate.

Equation 1 models the starvation signal as a step input. Upon starvation, *IME1* mRNA I_m and in turn Ime1 protein I_p are synthesized. The evolution of I_m is given by Equation 5 and of I_p is given by Equation 6. When I_p reaches the threshold k_2, *IRT2* transcription, *I2*, turns on. Here we assumed that *IRT2* transcription closely follows Ime1 protein concentration (Equation 4). Once *I2* reaches the threshold *A*, the indicator function χ is set at 1 (Equation 2) and *IRT1* transcription starts(Equation 3). Note that χ remains equal to 1, even if *I2* falls below *A*, as long as *I1* is greater than 0. *I1* is driven by the Rme1 concentration, *r*, which is assumed to be constant in the cell through the time period of Ime1 activation. Activation of *I1* suppresses *Ip* to various degrees depending on *r* (Figures 7A and S7A). The mathematical model shows how *IRT1* and *IRT2* control expression of *IME1* and Ime1 protein. Activating *IRT2* and no *IRT2* transcription are simulated by setting $I2 = A$ and $I2 = 0$, respectively, for $t > 0$ (Figures 7D and S7C). The mathematical model is simulated in MATLAB 2018a using ode45 function. Code is available upon request.

QUANTIFICATION AND STATISTICAL ANALYSIS

Statistical significance and tests are indicated in the figure legends and were performed using GraphPad Prism 7 and 8. When comparing only two groups of samples, we have used a parametric two-tailed Student's t test. When assessing the variance of more than two groups we performed an ANOVA analysis (one way or two ways in function of the experimental design), followed by a Fisher LSD test. Fisher's LSD test confer more power to the test, compared to performing multiple Student's t tests, in that t tests compute the pooled standard deviations from only the two groups being compared, while the Fisher's LSD test computes the pooled standard deviations from all the groups while assuming that all population have similar standard deviation.

Cell Reports, Volume *34*

Supplemental Information

Transcription levels of a noncoding RNA orchestrate opposing regulatory and cell fate outcomes in yeast

Fabien Moretto, N. Ezgi Wood, Minghao Chia, Cai Li, Nicholas M. Luscombe, and Folkert J. van Werven

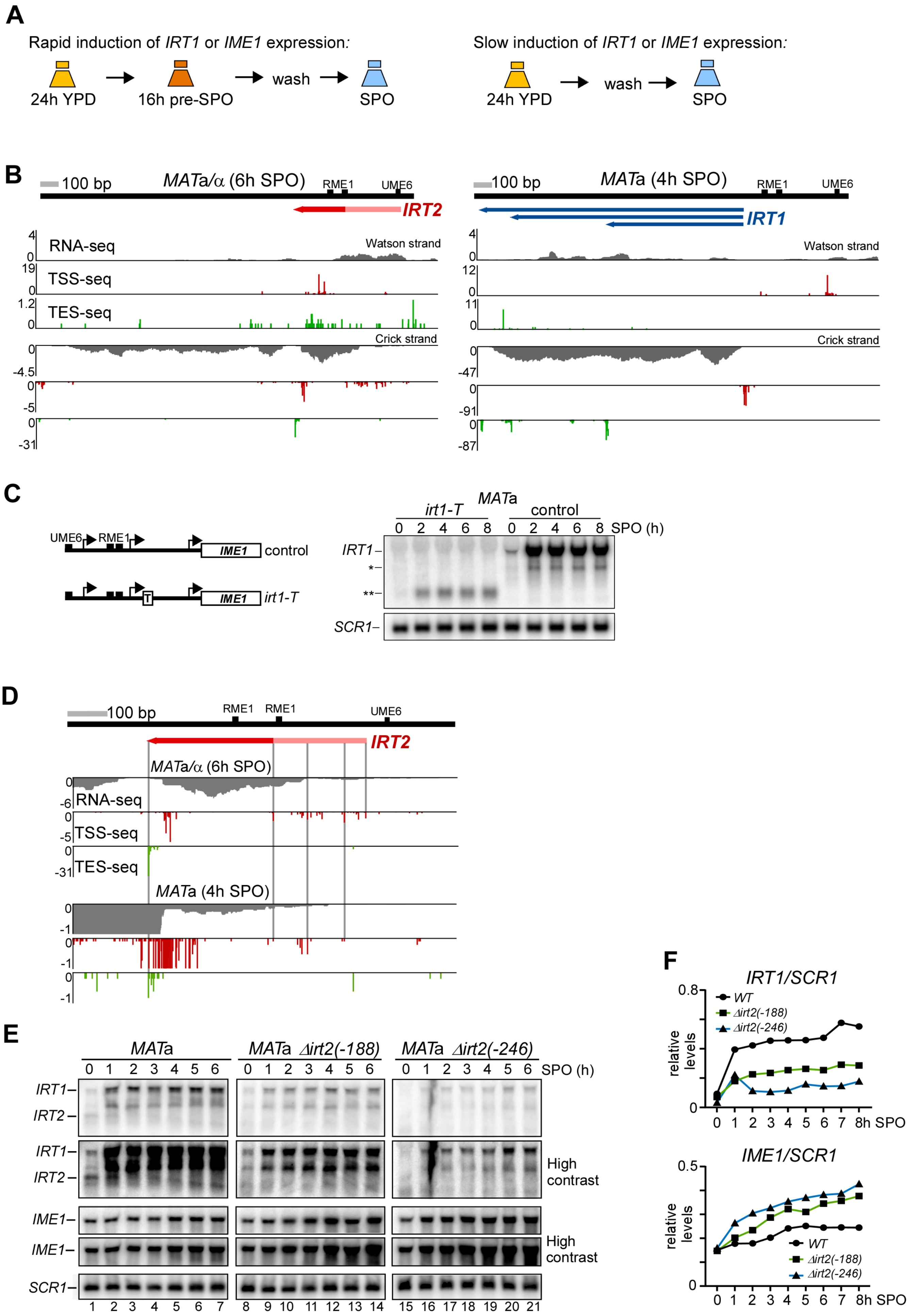
A
Rapid induction of IRT1 or IME1 expression:
24h YPD
16h pre-SPO
wash
SPO
Slow induction of IRT1 or IME1 expression:
24h YPD
wash
SPO
B
100 bp
MATa/α (6h SPO)
RME1
UME6
IRT2
RNA-seq
TSS-seq
TES-seq
Watson strand
Crick strand
100 bp
MATa (4h SPO)
IRT1
C
UME6
RME1
IME1
control
T
irt1-T
MATa
SPO (h)
IRT1
SCR1
D
100 bp
RME1
RME1
UME6
IRT2
MATa/α (6h SPO)
RNA-seq
TSS-seq
TES-seq
MATa (4h SPO)
E
MATa
MATa Δirt2(-188)
MATa Δirt2(-246)
SPO (h)
IRT1
IRT2
High contrast
IME1
SCR1
F
IRT1/SCR1
WT
Δirt2(-188)
Δirt2(-246)
relative levels
8h SPO
IME1/SCR1

Figure S1: related to Figure 1

***IRT2* is required for *IRT1* expression**

A, Scheme of growth conditions used throughout this study. Rapid induction of *IRT1* or *IME1* expression was achieved by growing cells in rich medium (YPD) till saturation (24 h), subsequently cells were shifted to pre-sporulation (pre-SPO) medium and grown for another 16 h. After a wash with sterile water, cells were transferred into sporulation medium (SPO). Slow induction of *IRT1* or *IME1* expression was achieved by shifting cells directly after growth till saturation in YPD to SPO.

B, RNA-seq, TSS-seq and TES-seq tracks of *IRT2* and *IRT1* in *MAT*a/α 6h in SPO (FW1511) and *MAT*a 4h in SPO (FW1509) cells. Displayed are the signals of normalized reads for both the Watson and Crick strands. Cells were grown as described in A (Rapid induction of *IRT1*).

C, *IRT1* expression as detected by northern blot in control (FW1509) and *irt1-T* strains (FW155). The *irt1-T* strain harbors a transcriptional terminator ~ 200 bp after the *IRT1* TSS. Cells were grown as described in A (Rapid induction of *IRT1*). *SCR1* was used as a loading control.

D, Similar data as in B, except that *IRT2* is highlighted, which demonstrates that *IRT2* is transcribed in *MAT*a cells (4h in SPO).

E, *IRT1*, *IRT2*, and *IME1* expression in WT *MAT*a cells (FW1509), and Δ*irt2*(-188) (FW1210) and Δ*irt2*(-246) (FW1356) mutants as presented in Figure 1E. Images with adjusted signals for both *IRT1-IRT2* combined probe and *IME1* blots are also included.

F, Northern blot quantification of *IRT1* (top) and *IME1* (bottom) signals from the blots presented in E. Signals from the blots were background corrected and normalised over loading control signal *SCR1*.

Moretto *et al*. Figure S2

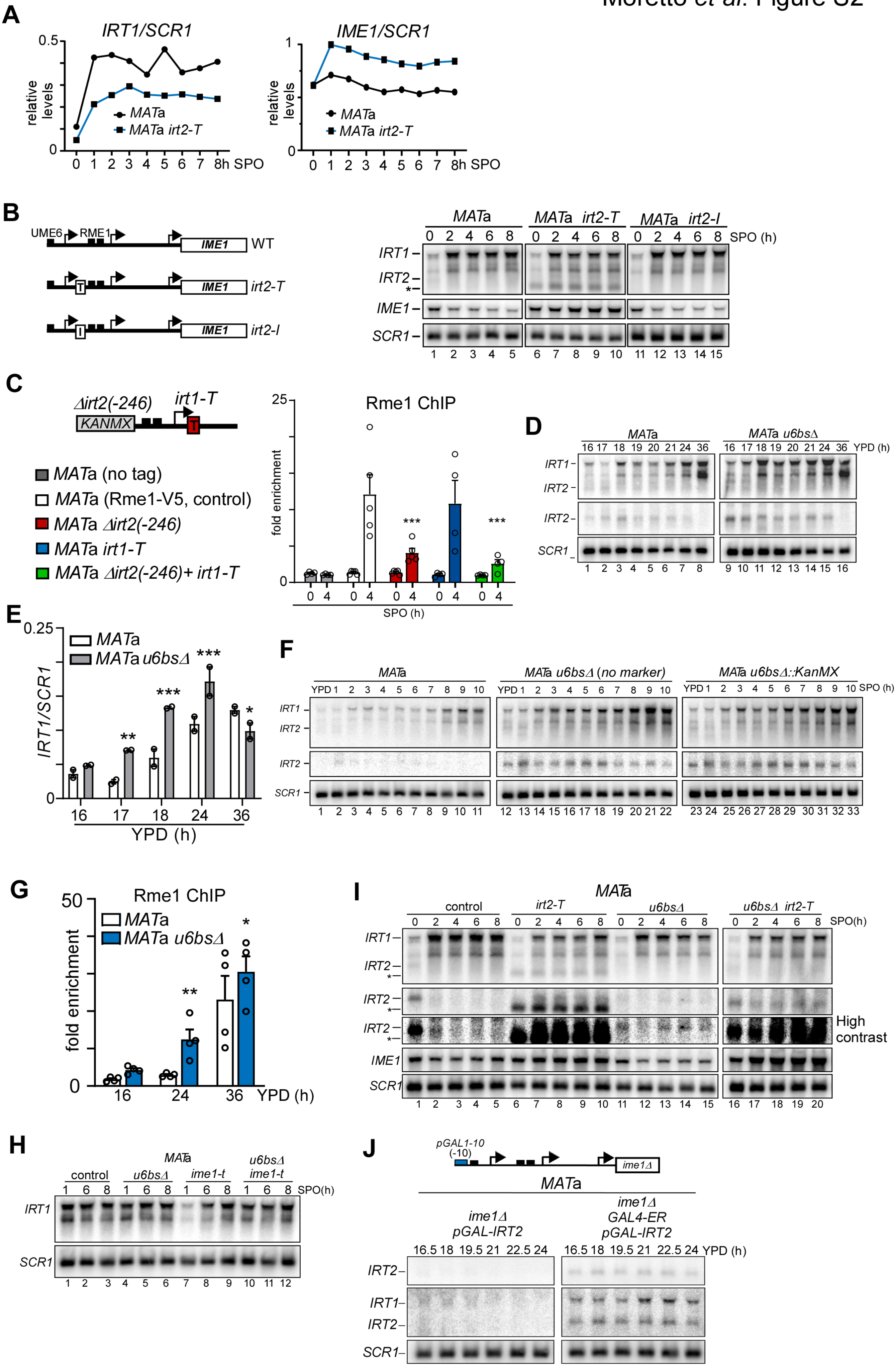

Figure S2: related to Figure 2

***IRT2* transcription is required and sufficient for inducing *IRT1* expression**

A, Northern blot quantification of *IRT1* (left) and *IME1* (right) signals from the blots presented in 2B. Signals were background corrected and normalised over loading control signal *SCR1*.

B, Scheme of the *IME1* locus harboring a transcriptional terminator *(irt2-T)* or a control insert (*irt2-I*) in *IRT2* (left). *IRT1*, *IRT2*, and *IME1* expression in WT *MAT*a cells (FW1509), Δ*irt2-T* (FW3596) and Δ*irt2-I* (FW4175) as detected by northern blot (right). *SCR1* was used as a loading control. The asterisk depicts the prematurely terminated form of *IRT2*. Cells were grown in YPD to saturation (24 h), shifted to pre-SPO (16 h), and subsequently transferred to SPO.

C, Rme1 ChIP followed by qPCR using oligos nested in *IRT2* in no tag control, *RME1-V5* control, *Δirt2(-246)* and *irt1-T* single mutants and *Δirt2(-246) irt1-T* double mutant (FW1509, FW4031, FW3140, FW155 and FW8909). Cells were grown as in B. qPCR were performed using a primer pair directed to Rme1 binding sites in the *IRT1* promoter. Signals were normalized to *HMR*. The error bars represent the standard error of the mean (SEM) of n = 3 biological repeats. *** correspond to a p-value < 0.0005 on a two-way ANOVA analysis followed by a Fisher's LSD test comparing the mutant strains with the control strain.

D, *IRT1* and *IRT2* expression in WT (FW1509) and *u6bs*Δ *MATa* cells (FW2438). Cells were grown in YPD to saturation and sampled at the indicated time points.

E, Quantification of *IRT1* expression described in D. *IRT1* signals were normalized over *SCR1*. n = 2 +/- SEM. *, ** and *** correspond to p-value < 0.05, < 0.005, < 0.0005 respectively on a two-way ANOVA analysis followed by a Fisher's LSD test.

F, *IRT1* and *IRT2* expression in *MAT*a cells (FW1509) and *u6bs*Δ cells harboring the deletion of the Ume6 binding site in absence of a selectable marker *u6bs*Δ (FW1378) or with a selectable marker *u6bsΔ::KanMX* (FW2438). Cells were grown in YPD to saturation and subsequently transferred to SPO.

G, ChIP of Rme1-V5 at *IRT1* under the same condition described in D, except for the presence of the *RME1-V5* allele in *MAT*a WT (FW4031) and *u6bs*Δ (FW3144). n = 4 +/- SEM. * and ** correspond to a p-value < 0.05 and < 0.005 respectively on a two-way ANOVA followed by Fisher's LSD test comparing *u6bs*Δ to the *MAT*a control.

H, *IRT1* and *IRT2* expression in *MAT*a cells (FW1509), *u6bsΔ::KanMX* (FW2438), *ime1-t* (FW2189) and *u6bsΔ::KanMX ime1-t* double mutant (FW2539). Cells were grown as in B.

I, *IRT1*, *IRT2*, and *IME1* expression in *MAT*a cells (FW1509), Δ*irt2-T* (FW3596), *u6bsΔ::KanMX* (FW2438) and *u6bsΔ::KanMX* Δ*irt2-T* double mutant (FW3493). Cells were grown as in B. An image presenting the adjusted signal highlighting *IRT2* expression from the northern blot is included.

J, *IRT1* and *IRT2* expression in *MAT*a cells harboring *ime1*Δ and the *GAL1-10* promoter integrated -10 bp from the *Ume6* binding site at *IRT2* site (*pGAL-IRT2*) (FW8759), or the same cells also expressing *GAL4-ER* (FW8758). Samples were taken from cells grown in YPD till the indicated time-points.

Moretto *et al.* Figure S3

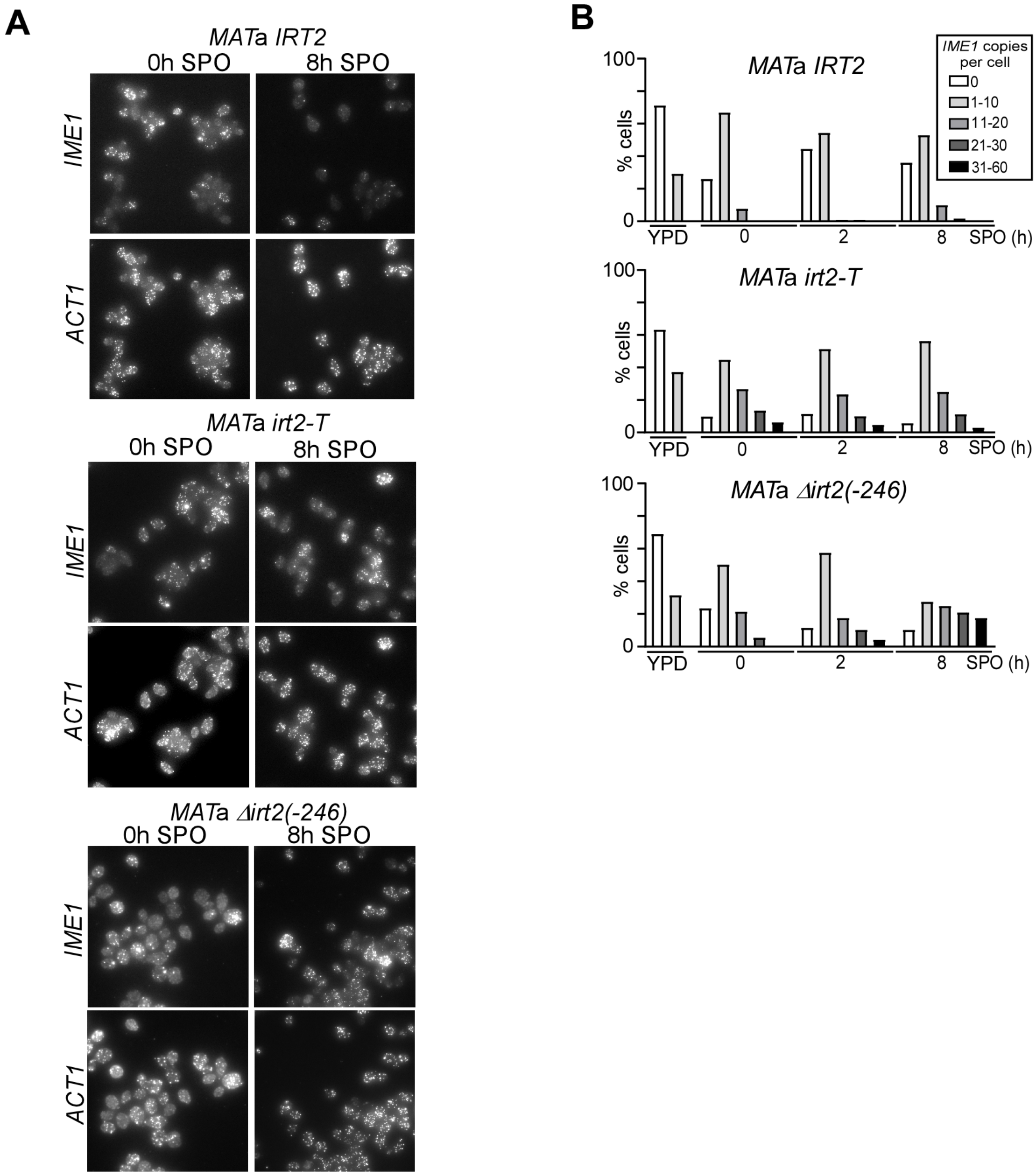

Figure S3: related to Figure 3

***IRT2* prevents entry into gametogenesis in cells with a single mating-type**

A, Representative images of *IME1* and *ACT1* expression in single cells as measured by single-molecule RNA FISH in control *MAT*a (FW1533), *irt2-T* (FW3585) and Δ*irt2*(-246) (FW3580). Cells were grown overnight to saturation in rich medium (YPD, 24 h), shifted to pre-SPO and grown for an addition 16 hours, and subsequently transferred to SPO. Cells were fixed at the indicated time points and hybridized with probes directed against *IME1* and *ACT1*.

B, Distribution of *IME1* expression in single cells. The data described in Figure 3A were binned according to the number of *IME1* copies per cell.

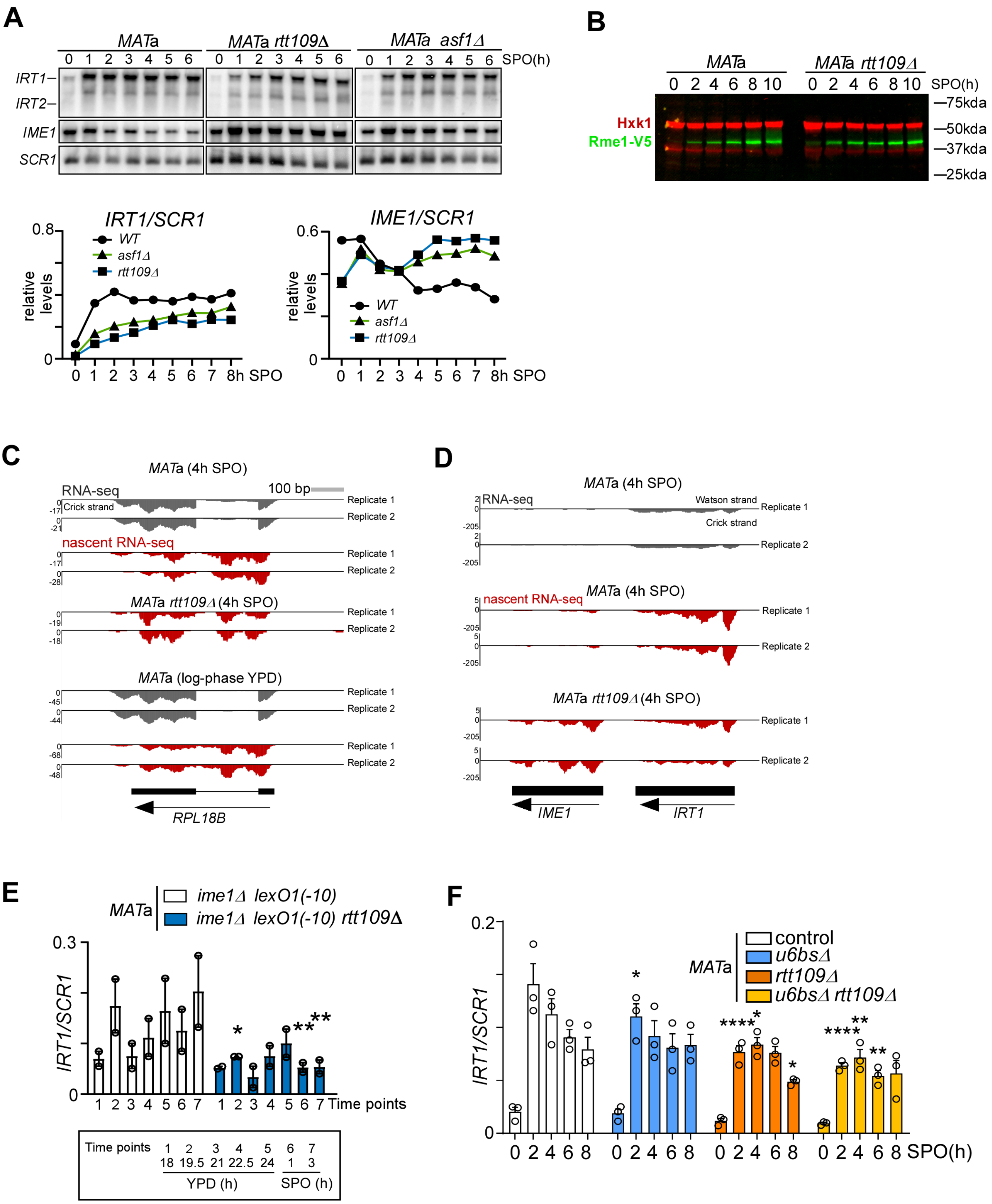
A
MATa
MATa rtt109Δ
MATa asf1Δ
SPO(h)
IRT1
IRT2
IME1
SCR1
IRT1/SCR1
IME1/SCR1
WT
asf1Δ
rtt109Δ
relative levels
8h SPO
B
MATa
MATa rtt109Δ
SPO(h)
Hxk1
Rme1-V5
75kda
50kda
37kda
25kda
C
MATa (4h SPO)
RNA-seq
Crick strand
100 bp
nascent RNA-seq
Replicate 1
Replicate 2
MATa rtt109Δ (4h SPO)
MATa (log-phase YPD)
RPL18B
D
MATa (4h SPO)
RNA-seq
Watson strand
Crick strand
nascent RNA-seq
MATa rtt109Δ (4h SPO)
IME1
IRT1
E
MATa
ime1Δ lexO1(-10)
ime1Δ lexO1(-10) rtt109Δ
IRT1/SCR1
Time points
Time points 1 2 3 4 5 6 7
18 19.5 21 22.5 24 1 3
YPD (h)
SPO (h)
F
MATa
control
u6bsΔ
rtt109Δ
u6bsΔ rtt109Δ
IRT1/SCR1
SPO(h)

Figure S4: related to Figure 4

Rtt109 and Asf1 promote *IRT1* transcription

A, *IRT1*, *IRT2*, and *IME1* expression in *MAT*a control (FW1509), *rtt109*Δ (FW4077) and *asf1Δ* (FW4521) cells as detected by northern blot (top). *SCR1* was used as loading control. Cells were grown in rich medium (YPD, 24 h) to saturation, shifted in pre-SPO (16 h), and subsequently transferred to SPO. Northern blot quantifications of the *IRT1* and *IME1* signals over SCR1 signals, are also presented (bottom).

B, Rme1-V5 control *MAT*a cells (FW4031) and *rtt109*Δ mutant (FW4075), as detected by western blot using anti-V5 (green channel). Hxk1 was used as loading control using anti-Hxk1 (red channel) antibodies. Cells were grown as described in A and samples were taken at the indicated time points.

C, Nascent RNA-seq data for *RPL18B*. Two replicates of RNA-seq and nascent RNA-seq (Pol II associated RNA) are displayed. Cells were harvested in SPO 4 h and during exponential growth (log phase YPD) for *MAT*a control, and SPO 4 h for *MAT*a *rtt109Δ*. Subsequently, Pol II was purified using the Rpb3-FLAG. The values on the y-axes are in Reads Per Million (RPM).

D, Similar to C, except that *IRT1* and *IME1* transcription data are displayed.

E, Northern blot quantification of *IRT1* expression for data shown in Figure 4F. *IRT1* expression was normalised over *SCR1*. n = 2 +/- SEM. * and ** correspond to p-value < 0.05 and <0.005 respectively on a two-way ANOVA followed by a Fisher's LSD test.

F, Northern blot quantification of *IRT1* expression for data shown in Figure 4G. *IRT1* expression was normalized over *SCR1*. n = 3 +/- SEM. *, ** and **** correspond to p-value < 0.05, < 0.005 and < 0.0001 respectively on a two-way ANOVA followed by a Fisher's LSD test.

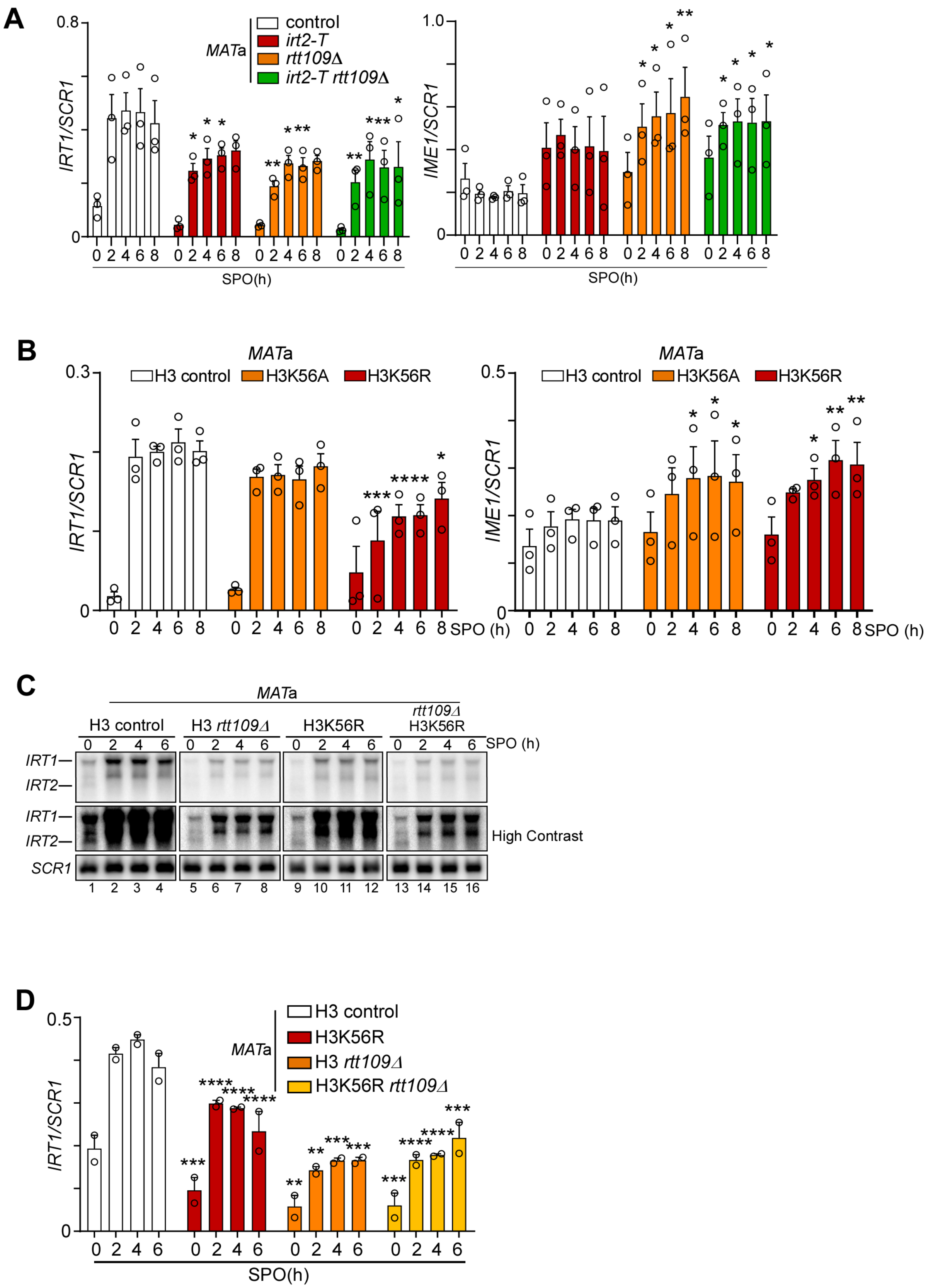
A
0.8
0
IRT1/SCR1
MATa
control
irt2-T
rtt109Δ
irt2-T rtt109Δ
0 2 4 6 8
SPO(h)
1.0
0
IME1/SCR1
B
MATa
0.3
H3 control
H3K56A
H3K56R
0.5
SPO (h)
C
MATa
H3 control
H3 rtt109Δ
H3K56R
rtt109Δ
H3K56R
SPO (h)
IRT1
IRT2
High Contrast
SCR1
1 2 3 4 5 6 7 8 9 10 11 12 13 14 15 16
D
0.5
H3 control
H3K56R
H3 rtt109Δ
H3K56R rtt109Δ
SPO(h)

Figure S5: related to Figure 5

***IRT2* transcription directs Histone H3 lysine 56 acetylation to chromatin locally to activate *IRT1* transcription**

A, Quantifications of *IRT1* and *IME1* expression for data shown in Figure 5A. *IRT1* and *IME1* signals were normalized over *SCR1*. n = 3, +/- SEM. *, ** and *** correspond to p-value < 0.05, < 0.005 and < 0.0005 respectively on a two-way ANOVA followed by a Fisher's LSD test.

B, Quantifications of *IRT1* and *IME1* expression for data shown in Figure 5D. *IRT1* and *IME1* signals were normalized over *SCR1*. n = 3, +/- SEM. *, **, *** and **** correspond to p-value < 0.05, < 0.005, < 0.0005 and < 0.0001 respectively on a two-way ANOVA followed by a Fisher's LSD test.

C, *IRT1* and *IRT2* expression in control *MAT*a H3 (FW5102), H3 *rtt109*Δ (FW6443), *H3K56R* (FW5116) and *rtt109*Δ *H3K56R* double mutant (FW5724) cells as detected by northern blot. *SCR1* was used as loading control. An image presenting an adjusted signal highlighting *IRT2* expression from the northern blot is also included. Cells were grown in rich medium (YPD, 24 h) to saturation, shifted and grown in pre-SPO (16 h), and subsequently transferred to SPO.

D, Quantification of *IRT1* expression as described in C. *IRT1* signals were normalized to *SCR1*. n = 2, mean values and data range are displayed. **, *** and **** correspond to p-value < 0.005, < 0.0005 and < 0.0001 respectively, on a two-way ANOVA followed by a Fisher's LSD test.

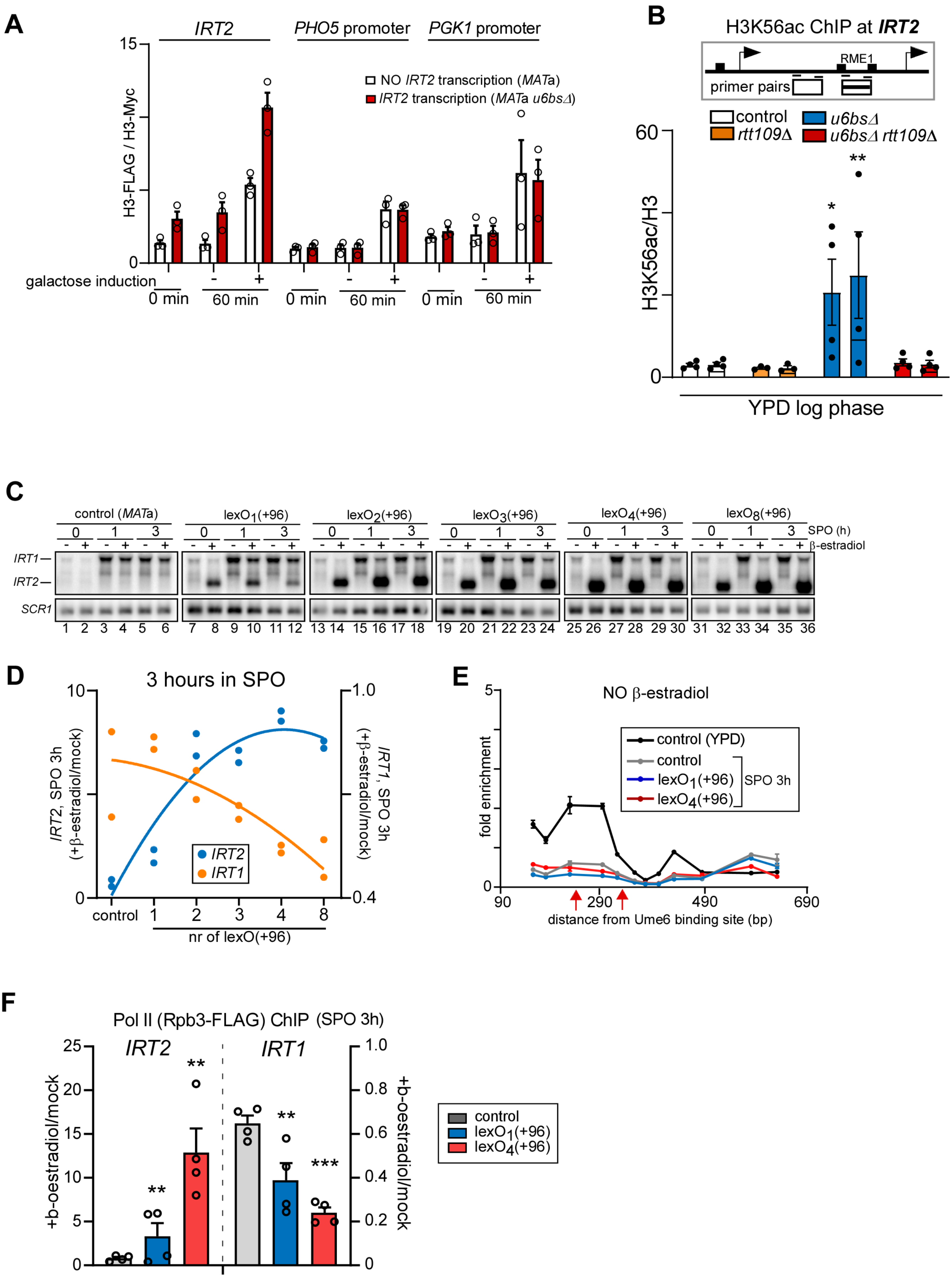
A
IRT2
PHO5 promoter
PGK1 promoter
H3-FLAG / H3-Myc
NO IRT2 transcription (MATa)
IRT2 transcription (MATa u6bsΔ)
galactose induction
0 min
60 min
B
H3K56ac ChIP at IRT2
RME1
primer pairs
control
rtt109Δ
u6bsΔ
u6bsΔ rtt109Δ
H3K56ac/H3
YPD log phase
C
control (MATa)
lexO1(+96)
lexO2(+96)
lexO3(+96)
lexO4(+96)
lexO8(+96)
SPO (h)
β-estradiol
IRT1
IRT2
SCR1
D
3 hours in SPO
IRT2, SPO 3h (+β-estradiol/mock)
IRT1, SPO 3h (+β-estradiol/mock)
IRT2
IRT1
control
nr of lexO(+96)
E
NO β-estradiol
fold enrichment
control (YPD)
control
lexO1(+96)
lexO4(+96)
SPO 3h
distance from Ume6 binding site (bp)
F
Pol II (Rpb3-FLAG) ChIP (SPO 3h)
IRT2
IRT1
+b-oestradiol/mock
control
lexO1(+96)
lexO4(+96)

Figure S6: related to Figure 6

***IRT2* transcription has a level dependent effect on local chromatin and transcription states**

A, Histone exchange at the *IRT2, PHO5* and *PGK1* promoters as measured by ChIP. Strains and growth condition as described in Figure 6A and B. To have constitutive levels of *IRT2* transcription we used the *u6bs*Δ mutation (FW7880), while the wild-type cells (FW7853), display no *IRT2* transcription in rich medium (YP). qPCRs primer pairs for the *PHO5* and *PGK1* promoters were used for the analysis. The signal for each histone H3 ChIP (Myc-H3 and FLAG-H3) were normalized to a telomere locus, and ratio for n = 3 +/- SEM are displayed. The start (0 min) and end points (60 min) of galactose induced and non-induced cells are displayed.

B, Histone H3 lysine 56 acetylation (H3K56ac) levels in the *IRT1* promoter as measured by ChIP in control (*MAT*a, FW1509), rtt109 (FW4077), *u6bsΔ* (FW2438) and *u6bsΔ rtt109Δ* (FW5225) cells. Samples were taken during exponential growth (YPD log phase). H3K56ac ChIP signals were normalized to histone H3. n = 4 +/- SEM for all condition except n = 3 for *rtt109*Δ. * and ** correspond to a p-value < 0.05 and < 0.005 respectively on a two-way ANOVA followed by Fisher's LSD test performed on each primer pair individually. qPCRs primer pairs are presented on the top scheme.

C, *IRT1* and *IRT2* expression in control *MAT*a (FW6560), lexO$_1$(+96) (FW6594), lexO$_2$(+96) (FW6599), lexO$_3$(+96) (FW6607), lexO$_4$(+96) (FW6611), and lexO$_8$(+96) (FW6619) cells as detected by northern blot. These cells also expressed LexA-ER. Cells were grown in rich medium (YPD) to saturation (24 h), shifted and grown in pre-SPO for 15 h, and cells either mock treated or treated with β-estradiol (25 nM) for an additional 1 hour. Subsequently cells were transferred to SPO and the treatments were continued. Samples were taken at the indicated time points. *SCR1* was used as loading control.

D, Quantification of *IRT1* expression as described in C. Signals were normalized to *SCR1* and presented as a ratio of β-estradiol over mock treatment for the 3 h time points.

E, Chromatin structure at the *IRT1* promoter in the presence of distinct levels of *IRT2* transcription. *MAT*a control cells (FW6560) or cells harbouring 1 or 4 lexO(+96) sites (FW6594 or FW6611) were grown as described in C and the mock treated condition is displayed. β-estradiol treated condition is displayed in figure 6F. Cells grown in rich nutrient conditions (YPD) till exponential growth were included in the analysis. Chromatin was digested with micrococcal nuclease (MNase) followed by qPCR using scanning primer pairs in *IRT2*. The red arrows indicate the position of the Rme1 binding sites. The signals were normalized over a telomeric region from chromosome VI. n = 3 +/- SEM.

F, Pol II binding at *IRT1* as measured by ChIP. Similar to E, except that cells also contained Rpb3-FLAG, which was used for the immunoprecipitation step (FW8808, FW8812 and FW8827). Cells were grown as described in C, and samples for ChIP were taken at 3 h in SPO. Signals were normalized over the silent mating type locus *HMR*. The ratio β-estradiol treated over mock treated are presented. n = 4 +/- SEM. ** and *** correspond to p-value < 0.005 and < 0.0005 respectively on a one-way ANOVA followed by a Fisher's LSD test.

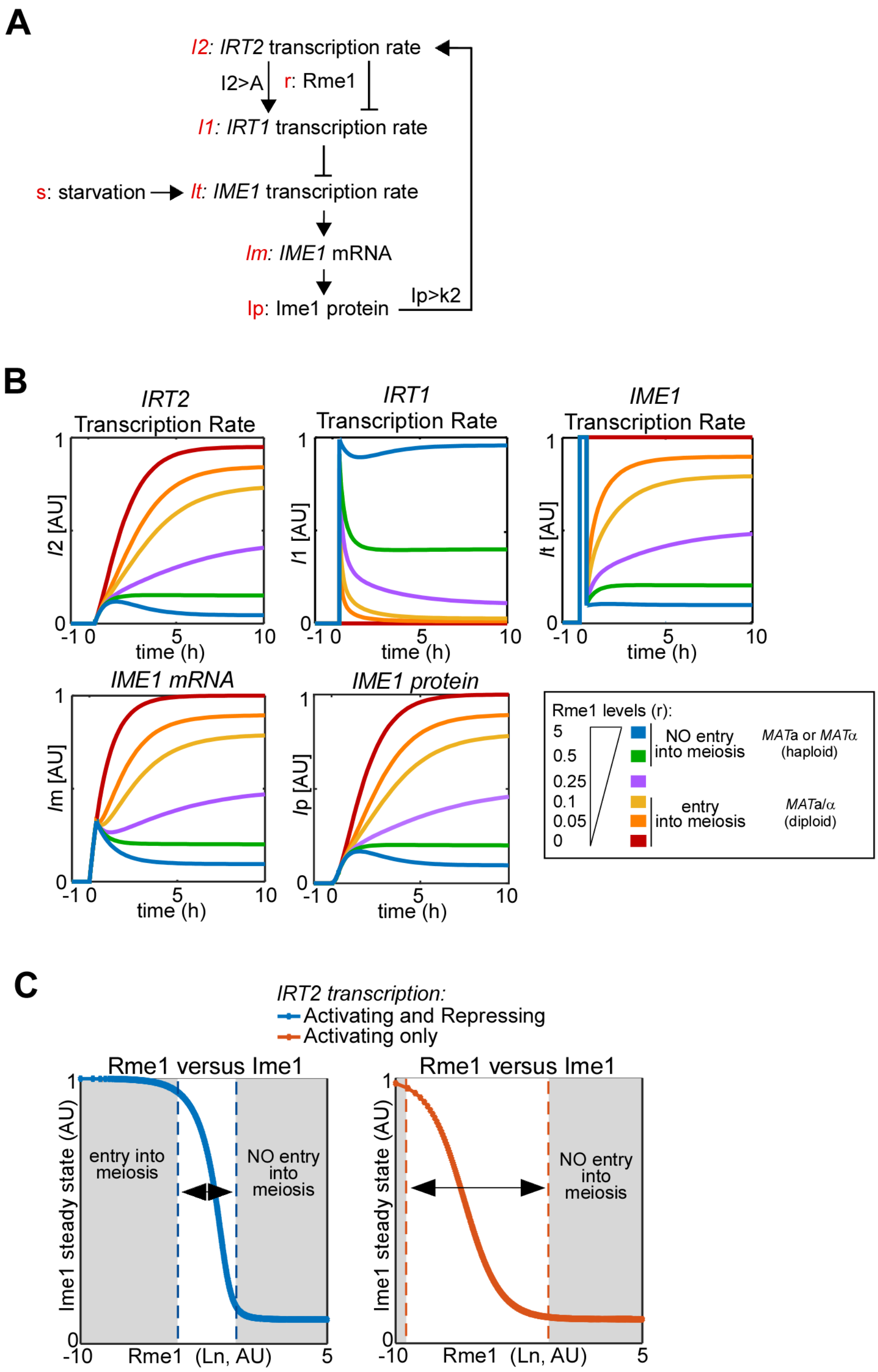
A
I2: IRT2 transcription rate
I2>A
r: Rme1
I1: IRT1 transcription rate
s: starvation
It: IME1 transcription rate
Im: IME1 mRNA
Ip: Ime1 protein
Ip>k2
B
IRT2 Transcription Rate
I2 [AU]
IRT1 Transcription Rate
I1 [AU]
IME1 Transcription Rate
It [AU]
IME1 mRNA
Im [AU]
IME1 protein
Ip [AU]
time (h)
Rme1 levels (r):
5
0.5
0.25
0.1
0.05
0
NO entry into meiosis
MATa or MATα (haploid)
entry into meiosis
MATa/α (diploid)
C
IRT2 transcription:
Activating and Repressing
Activating only
Rme1 versus Ime1
Ime1 steady state (AU)
entry into meiosis
NO entry into meiosis
Rme1 (Ln, AU)

Figure S7: related to Figure 7

Modelling of cell-type specific control of Ime1 expression involving noncoding transcription

A, Model for Ime1 regulation by *IRT1*, *IRT2*, and *IME1* (see Methods section for details).

B, Simulation of *IRT1*, *IRT2*, and *IME1* transcription rates, *IME1* mRNA and Ime1 protein levels in the presence of different concentrations of Rme1. Different Rme1 concentrations are indicated (r= 0, 0.05, 0.1, 0.25, 0.5 and 5, arbitrary units), leading to either no entry into meiosis in *MAT*a or *MAT*α (haploid), or entry in meiosis in *MAT*a/α (diploid) cells.

C, Dose response relationship between Ime1 steady state levels and Rme1 concentration, simulated for WT *IRT2* (activating and repressing levels) and *IRT2* activating levels only, which are simulated by setting I2=A. Indicated are the levels of Rme1 corresponding to either no entry into meiosis in haploid cells, or entry in meiosis into diploid cells.

Table S1. Screen for mutants that affect *IRT1* expression. Related to Figure 4.

gene delete	*IRT1*	*IME1*	*RME1*
RME1	---	+++	none
ASF1	-	+	NT
CAC2	0	NT	NT
CTK1	---	+	NT
ELP4	0	0	NT
ELP6	0	0	NT
GCN5	---	+++	---
HIR1	0	NT	NT
HST3	0	0	NT
HST4	0	0	NT
JHD2	0	0	NT
NAP1	0	0	NT
RLF2	+	NT	NT
RPB9	--	++	NT
RTR1	0	0	NT
RTT106	0	NT	NT
RTT109	--	++	0
SET1	0	0	NT
SET3	0	+	NT
SNF5	---	NT	---
SUB1	0	0	NT
SWC3	0	0	NT
SWC5	0	0	NT
SWR1	0	NT	0
VPS75	0	0	NT
HOS2	0	0	NT

NT: not tested
+/-: difference with WT
0: no difference with WT

Table S3: Oligo nucleotide sequences used. Related to STAR methods.

FW463	caacgcctccgataatgtatatg	IME1 northern probe
FW464	acgtcgaaggcaatttctaatg	IME1 northern probe
FW481	atttttagcgactgccgaaa	IRT2 qPCR (Rme1 ChIP)
FW482	atgcaacgcctacttgtttt	IRT2 qPCR (Rme1 ChIP)
FW1895	aaaatgaaaggcagaagatg	IRT2 qPCR (H3 ChIP oligo pair 1)
FW1896	ctggtatggtattgtaagga	IRT2 qPCR (H3 ChIP oligo pair 1)
FW1899	atcatgctgttctttccgcc	IRT2 qPCR (H3 ChIP oligo pair 2)
FW1900	cccacccttcttttattgag	IRT2 qPCR (H3 ChIP oligo pair 2)
FW1905	tcaagaagtccactaaatgg	IRT2 qPCR (H3 ChIP oligo pair 3)
FW1906	acaattttatgcttttgagg	IRT2 qPCR (H3 ChIP oligo pair 3)
FW43	acgatccccgtccaagttatg	HMR qPCR (Rme1 and Pol II ChIP)
FW50	cttcaaaggagtcttaatttccctg	HMR qPCR (Rme1 and Pol II ChIP)
FW490	tgatatgtatgggttaaaaaggatg	IRT1-IRT2 dual northern probe
FW540	ggcagttcaaaggcttttctta	IRT1-IRT2 dual northern probe, ChIP and RT-qPCR, IRT1
FW489	atgcaacgcctacttgtttt	IRT2 northern probe
FW493	gatggagggttggcataaaa	IRT2 northern probe
FW1555	gctgcagaacttggttcataca	IME1 northern probe
FW464	acgtcgaaggcaatttctaatg	IME1 northern probe
FW1841	gaagtgtcccggctataataaa	SCR1 northern probe
FW1842	gacgctggataaaactcccc	SCR1 northern probe
FW2448	tccgaacgctattccagaaagt	ChIP/MNase normalisation (Telomere locus)
FW2449	ccataatgcctcctatatttagcctttt	ChIP/MNase normalisation (Telomere locus)
FW2450	catatattatctatatcatgc	MNase qPCR
FW2451	ccgtgagggaatacattaat	MNase qPCR
FW1899	atcatgctgttctttccgcc	MNase qPCR
FW1900	cccacccttcttttattgag	MNase qPCR
FW1901	attaatgtattccctcacgg	MNase qPCR
FW1902	cttcttgaggttcttttgacatc	MNase qPCR
FW1087	ggatgtcaaaagaacctcaaga	MNase qPCR
FW1088	tttcggcagtcgctaaaaat	MNase qPCR
FW1905	tcaagaagtccactaaatgg	MNase qPCR
FW1906	acaattttatgcttttgagg	MNase qPCR
FW1907	gccgaaaacgtacggctaac	MNase qPCR
FW488	atttttagcgactgccgaaa	MNase qPCR
FW487	tttttgttatctgcctgaaacg	MNase qPCR
FW1908	gctcactttttttcctaccca	MNase qPCR

FW2452	gctgtacctcaaaagcataa	MNase qPCR
FW2453	tccagaaacggtttcttatat	MNase qPCR
FW1909	aaaacaagtaggcgttgcat	MNase qPCR
FW1910	accctatttcttcacgaggg	MNase qPCR
FW1030	gagcgccaacactatataag	MNase qPCR
FW1031	caaattctttaaactaagcgc	MNase qPCR
FW1913	gcgcttagtttaaagaatttg	MNase qPCR
FW1914	cctttgttttctctttatcccc	MNase qPCR
FW1915	cttgattattggcattccgc	MNase qPCR
FW1916	ttgctcggaggtactagtca	MNase qPCR
FW539	gggtcttaaatacgcagggaat	ChIP and RT- qPCR, IRT1
FW106	gtaccaccatgttcccaggtatt	RT- qPCR, ACT1 control
FW107	caagatagaaccaccaatccaga	RT- qPCR, ACT1 control

Table S4: Plasmids. Related to STAR methods.

pFW7	pRS306-MATa-URA3	(van Werven et al., 2012)
pFW244	pRS304 MATa-TRP1	This study
pFW355	pRS304-GPDpr-CRE-EBD78-CYC1t	Gift Celine Bouchoux
pFW211	pUG6-Myc-C-Avitag	(van Werven and Timmers, 2006)
pFW502	pDM9-HHT2-HHF2-URA3	(Sommermeyer et al., 2013)
pFW503	pDM9-H3K56A-HFF1-URA3	This study
pFW504	pDM9-H3K56R-HFF1-URA3	This study
pFW643	pUB921_p1LexOCYC1-3V5	Gift Elçin Ünal (Chia et al., 2017)
pFW644	pUB922_p2LexOCYC1-3V5	Gift Elçin Ünal (Chia et al., 2017)
pFW645	pUB923_p3LexOCYC1-3V5	Gift Elçin Ünal (Chia et al., 2017)
pFW646	pUB924_p4LexOCYC1-3V5	Gift Elçin Ünal
pFW647	pUB925_p8LexOCYC1-3V5	Gift Elçin Ünal (Chia et al., 2017)

Data S1. Compilation of uncropped northern blot scans used for the figures throughout the manuscript. Related to Figures 1, 2, 4, 5, S1, S2, S4, S5, and S6.

Figure 1B

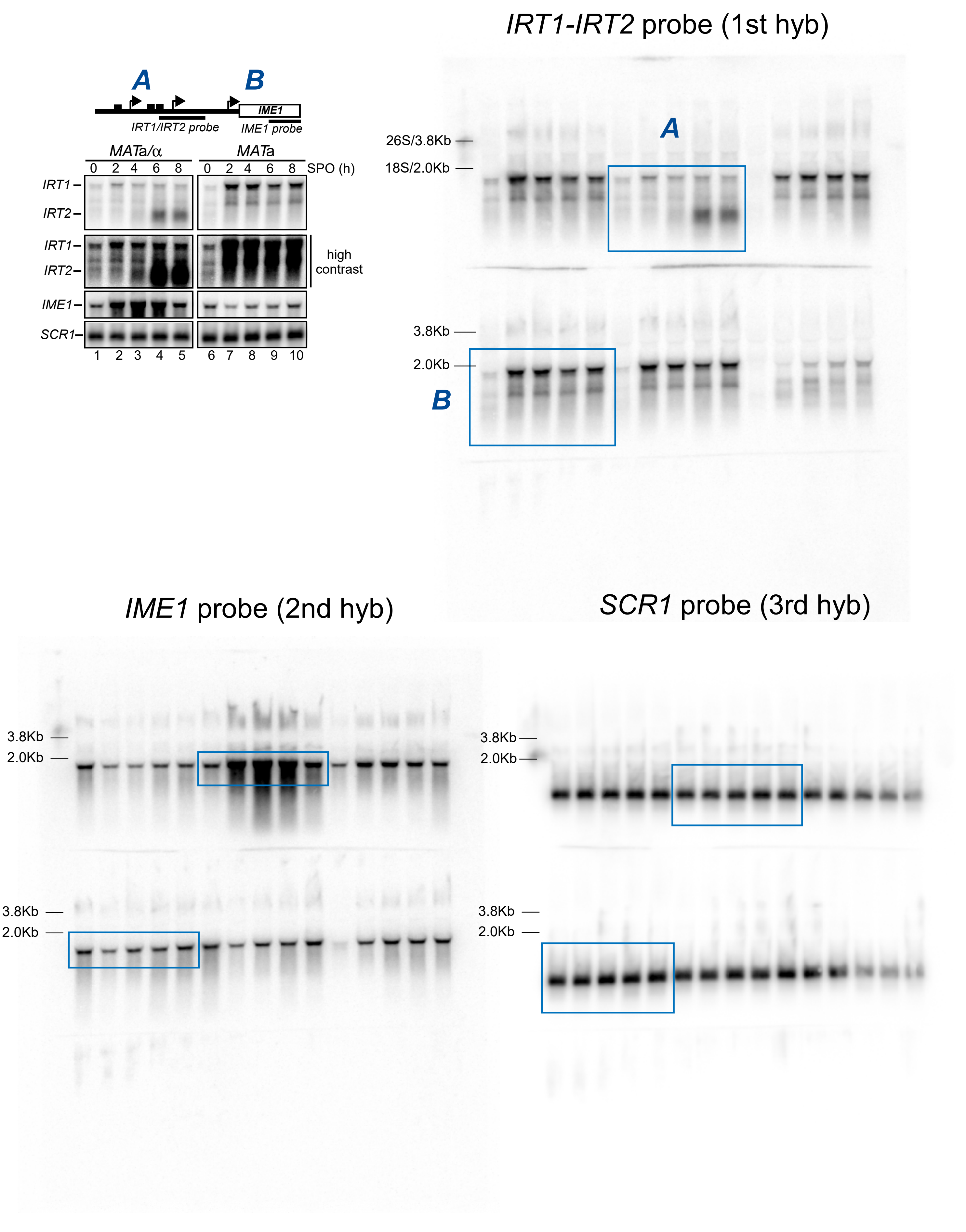
IRT1-IRT2 probe (1st hyb)
IME1 probe (2nd hyb)
SCR1 probe (3rd hyb)
A
B
IME1
IRT1/IRT2 probe
IME1 probe
MATa/α
MATa
0 2 4 6 8
0 2 4 6 8
SPO (h)
IRT1
IRT2
IRT1
IRT2
high contrast
IME1
SCR1
1 2 3 4 5 6 7 8 9 10
26S/3.8Kb
18S/2.0Kb
A
3.8Kb
2.0Kb
B
3.8Kb
2.0Kb
3.8Kb
2.0Kb
3.8Kb
2.0Kb
3.8Kb
2.0Kb

Figure 1D

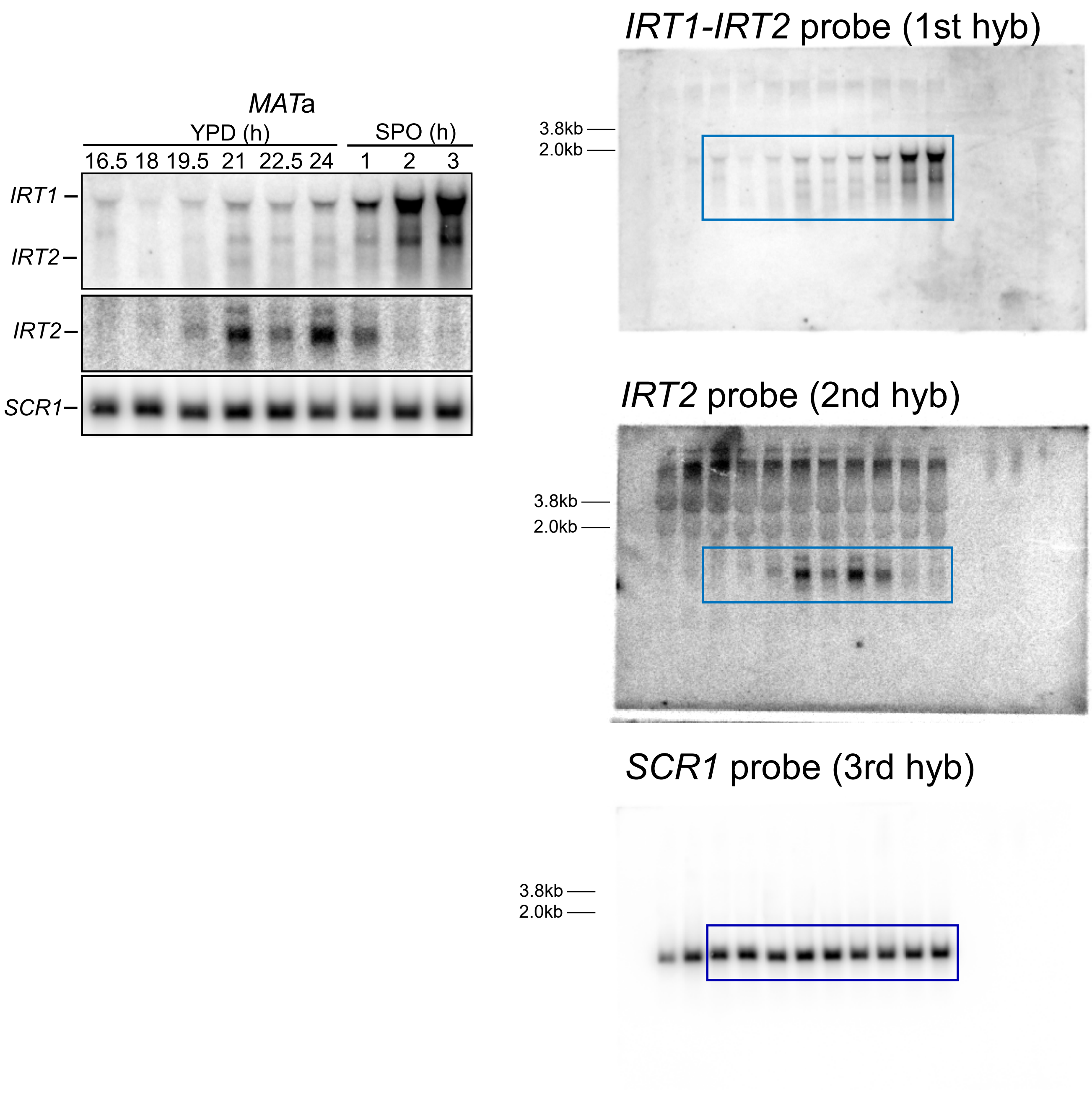
MATa
YPD (h)
SPO (h)
16.5 18 19.5 21 22.5 24 1 2 3
IRT1
IRT2
IRT2
SCR1
IRT1-IRT2 probe (1st hyb)
3.8kb
2.0kb
IRT2 probe (2nd hyb)
3.8kb
2.0kb
SCR1 probe (3rd hyb)
3.8kb
2.0kb

Figure 1E and Figure S1E

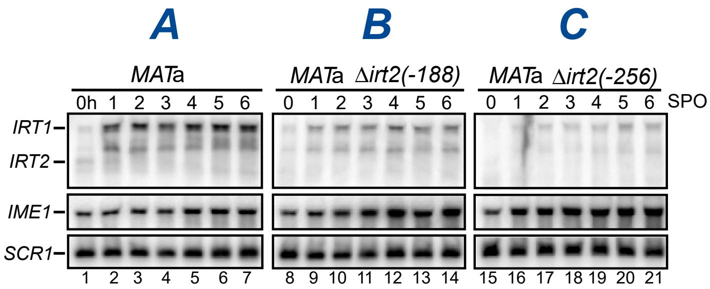

IRT1-IRT2 probe (1st hyb)

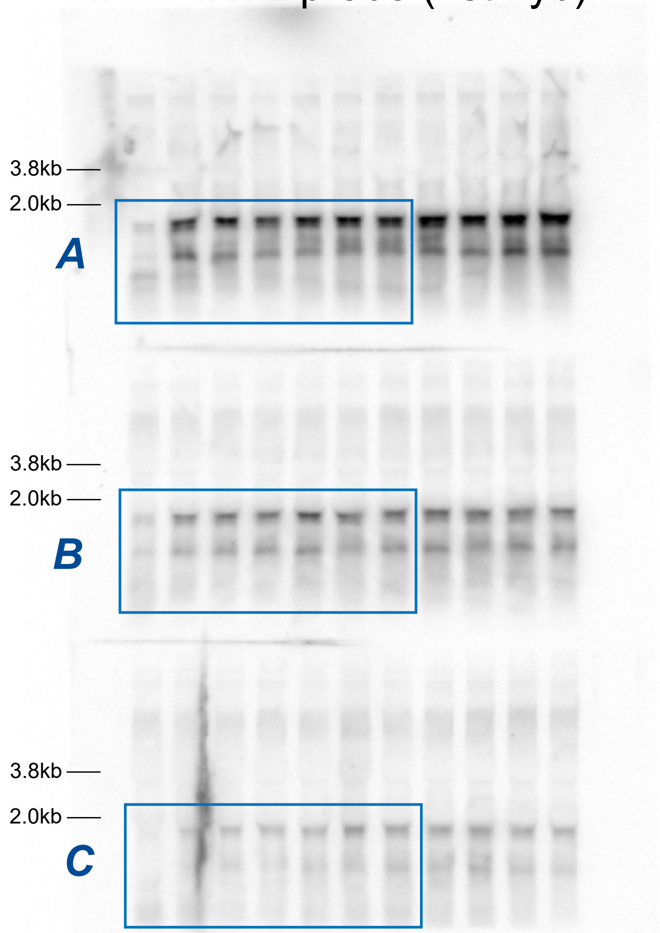

SCR1 probe (3rd hyb)

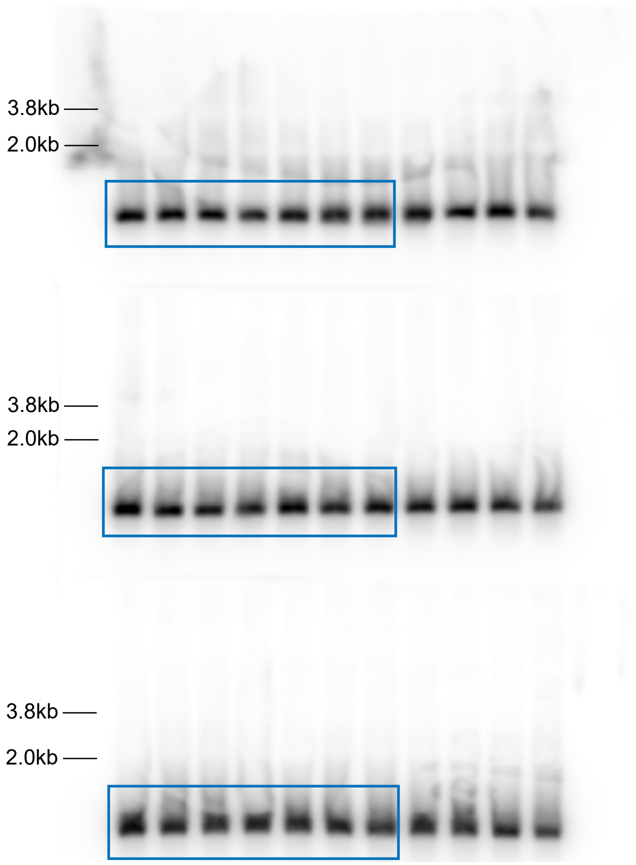

IME1 probe (2nd hyb)

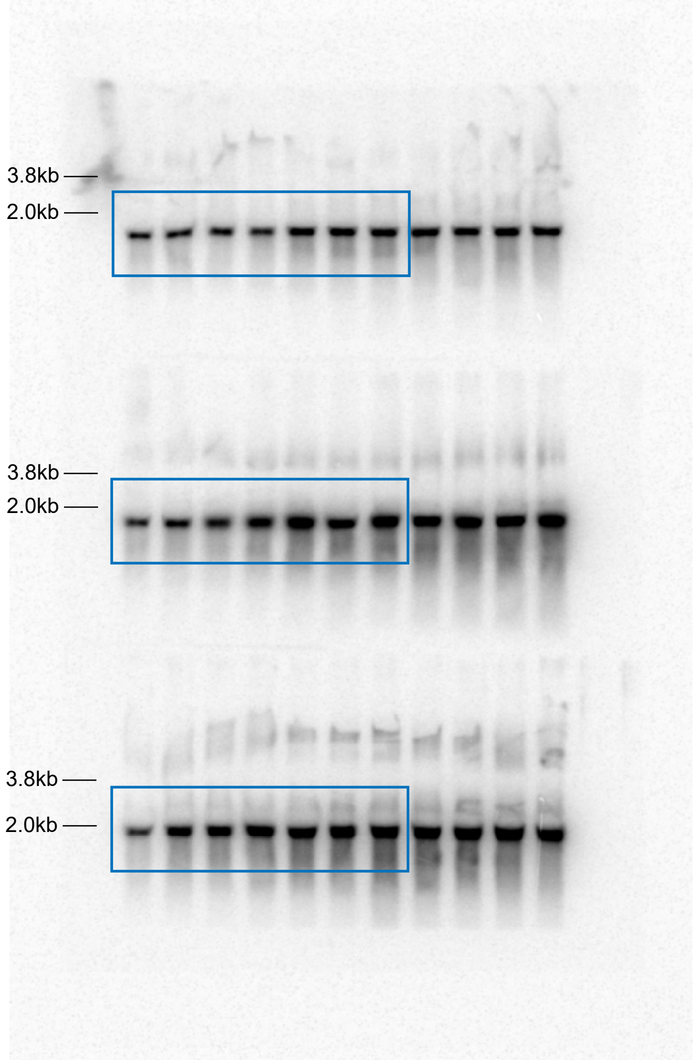

Figure 2B

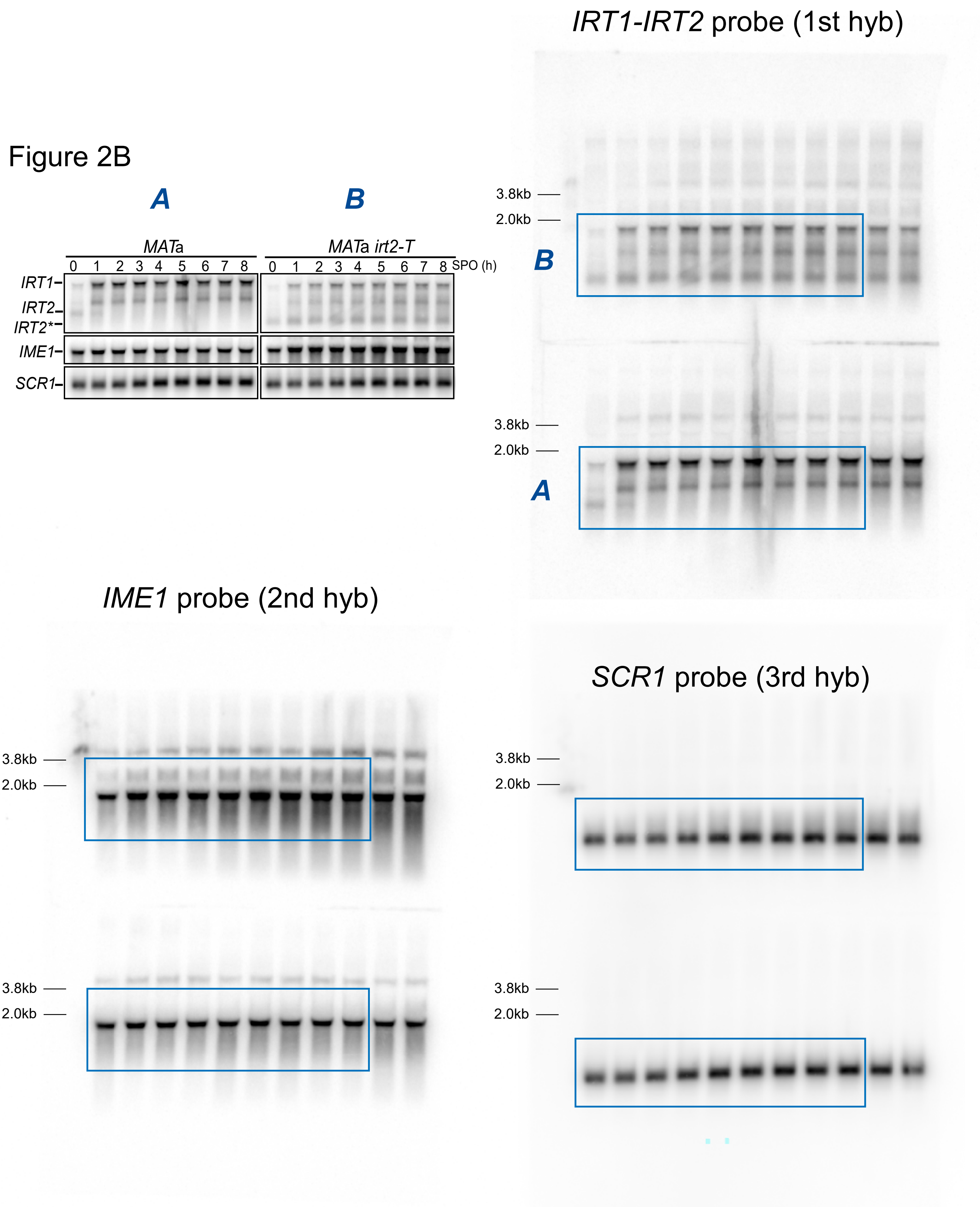

A
B
MATa
MATa irt2-T
0 1 2 3 4 5 6 7 8
0 1 2 3 4 5 6 7 8 SPO (h)
IRT1
IRT2
IRT2*
IME1
SCR1
IRT1-IRT2 probe (1st hyb)
3.8kb
2.0kb
B
3.8kb
2.0kb
A
IME1 probe (2nd hyb)
3.8kb
2.0kb
3.8kb
2.0kb
SCR1 probe (3rd hyb)
3.8kb
2.0kb
3.8kb
2.0kb

Figure 2F

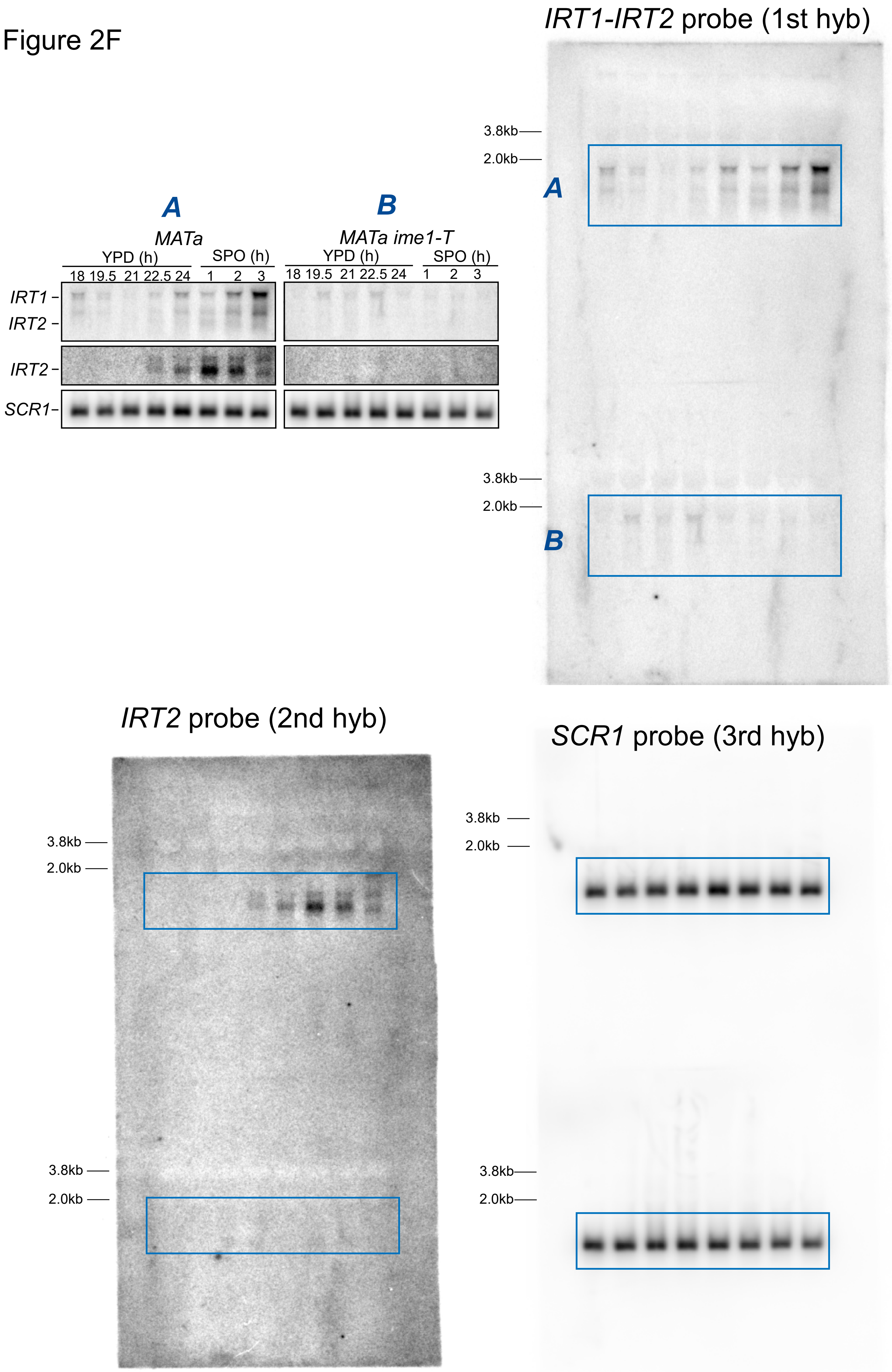
A
B
MATa
MATa ime1-T
YPD (h)
SPO (h)
18 19.5 21 22.5 24 1 2 3
IRT1
IRT2
IRT2
SCR1
IRT1-IRT2 probe (1st hyb)
3.8kb
2.0kb
A
B
IRT2 probe (2nd hyb)
SCR1 probe (3rd hyb)

Figure 2H

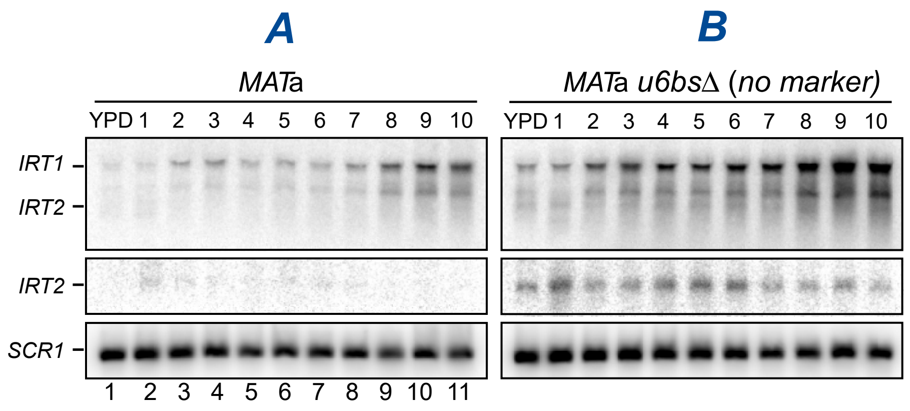
A
B
MATa
MATa u6bsΔ (no marker)
YPD 1 2 3 4 5 6 7 8 9 10
YPD 1 2 3 4 5 6 7 8 9 10
IRT1
IRT2
IRT2
SCR1
1 2 3 4 5 6 7 8 9 10 11

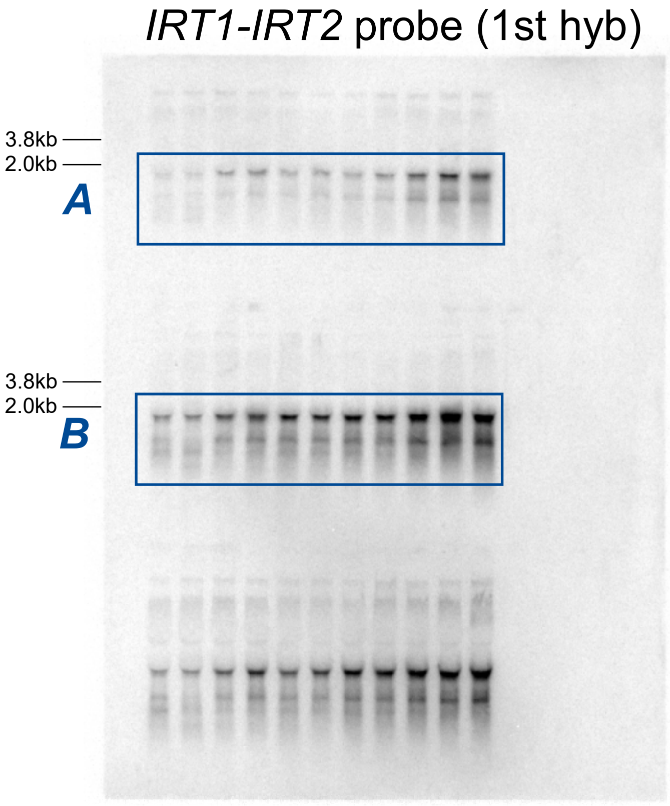
IRT1-IRT2 probe (1st hyb)
3.8kb
2.0kb
A
3.8kb
2.0kb
B

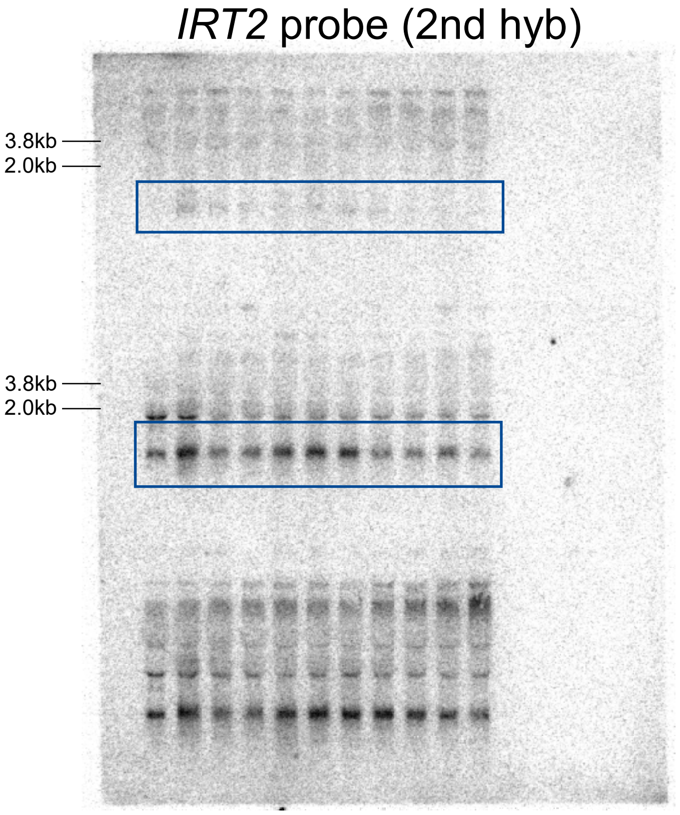
IRT2 probe (2nd hyb)
3.8kb
2.0kb
3.8kb
2.0kb

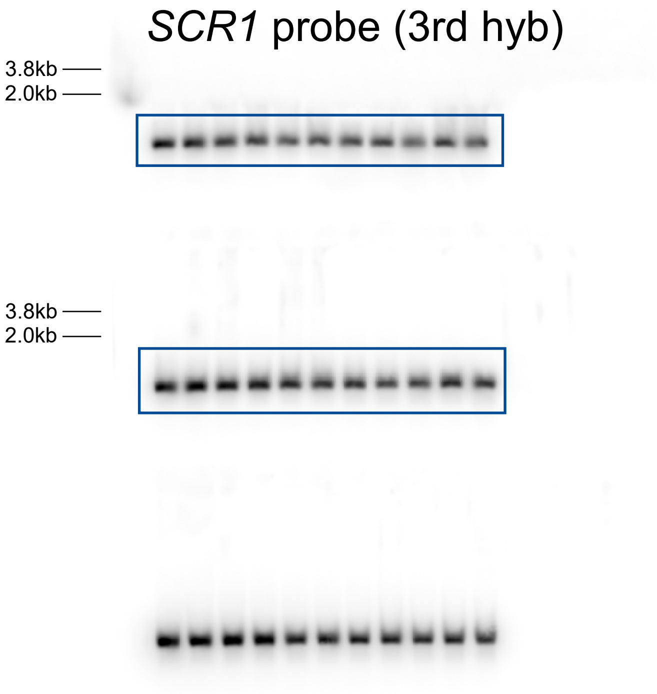
SCR1 probe (3rd hyb)
3.8kb
2.0kb
3.8kb
2.0kb

Figure 2I

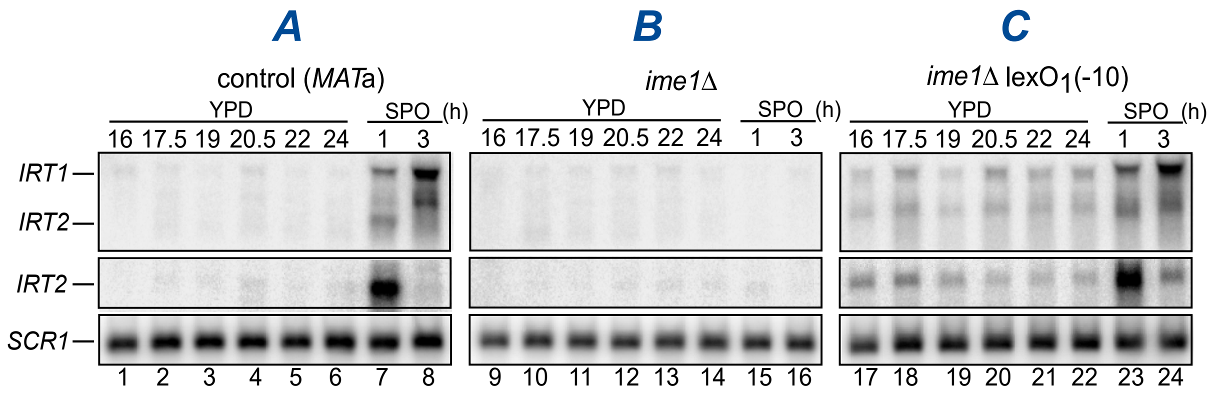

IRT1-IRT2 probe (1st hyb)

IRT2 probe (2nd hyb)

SCR1 probe (3rd hyb)

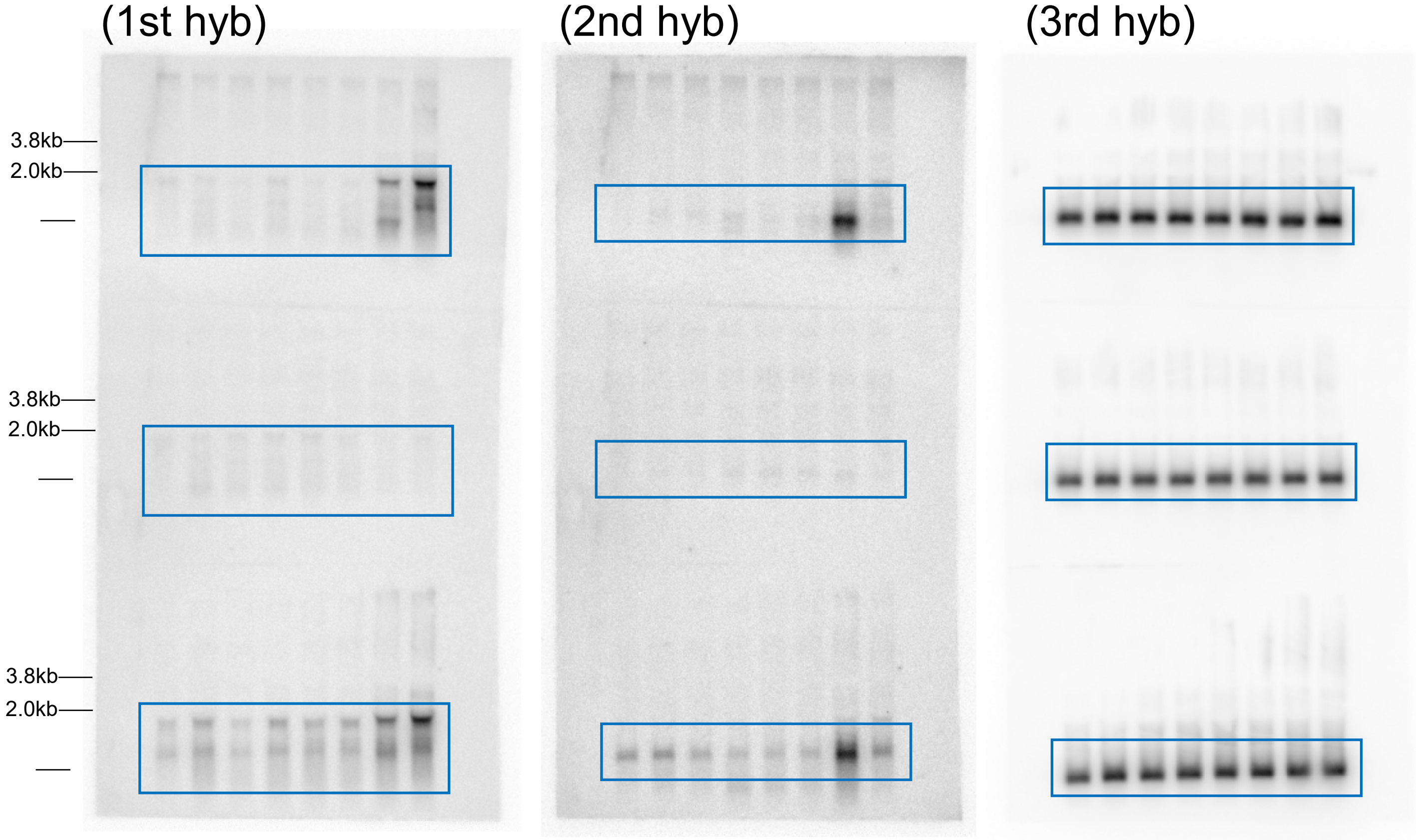

Figure 4B (panels A and B) and Figure S4A (panels A and C)

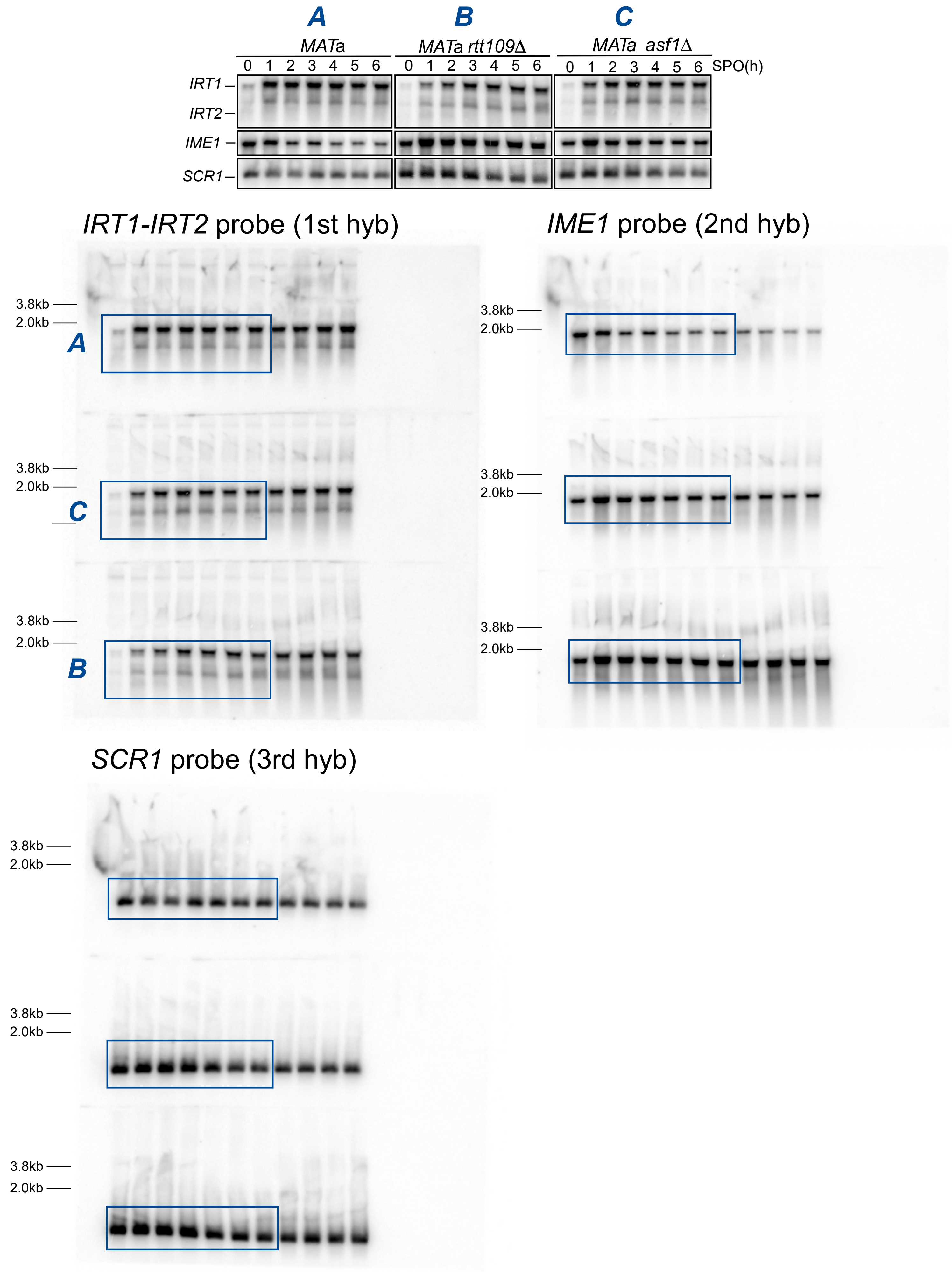

Figure 4F

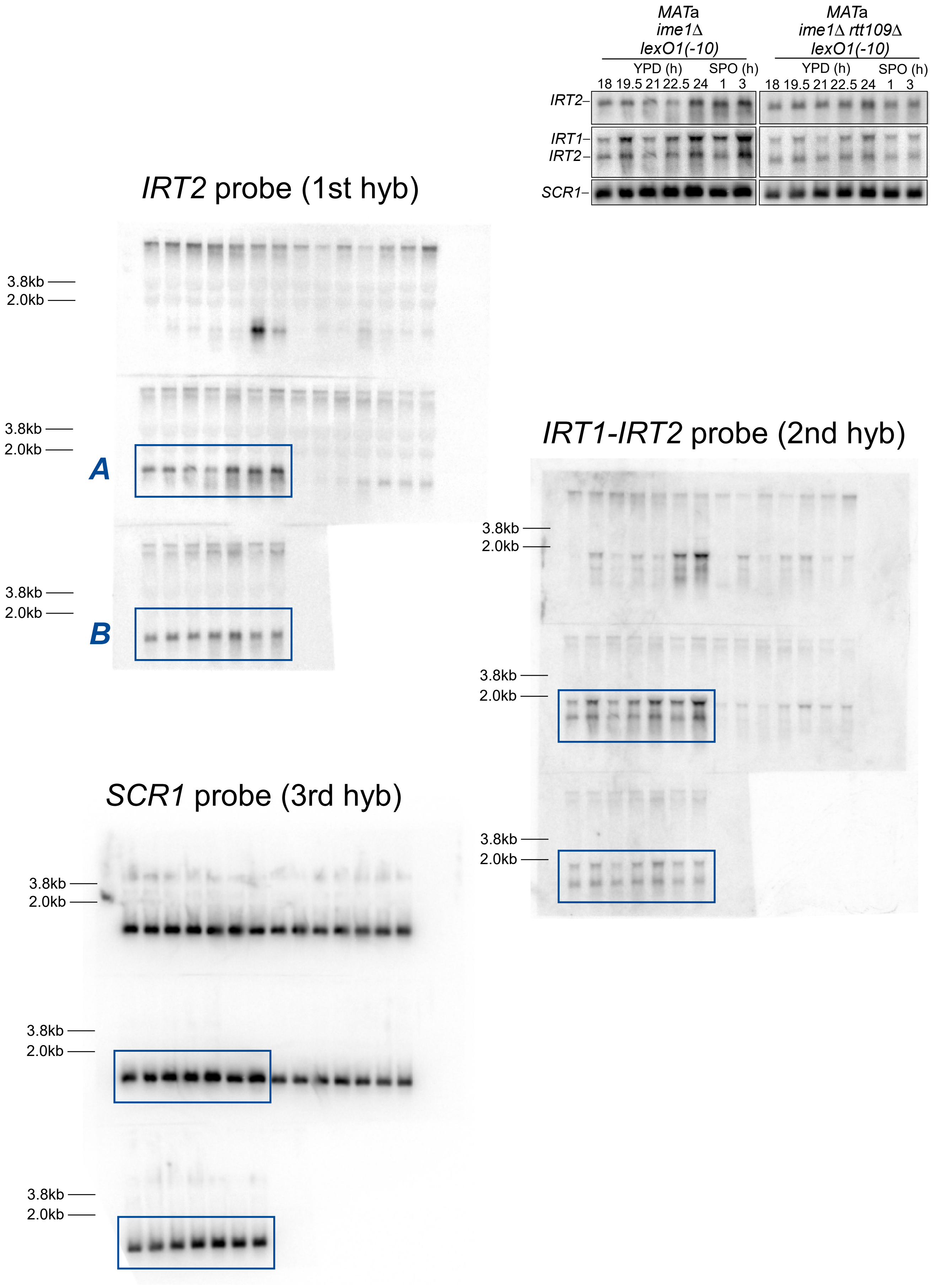
MATa
ime1Δ
lexO1(-10)
MATa
ime1Δ rtt109Δ
lexO1(-10)
YPD (h)
SPO (h)
YPD (h)
SPO (h)
18 19.5 21 22.5 24 1 3
18 19.5 21 22.5 24 1 3
IRT2–
IRT1–
IRT2–
SCR1–
IRT2 probe (1st hyb)
3.8kb
2.0kb
3.8kb
2.0kb
A
3.8kb
2.0kb
B
IRT1-IRT2 probe (2nd hyb)
3.8kb
2.0kb
3.8kb
2.0kb
3.8kb
2.0kb
SCR1 probe (3rd hyb)
3.8kb
2.0kb
3.8kb
2.0kb
3.8kb
2.0kb

Figure 4G

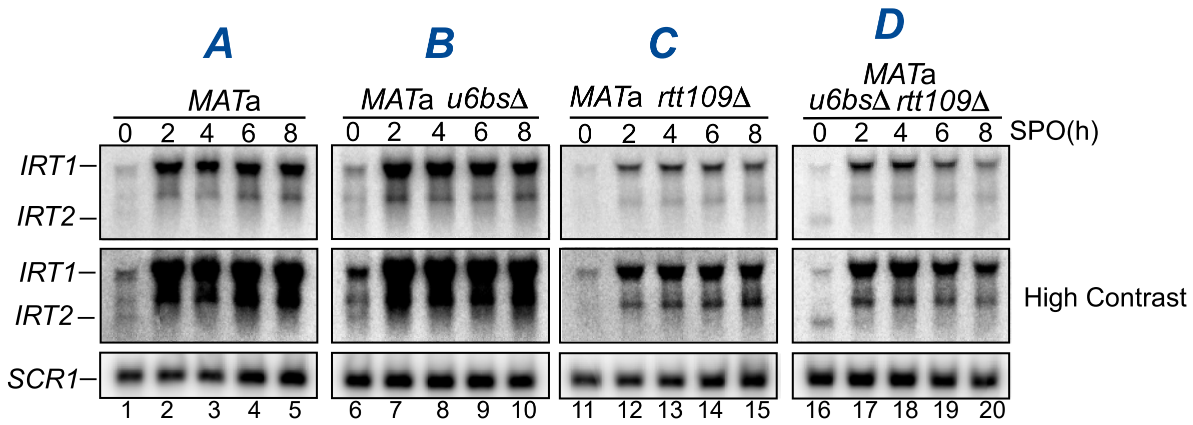
A
B
C
D
MATa
MATa u6bsΔ
MATa rtt109Δ
MATa u6bsΔ rtt109Δ
0 2 4 6 8
SPO(h)
IRT1
IRT2
High Contrast
SCR1
1 2 3 4 5 6 7 8 9 10 11 12 13 14 15 16 17 18 19 20

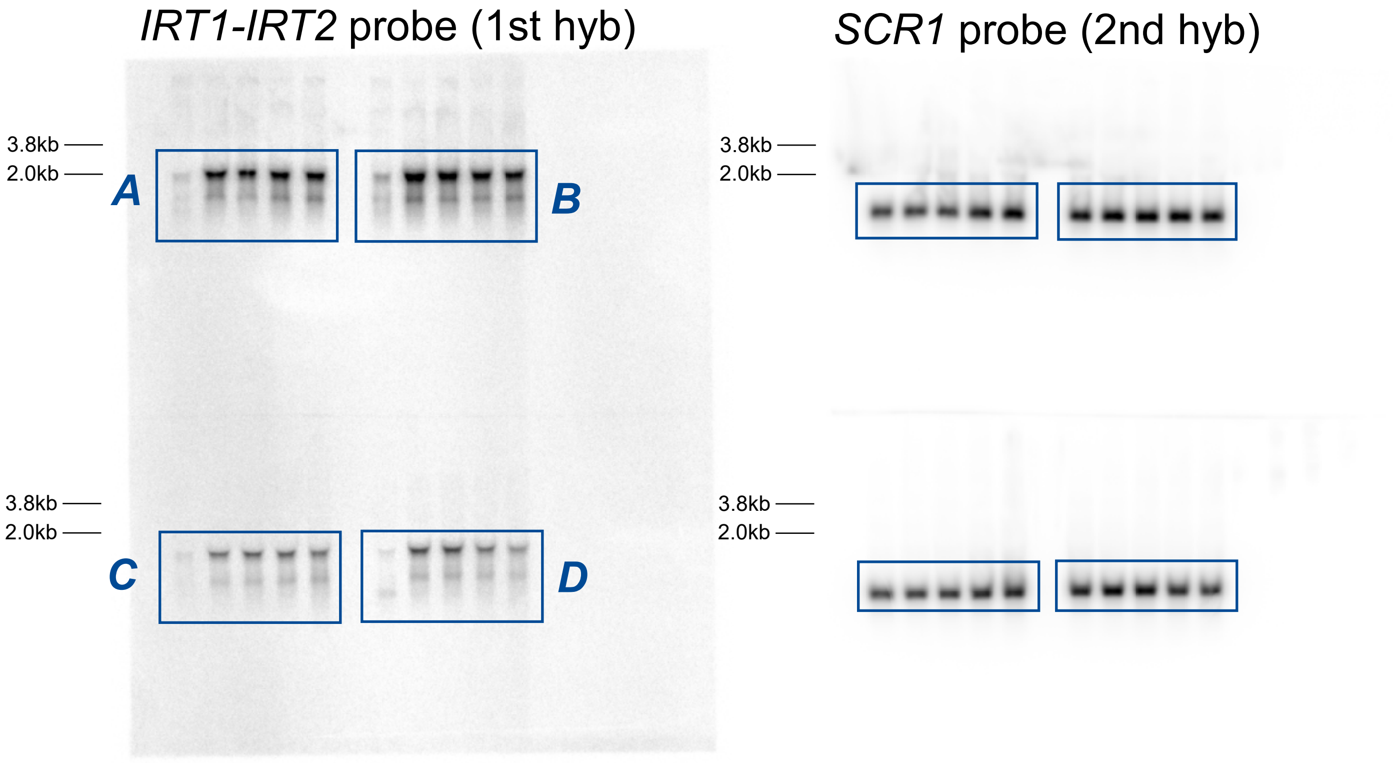
IRT1-IRT2 probe (1st hyb)
SCR1 probe (2nd hyb)
3.8kb
2.0kb
A
B
C
D

Figure 5A

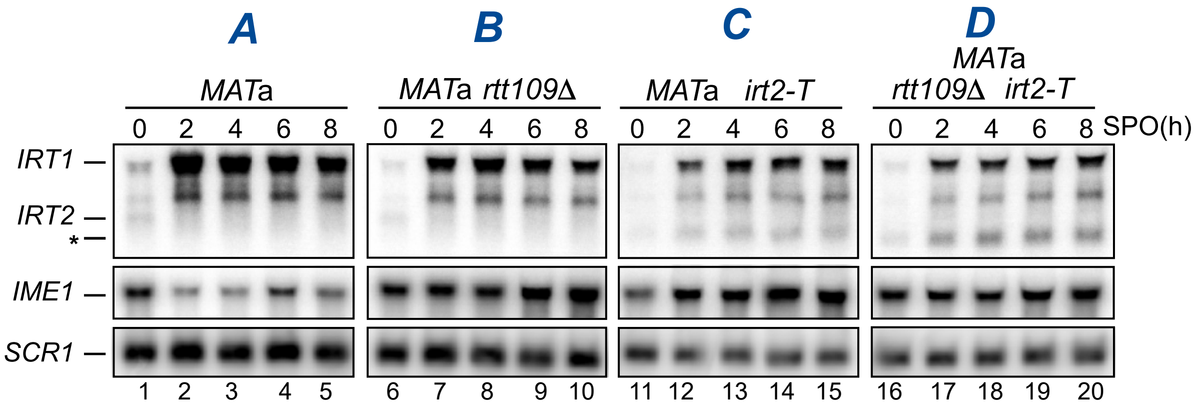
A
B
C
D
MATa
MATa rtt109Δ
MATa irt2-T
MATa
rtt109Δ irt2-T
0 2 4 6 8
SPO(h)
IRT1
IRT2
*
IME1
SCR1
1 2 3 4 5 6 7 8 9 10 11 12 13 14 15 16 17 18 19 20

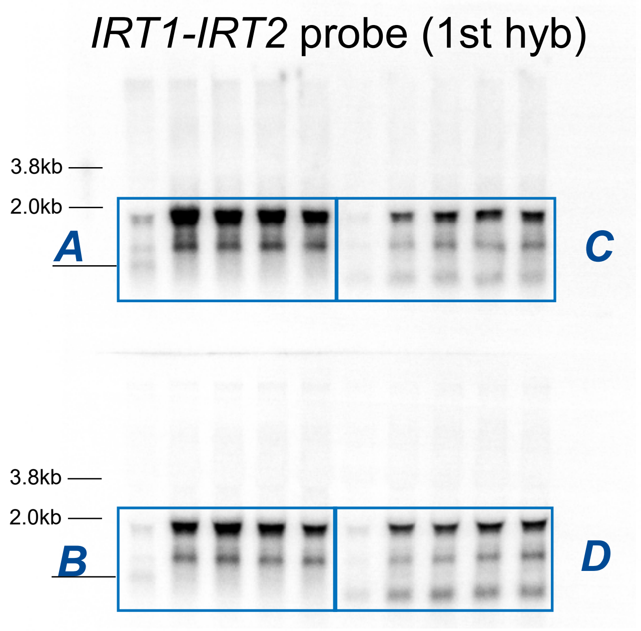
IRT1-IRT2 probe (1st hyb)
3.8kb
2.0kb
A
C
3.8kb
2.0kb
B
D

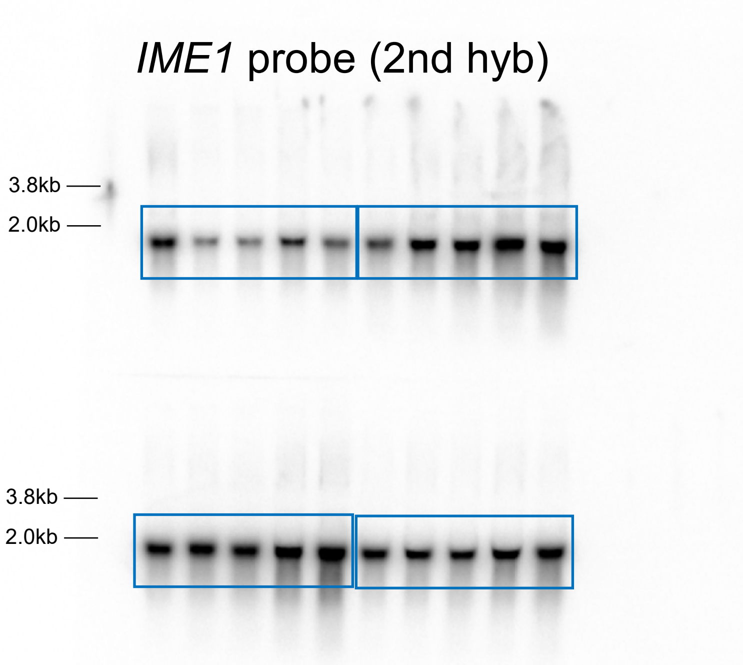
IME1 probe (2nd hyb)
3.8kb
2.0kb
3.8kb
2.0kb

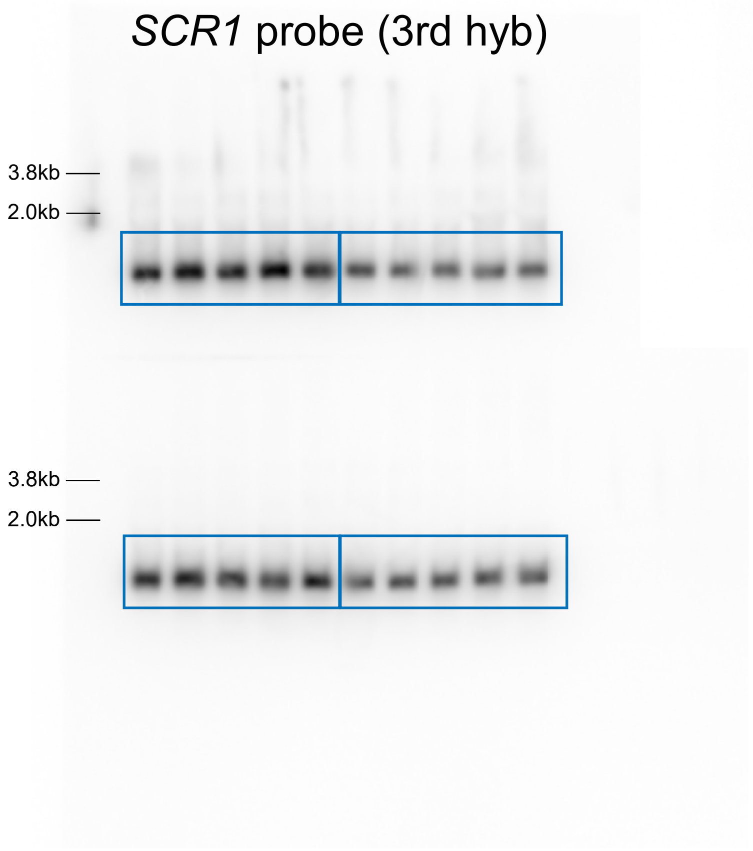
SCR1 probe (3rd hyb)
3.8kb
2.0kb
3.8kb
2.0kb

Figure 5D

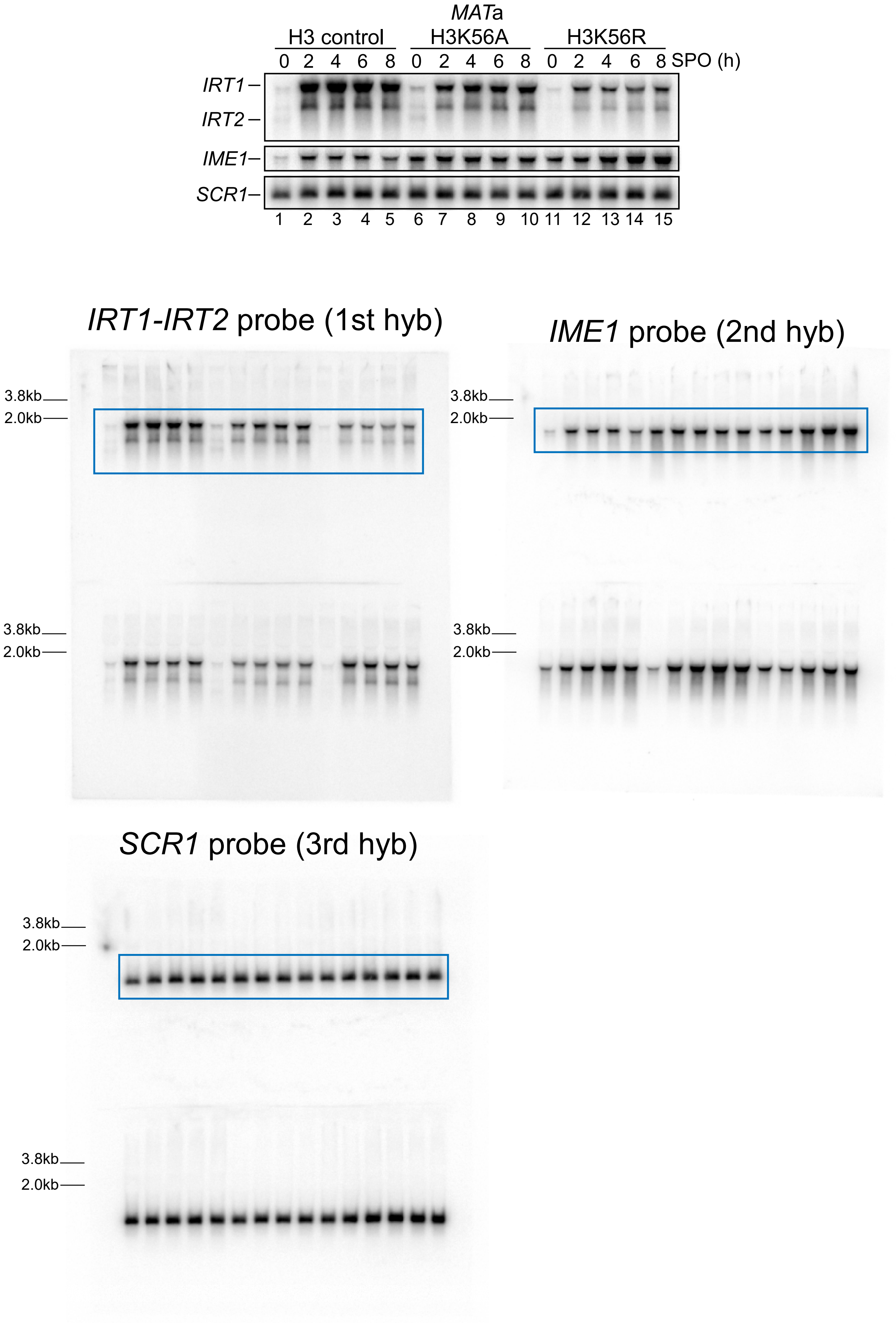
MATa
H3 control
H3K56A
H3K56R
0 2 4 6 8 0 2 4 6 8 0 2 4 6 8 SPO (h)
IRT1
IRT2
IME1
SCR1
1 2 3 4 5 6 7 8 9 10 11 12 13 14 15
IRT1-IRT2 probe (1st hyb)
IME1 probe (2nd hyb)
SCR1 probe (3rd hyb)
3.8kb
2.0kb

Figure S1C

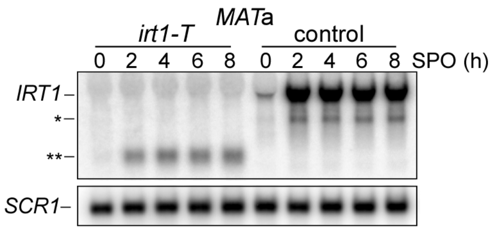

MATa
irt1-T
control
0 2 4 6 8 0 2 4 6 8 SPO (h)
IRT1
*
**
SCR1

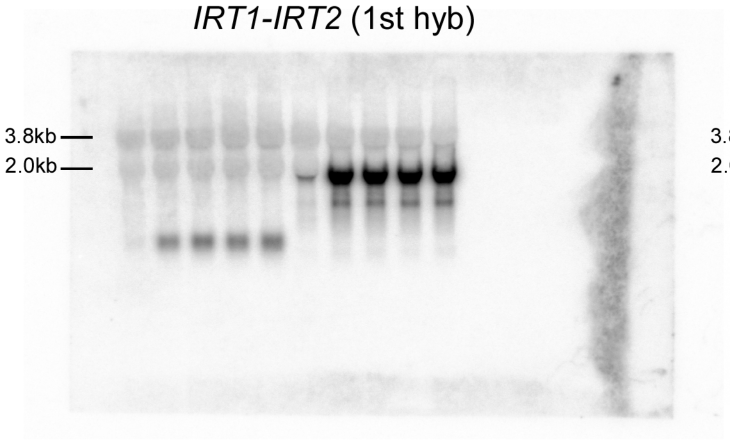

IRT1-IRT2 (1st hyb)
3.8kb
2.0kb

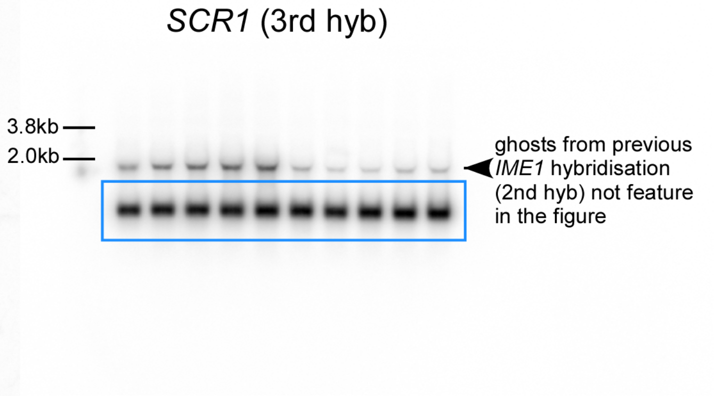

SCR1 (3rd hyb)
3.8kb
2.0kb
ghosts from previous IME1 hybridisation (2nd hyb) not feature in the figure

Figure S2B

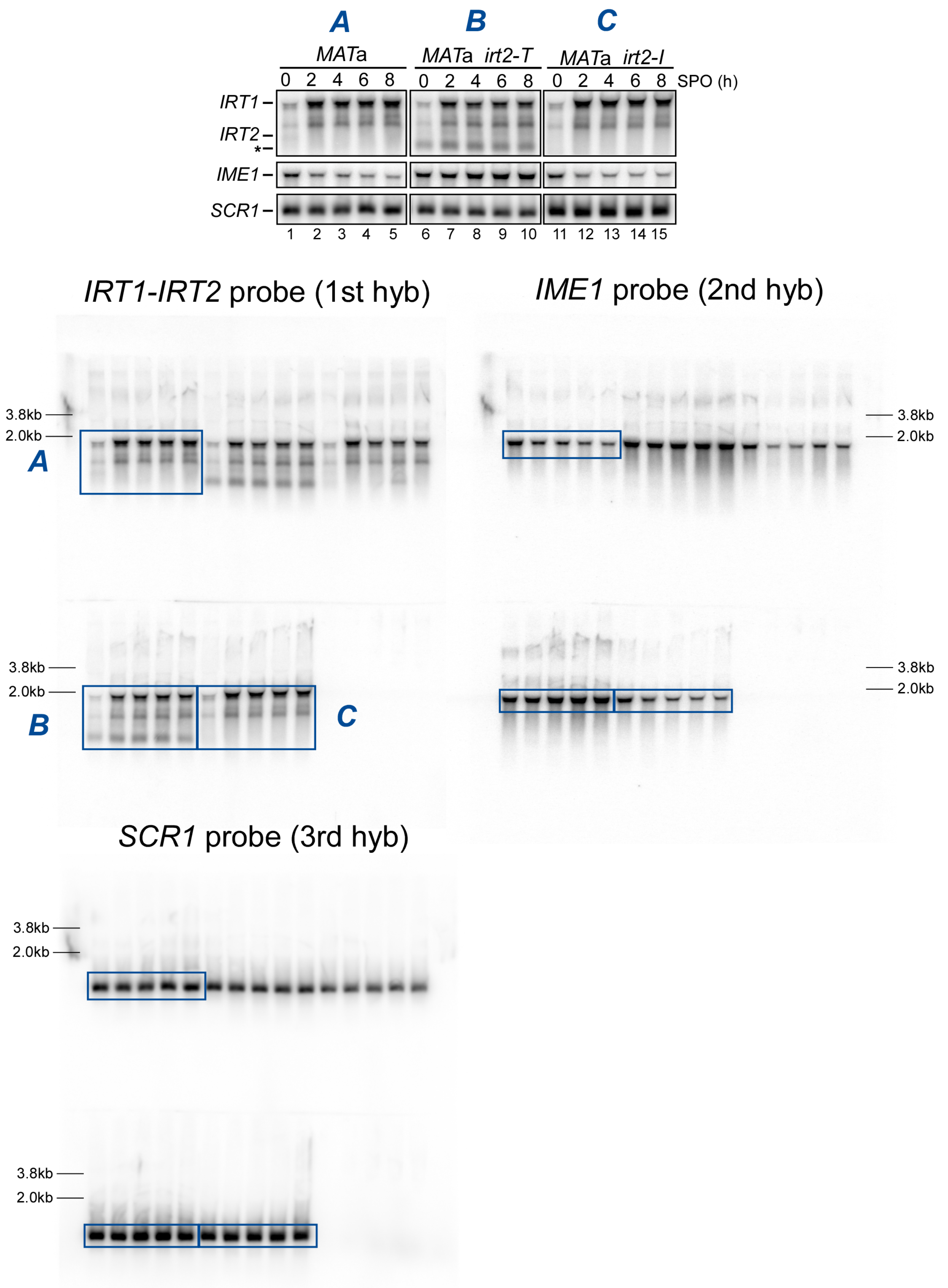
A
B
C
MATa
MATa irt2-T
MATa irt2-I
0 2 4 6 8
0 2 4 6 8
0 2 4 6 8
SPO (h)
IRT1
IRT2
*
IME1
SCR1
1 2 3 4 5 6 7 8 9 10 11 12 13 14 15
IRT1-IRT2 probe (1st hyb)
IME1 probe (2nd hyb)
SCR1 probe (3rd hyb)
3.8kb
2.0kb
A
B
C

Figure S2D

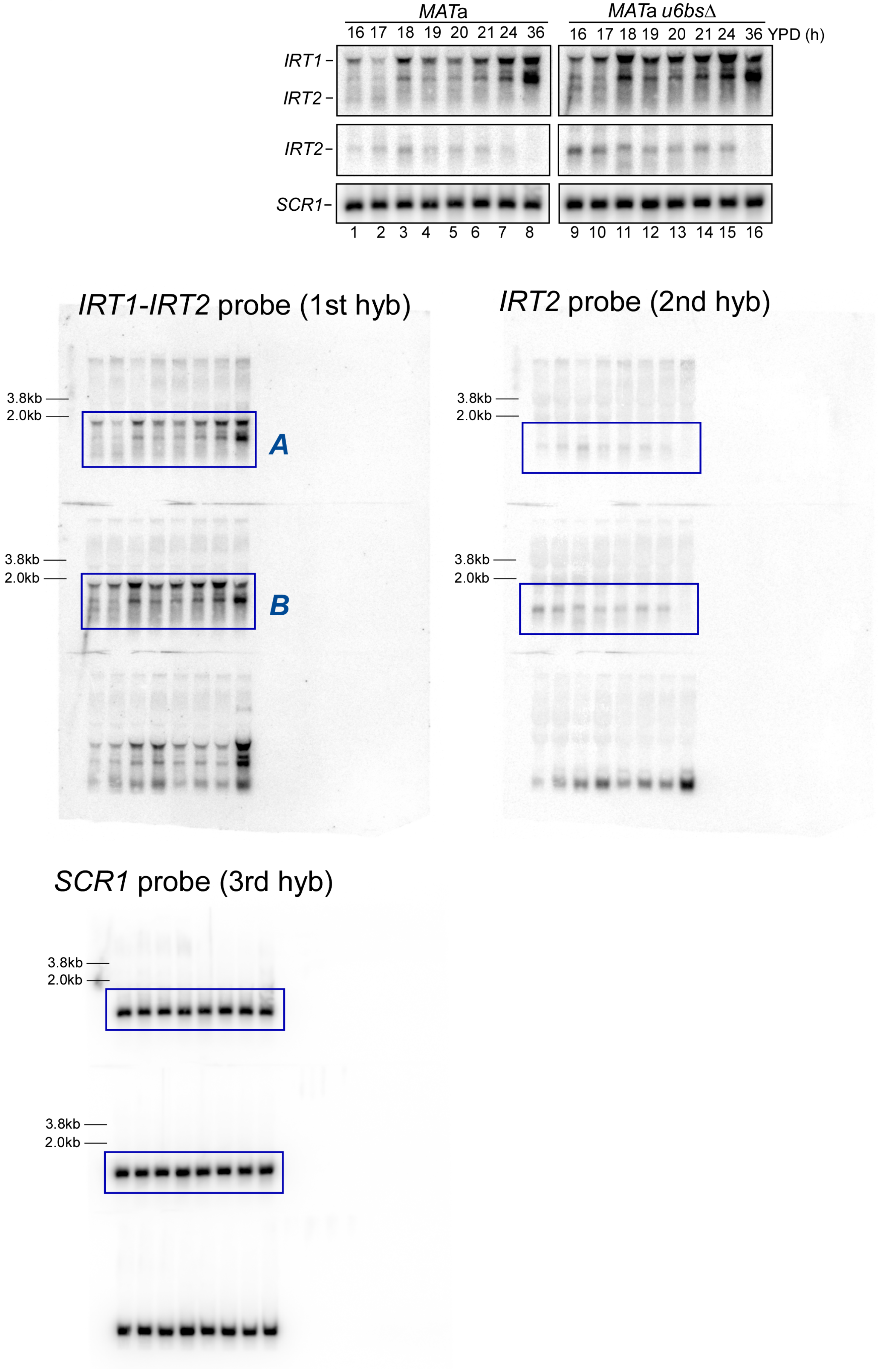
A
B
MATa
MATa u6bsΔ
16 17 18 19 20 21 24 36
16 17 18 19 20 21 24 36 YPD (h)
IRT1
IRT2
IRT2
SCR1
1 2 3 4 5 6 7 8
9 10 11 12 13 14 15 16
IRT1-IRT2 probe (1st hyb)
IRT2 probe (2nd hyb)
3.8kb
2.0kb
A
B
SCR1 probe (3rd hyb)

Figure S2F

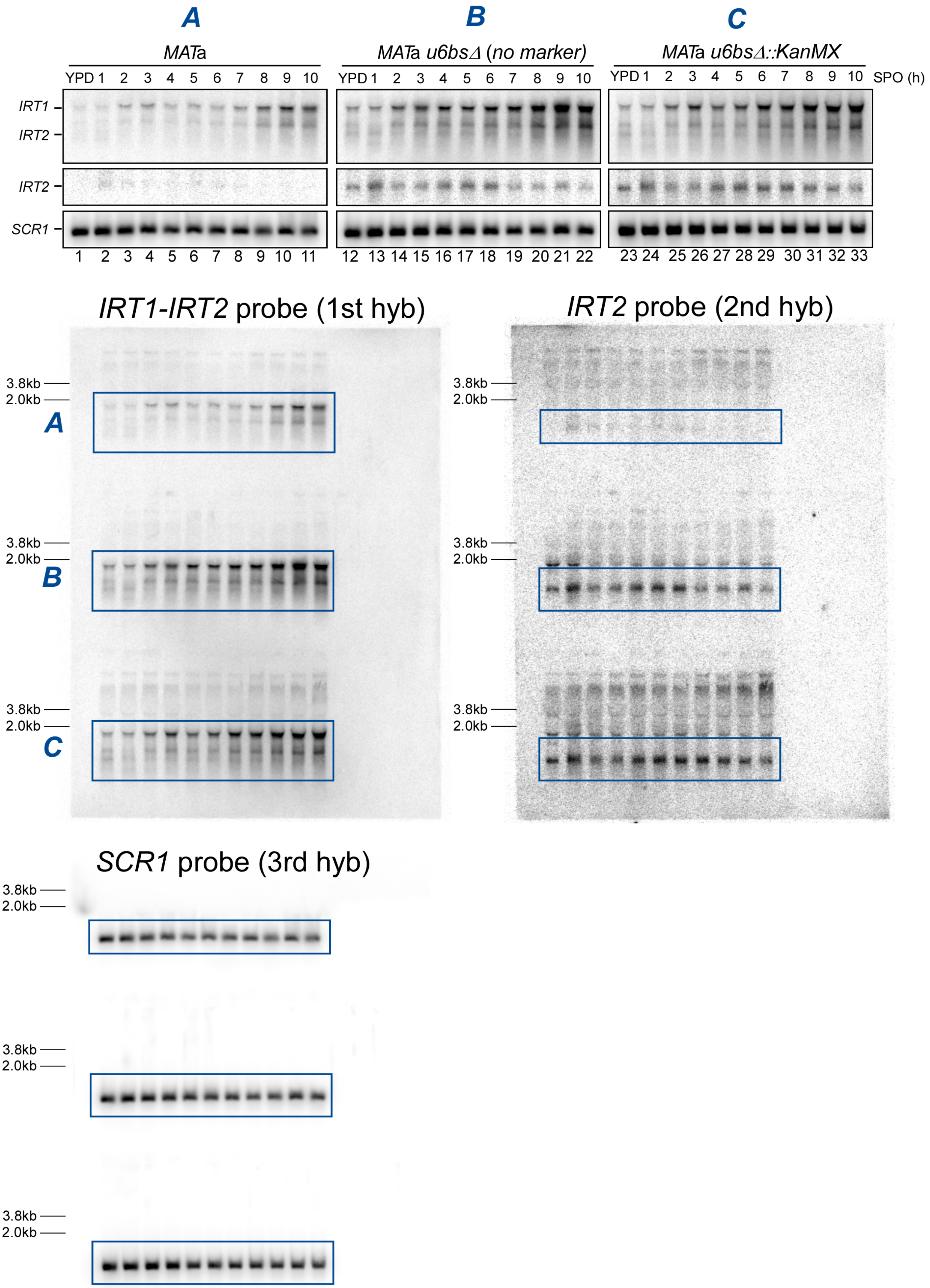

A
B
C
MATa
MATa u6bsΔ (no marker)
MATa u6bsΔ::KanMX
YPD 1 2 3 4 5 6 7 8 9 10
YPD 1 2 3 4 5 6 7 8 9 10
YPD 1 2 3 4 5 6 7 8 9 10
SPO (h)
IRT1
IRT2
IRT2
SCR1
1 2 3 4 5 6 7 8 9 10 11
12 13 14 15 16 17 18 19 20 21 22
23 24 25 26 27 28 29 30 31 32 33
IRT1-IRT2 probe (1st hyb)
IRT2 probe (2nd hyb)
SCR1 probe (3rd hyb)
3.8kb
2.0kb

Figure S2H

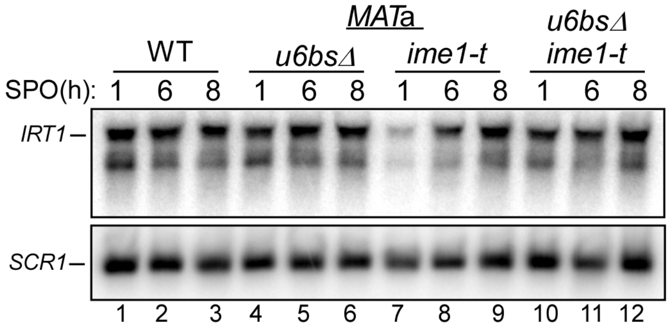

IRT1-IRT2 probe (1st hyb)

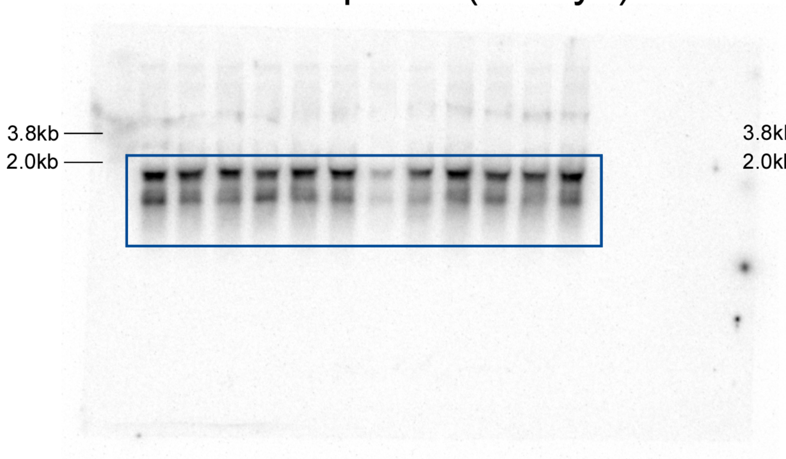

SCR1 probe (2nd hyb)

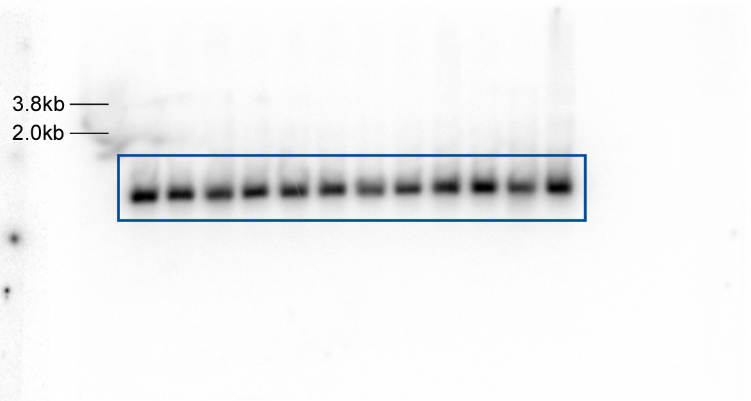

Figure S2I

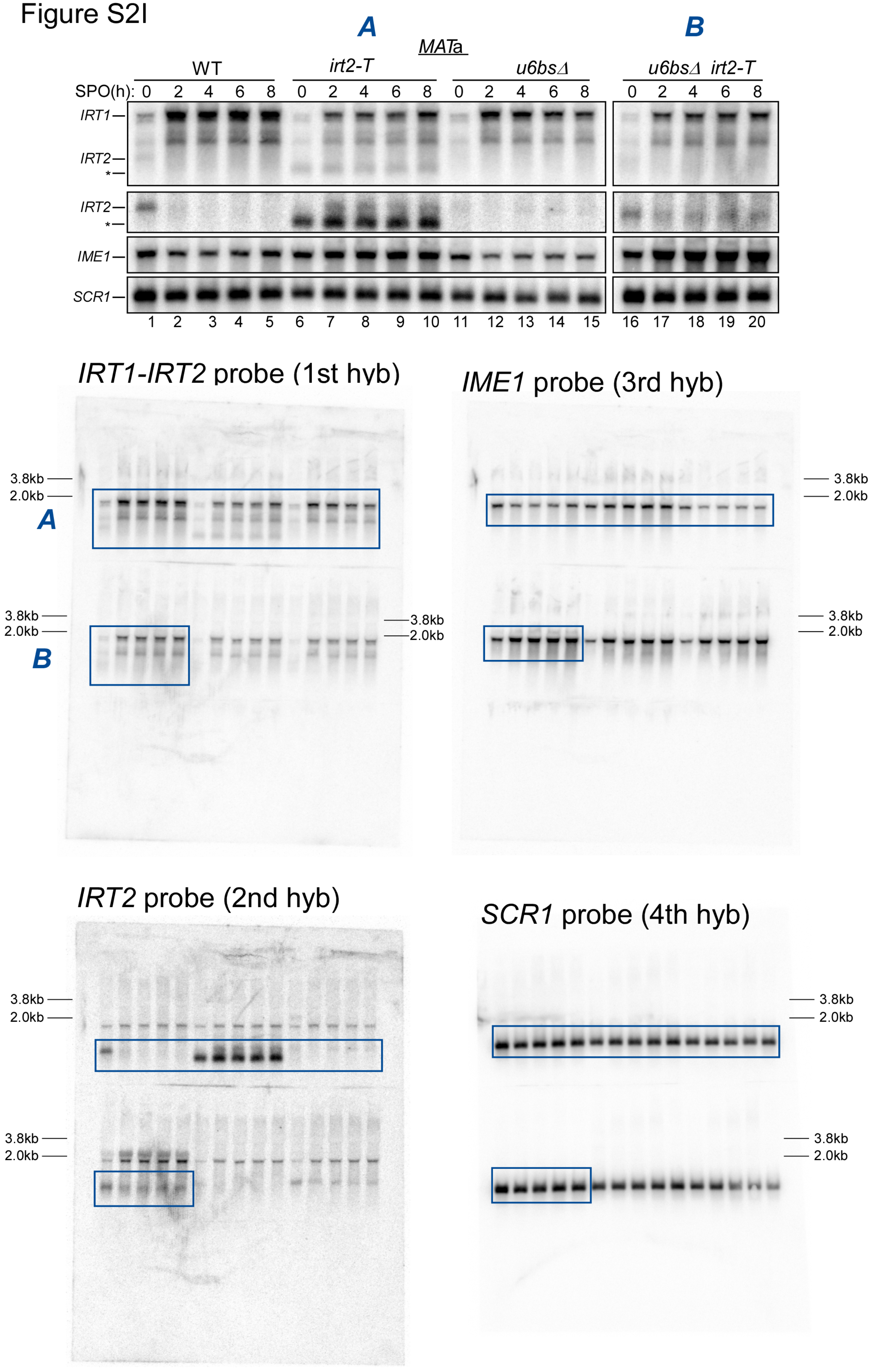

A
B
MATa
WT
irt2-T
u6bsΔ
u6bsΔ irt2-T
SPO(h): 0 2 4 6 8 0 2 4 6 8 0 2 4 6 8 0 2 4 6 8
IRT1
IRT2
*
IRT2
*
IME1
SCR1
1 2 3 4 5 6 7 8 9 10 11 12 13 14 15 16 17 18 19 20
IRT1-IRT2 probe (1st hyb)
IME1 probe (3rd hyb)
IRT2 probe (2nd hyb)
SCR1 probe (4th hyb)
3.8kb
2.0kb

Figure S2J

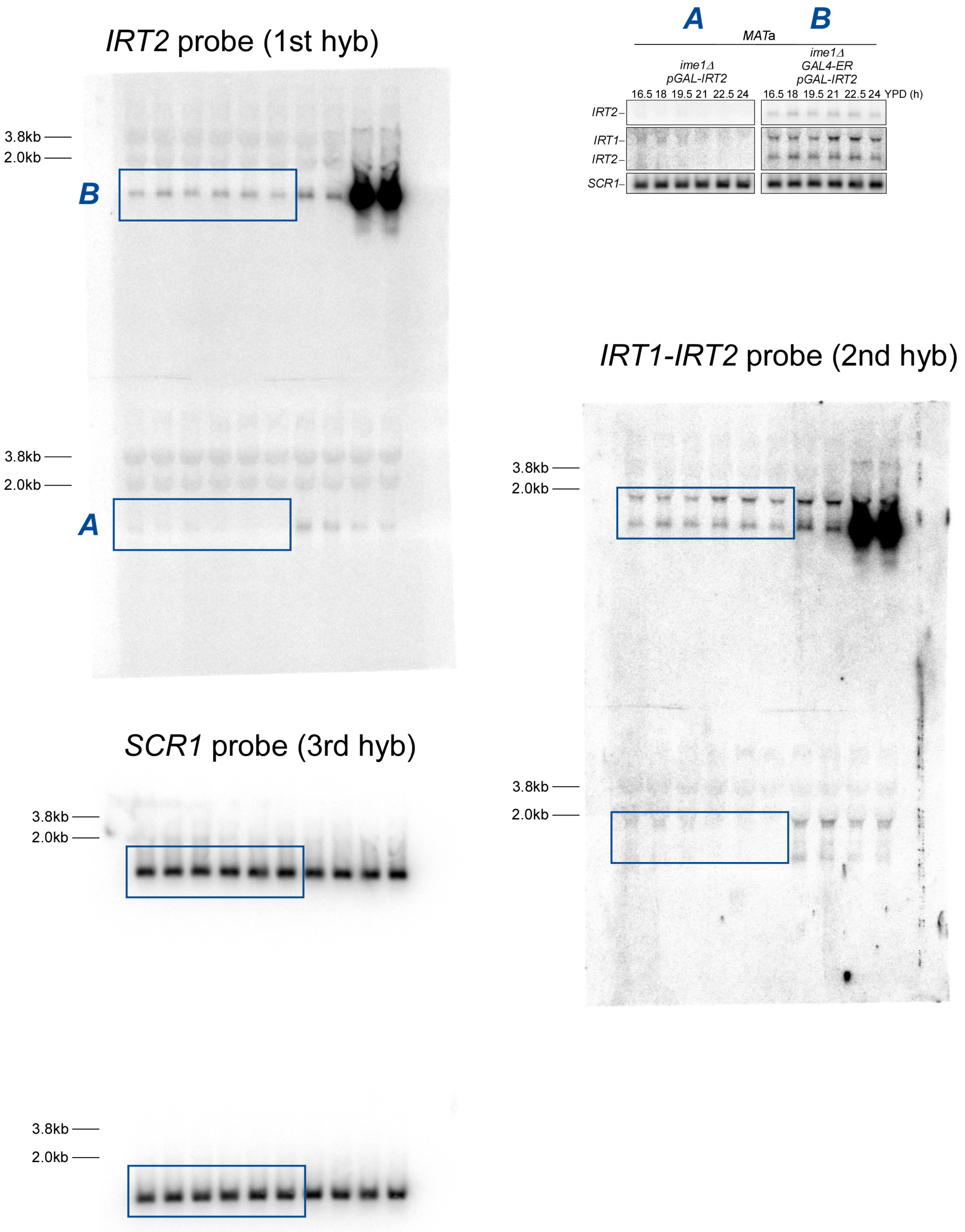
A
B
MATa
ime1Δ
pGAL-IRT2
ime1Δ
GAL4-ER
pGAL-IRT2
16.5 18 19.5 21 22.5 24
16.5 18 19.5 21 22.5 24 YPD (h)
IRT2
IRT1
IRT2
SCR1
IRT2 probe (1st hyb)
3.8kb
2.0kb
B
3.8kb
2.0kb
A
IRT1-IRT2 probe (2nd hyb)
3.8kb
2.0kb
3.8kb
2.0kb
SCR1 probe (3rd hyb)
3.8kb
2.0kb
3.8kb
2.0kb

Figure S4A

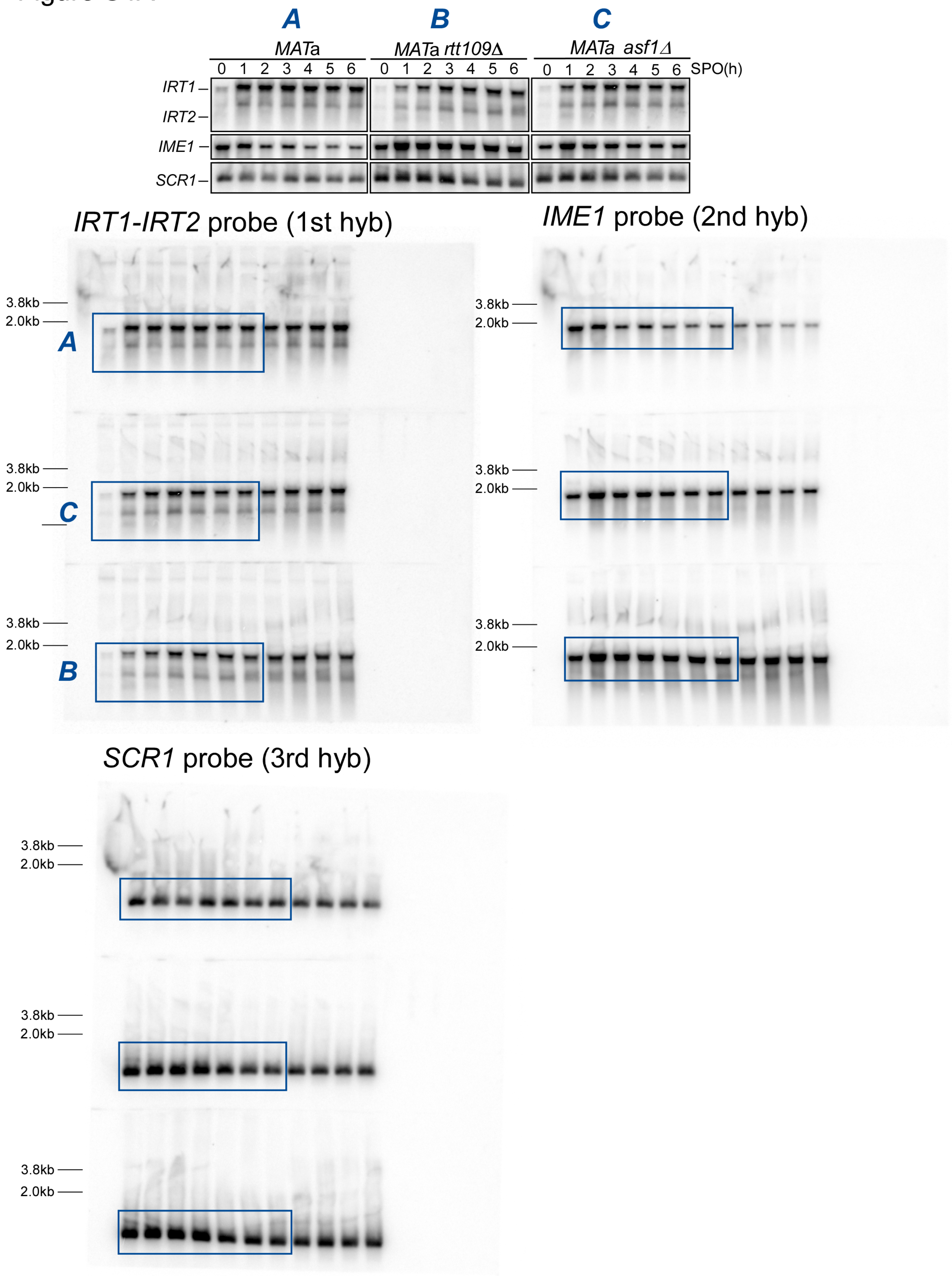
A
B
C
MATa
MATa rtt109Δ
MATa asf1Δ
0 1 2 3 4 5 6
SPO(h)
IRT1
IRT2
IME1
SCR1
IRT1-IRT2 probe (1st hyb)
IME1 probe (2nd hyb)
SCR1 probe (3rd hyb)
3.8kb
2.0kb

Figure S5C

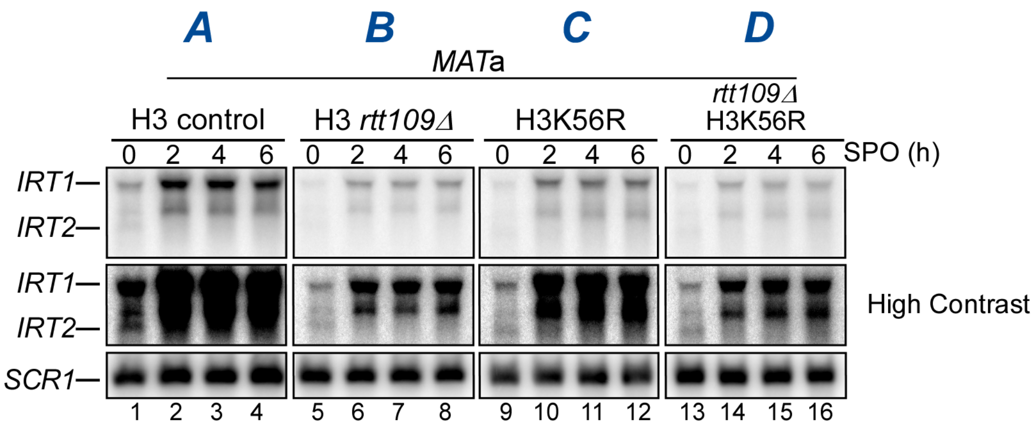

IRT1-IRT2 probe (1st hyb)

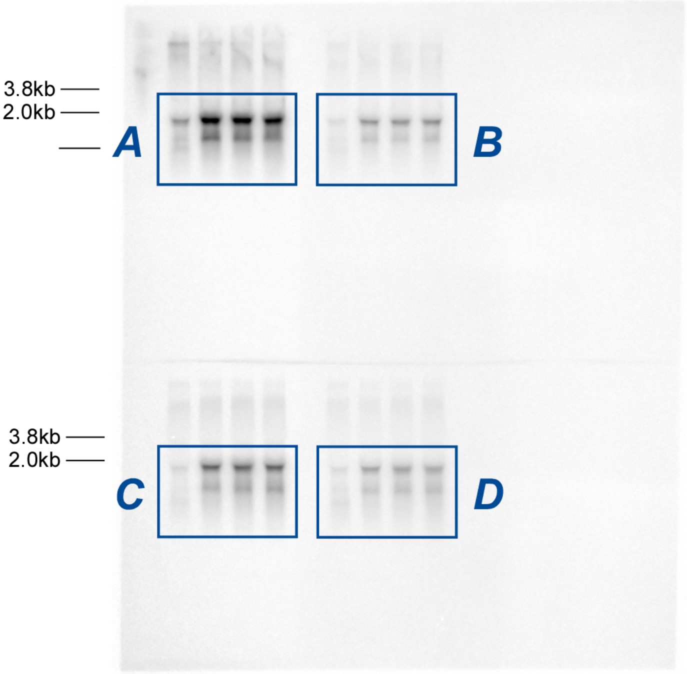

SCR1 probe 2nd hyb)

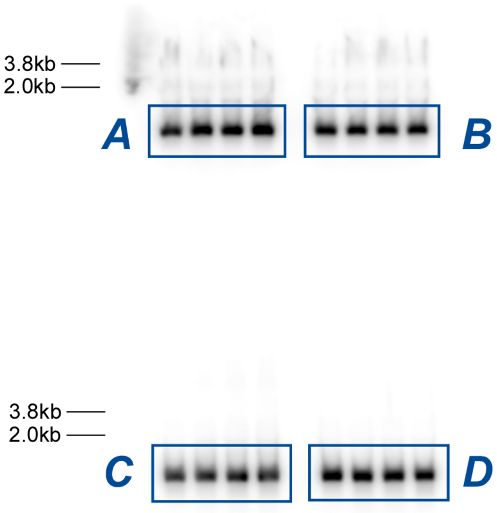

Figure S6C

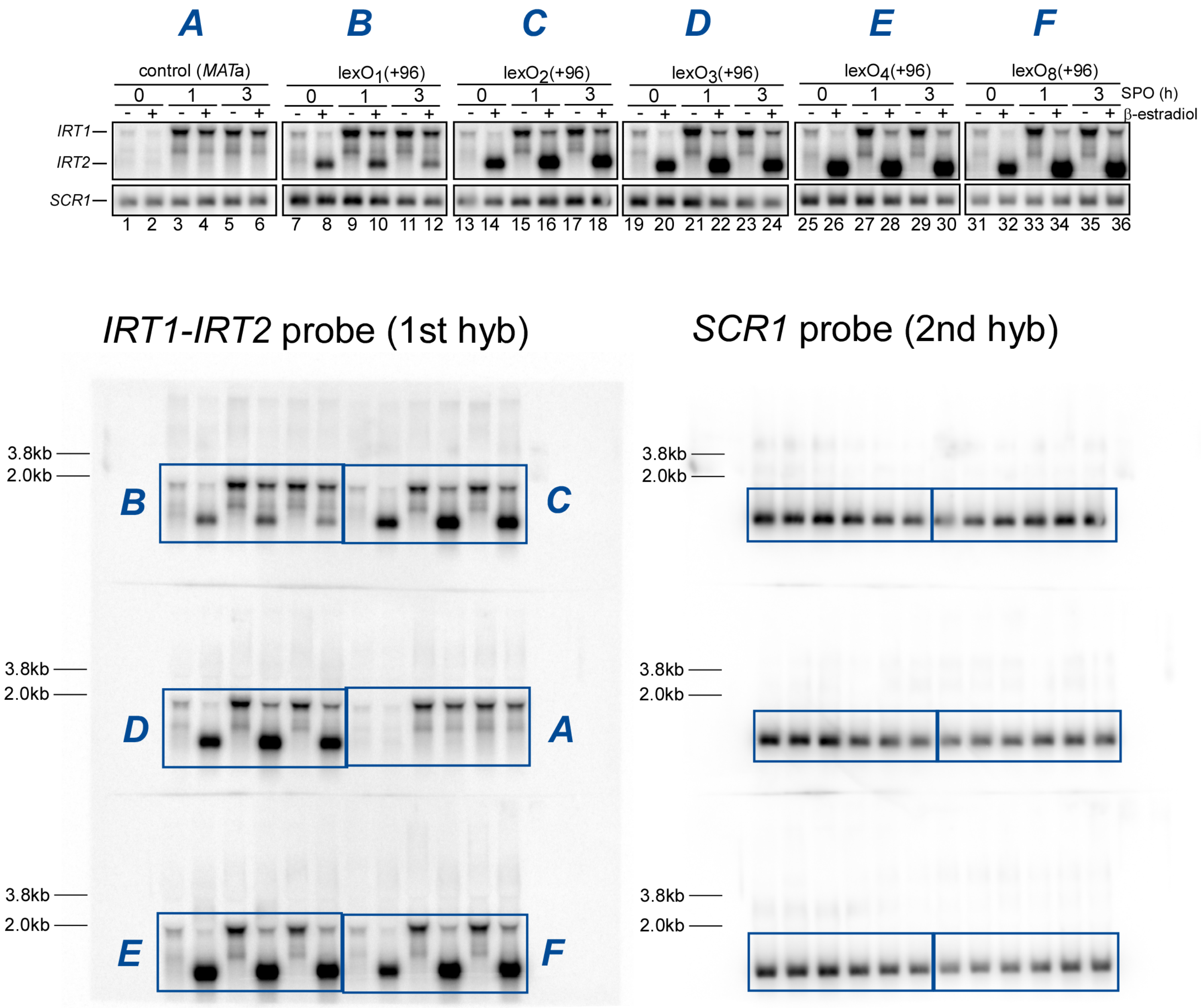

A
B
C
D
E
F
control (MATa)
lexO1(+96)
lexO2(+96)
lexO3(+96)
lexO4(+96)
lexO8(+96)
0 1 3
SPO (h)
- + - + - +
β-estradiol
IRT1
IRT2
SCR1
1 2 3 4 5 6 7 8 9 10 11 12 13 14 15 16 17 18 19 20 21 22 23 24 25 26 27 28 29 30 31 32 33 34 35 36
IRT1-IRT2 probe (1st hyb)
SCR1 probe (2nd hyb)
3.8kb
2.0kb
